# Phylogenomic Analysis of Marine *Roseobacters*

**DOI:** 10.1371/journal.pone.0011604

**Published:** 2010-07-15

**Authors:** Kai Tang, Hongzhan Huang, Nianzhi Jiao, Cathy H. Wu

**Affiliations:** 1 State Key Laboratory of Marine Environmental Science, Xiamen University, Xiamen, China; 2 Protein Information Resource (PIR), Georgetown University, Washington, D. C., United States of America; 3 Center for Bioinformatics and Computational Biology, University of Delaware, Newark, Delaware, United States of America; University of Maryland, United States of America

## Abstract

**Background:**

Members of the *Roseobacter* clade which play a key role in the biogeochemical cycles of the ocean are diverse and abundant, comprising 10–25% of the bacterioplankton in most marine surface waters. The rapid accumulation of whole-genome sequence data for the *Roseobacter* clade allows us to obtain a clearer picture of its evolution.

**Methodology/Principal Findings:**

In this study about 1,200 likely orthologous protein families were identified from 17 *Roseobacter* bacteria genomes. Functional annotations for these genes are provided by iProClass. Phylogenetic trees were constructed for each gene using maximum likelihood (ML) and neighbor joining (NJ). Putative organismal phylogenetic trees were built with phylogenomic methods. These trees were compared and analyzed using principal coordinates analysis (PCoA), approximately unbiased (AU) and Shimodaira–Hasegawa (SH) tests. A core set of 694 genes with vertical descent signal that are resistant to horizontal gene transfer (HGT) is used to reconstruct a robust organismal phylogeny. In addition, we also discovered the most likely 109 HGT genes. The core set contains genes that encode ribosomal apparatus, ABC transporters and chaperones often found in the environmental metagenomic and metatranscriptomic data. These genes in the core set are spread out uniformly among the various functional classes and biological processes.

**Conclusions/Significance:**

Here we report a new multigene-derived phylogenetic tree of the *Roseobacter* clade. Of particular interest is the HGT of eleven genes involved in vitamin B12 synthesis as well as key enzynmes for dimethylsulfoniopropionate (DMSP) degradation. These aquired genes are essential for the growth of *Roseobacters* and their eukaryotic partners.

## Introduction

Members of the *Roseobacter* clade are diverse and abundant, comprising 10–25% of the bacterioplankton in most marine surface waters [1]–[3]. *Roseobacter* are usually aerobic mixotrophs that have adapted to occupy a wide variety of marine ecological niches. Members of the *Roseobacter* lineage are involved in aerobic anoxygenic photosynthesis, dimethylsulfoniopropionate (DMSP) degradation, and CO utilization in marine surface waters [2]. Among them, aerobic anoxygenic phototrophic bacteria are a group of heterotrophic bacteria with the capability of phototrophy that appear to have a particular role in the ocean's carbon cycling [4], [5]. Thus, they could have a large impact on the cycling of carbon and other important nutrients in the oceans.

Given the importance of *Roseobacters* in biogeochemical cycles of the ocean, their well-characterized genome sequences [6] within a clade, and global abundance, the marine *Roseobacter* clade is ideal for elucidating bacterial diversification and adaptation to ocean environments. Currently, the rapid accumulation of bacterial whole-genome sequence data for *Roseobacter* [6] prompted us to investigate *Roseobacter* evolution from a genomic perspective.

To study the evolution of bacteria, it is important to distinguish between vertical and non-vertical phylogenetic signals; the latter will affect the inference of phylogenetic relationships. Single-gene phylogenies are generally poorly resolved due to the limited number of informative positions and random noise [7]. Phylogenomics based on large multigene data sets not only provide more accurate phylogenetic resolution than single-gene phylogeny but also can be used to reconstruct genome-scale events [8], [9] such as horizontal gene transfer (HGT). HGT is now known to be a major force in bacterial metabolic, physiological and ecological evolution and in shaping the genome [10]–[13]. More and more studies are revealing possible cases of gene transfers between bacteria [14]–[16]. The recent discovery of plasmids in *Roseobacter* strains opens up the possibility that horizontal gene transfer may be common between the *Roseobacter* populations [2]. Furthermore, there is a recent report of gene transfer agent mediated gene transfer in the natural populations of *Roseobacter* [15]. Whole-genome phylogeny has the potential to detect HGT [17]. Three different approaches for phylogenomic analysis have been proven useful: consensus trees, concatenated sequences and supertrees [17].

Here we identify a core gene set by first selecting a set of probable ortholog families and then reconstructing the organismal phylogeny for the *Roseobacter* clade. We examine the impact of HGT on the *Roseobacter* clade. A major implication of our results is that HGT is common among the *Roseobacter* clade. A consequence is that vitamin B12 biosynthesis and DMSP degradation genes acquired by HGT possibly contribute to the interactions of the *Roseobacter* clade bacteria with phytoplankton.

## Results and Discussion

### Orthology identification

The G + C content in the seventeen organisms is relatively similar (ranging from 54% to 66%; Table 1), but the genome size (from 3.5 to 5.3 Mb) and the number of protein coding genes per genome (from 3,656 to 5,495) are much more variable. There are 4,844 clusters of proteins present in at least four of the genome, in which 3,795 single-copy gene clusters were found. Carbon monoxide dehydrogenase was found in all species. Only 7 organisms possessed photosynthetic genes (34 genes) and all organisms possessed at least one DSMP degradation gene (*ddd*L and *dmd*A). A total of 1,295 putatively orthologous protein families across all 17 species was generated, with 1,197 of these containing only a single gene from each genome. Although it has been shown that only about 206 and 684 orthologous proteins are shared by 13 Gamma-Proteobacteria species and 13 cyanobacteria species, respectively [18], [19], our results indicate that many gene families are conserved among *Roseobacters*. In our study, the 1,197 single-copy genes (Table S1) representing likely orthologs were designated as candidates for inferring the organismal phylogeny to minimize the risk of reconstruction artifacts due to hidden paralogy.These orthologous groups annotated according to the COG database are spread out among the various functional classes, as shown in Table S2. The orthologs with GO term annotation in iProClass [20] reveal the frequencies of gene families involved in different biological processes or with distinct biochemical functions (Figure S1 and Figure S2). Most of these genes from various cellular components (Figure S3) are important because of their central roles in essential metabolic pathways or cellular functions (Figure S1 and Figure S2). The largest functional group contains 48 orthologous families and corresponds to the ribosomal protein family. The second-largest group, with 36 families, corresponds to the ABC transporter family.

**Table 1:** Genome sizes, GC contents, protein number and biogeochemistry related genes of *Roseobacter* clade organisms.

Abbr	Genome	Size (Mb)	(G+C) %	Protein coding genes	CO utilization*	Dimethylsulfoniopropionate degradation†			Phototrophy‡
					*CODH*	*dmdA*	*dddL*	*dddD*	Photosynthetic genes
DSH	*Dinoroseobacter shibae* DFL12	4.3	65	4166	√	√	√	√	√
JAN	*Jannaschia* sp. CCS1	4.4	62	4283	√	√			√
LVE	*Loktanella vestfoldensis* SKA53	4.3	65	4166	√		√		√
OAN	*Octadecabacter antarcticus* 307	4.9	54	5495	√	√			
OBA	*Oceanicola batsensis* HTCC2597	4.4	66	4212	√		√		
OIN	*Oceanibulbus indolifex* HEL-45	4.1	59	4153	√	√			
PGA	*Phaeobacter gallaeciensis* BS107	4.2	59	4059	√	√			
RCC	*Roseobacter* sp. CCS2	3.5	55	3696	√	√			√
RDE	*Roseobacter denitrificans* Och114	4.1	58	3946	√	√	√		√
RGR	*Ruegeria* sp. R11	3.8	59	3656	√	√			
RHB	*Rhodobacterales bacterium* HTCC2654	4.5	64	4712	√		√		
RLO	*Roseobacter litoralis* Och 149	4.7	57	4746	√	√	√		√
ROS	*Roseovarius* sp 217	4.8	60	4772	√	√			√
SIL	*Silicibacter* sp. TM1040	4.2	60	3864	√	√			
SPO	*Silicibacter pomeroyi* DSS-3	4.6	64	4283	√	√	√	√	
SSE	*Sagittula stellata* E-37	5.3	65	5067	√			√	
SUL	*Sulfitobacter* sp. EE-36	3.5	60	3474	√		√		

(√ means gene exists).

*The *CODH* gene encodes carbon monoxide dehydrogenase, which is the biological catalyst for reversible oxidation of CO to CO_2_ with water as the source of oxygen.

† The *dddL* gene encodes dimethylsulfoniopropionate lyase involved in dimethylsulfoniopropionate (DMSP) degradation I (cleavage) and the *dddD* gene encodes dimethylsulfoniopropionate a CoA transferase involved in DMSP degradation I (cleavage). A *dmdA* gene encoding dimethylsulfoniopropionate demthylase may participate in DMSP degradation III (demethylation). (Information from http://metacyc.org/).

‡ Including genes encoding for light harvesting systems, reaction center and bacteriochlorophyll biosynthesis proteins (see Table S3).

### Phylogeny of orthologous proteins

**Figure 1:**
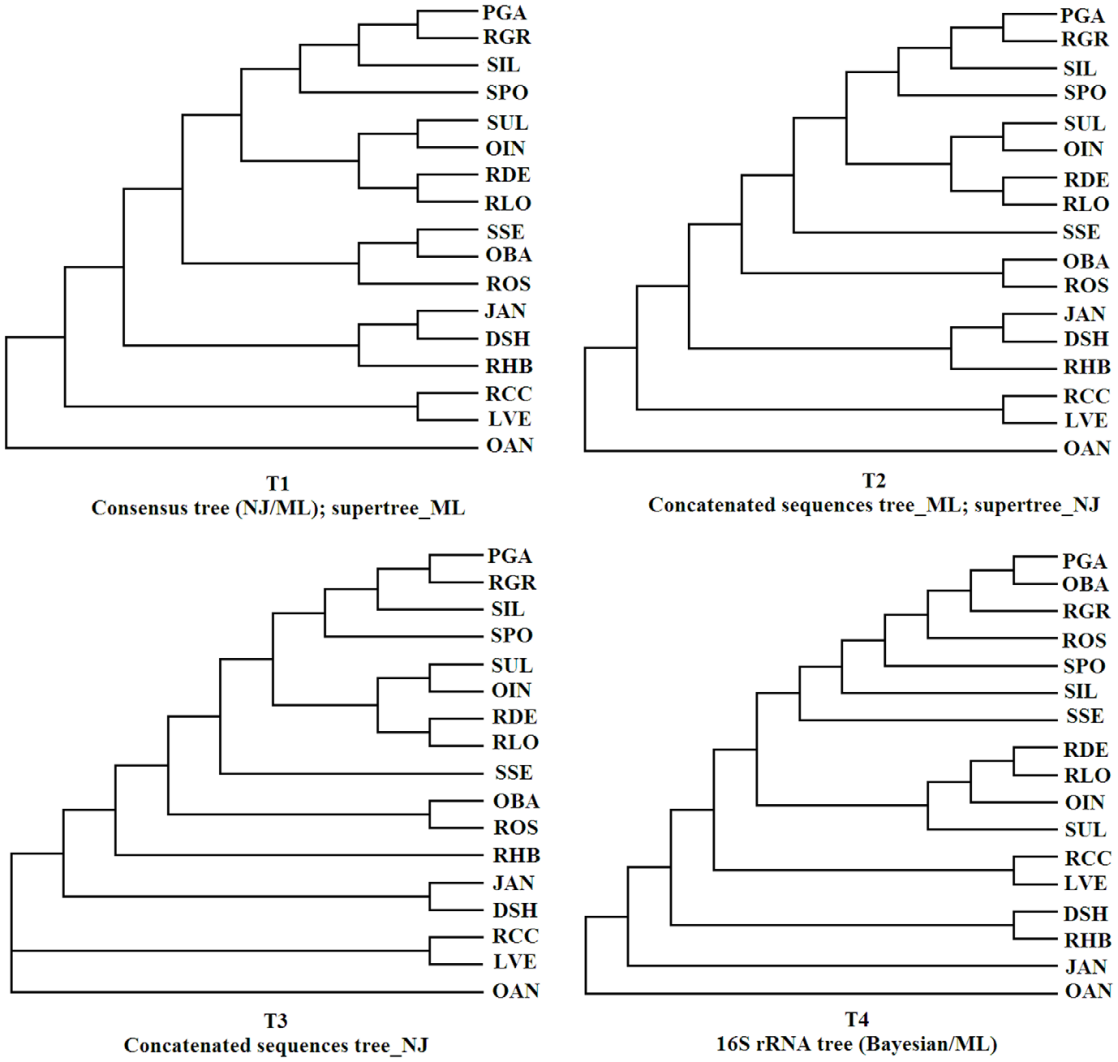
Representative backbone tree topologies. Phylogenetic trees were constructed by using both orthologous proteins through phylogenomic approaches and 16S rRNA gene (For details on evolutionary models and phylogenetic methods, see *Materials and Methods*). T1 corresponds to the consensus of 1,197 NJ or ML trees and the supertree made with ML trees. T2 corresponds to the concatenated sequences tree built with ML and the supertree constructed with NJ trees. T3 corresponds to the concatenated sequences tree inferred with NJ. T4 corresponds to 16S rRNA tree inferred with Bayesian or ML. Trees derived from the phylogenomic analysis of the conserved 694 core genes show the same topology T1.

A set of likely gene orthologs and alignments without uncertain sites by Gblocks, was used to produce single-gene phylogenies of the *Roseobacter* clade. Individual trees constructed by neighbor joining (NJ) and maximum likelihood (ML) are available upon request. By constructing trees based on several combinations of data using the different methodologies (see Materials and Methods), from single-gene to genome-scale phylogenies, we constructed a multigene-derived phylogenetic tree of the *Roseobacter* clade. As shown in Figure 1, these analyses produced a total of three topologies. Topology 1 (T1) corresponds to the consensus 1,197 phylogenic trees built by ML or NJ methods. The supertree constructed with ML also reached the same topology. Topology 2 (T2) was obtained from the 1,197 concatenated orthologous sequences by the ML method, which was identical to the supertree by the NJ method. Topology 3 (T3) corresponds to the concatenated trees by the NJ method. Topologies 1–3 were similar trees on a coherent phylogenetic pattern, they differ only with regard to the position of SSE and RHB (species abbreviations as in Table 1). On the other hand, ML and Bayesian 16s rRNA trees correspond to topology 4 (T4). There is unexpected conflict among T1–T3 and the tree based on the 16S rRNA sequences, which is the most frequently used phylogenetic analysis for evolution of microorganisms.

### Comparison of gene trees

**Figure 2:**
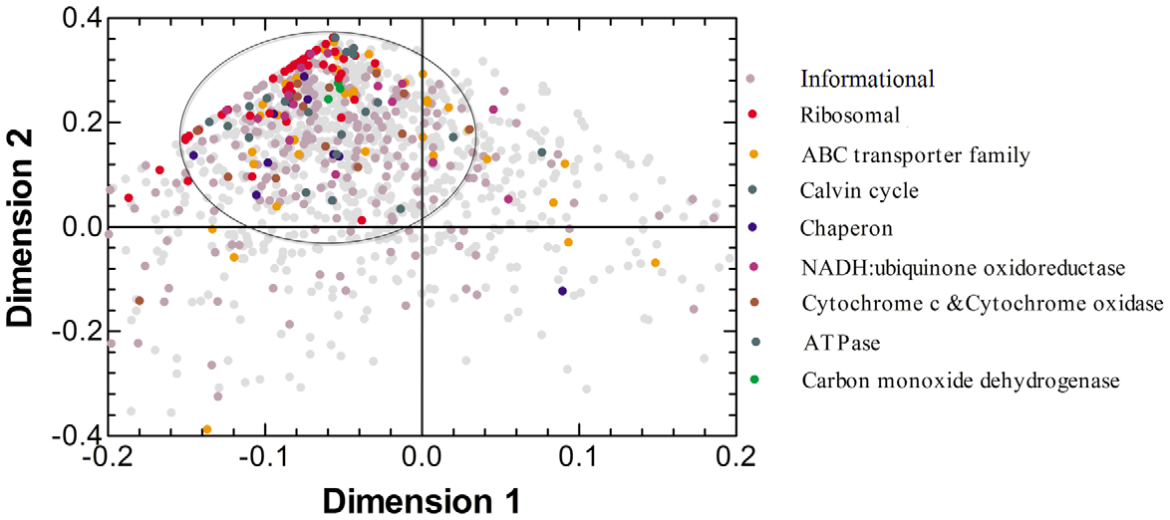
Plot of the two first axes of the principal coordinates analysis (PCoA) made from ML trees compared with Robinson and Foulds distance. The other 69 data points are outside the axis limits. The same experiment with NJ trees gave very similar results. Different genes are color coded based on their respective functions. For example, red dots correspond to genes coding conserved ribosomal proteins and other orange dots correspond to genes coding ABC transporter families that are present in the core. The ellipse depicts 868 orthologs in the densest region. The ellipse contains the 694 orthologs (core genes) retained through statistic tests for organismal tree reconstruction (see Table S1). The first (x) axis 1 expresses 46.1% of the total variation, and axis 2 represents 45.8% of the total variation.

In order to analyze the congruence among the gene trees above, we firstly measured topological similarity between trees based on the Robinson-Foulds distance. Figure 2 shows the extent of clustering similar topologies using principal coordinates (PCoA) analysis, suggesting a coherent phylogenetic signal within some genes. In all, there are 868 genes in a dense cloud on the two first axes of PCoA. Most informational genes, such as ribosomal genes, are present in the dense cloud of PCoA data. Some operational genes that mainly encode housekeeping functions also seem to be an essential component of this core. For example, many members of the ABC transporter family and highly conserved chaperones were found in this region. The cloud in this analysis reflects the high degree of congruence for *Roseobacter* gene trees based on a group of genes possessing similar topologies, indicating that a common evolutionary history is shared by many genes in *Roseobacters*. However, genes that retain both weak phylogenetic signal and noise from an HGT event could have some influence on our interpretation of this analysis since their tree topologies also might cluster in a dense cloud of PCoA [21].

To assess further potential conflict between trees, we used more sophisticated statistical methods (approximately unbiased (AU) and Shimodaira–Hasegawa (SH) tests). In these tests, each gene was compared against all the other gene trees and a usual value of p≤0.05 was used for the rejection of a given tree topology, suggesting that alignment reflects a non-vertical inheritance [22], [23]. The tree generated from carbamoyl-phosphate synthase (large subunit) had the largest number of genes supporting it (1101); other slightly different topologies were also in agreement with the majority of alignments (for T1–T3 agreement was with 1094, 1085, 1084 protein alignments). Although the T1 is the second largest supported tree topology by genes based on the SH test, T1 received the strongest support by 656 of 1197 protein alignments signal (SH p >0.95). Furthermore, T1 has the largest number of genes supporting it based on AU test. Thus, T1 obtained the best support from genes among all plausible topologies. There is thus a primary phylogenetic signal in the dataset that supports T1. By contrast, the 16S rRNA tree has the fewest number of genes supporting it (only 141 based on SH tests and 80 based on AU tests). The ribosomal RNA gene is not sufficiently informative to give a highly resolved and well-supported phylogeny for these taxa, probably due to little variance (with sequence similarities of 93.5% or up) or recombination events in the 16S rRNA within ecologically closely related organisms [24], [25].

### Core genes and organismal phylogenetic tree

The combination of SH and AU tests makes it possible to cover a core of genes that show predominantly vertical inheritance in *Roseobacter* clade organisms. This study defines a set of genes giving a consistent and main phylogenetic signal as putative core genes in *Roseobacter* clade based on AU and SH tests. The criterion for filtering core genes was that the gene alignment supports T1 (both of AU and SH p values >0.05). Most of genes in PCoA dense region in Figure 2 are core genes. Although PCoA analysis could not reveal the exact number of core genes and some wrong trees might affect it, PCoA is a complementary method to AU and SH tests for revealing functional clusters of core genes with the advantage of visualization.

The 694 putative core genes selected belong to various functional classes, as shown in Figure S1. This core includes numerous genes in both “informational” and “operational” functional categories (Figure S1). Certainly, some “real” core genes have been possibly filtered out of our analysis due to uncertainty in analytical methods (for example, statistics test outcome is dependent on the confidence level), however, the set of retained genes provided sufficient information for establishing the main phylogenetic signal to reconstruct a reliable organismal phlylogenetic tree. The topologies identical to T1 were recovered with a dataset of putative core genes through different phylogenetic reconstruction methods (ML and NJ). The tree based on “informational genes” of the putative core genes (the dataset of sequences assigned the categories J, K, and L from the COG database) or non-JKL sequences yielded exactly the same topology as T1. The tree using a concatenation of the best conserved ribosomal protein genes in the living world, constructed with each method, also reached the same topology.

To exclude, to the extent possible, erroneous inference arising from core gene choice, we further restricted our dataset to those 632 families for which each member supported at least one of T1–T3 (both of AU and SH p values >0.05) and the gene was clustered in a PCoA dense area as shown in Figure 2. The topologies obtained with this dataset were identical with those obtained with the putative core gene dataset for each method: the ML and NJ methods, supertree, consensus and concatenation recovered the topology T1 presented in Figure 1. Thus, T1 is a significantly more likely organismal phylogenetic tree than the alternative topologies (T2 and T3).Together, these results indicate that the true phylogenetic tree exists, and provide a good explanatory hypothesis basis for the evolution of the genes under study.

Our results from various selected dataset analyses strongly support the existence of a core of genes that has evolved mainly through vertical inheritance in *Roseobacters* and that carries a bona fide phylogenetic signal that can be used to retrace the evolutionary history of this organism. These core genes produce congruent phylogenies. The functions associated with the core genes are abundant in ocean metagenomics, metaproteomics and metatranstriptomics analysis [26]–[28]. For example, the ABC transporter systems and ribosomal proteins, chaperon GroEL, ATP synthase from OM43 clade and SAR11 were found as abundant proteins near the Oregon Coast [28]. Thus, the core contains the genes that provide essential biological functions for bacterial adaptation to ocean environments.

### Detection of horizontally transferred genes

Phylogenomic analyses detected some genes with strongly conflicting signal within the *Roseobacter* clade. Incoherence between the gene tree and the putative species tree can be the result of systematic errors (such as Long Branch Attraction (LBA)) [29], or of incorrect orthology (hidden paralogy or HGT) [30]. All phylogenetic and phylogenomic analyses recover a single clade for closely related taxa of bacteria, excluding distant species with significantly different evolutionary rates. Thus, phylogenetic incongruence is unlikely due to artifacts from LBA. This incongruence is not related to methodological problems and limitations since very similar results were obtained with NJ or ML methods. Hidden paralogy is rare in single-copy gene families selected as likely orthologs (orthology establishment) under application of the reciprocal hit criterion [31]. Thus, the observed conflicts could be due to gene transfers that occur within this clade or between *Roseobacter* and other phyla.

The 109 genes in Table S1 display statistically supported incongruence with the organismal phylogeny on both SH and AU tests (see alignments in File S1). Based on AU tests, 61 families out of 109 showed a conflict at the significance level of 0.0001 or less, 29 conflicts were found at the significance level of 0.01 and 19 conflicts at the significance level of 0.05. Operational genes seem to predominate among HGT genes, although several informational genes (e.g. tRNA synthetases) are included.

Several studies find that HGT is rare for single-copy orthologous proteins shared by all Gamma-Proteobacteria [18], [32], [33]. Only 1% (2 out of 206) of these orthologous genes are likely to be involved in HGT events, as indicated by the results of SH test [18]. In contrast, a recent study shows that at least 10% of these genes have been laterally transferred in Gamma-Proteobacteria using AU test combined with heatmap methods [21]. A few hundred HGT events in the set of orthologous genes from marine cyanobacteria were detected with PCoA or quartet phylogenies methods [19], [34]. Unfortunately, identifying all instances of HGT is quite difficult, and different methods of gene family selection, phylogenetic reconstruction, and HGT identification give contradictory results. Nevertheless, HGT may be more common among closely related bacteria than previously thought [35]–[37]. In this study, the estimation of HGT in gene families relies on two statistical tests. HGT is inferred when SH and AU tests supported phylogenetic incongruence. Indeed, our screening approach is conservative and likely to result in underestimating the total number of transfers. However, the result of HGT identification (109 out of 1197 sequences) supports the view that HGT occurs commonly in bacteria[35], [36].

The ML tree with the extended datasets reconstructed from a majority of data sets (around 76 out of 109) supports the groupings of *Roseobacter* with high BP values 80–100% (see File S2). However, twenty-two data sets lack support for the *Roseobacter* clade, showing other organisms within this group, or *Roseobacter* bacteria embedded within other phyla, indicating that possible transfer events to or from *Roseobacter* bacteria (see File S2).

**Figure 3:**
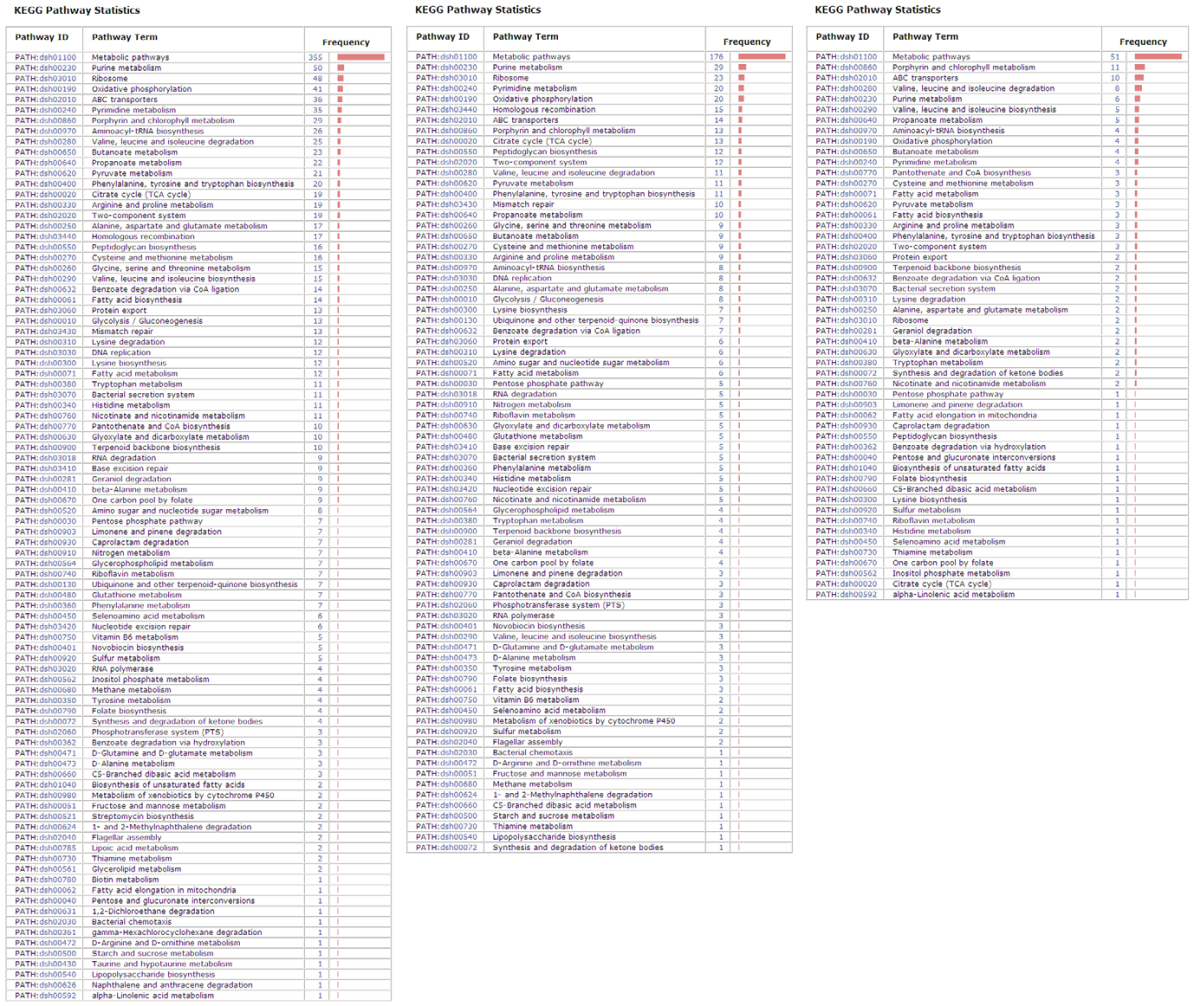
Functional classification of the genome representing the statistics for likely orthologous genes (*left*), core genes (*middle*) and HGT genes (*right*) KEGG term concerning metabolism. HGT genes are significantly enriched in “Porphyrin and chlorophyll metabolism”, while core genes are enriched in “Ribosome”. The ABC transporter families are rich in both HGT and core genes.

The majority of horizontally transferred genes are involved in metabolism as shown in Figure 3. It was noted that some of ABC transporter family genes and the key enzymes for valine, isoleucine, and leucine degradation genes were subjected to HGT. These genes provided potential for uptake and utilization of organic compounds in the *Roseobacter* clade. In particular, HGT genes are enriched for porphyrin and chlorophyll metabolism (Figure 3). These genes are involved in the cobalamin (coenzyme B12) biosynthetic pathway, and show significant conflict with the species tree since genes alignments should have very low p-values for AU and SH tests against the organismal phylogenetic tree(Table 2). Among bacteria, half of the sequenced B12-utilizing organisms lack the ability to synthesize B12 [38]. The *Roseobacter* strains are closely associated with diverse eukaryotic partners, e.g. algae [39]. Recent studies show that B12 synthesis contribute to not only to growth of *Roseobacter* clade bacteria but also to their interactions with marine algae in the nutrient-depleted environment, where B12 and cobalt are both found in exceedingly low concentrations [39]–[41]. In a mutualism relationship between algae and bacteria, the algae obtain the required vitamin B12 from bacteria and the metabolites they generate can serve as a consistent nutrient supply, including dissolved organic carbon (DOC) or DMSP, for the bacteria. Therefore, the genes involved in DMSP degradation also play a role in mutualistic interactions between *Roseobacter* strains and marine algae [42]. Phylogenetic analysis showed that the genes *ddd*L and *dmd*A that encode key enzymes in two principal DMSP degradation routes have undergone extensive lateral transfer (Table S3). These HGT events possibly promote mutuality relationships between the *Roseobacter* clade bacteria and phytoplankton. In summary, HGT can be beneficial for the *Roseobacter* clade competition for multiple nutrients in the natural planktonic bacterial community.

**Table 2:** Vitamin B12 biosynthetic genes p-values for AU and SH tests against species tree.

Protein family code	Gene name	Protein Name	p-SH	p-AU
ort477	*cobQ*	Cobyric acid synthase CobQ	<0.001	0.003
ort594	*cobB*	Cobyrinic acid a,c-diamide synthase	<0.001	5.0e-67
ort595	*cobK*	Precorrin-6× reductase; (EC = 1.3.1.54)	<0.001	4.0e-52
ort1114	*cobM*	Precorrin-4 C11-methyltransferase	<0.001	7.0e-07
ort1115	*cbiG*	Precorrin-3B C17-methyltransferase	<0.001	2.0e-06
ort1116	*cobI*	Precorrin-2 C20-methyltransferase	<0.001	0.001
ort1117	*cobL*	Precorrin-6y C5,15-methyltransferase (Decarboxylating), CbiE subunit; (EC = 2.1.1.132)	<0.001	3.0e-07
ort1118	*cobH*	Precorrin-8X methylmutase; (EC = 5.4.1.2)	<0.001	7.0e-47
ort1123	*cobW*	Cobalamin biosynthesis protein CobW	<0.001	1.0e-05
ort1124	*cobN*	Cobaltochelatase, CobN subunit; (in EC = 6.6.1.2)	<0.001	3.0e-95
ort1125	*cobA*	Cob(I)alamin adenosyltransferase; (EC = 2.5.1.17)	0.0003	7.0e-103

We have also analyzed photosynthetic genes from *Roseobacter* clade bacteria for phylogenetic relationship. No photosynthetic related genes conflict with the species tree (Table S3), indicating that they are immune to HGT among *Roseobacter* clade bacteria. Similarly, transfer of the key photosynthetic genes is very rare among closely related cyanobacterial strains [19]. The complexity of macromolecular interactions in complex photosynthetic machinery makes it difficult to transfer the essential components of photosynthesis to other prokaryotes [19].

## Materials and Methods

### Data collection

Seventeen genome sequences that are publicly available and are complete or nearly complete were downloaded from the National Center for Biotechnology Information (NCBI) database. The genomes used are shown in Table 1. The 16S rRNA was extracted from the integrated microbial genomes (IMG) database [43].

### Orthologous genes

Orthologous genes were identified using OrthoMCL (version 1.3) [44]. This program begins with an all versus all BLASTP [45] search performed on annotated genomes. The putative orthologous pairs were defined based on the reciprocal hit criterion and then analyzed with the program MCL, which utilizes Markov Clustering (MCL) by creating a similarity matrix from e-values and then clustering proteins into related families. OrthoMCL was run with a BLAST e-value cut-off of 1e-4, and an inflation parameter of 1.5. Protein families were constructed and only those that contained one representative per species were used for further study. Therefore, paralogs that could bias our analyses were discarded.

### Functional annotation of the gene families

Orthologous genes were functionally annotated using well-investigated *Dinoroseobacter shibae* DFL12 genome as a reference through iProClass at Protein Information Resources (PIR: http://pir.georgetown.edu) for the GO term or KEGG term [20]. Orthologous genes were assigned to functional categories using BLASTP against the COG database [46], choosing the category of the top-scoring BLAST hit. We used 18 specific functional categories and 4 general ones as defined in the COG database.

### Phylogenetic tree and consensus tree construction

1,197 proteins were identified that had one member in each of the 17 genomes. Detailed information is in Table S1. These 1,197 sets of orthologs were aligned independently with ClustalW [47]. Gblocks (version 0.91) [48] with the default settings was used to remove alignment regions that contain gaps or are highly divergent. The 1,197 protein sequence alignments were concatenated into a single alignment for phylogenetic inference. The resulting protein sequence alignment for each gene was used in the main phylogenetic analysis as described below.

Phylogenetic trees were inferred using maximum likelihood (ML) and neighbor joining (NJ) distance algorithms. ML-phylogenetic analyses were performed by PhyML (version 3.0) [49] with the Jones-Taylor-Thornton substitution (JTT) model, gamma distribution with eight categories plus invariant sites, and shape parameter and fraction of invariable sites estimated from each dataset. ProTest (version 2.4) [50] supported the use of JTT mixture model in this work (data not shown). NJ trees were constructed using NEIGHBOR in the PHYLIP package (version 3.6.2) [51] with species input order randomization enabled. The distance matrices were calculated by Tree-Puzzle (version 5.2) [52]. The parameters used in Tree-Puzzle were set to the JTT substitution model, the mixed model of rate heterogeneity with one invariant and eight gamma rate categories, and the exact and slow parameter estimation. One hundred bootstrap samples were generated using the SEQBOOT program [51]. The consensus tree was inferred by the CONSENSE program in the PHYLIP package using the extended majority rule [51]. Phylogenetic trees were visualized with TreeView [53].

### Tree comparison

The topological distances among phylogenetic trees were calculated based on the symmetric difference of Robinson and Foulds [54] as implemented in TREEDIST in the PHYLIP package [51]. Similarity relationships among phylogenetic trees were assessed by using principal coordinates analysis (PCoA), in which a distance matrix is used to plot the n trees in (*n*−1) dimensional space. On the *n*×*n* distance matrix obtained (*n* is the number of trees), a PCoA was conducted with the Ginkgo software. The Ginkgo interface returns information on all principal coordinate axes in the dataset, and then a multivariate dataset can be plotted as axes in two dimensions for visualization [55].

To test the significance of the differences between phylogenies derived from individual genes and the reference trees, the approximately unbiased (AU) test [22] and the Shimodaira–Hasegawa (SH) test [23] were performed. In these tests, different tree topologies are compared based upon the comparison of their log-likelihood values. Usually, an AU test p-value <0.05 is used for the rejection of a given tree topology. Site-wise likelihood values were computed by Tree-puzzle (JTT model, gamma distribution with eight categories plus invariant sites), and were subsequently used as inputs for CONSEL [22] with the default settings.

### 16s rRNA tree, concatenated trees and supertree constructions

The unambiguously aligned 16s rRNA sequences by Gblock (default parameters) were used to construct a phylogenetic tree using ML and Bayesian methods. The evolutionary model and corresponding parameters for the ML phylogeny inference analyses were chosen using Modeltest (version 3.7) [56]. The General Time Reversible model (GTR) + Invariable sites (I) + gamma (G) was selected as the best fitting model in ML and Bayesian analyses. The ML analysis of 16S rRNA gene sequences (100 bootstrap resampling) was done in PhyML. The Bayesian analysis was computed using MrBayes (version 3.1) [57] with four chains for 100,000 generations.

For the concatenated alignments of all individual genes or selected genes in this study, the maximum likelihood topology was obtained through RAxML [58] web servers using the JTT model with invariable sites. Concatenation trees were also built with PHYLIP using NJ methods. Trees chosen for the supertree computation were coded into a binary matrix using the “matrix representation using parsimony”(MRP) method as implemented in Clann software (version 2.0.2) [59]. The matrices obtained are concatenated into supermatrix. Supertrees are then generated from the supermatrix by the maximum parsimony technique using the program PAUP^*^ (version 4.0beta10).

### Extended phylogenetic analysis for HGT

We retained the candidate orthologs with the essential functional categories in the marine *Roseobacters*, including the photosynthetic genes and DMSP degradation genes (*ddd*L and *dmd*A). The trees inferred by ML and NJ were preformed as described above. Briefly, the proposed species trees comprising these taxa were generated based on the consensus of the ML or NJ individual gene trees, or on supertree computation procedures. These different approaches yielded the same topology. The protein sequence alignment from these special orthologs candidate was used for further SH and AU tests.

To detect inter-phylum HGT events between the *Roseobacter* and organisms from other phyla, we added highly homologous sequences from other phyla and reconstructed phylogenetic trees with the extended datasets. Homologous sequences to each *Roseobacter* clade data set were detected by performing BLASTP [45] similarity searches against the NCBI nr database with e-value cut-off of 1e-20 and only keeping the highest-scoring hit in main phyla (to reduce the computational time). The alignments for each identified extended gene family were created using the ClustalW [47] program. The alignments were filtered by Gblocks [48] using default settings to remove regions that contain gaps or are highly divergent. One hundred bootstrap samples were generated for e using SEQBOOT in PHYLIP [51], and were subsequently analyzed with PhyML [49] (JTT model, gamma distribution with eight categories plus invariant sites) and finally with CONSENSE [51] to generate an unrooted bootstrapped tree.

## Supporting Information

Figure S1 Functional classification of the genome representing the statistics for likely orthologous genes (*left*), core genes (*middle*) and HGT genes (*right*) based on their annotations to terms in the GO molecular function vocabularies.
(0.22 MB TIF)

Figure S2 Functional classification of the genome representing the statistics for likely orthologous genes (*left*), core genes (*middle*) and HGT genes (*right*) based on their annotations to terms in the GO biological process vocabularies.
(0.69 MB TIF)

Figure S3 Functional classification of the genome representing the statistics for likely orthologous genes (*left*), core genes (*middle*) and HGT genes (*right*) based on their annotations to terms in the GO cellular component.
(0.24 MB TIF)

Table S1 Gene information for phylogenetic and phylogenomic analyses (horizontal gene transfer candidates are colored green, core genes are colored blue and undefined genes are gray).
(0.42 MB XLS)

Table S2 Summary of the distribution of the COG functional categories of the likely orthologous genes in the *Roseobacter* clade.
(0.06 MB DOC)

Table S3 Photosynthetic genes and DMSP degradation genes p−values for AU and SH tests against species tree.
(0.07 MB DOC)

File S1 Protein sequence alignment of 109 HGT genes. Alignment was filtered by Gblocks to obtain reliable alignment regions for NJ or ML phylogenetic analyses.
(0.20 MB ZIP)

File S2 Tree topologies with the extended data. The multi-documents have been combined into a single ZIP-formatted file. The trees should be considered unrooted. The tree topologies were calculated in PhyML as described in Methods. Numbers refer to bootstrap values. The tree topology (separate pdf) shows that *Roseobacter* bacteria form a monophyletic group and was deposited in a document named “high bootstrap”. The other organisms embedded within the *Roseobacter* clade, or *Roseobacter* bacteria embedded within other phyla are shown in red (deposited in a document named “inter-phylum”). Individual file name corresponds to gene family code listed in Table S1. The non *Roseobacter* organism taxonomic name is detailed in the amino acid fasta of the sequences (a document named “sequences”).
(4.62 MB ZIP)
