# Supplementary figures and images for: Phylogenomic Analysis of Marine Roseobacters (part 1 of 2)
Source: PLoS One. 2010 Jul 15;5(7):e11604. doi: 10.1371/journal.pone.0011604 (PMC2904699; doi:10.1371/journal.pone.0011604)

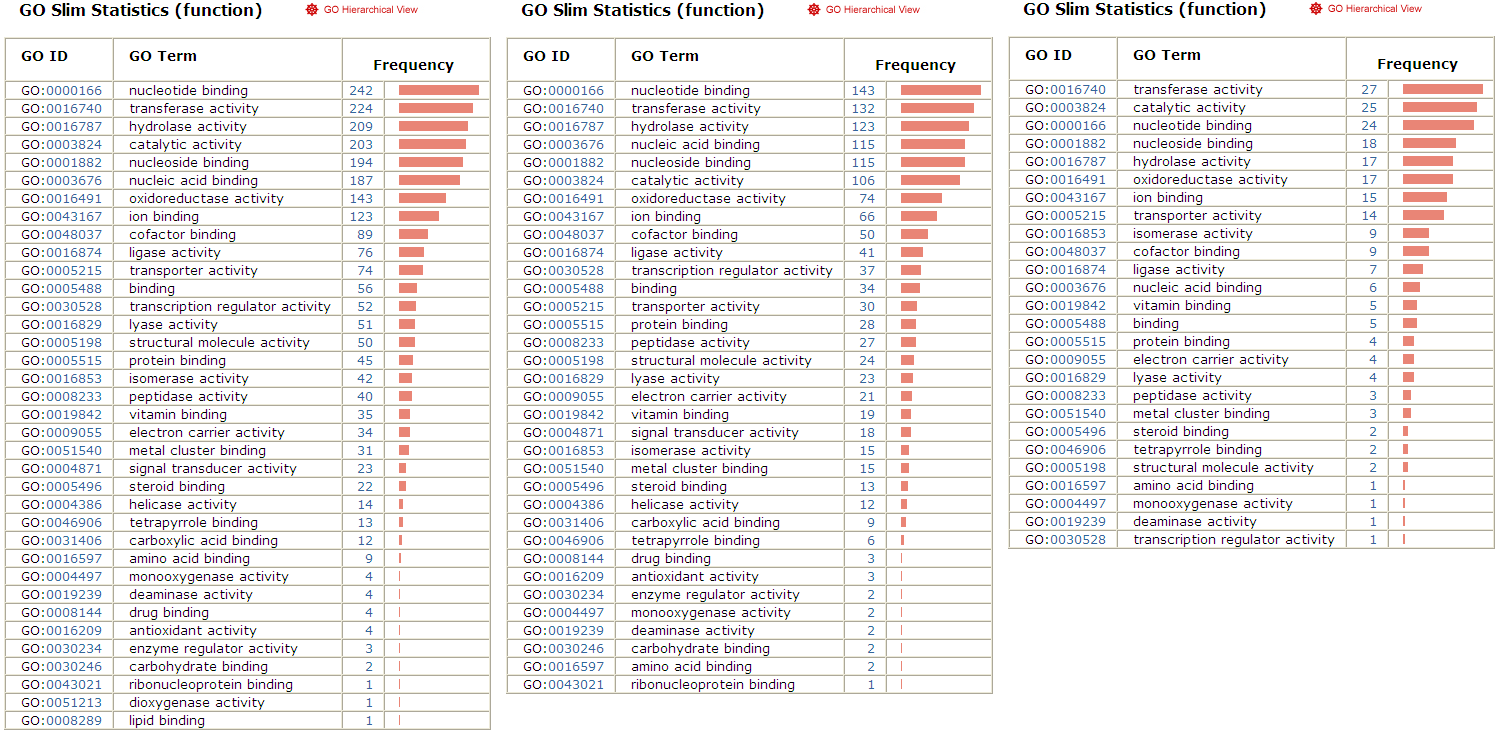

Supplement: Figure S1 — Functional classification of the genome representing the statistics for likely orthologous genes (left), core genes (middle) and HGT genes (right) based on their annotations to terms in the GO molecular function vocabularies. (0.22 MB TIF) [file pone.0011604.s001.tif]

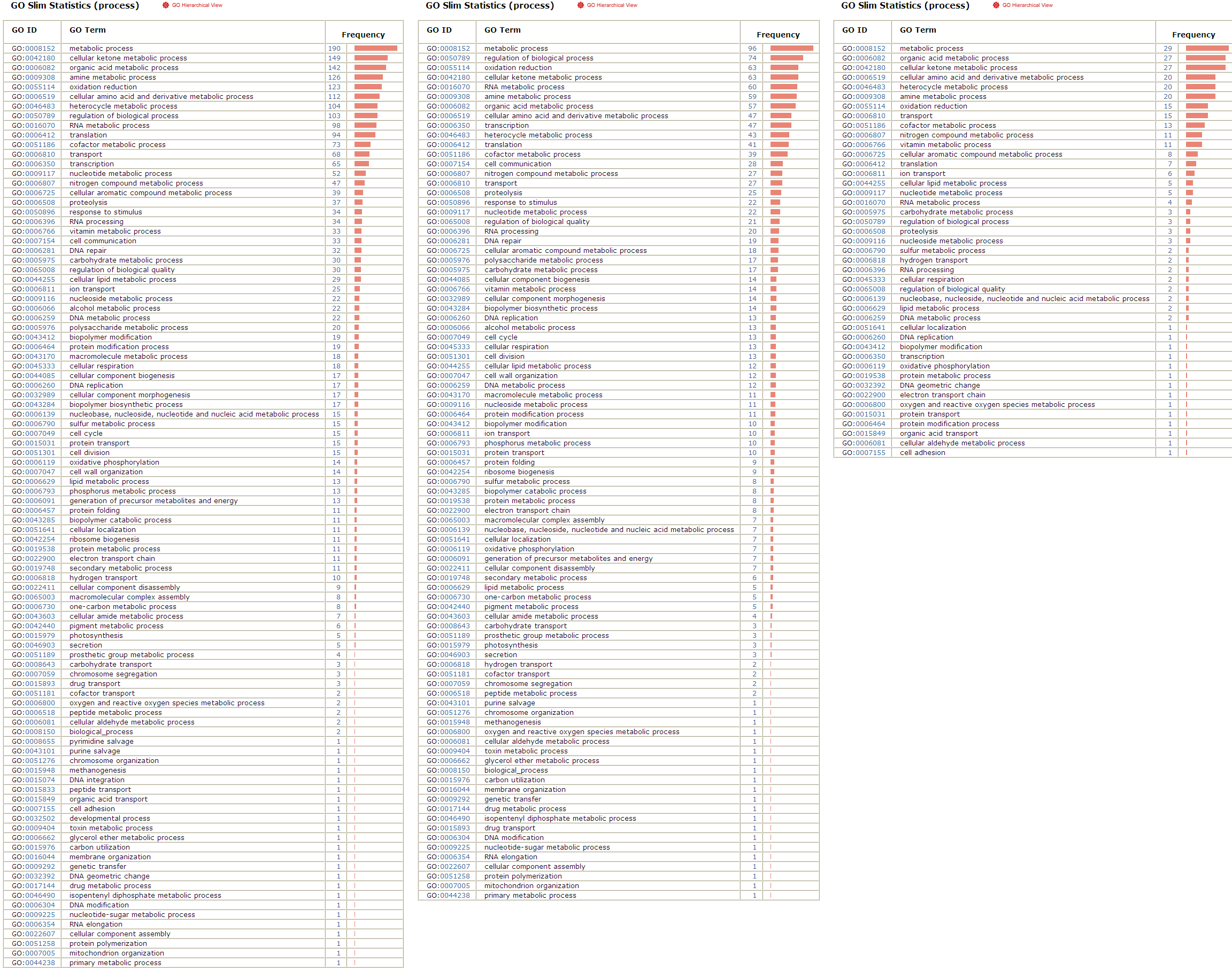

Supplement: Figure S2 — Functional classification of the genome representing the statistics for likely orthologous genes (left), core genes (middle) and HGT genes (right) based on their annotations to terms in the GO biological process vocabularies. (0.69 MB TIF) [file pone.0011604.s002.tif]

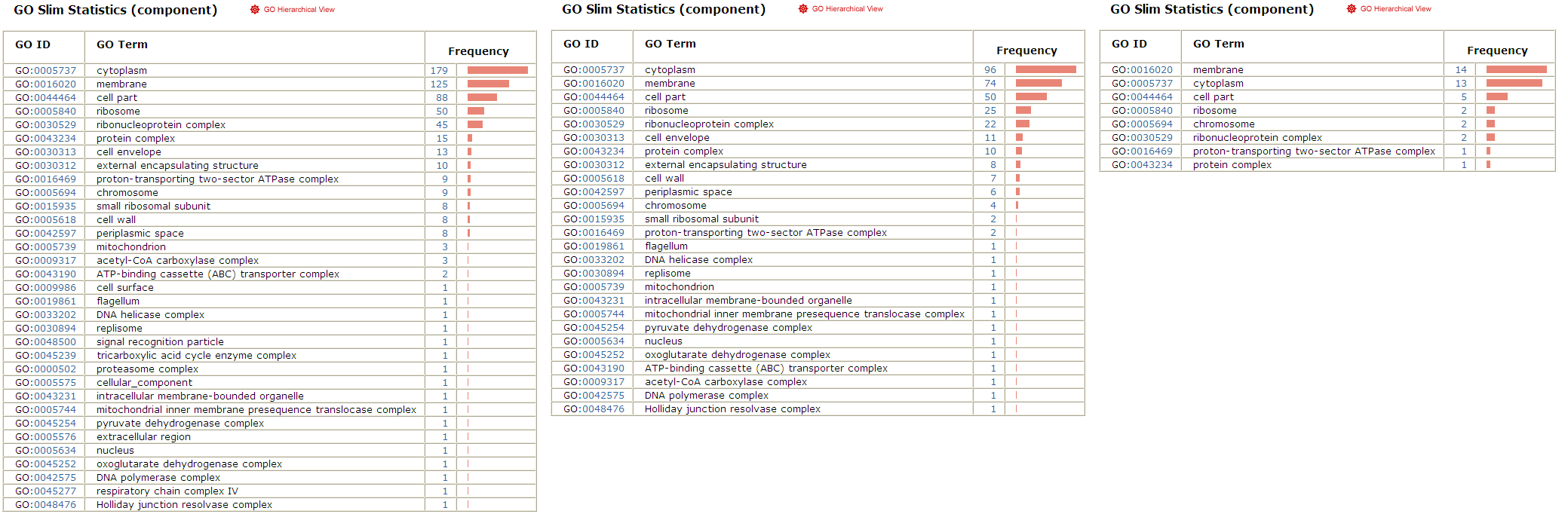

Supplement: Figure S3 — Functional classification of the genome representing the statistics for likely orthologous genes (left), core genes (middle) and HGT genes (right) based on their annotations to terms in the GO cellular component. (0.24 MB TIF) [file pone.0011604.s003.tif]

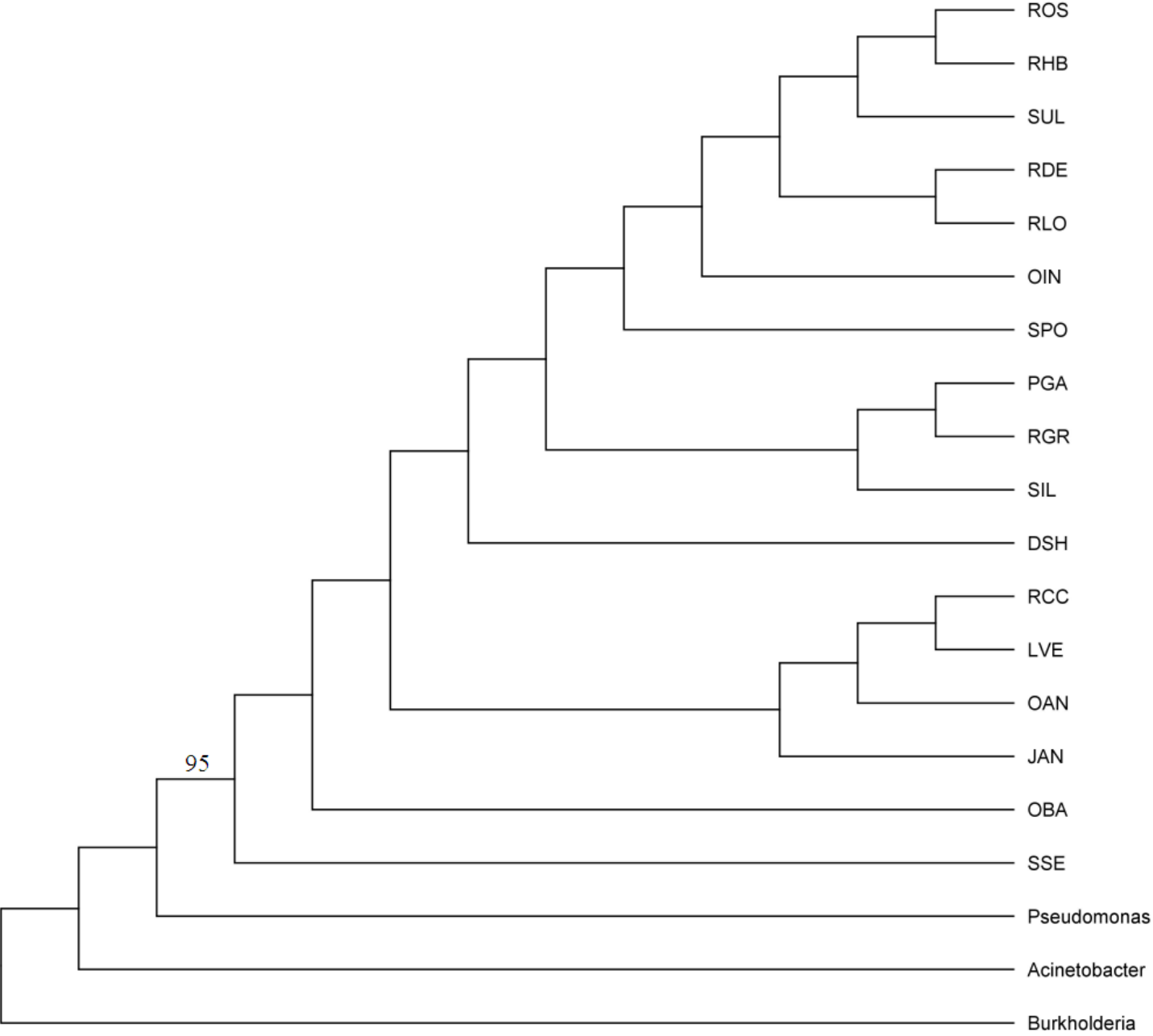

Supplement: File S2 — Tree topologies with the extended data. The multi-documents have been combined into a single ZIP-formatted file. The trees should be considered unrooted. The tree topologies were calculated in PhyML as described in Methods. Numbers refer to bootstrap values. The tree topology (separate pdf) shows that Roseobacter bacteria form a monophyletic group and was deposited in a document named “high bootstrap”. The other organisms embedded within the Roseobacter clade, or Roseobacter bacteria embedded within other phyla are shown in red (deposited in a document named “inter-phylum”). Individual file name corresponds to gene family code listed in Table S1. The non Roseobacter organism taxonomic name is detailed in the amino acid fasta of the sequences (a document named “sequences”). (4.62 MB ZIP) [file pone.0011604.s008.zip › high bootstrap/ort1000.pdf]

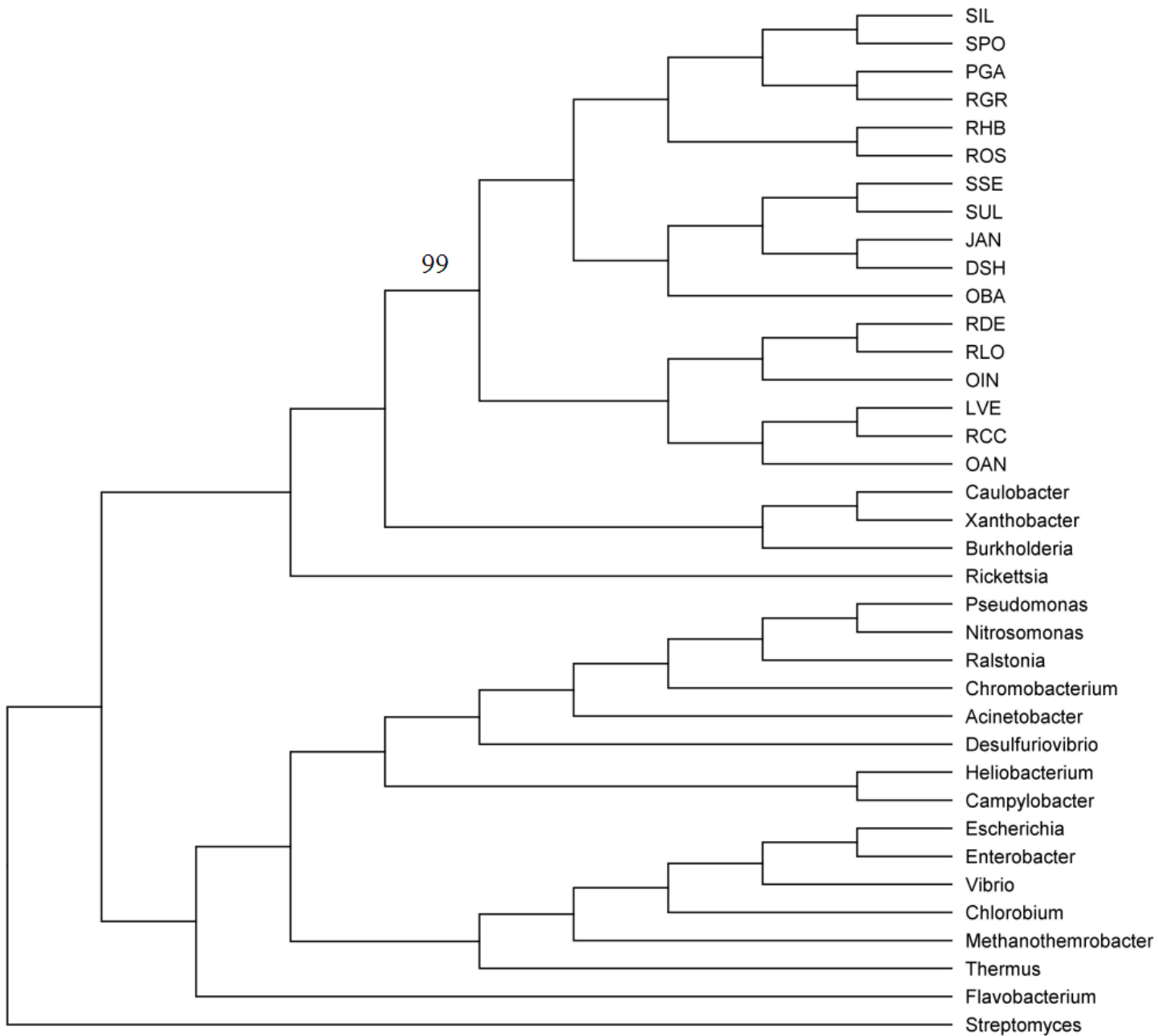

Supplement: File S2 — Tree topologies with the extended data. The multi-documents have been combined into a single ZIP-formatted file. The trees should be considered unrooted. The tree topologies were calculated in PhyML as described in Methods. Numbers refer to bootstrap values. The tree topology (separate pdf) shows that Roseobacter bacteria form a monophyletic group and was deposited in a document named “high bootstrap”. The other organisms embedded within the Roseobacter clade, or Roseobacter bacteria embedded within other phyla are shown in red (deposited in a document named “inter-phylum”). Individual file name corresponds to gene family code listed in Table S1. The non Roseobacter organism taxonomic name is detailed in the amino acid fasta of the sequences (a document named “sequences”). (4.62 MB ZIP) [file pone.0011604.s008.zip › high bootstrap/ort103.pdf]

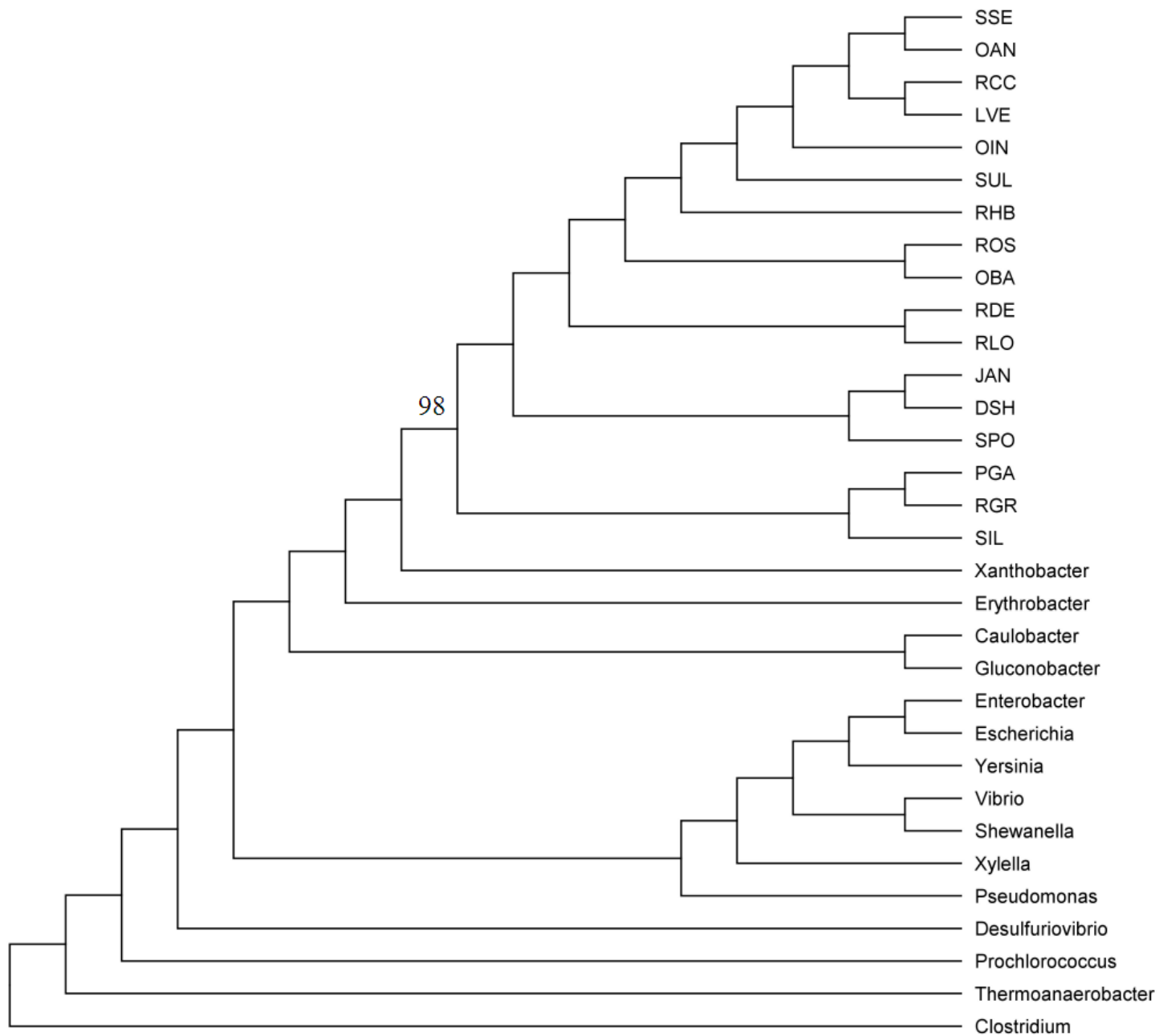

Supplement: File S2 — Tree topologies with the extended data. The multi-documents have been combined into a single ZIP-formatted file. The trees should be considered unrooted. The tree topologies were calculated in PhyML as described in Methods. Numbers refer to bootstrap values. The tree topology (separate pdf) shows that Roseobacter bacteria form a monophyletic group and was deposited in a document named “high bootstrap”. The other organisms embedded within the Roseobacter clade, or Roseobacter bacteria embedded within other phyla are shown in red (deposited in a document named “inter-phylum”). Individual file name corresponds to gene family code listed in Table S1. The non Roseobacter organism taxonomic name is detailed in the amino acid fasta of the sequences (a document named “sequences”). (4.62 MB ZIP) [file pone.0011604.s008.zip › high bootstrap/ort106.pdf]

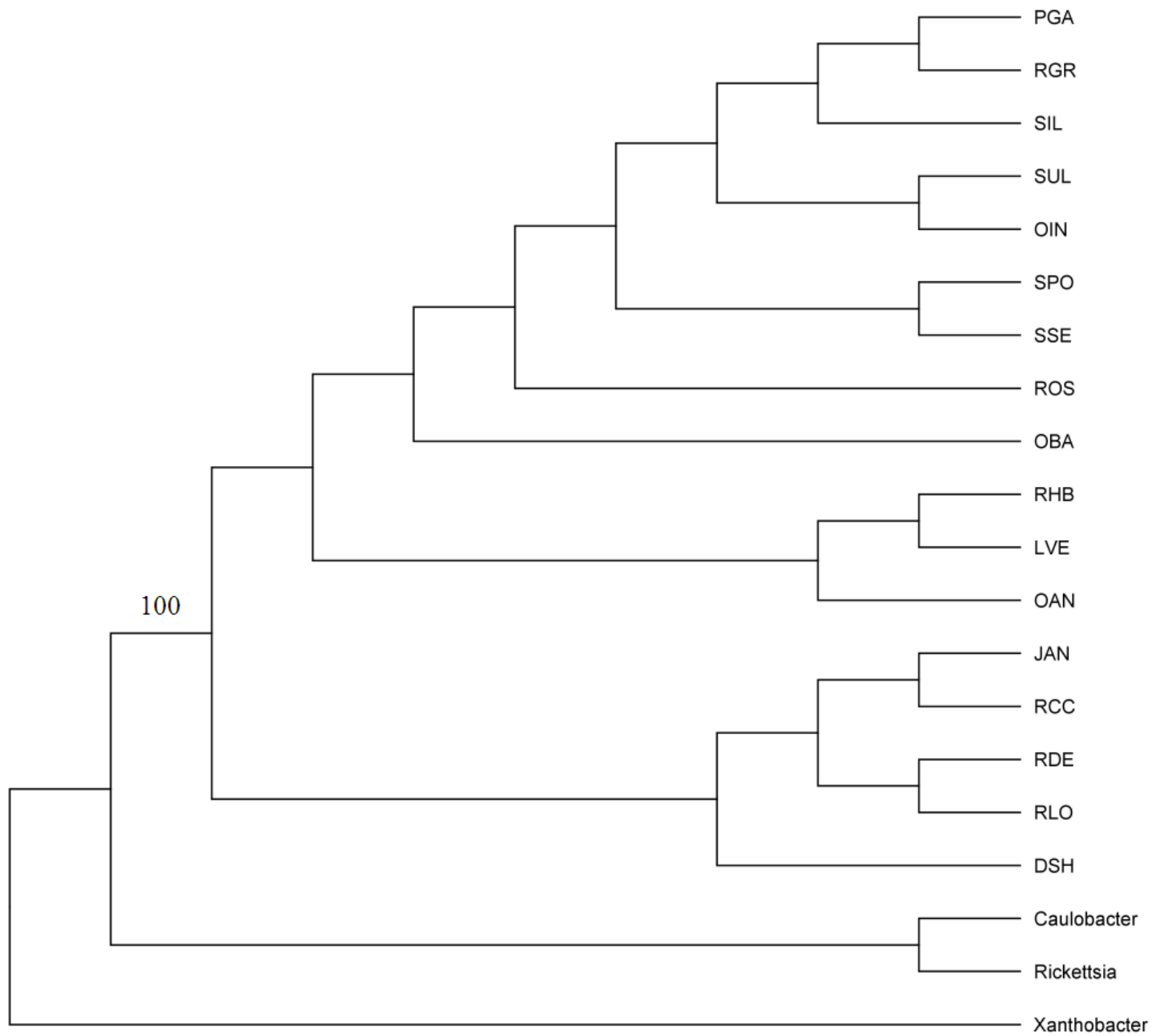

Supplement: File S2 — Tree topologies with the extended data. The multi-documents have been combined into a single ZIP-formatted file. The trees should be considered unrooted. The tree topologies were calculated in PhyML as described in Methods. Numbers refer to bootstrap values. The tree topology (separate pdf) shows that Roseobacter bacteria form a monophyletic group and was deposited in a document named “high bootstrap”. The other organisms embedded within the Roseobacter clade, or Roseobacter bacteria embedded within other phyla are shown in red (deposited in a document named “inter-phylum”). Individual file name corresponds to gene family code listed in Table S1. The non Roseobacter organism taxonomic name is detailed in the amino acid fasta of the sequences (a document named “sequences”). (4.62 MB ZIP) [file pone.0011604.s008.zip › high bootstrap/ort110.pdf]

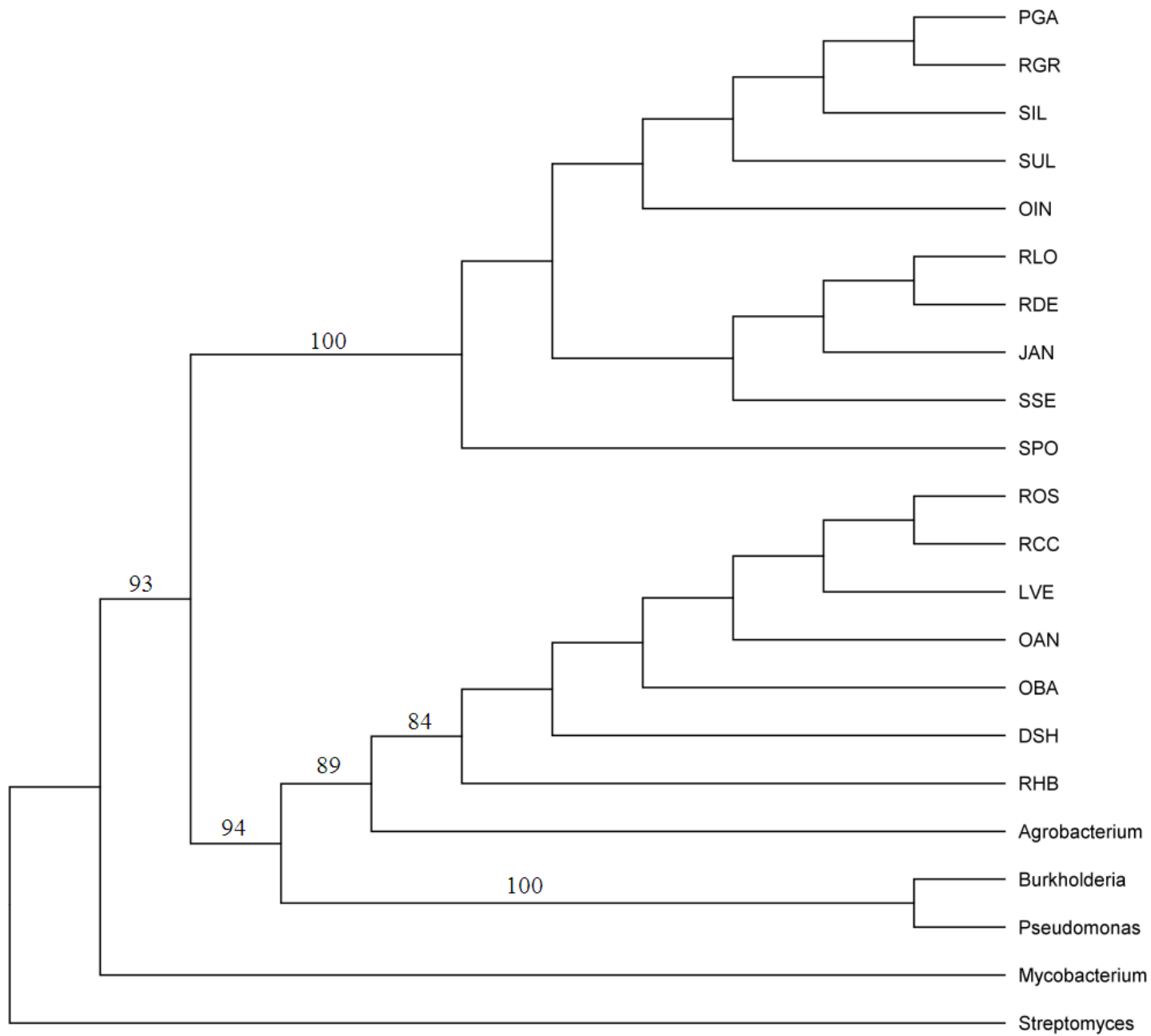

Supplement: File S2 — Tree topologies with the extended data. The multi-documents have been combined into a single ZIP-formatted file. The trees should be considered unrooted. The tree topologies were calculated in PhyML as described in Methods. Numbers refer to bootstrap values. The tree topology (separate pdf) shows that Roseobacter bacteria form a monophyletic group and was deposited in a document named “high bootstrap”. The other organisms embedded within the Roseobacter clade, or Roseobacter bacteria embedded within other phyla are shown in red (deposited in a document named “inter-phylum”). Individual file name corresponds to gene family code listed in Table S1. The non Roseobacter organism taxonomic name is detailed in the amino acid fasta of the sequences (a document named “sequences”). (4.62 MB ZIP) [file pone.0011604.s008.zip › high bootstrap/ort1116.pdf]

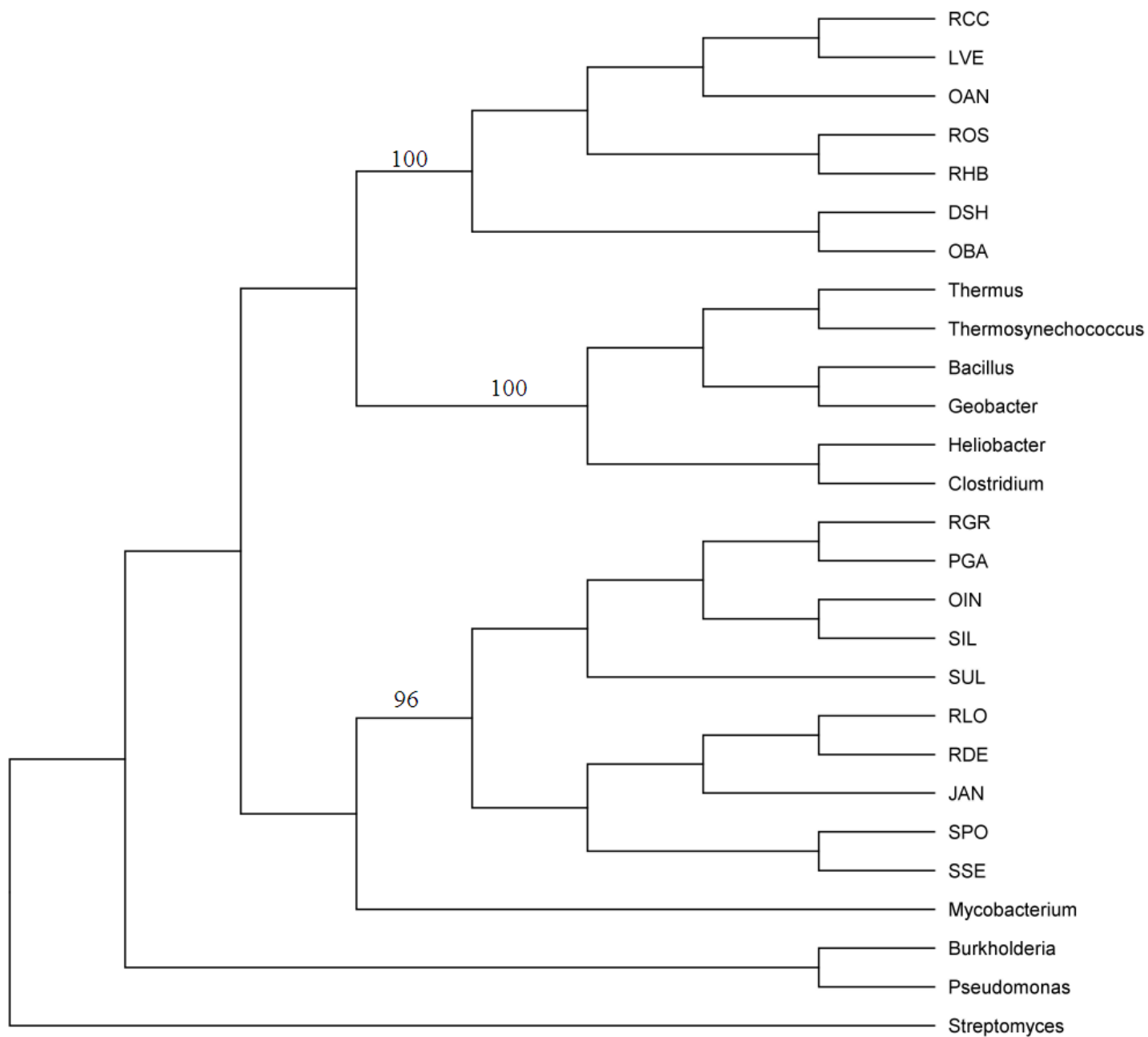

Supplement: File S2 — Tree topologies with the extended data. The multi-documents have been combined into a single ZIP-formatted file. The trees should be considered unrooted. The tree topologies were calculated in PhyML as described in Methods. Numbers refer to bootstrap values. The tree topology (separate pdf) shows that Roseobacter bacteria form a monophyletic group and was deposited in a document named “high bootstrap”. The other organisms embedded within the Roseobacter clade, or Roseobacter bacteria embedded within other phyla are shown in red (deposited in a document named “inter-phylum”). Individual file name corresponds to gene family code listed in Table S1. The non Roseobacter organism taxonomic name is detailed in the amino acid fasta of the sequences (a document named “sequences”). (4.62 MB ZIP) [file pone.0011604.s008.zip › high bootstrap/ort1117.pdf]

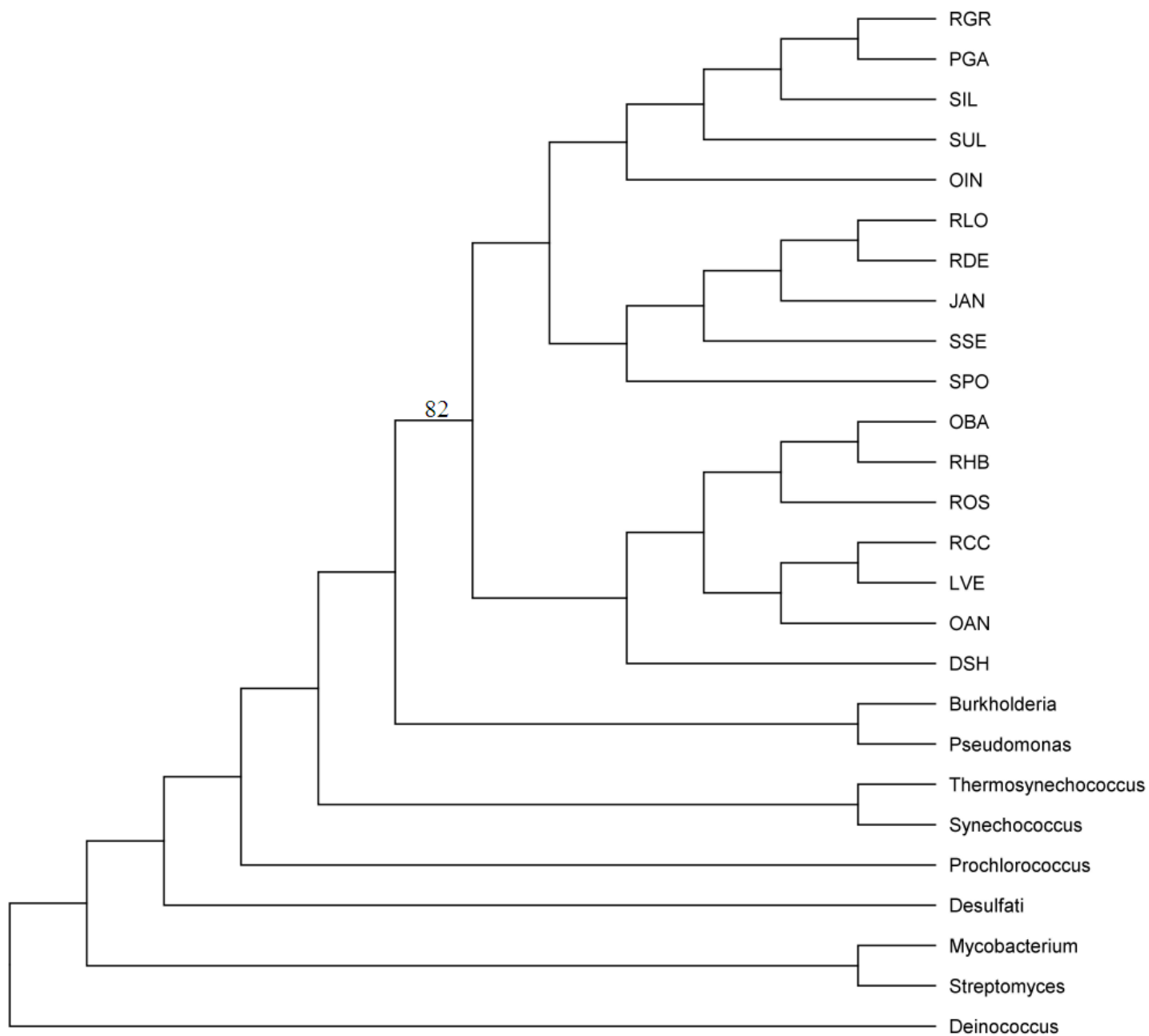

Supplement: File S2 — Tree topologies with the extended data. The multi-documents have been combined into a single ZIP-formatted file. The trees should be considered unrooted. The tree topologies were calculated in PhyML as described in Methods. Numbers refer to bootstrap values. The tree topology (separate pdf) shows that Roseobacter bacteria form a monophyletic group and was deposited in a document named “high bootstrap”. The other organisms embedded within the Roseobacter clade, or Roseobacter bacteria embedded within other phyla are shown in red (deposited in a document named “inter-phylum”). Individual file name corresponds to gene family code listed in Table S1. The non Roseobacter organism taxonomic name is detailed in the amino acid fasta of the sequences (a document named “sequences”). (4.62 MB ZIP) [file pone.0011604.s008.zip › high bootstrap/ort1124.pdf]

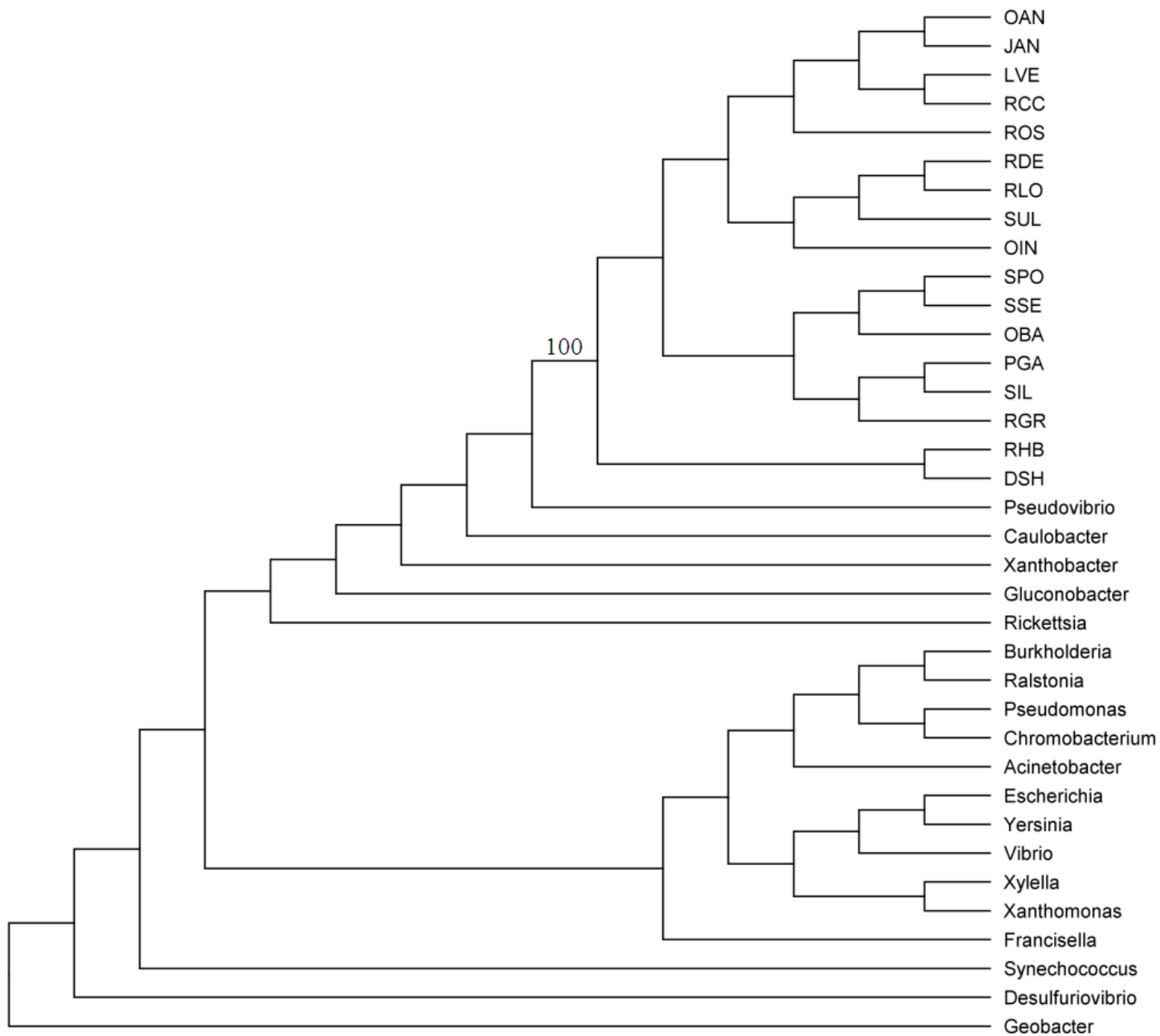

Supplement: File S2 — Tree topologies with the extended data. The multi-documents have been combined into a single ZIP-formatted file. The trees should be considered unrooted. The tree topologies were calculated in PhyML as described in Methods. Numbers refer to bootstrap values. The tree topology (separate pdf) shows that Roseobacter bacteria form a monophyletic group and was deposited in a document named “high bootstrap”. The other organisms embedded within the Roseobacter clade, or Roseobacter bacteria embedded within other phyla are shown in red (deposited in a document named “inter-phylum”). Individual file name corresponds to gene family code listed in Table S1. The non Roseobacter organism taxonomic name is detailed in the amino acid fasta of the sequences (a document named “sequences”). (4.62 MB ZIP) [file pone.0011604.s008.zip › high bootstrap/ort1172.pdf]

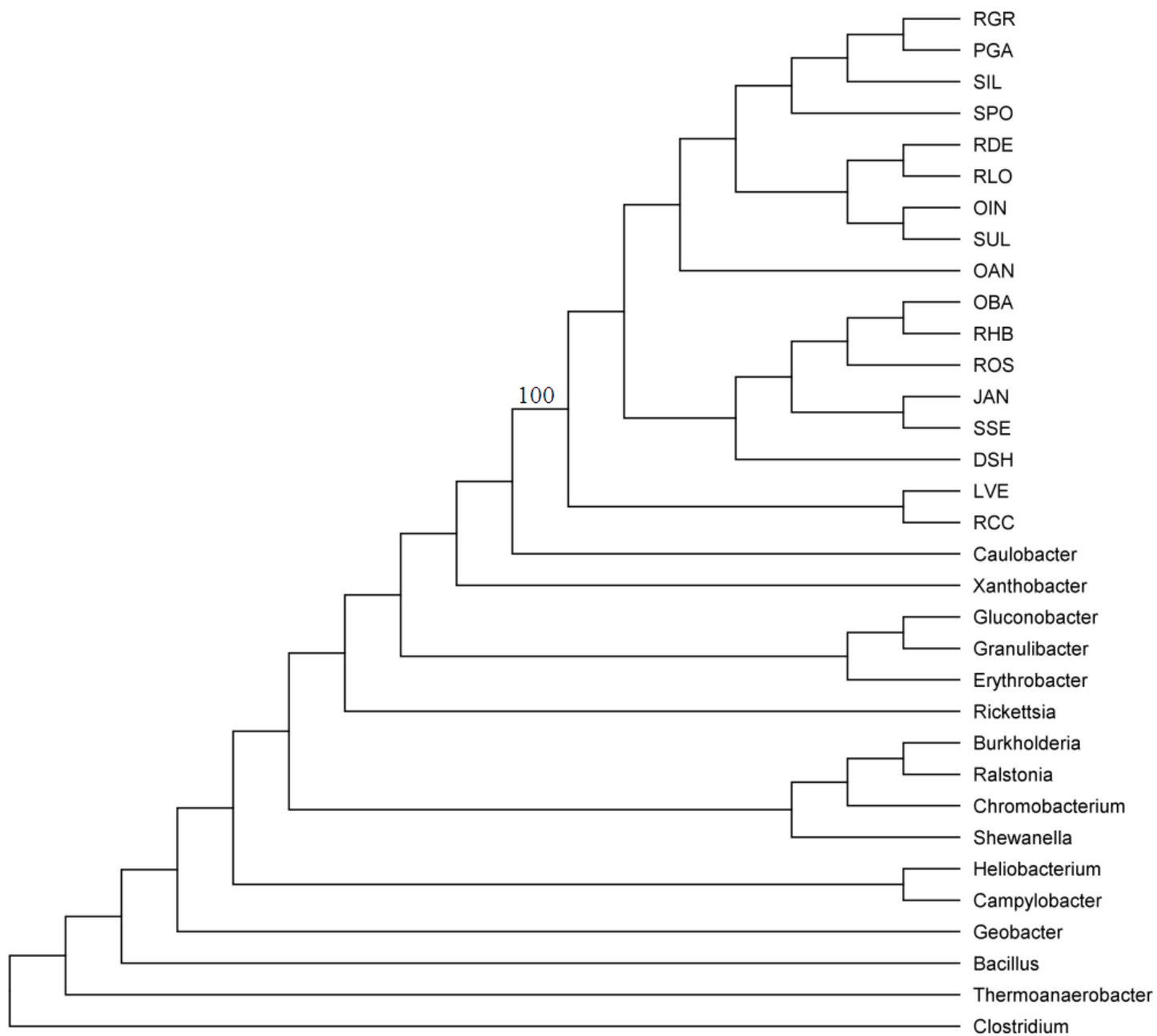

Supplement: File S2 — Tree topologies with the extended data. The multi-documents have been combined into a single ZIP-formatted file. The trees should be considered unrooted. The tree topologies were calculated in PhyML as described in Methods. Numbers refer to bootstrap values. The tree topology (separate pdf) shows that Roseobacter bacteria form a monophyletic group and was deposited in a document named “high bootstrap”. The other organisms embedded within the Roseobacter clade, or Roseobacter bacteria embedded within other phyla are shown in red (deposited in a document named “inter-phylum”). Individual file name corresponds to gene family code listed in Table S1. The non Roseobacter organism taxonomic name is detailed in the amino acid fasta of the sequences (a document named “sequences”). (4.62 MB ZIP) [file pone.0011604.s008.zip › high bootstrap/ort1190.pdf]

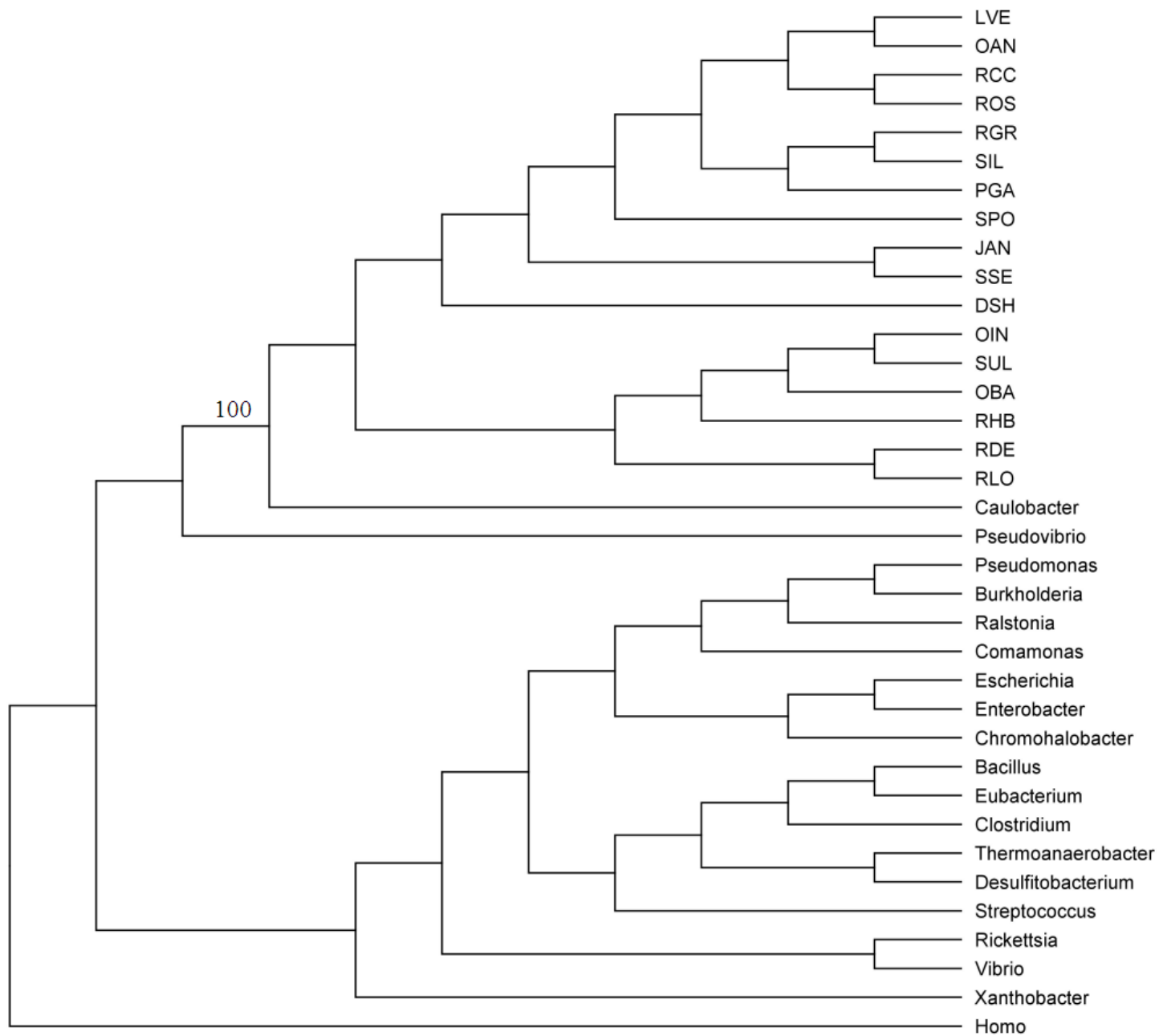

Supplement: File S2 — Tree topologies with the extended data. The multi-documents have been combined into a single ZIP-formatted file. The trees should be considered unrooted. The tree topologies were calculated in PhyML as described in Methods. Numbers refer to bootstrap values. The tree topology (separate pdf) shows that Roseobacter bacteria form a monophyletic group and was deposited in a document named “high bootstrap”. The other organisms embedded within the Roseobacter clade, or Roseobacter bacteria embedded within other phyla are shown in red (deposited in a document named “inter-phylum”). Individual file name corresponds to gene family code listed in Table S1. The non Roseobacter organism taxonomic name is detailed in the amino acid fasta of the sequences (a document named “sequences”). (4.62 MB ZIP) [file pone.0011604.s008.zip › high bootstrap/ort148.pdf]

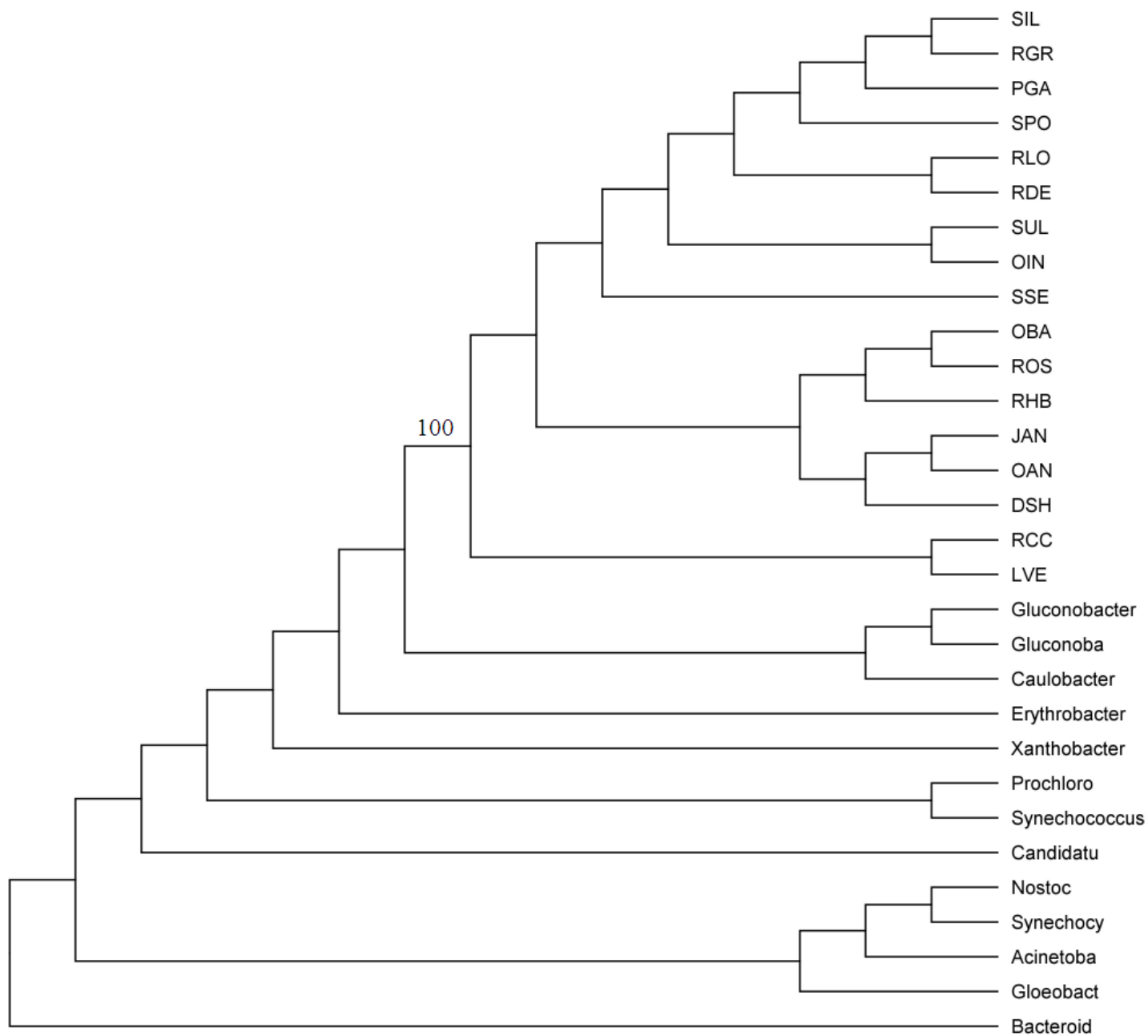

Supplement: File S2 — Tree topologies with the extended data. The multi-documents have been combined into a single ZIP-formatted file. The trees should be considered unrooted. The tree topologies were calculated in PhyML as described in Methods. Numbers refer to bootstrap values. The tree topology (separate pdf) shows that Roseobacter bacteria form a monophyletic group and was deposited in a document named “high bootstrap”. The other organisms embedded within the Roseobacter clade, or Roseobacter bacteria embedded within other phyla are shown in red (deposited in a document named “inter-phylum”). Individual file name corresponds to gene family code listed in Table S1. The non Roseobacter organism taxonomic name is detailed in the amino acid fasta of the sequences (a document named “sequences”). (4.62 MB ZIP) [file pone.0011604.s008.zip › high bootstrap/ort173.pdf]

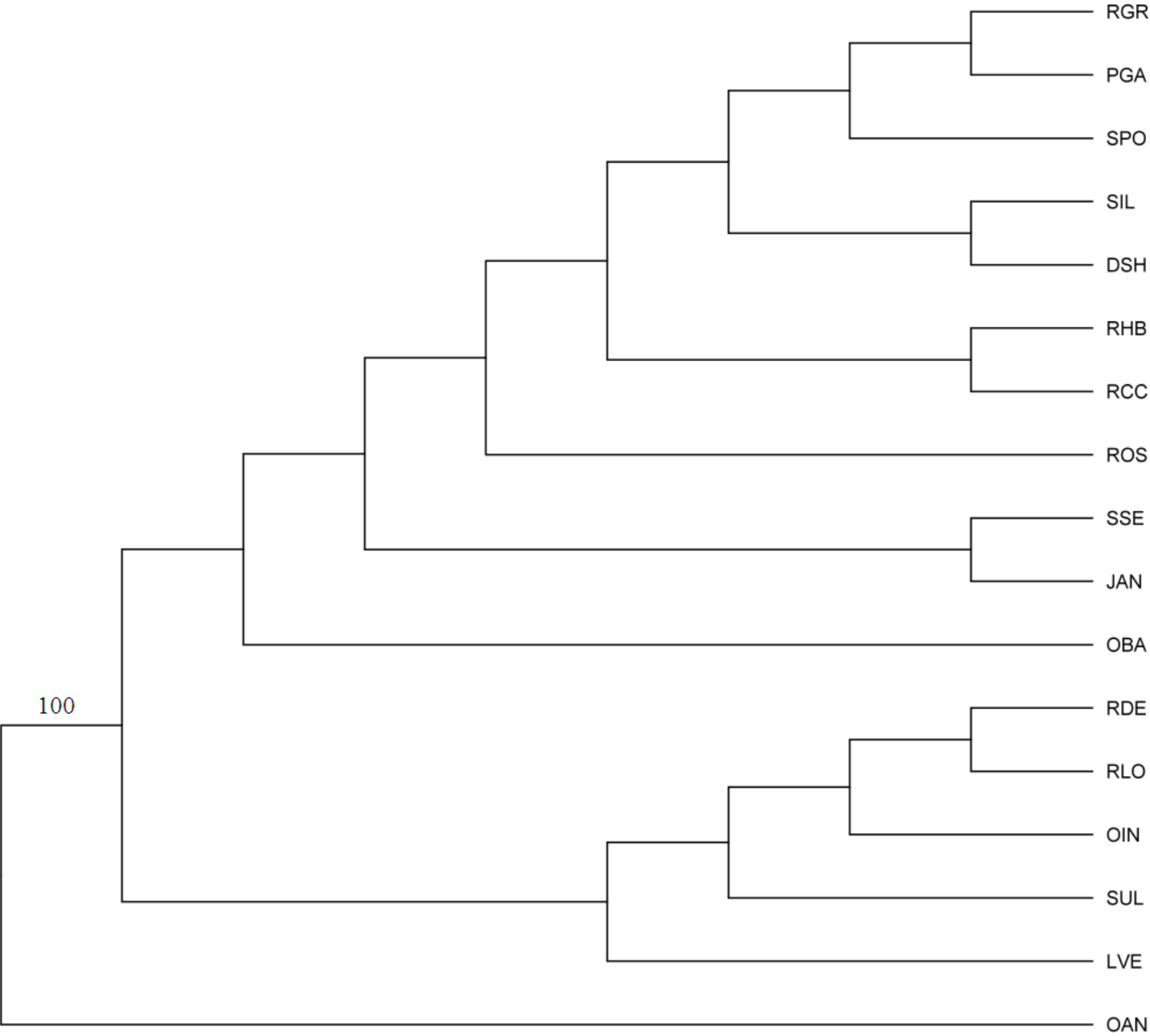

Supplement: File S2 — Tree topologies with the extended data. The multi-documents have been combined into a single ZIP-formatted file. The trees should be considered unrooted. The tree topologies were calculated in PhyML as described in Methods. Numbers refer to bootstrap values. The tree topology (separate pdf) shows that Roseobacter bacteria form a monophyletic group and was deposited in a document named “high bootstrap”. The other organisms embedded within the Roseobacter clade, or Roseobacter bacteria embedded within other phyla are shown in red (deposited in a document named “inter-phylum”). Individual file name corresponds to gene family code listed in Table S1. The non Roseobacter organism taxonomic name is detailed in the amino acid fasta of the sequences (a document named “sequences”). (4.62 MB ZIP) [file pone.0011604.s008.zip › high bootstrap/ort182.pdf]

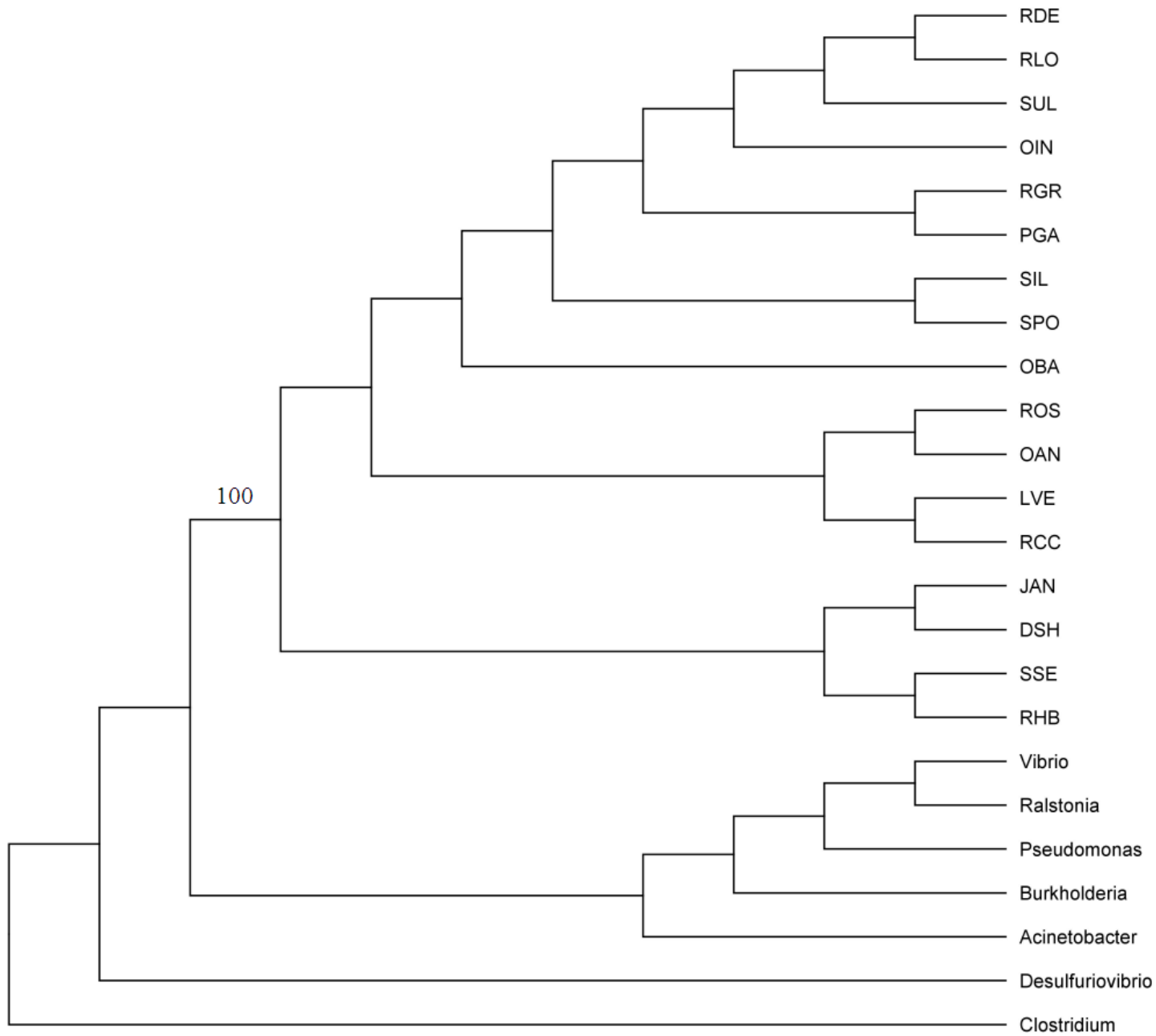

Supplement: File S2 — Tree topologies with the extended data. The multi-documents have been combined into a single ZIP-formatted file. The trees should be considered unrooted. The tree topologies were calculated in PhyML as described in Methods. Numbers refer to bootstrap values. The tree topology (separate pdf) shows that Roseobacter bacteria form a monophyletic group and was deposited in a document named “high bootstrap”. The other organisms embedded within the Roseobacter clade, or Roseobacter bacteria embedded within other phyla are shown in red (deposited in a document named “inter-phylum”). Individual file name corresponds to gene family code listed in Table S1. The non Roseobacter organism taxonomic name is detailed in the amino acid fasta of the sequences (a document named “sequences”). (4.62 MB ZIP) [file pone.0011604.s008.zip › high bootstrap/ort193.pdf]

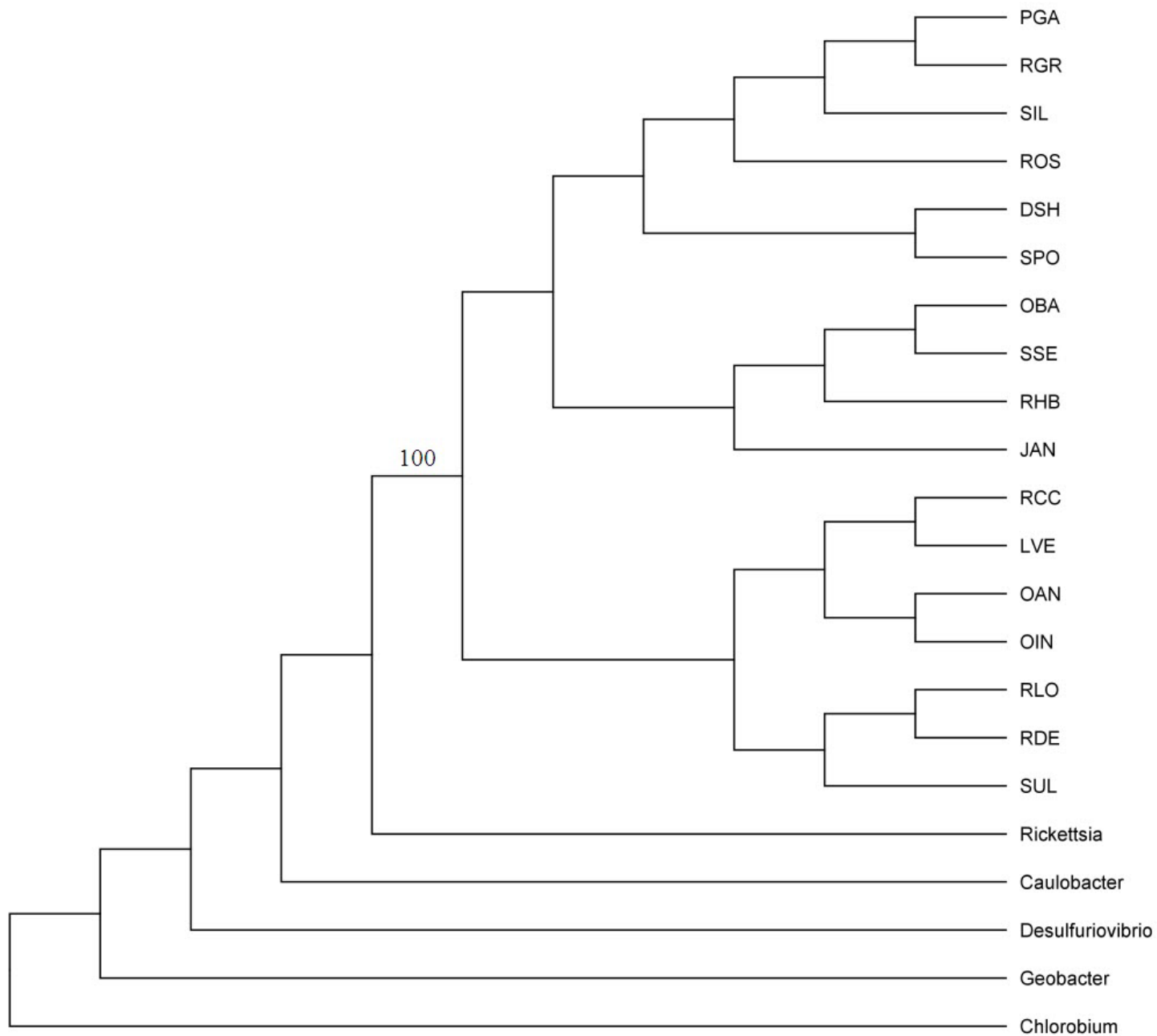

Supplement: File S2 — Tree topologies with the extended data. The multi-documents have been combined into a single ZIP-formatted file. The trees should be considered unrooted. The tree topologies were calculated in PhyML as described in Methods. Numbers refer to bootstrap values. The tree topology (separate pdf) shows that Roseobacter bacteria form a monophyletic group and was deposited in a document named “high bootstrap”. The other organisms embedded within the Roseobacter clade, or Roseobacter bacteria embedded within other phyla are shown in red (deposited in a document named “inter-phylum”). Individual file name corresponds to gene family code listed in Table S1. The non Roseobacter organism taxonomic name is detailed in the amino acid fasta of the sequences (a document named “sequences”). (4.62 MB ZIP) [file pone.0011604.s008.zip › high bootstrap/ort211.pdf]

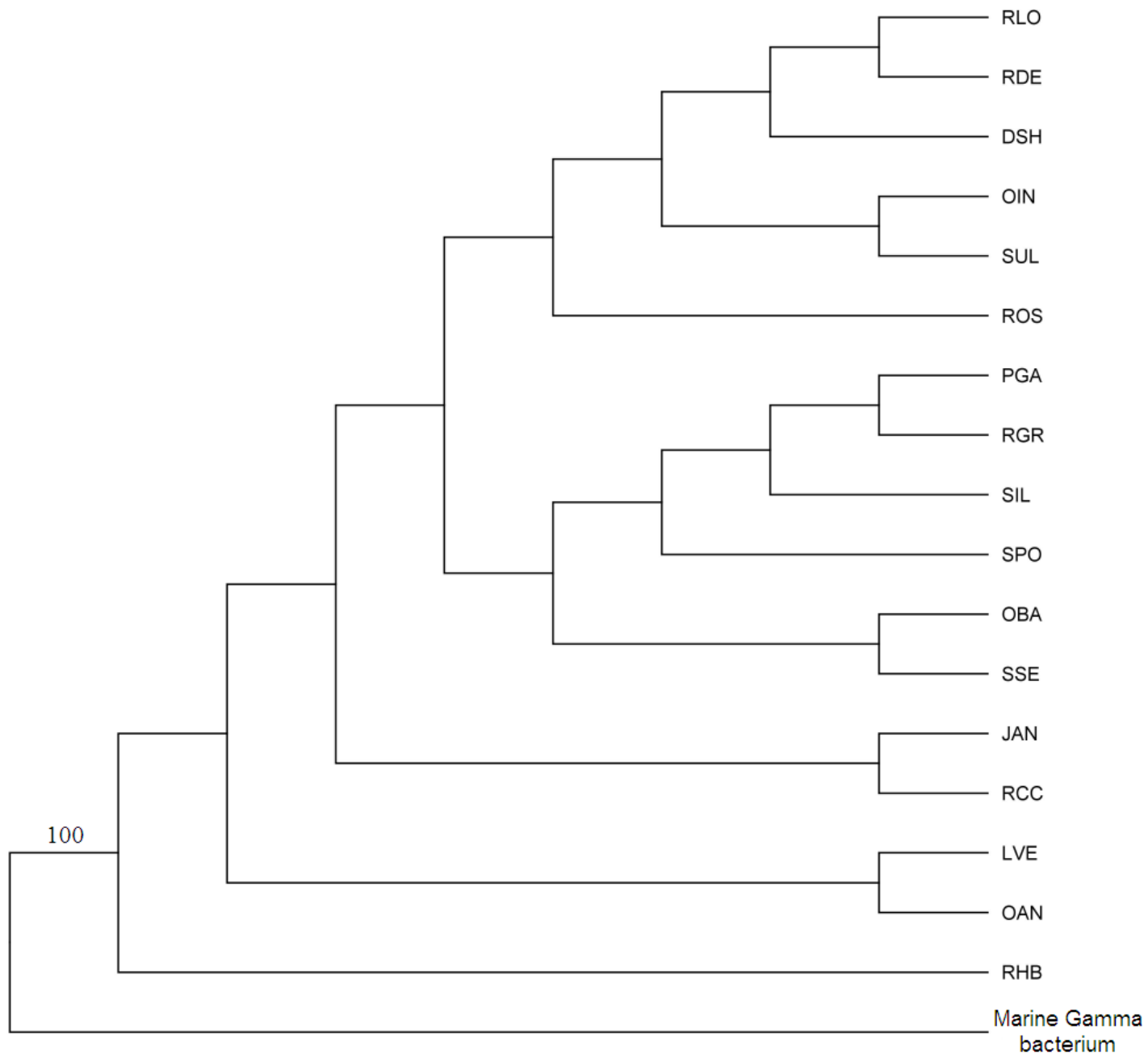

Supplement: File S2 — Tree topologies with the extended data. The multi-documents have been combined into a single ZIP-formatted file. The trees should be considered unrooted. The tree topologies were calculated in PhyML as described in Methods. Numbers refer to bootstrap values. The tree topology (separate pdf) shows that Roseobacter bacteria form a monophyletic group and was deposited in a document named “high bootstrap”. The other organisms embedded within the Roseobacter clade, or Roseobacter bacteria embedded within other phyla are shown in red (deposited in a document named “inter-phylum”). Individual file name corresponds to gene family code listed in Table S1. The non Roseobacter organism taxonomic name is detailed in the amino acid fasta of the sequences (a document named “sequences”). (4.62 MB ZIP) [file pone.0011604.s008.zip › high bootstrap/ort234.pdf]

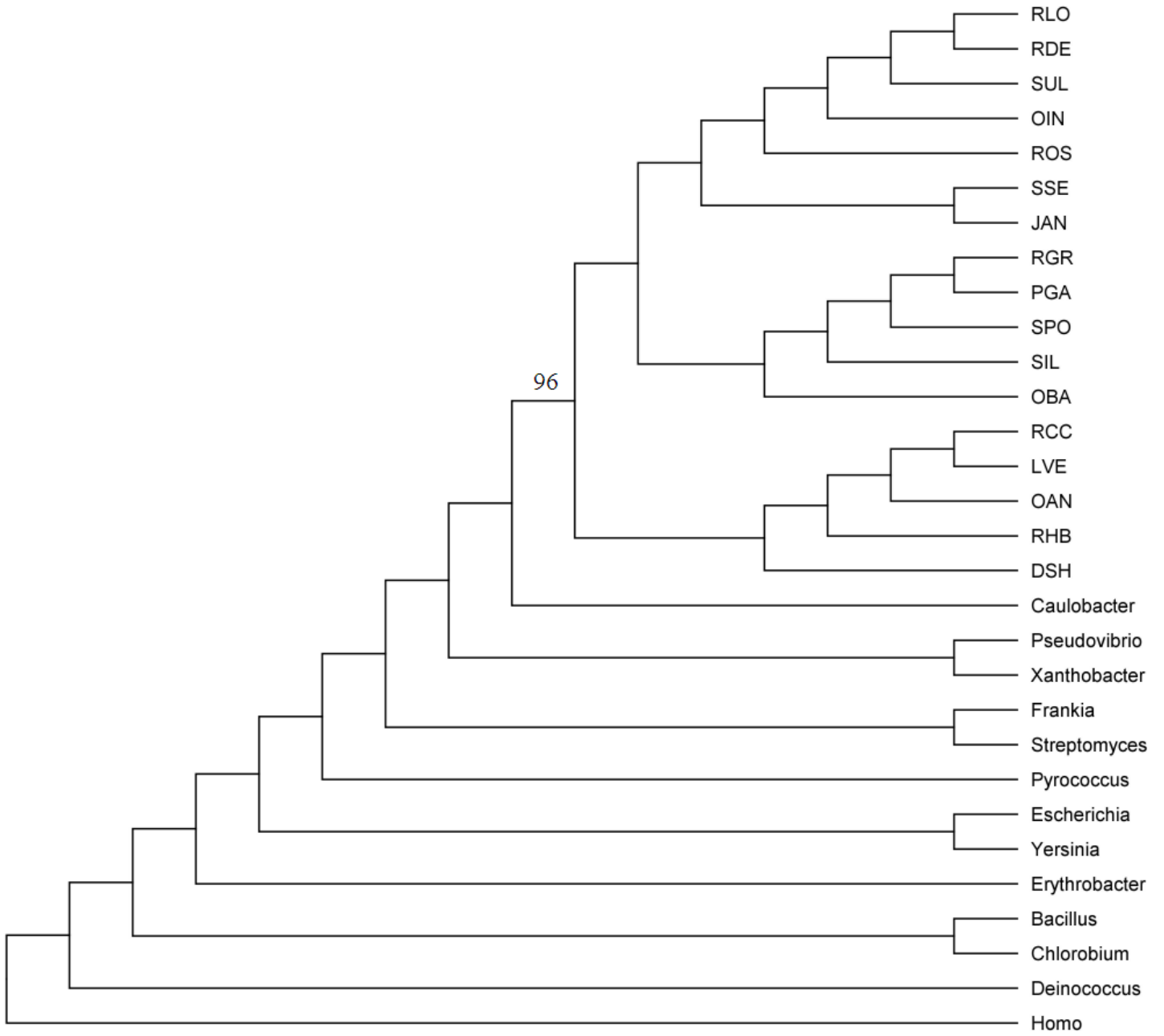

Supplement: File S2 — Tree topologies with the extended data. The multi-documents have been combined into a single ZIP-formatted file. The trees should be considered unrooted. The tree topologies were calculated in PhyML as described in Methods. Numbers refer to bootstrap values. The tree topology (separate pdf) shows that Roseobacter bacteria form a monophyletic group and was deposited in a document named “high bootstrap”. The other organisms embedded within the Roseobacter clade, or Roseobacter bacteria embedded within other phyla are shown in red (deposited in a document named “inter-phylum”). Individual file name corresponds to gene family code listed in Table S1. The non Roseobacter organism taxonomic name is detailed in the amino acid fasta of the sequences (a document named “sequences”). (4.62 MB ZIP) [file pone.0011604.s008.zip › high bootstrap/ort240.pdf]

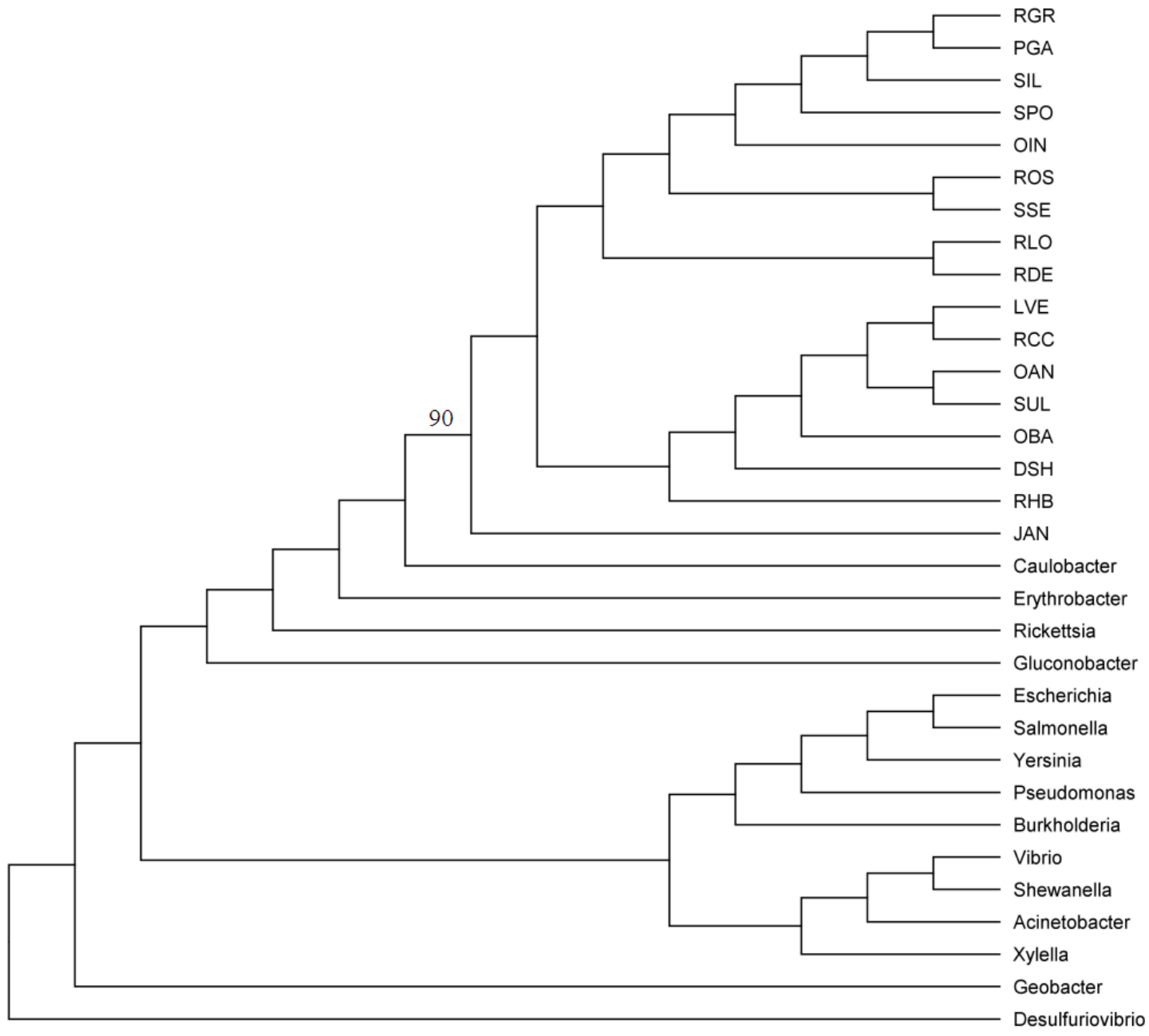

Supplement: File S2 — Tree topologies with the extended data. The multi-documents have been combined into a single ZIP-formatted file. The trees should be considered unrooted. The tree topologies were calculated in PhyML as described in Methods. Numbers refer to bootstrap values. The tree topology (separate pdf) shows that Roseobacter bacteria form a monophyletic group and was deposited in a document named “high bootstrap”. The other organisms embedded within the Roseobacter clade, or Roseobacter bacteria embedded within other phyla are shown in red (deposited in a document named “inter-phylum”). Individual file name corresponds to gene family code listed in Table S1. The non Roseobacter organism taxonomic name is detailed in the amino acid fasta of the sequences (a document named “sequences”). (4.62 MB ZIP) [file pone.0011604.s008.zip › high bootstrap/ort251.pdf]

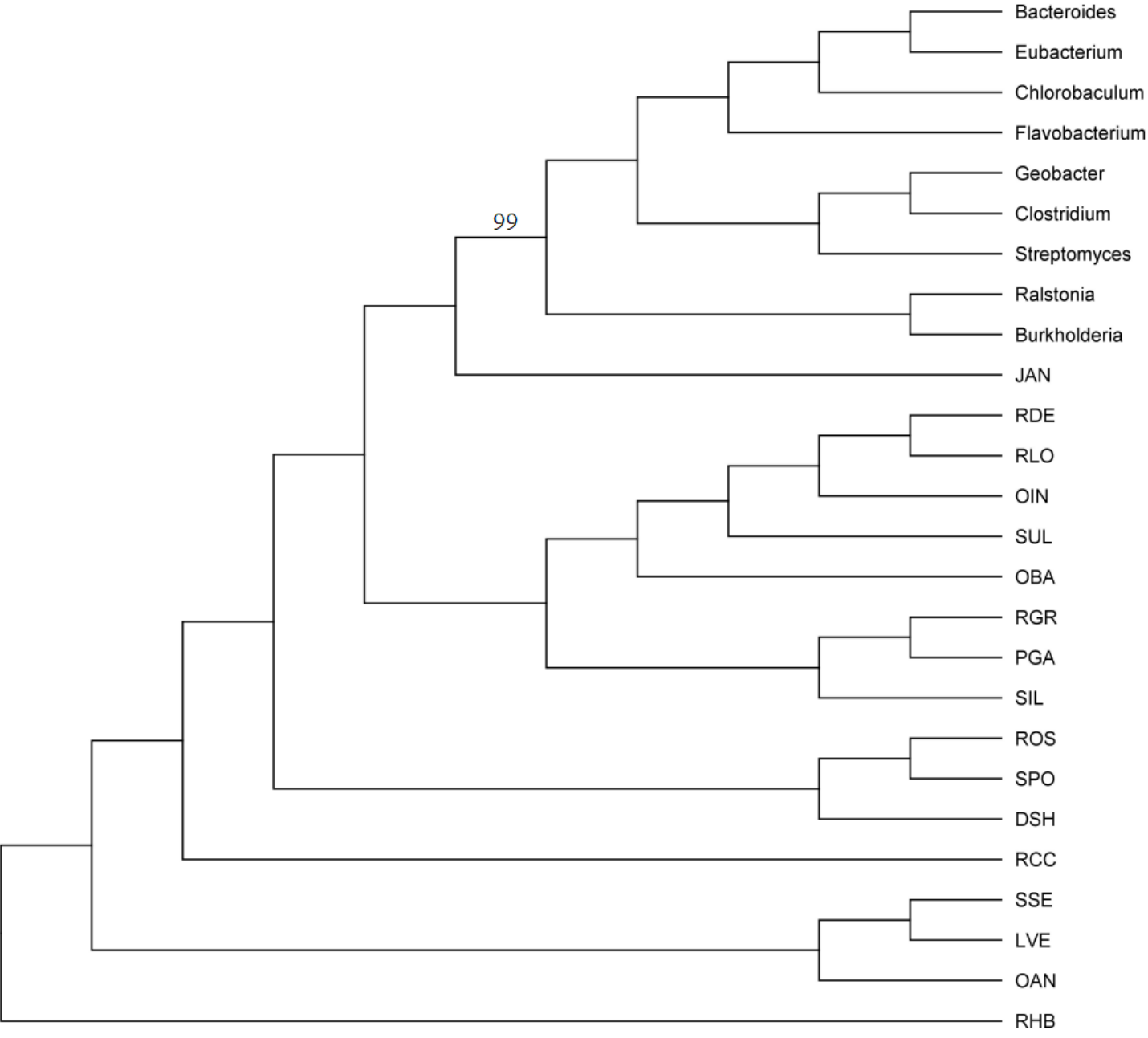

Supplement: File S2 — Tree topologies with the extended data. The multi-documents have been combined into a single ZIP-formatted file. The trees should be considered unrooted. The tree topologies were calculated in PhyML as described in Methods. Numbers refer to bootstrap values. The tree topology (separate pdf) shows that Roseobacter bacteria form a monophyletic group and was deposited in a document named “high bootstrap”. The other organisms embedded within the Roseobacter clade, or Roseobacter bacteria embedded within other phyla are shown in red (deposited in a document named “inter-phylum”). Individual file name corresponds to gene family code listed in Table S1. The non Roseobacter organism taxonomic name is detailed in the amino acid fasta of the sequences (a document named “sequences”). (4.62 MB ZIP) [file pone.0011604.s008.zip › high bootstrap/ort266.pdf]

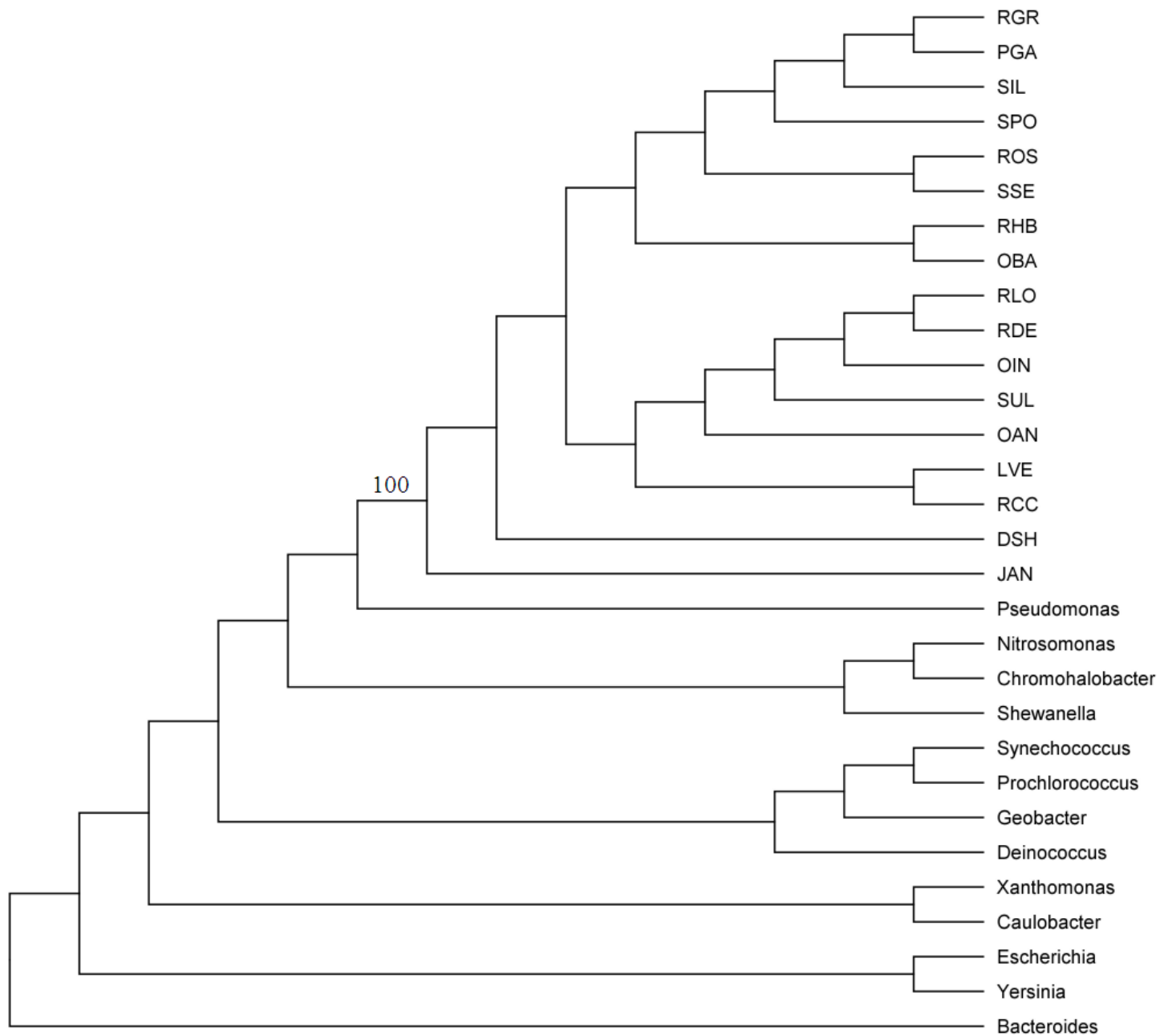

Supplement: File S2 — Tree topologies with the extended data. The multi-documents have been combined into a single ZIP-formatted file. The trees should be considered unrooted. The tree topologies were calculated in PhyML as described in Methods. Numbers refer to bootstrap values. The tree topology (separate pdf) shows that Roseobacter bacteria form a monophyletic group and was deposited in a document named “high bootstrap”. The other organisms embedded within the Roseobacter clade, or Roseobacter bacteria embedded within other phyla are shown in red (deposited in a document named “inter-phylum”). Individual file name corresponds to gene family code listed in Table S1. The non Roseobacter organism taxonomic name is detailed in the amino acid fasta of the sequences (a document named “sequences”). (4.62 MB ZIP) [file pone.0011604.s008.zip › high bootstrap/ort285.pdf]

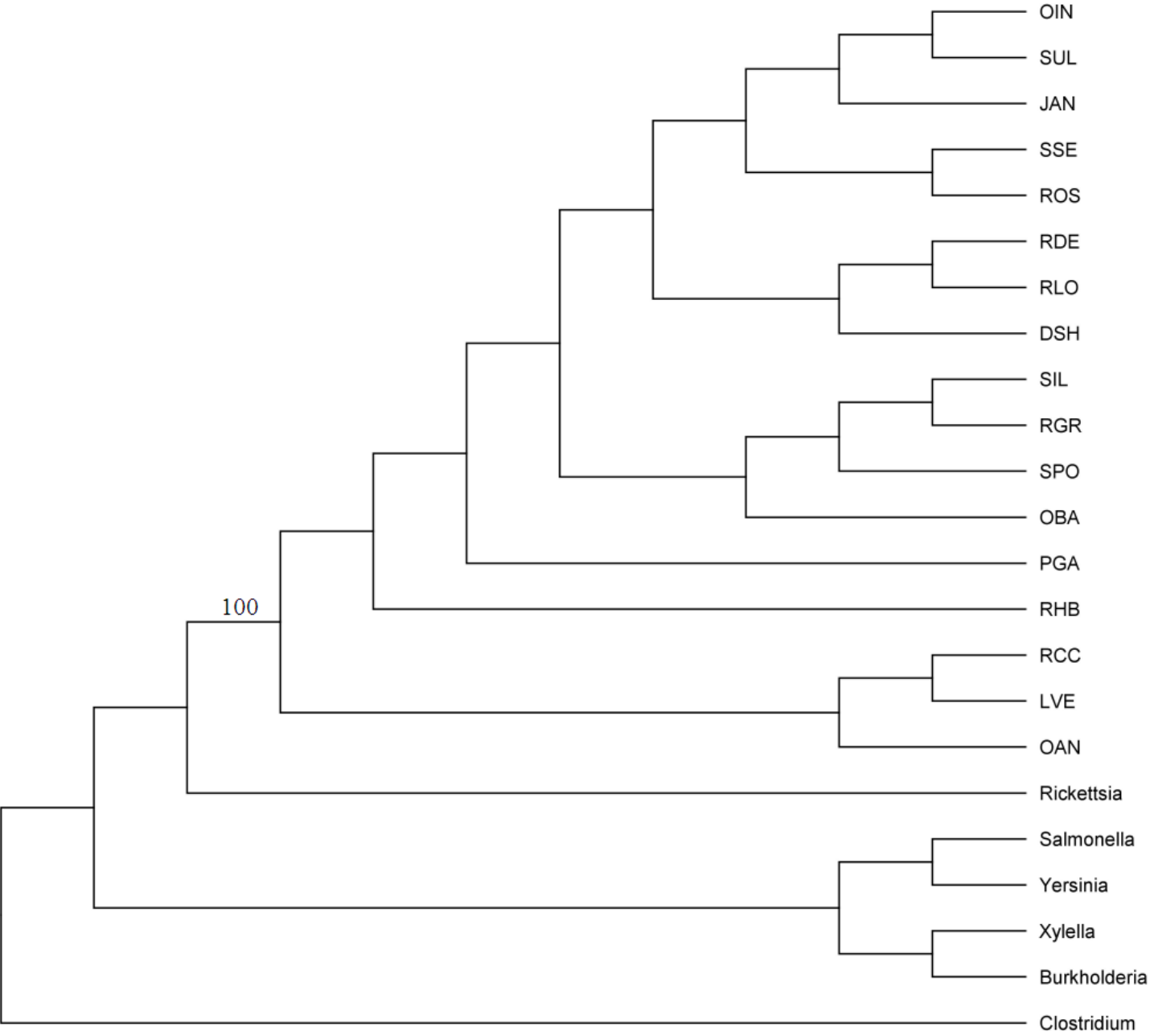

Supplement: File S2 — Tree topologies with the extended data. The multi-documents have been combined into a single ZIP-formatted file. The trees should be considered unrooted. The tree topologies were calculated in PhyML as described in Methods. Numbers refer to bootstrap values. The tree topology (separate pdf) shows that Roseobacter bacteria form a monophyletic group and was deposited in a document named “high bootstrap”. The other organisms embedded within the Roseobacter clade, or Roseobacter bacteria embedded within other phyla are shown in red (deposited in a document named “inter-phylum”). Individual file name corresponds to gene family code listed in Table S1. The non Roseobacter organism taxonomic name is detailed in the amino acid fasta of the sequences (a document named “sequences”). (4.62 MB ZIP) [file pone.0011604.s008.zip › high bootstrap/ort292.pdf]

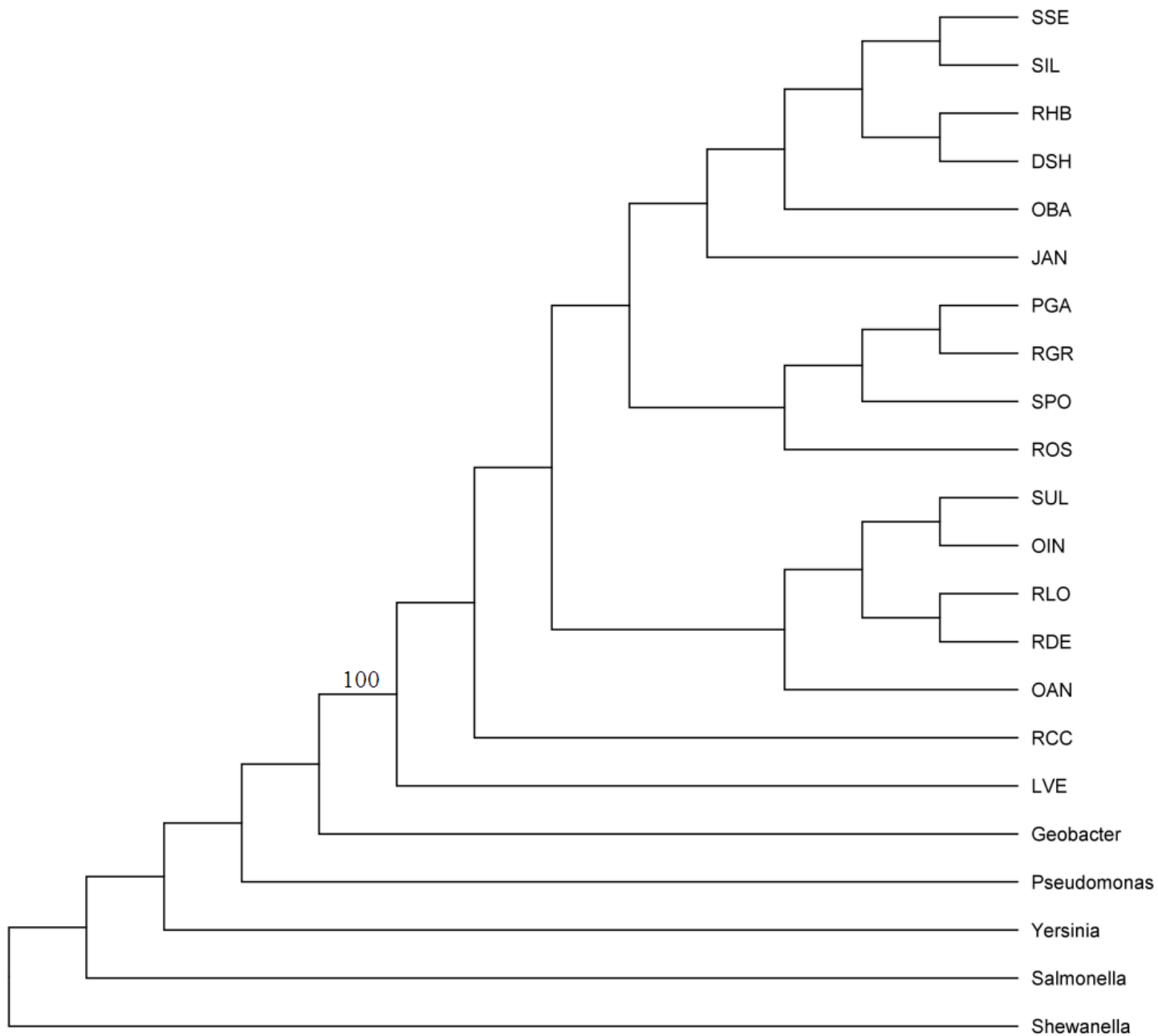

Supplement: File S2 — Tree topologies with the extended data. The multi-documents have been combined into a single ZIP-formatted file. The trees should be considered unrooted. The tree topologies were calculated in PhyML as described in Methods. Numbers refer to bootstrap values. The tree topology (separate pdf) shows that Roseobacter bacteria form a monophyletic group and was deposited in a document named “high bootstrap”. The other organisms embedded within the Roseobacter clade, or Roseobacter bacteria embedded within other phyla are shown in red (deposited in a document named “inter-phylum”). Individual file name corresponds to gene family code listed in Table S1. The non Roseobacter organism taxonomic name is detailed in the amino acid fasta of the sequences (a document named “sequences”). (4.62 MB ZIP) [file pone.0011604.s008.zip › high bootstrap/ort297.pdf]

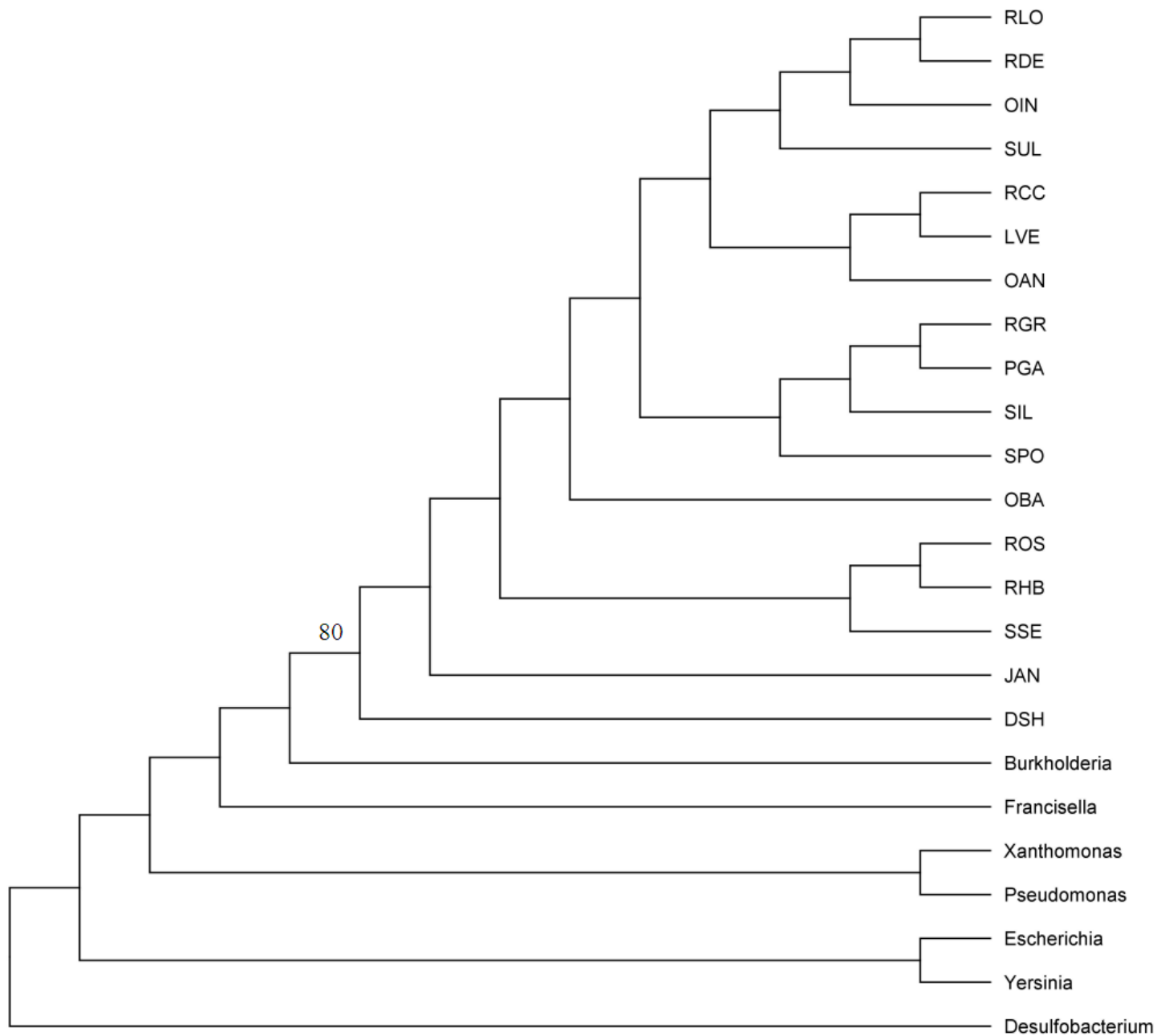

Supplement: File S2 — Tree topologies with the extended data. The multi-documents have been combined into a single ZIP-formatted file. The trees should be considered unrooted. The tree topologies were calculated in PhyML as described in Methods. Numbers refer to bootstrap values. The tree topology (separate pdf) shows that Roseobacter bacteria form a monophyletic group and was deposited in a document named “high bootstrap”. The other organisms embedded within the Roseobacter clade, or Roseobacter bacteria embedded within other phyla are shown in red (deposited in a document named “inter-phylum”). Individual file name corresponds to gene family code listed in Table S1. The non Roseobacter organism taxonomic name is detailed in the amino acid fasta of the sequences (a document named “sequences”). (4.62 MB ZIP) [file pone.0011604.s008.zip › high bootstrap/ort304.pdf]

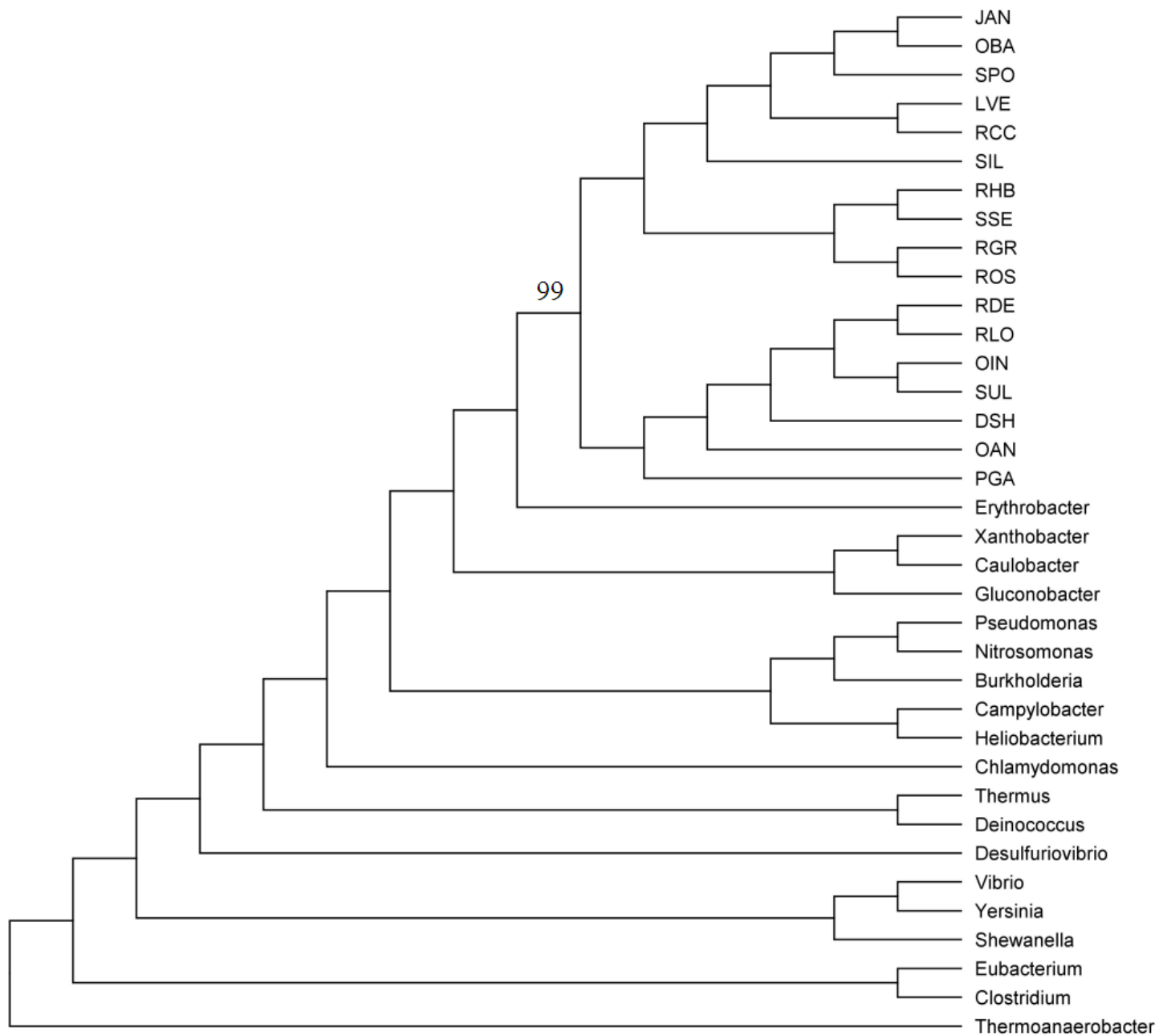

Supplement: File S2 — Tree topologies with the extended data. The multi-documents have been combined into a single ZIP-formatted file. The trees should be considered unrooted. The tree topologies were calculated in PhyML as described in Methods. Numbers refer to bootstrap values. The tree topology (separate pdf) shows that Roseobacter bacteria form a monophyletic group and was deposited in a document named “high bootstrap”. The other organisms embedded within the Roseobacter clade, or Roseobacter bacteria embedded within other phyla are shown in red (deposited in a document named “inter-phylum”). Individual file name corresponds to gene family code listed in Table S1. The non Roseobacter organism taxonomic name is detailed in the amino acid fasta of the sequences (a document named “sequences”). (4.62 MB ZIP) [file pone.0011604.s008.zip › high bootstrap/ort33.pdf]

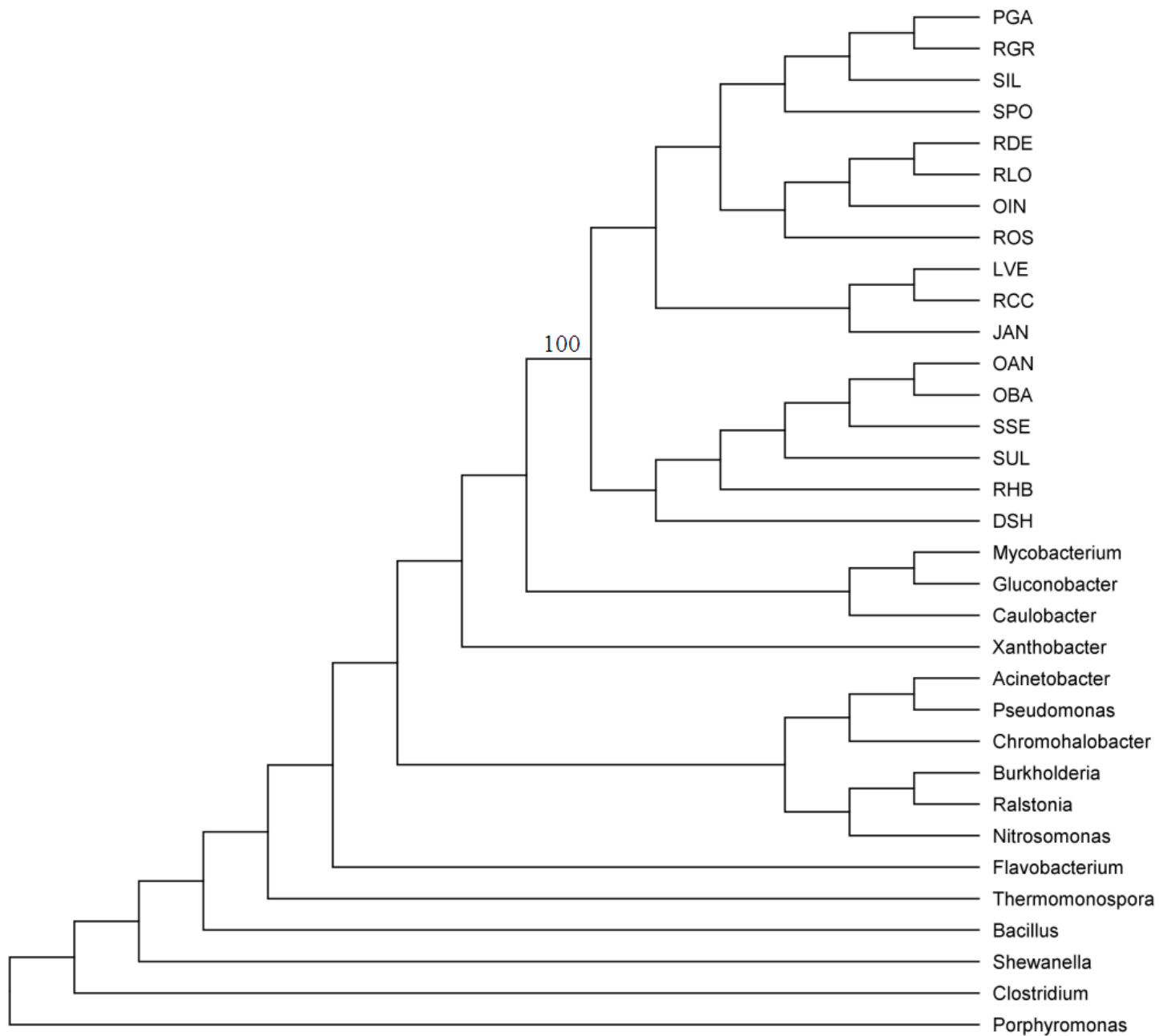

Supplement: File S2 — Tree topologies with the extended data. The multi-documents have been combined into a single ZIP-formatted file. The trees should be considered unrooted. The tree topologies were calculated in PhyML as described in Methods. Numbers refer to bootstrap values. The tree topology (separate pdf) shows that Roseobacter bacteria form a monophyletic group and was deposited in a document named “high bootstrap”. The other organisms embedded within the Roseobacter clade, or Roseobacter bacteria embedded within other phyla are shown in red (deposited in a document named “inter-phylum”). Individual file name corresponds to gene family code listed in Table S1. The non Roseobacter organism taxonomic name is detailed in the amino acid fasta of the sequences (a document named “sequences”). (4.62 MB ZIP) [file pone.0011604.s008.zip › high bootstrap/ort369.pdf]

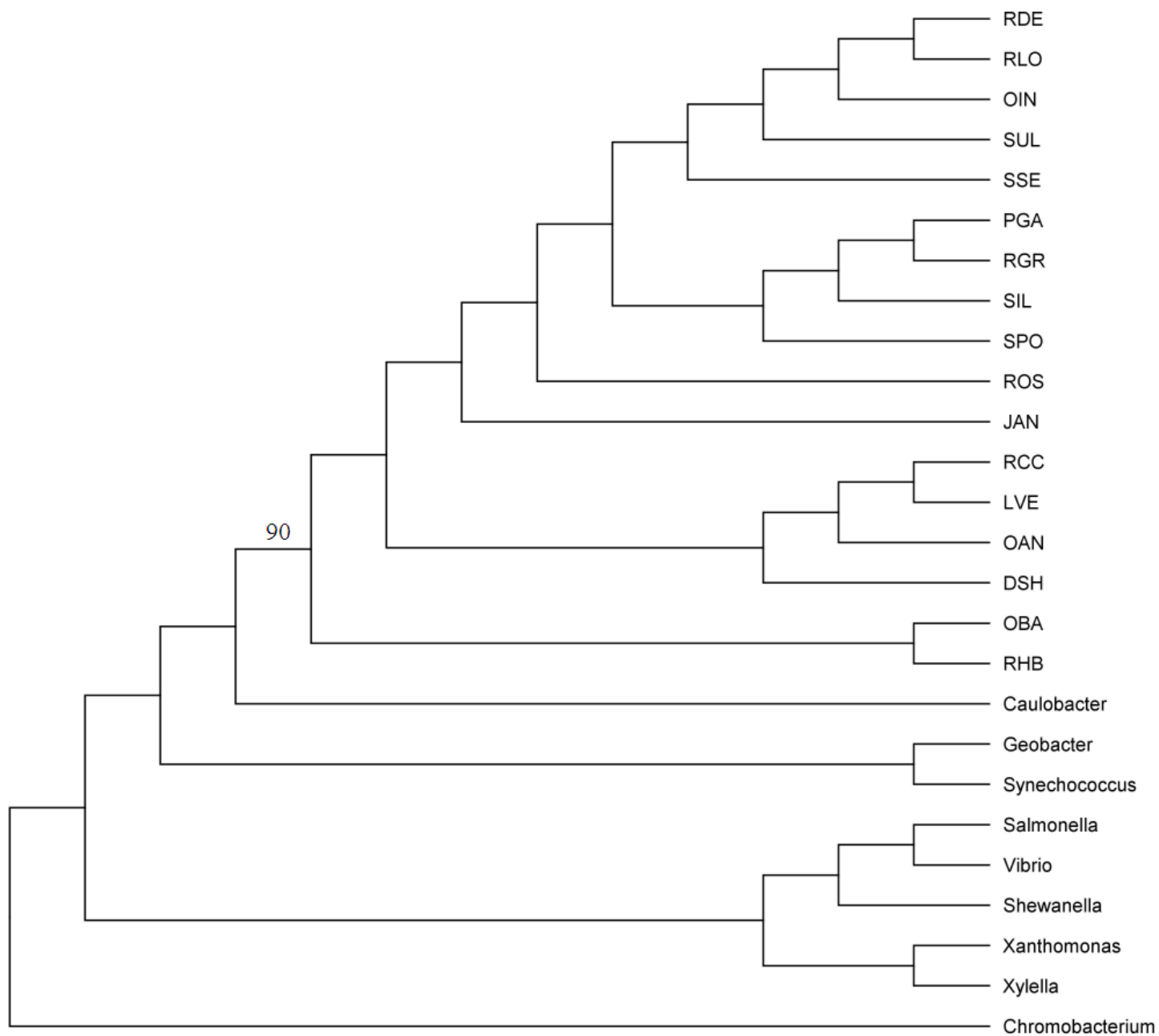

Supplement: File S2 — Tree topologies with the extended data. The multi-documents have been combined into a single ZIP-formatted file. The trees should be considered unrooted. The tree topologies were calculated in PhyML as described in Methods. Numbers refer to bootstrap values. The tree topology (separate pdf) shows that Roseobacter bacteria form a monophyletic group and was deposited in a document named “high bootstrap”. The other organisms embedded within the Roseobacter clade, or Roseobacter bacteria embedded within other phyla are shown in red (deposited in a document named “inter-phylum”). Individual file name corresponds to gene family code listed in Table S1. The non Roseobacter organism taxonomic name is detailed in the amino acid fasta of the sequences (a document named “sequences”). (4.62 MB ZIP) [file pone.0011604.s008.zip › high bootstrap/ort376.pdf]

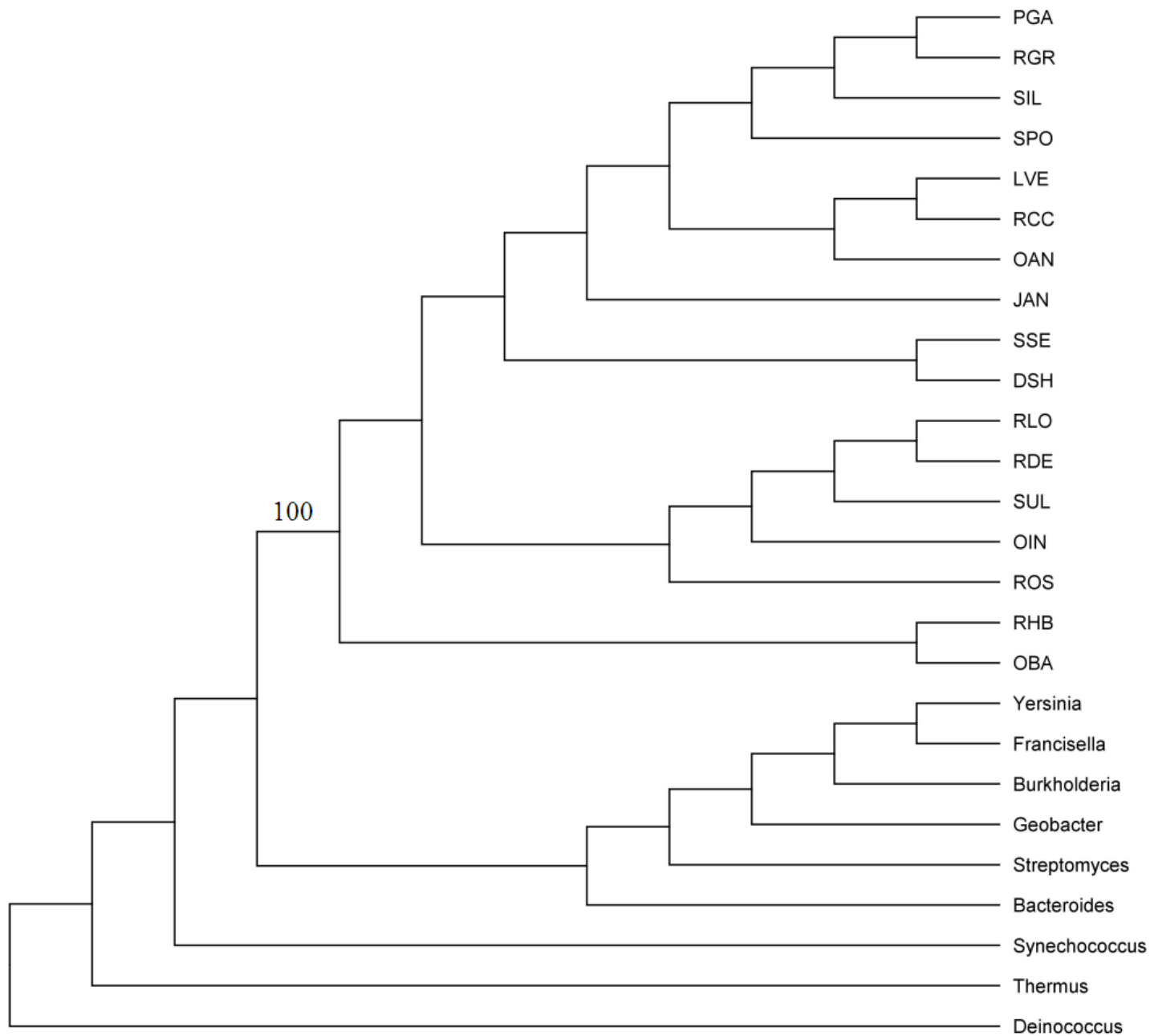

Supplement: File S2 — Tree topologies with the extended data. The multi-documents have been combined into a single ZIP-formatted file. The trees should be considered unrooted. The tree topologies were calculated in PhyML as described in Methods. Numbers refer to bootstrap values. The tree topology (separate pdf) shows that Roseobacter bacteria form a monophyletic group and was deposited in a document named “high bootstrap”. The other organisms embedded within the Roseobacter clade, or Roseobacter bacteria embedded within other phyla are shown in red (deposited in a document named “inter-phylum”). Individual file name corresponds to gene family code listed in Table S1. The non Roseobacter organism taxonomic name is detailed in the amino acid fasta of the sequences (a document named “sequences”). (4.62 MB ZIP) [file pone.0011604.s008.zip › high bootstrap/ort38.pdf]

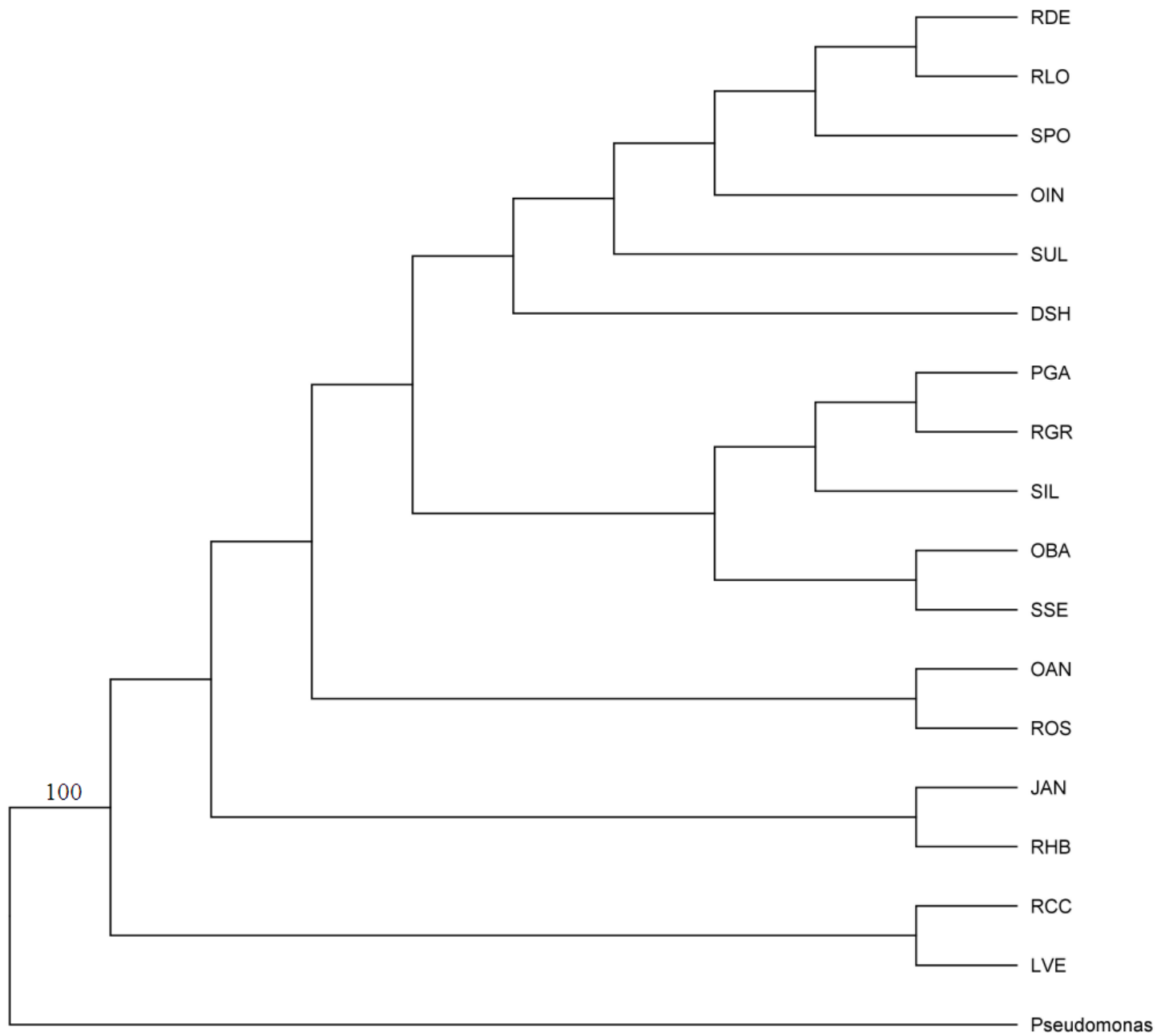

Supplement: File S2 — Tree topologies with the extended data. The multi-documents have been combined into a single ZIP-formatted file. The trees should be considered unrooted. The tree topologies were calculated in PhyML as described in Methods. Numbers refer to bootstrap values. The tree topology (separate pdf) shows that Roseobacter bacteria form a monophyletic group and was deposited in a document named “high bootstrap”. The other organisms embedded within the Roseobacter clade, or Roseobacter bacteria embedded within other phyla are shown in red (deposited in a document named “inter-phylum”). Individual file name corresponds to gene family code listed in Table S1. The non Roseobacter organism taxonomic name is detailed in the amino acid fasta of the sequences (a document named “sequences”). (4.62 MB ZIP) [file pone.0011604.s008.zip › high bootstrap/ort380.pdf]

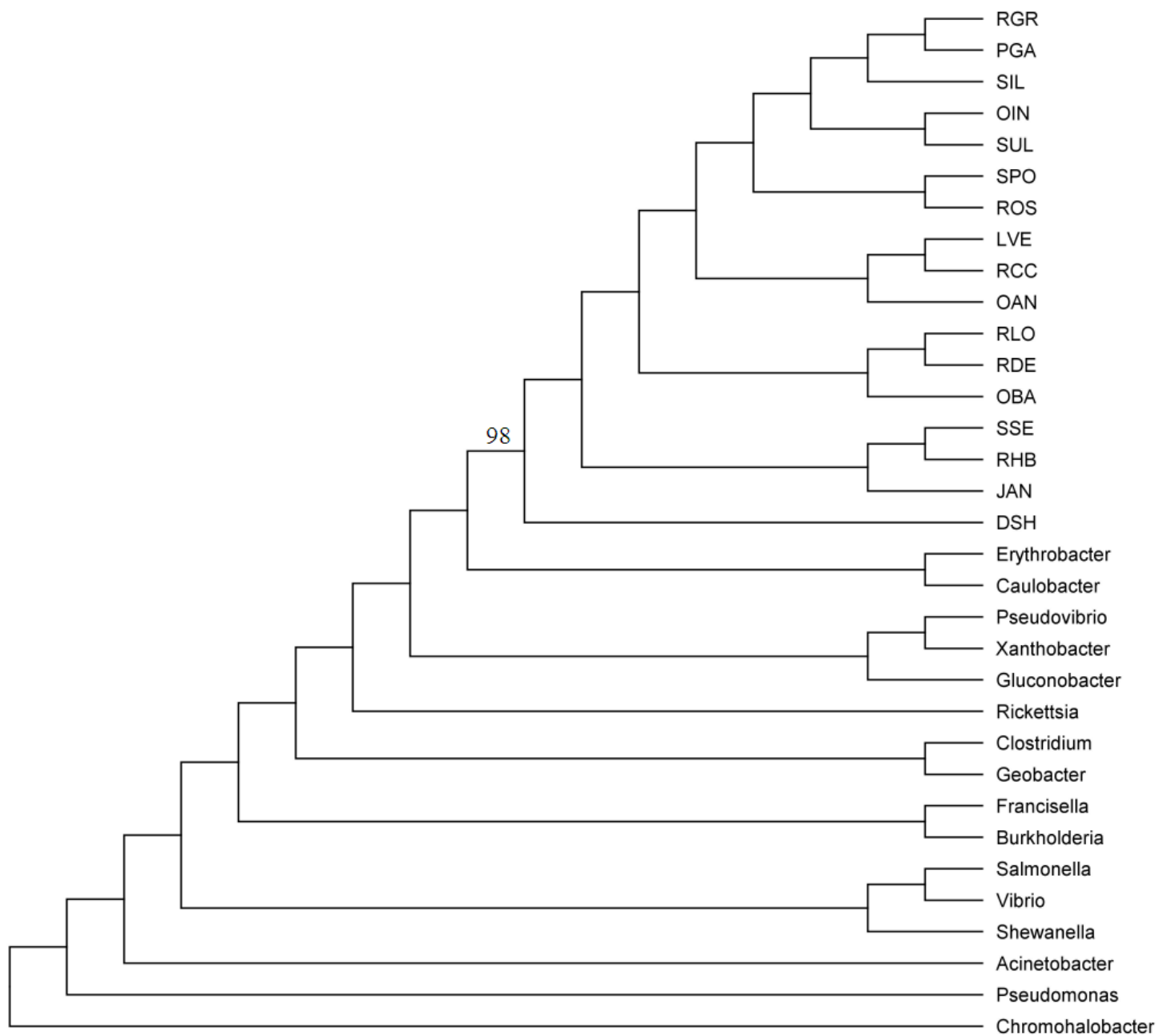

Supplement: File S2 — Tree topologies with the extended data. The multi-documents have been combined into a single ZIP-formatted file. The trees should be considered unrooted. The tree topologies were calculated in PhyML as described in Methods. Numbers refer to bootstrap values. The tree topology (separate pdf) shows that Roseobacter bacteria form a monophyletic group and was deposited in a document named “high bootstrap”. The other organisms embedded within the Roseobacter clade, or Roseobacter bacteria embedded within other phyla are shown in red (deposited in a document named “inter-phylum”). Individual file name corresponds to gene family code listed in Table S1. The non Roseobacter organism taxonomic name is detailed in the amino acid fasta of the sequences (a document named “sequences”). (4.62 MB ZIP) [file pone.0011604.s008.zip › high bootstrap/ort395.pdf]

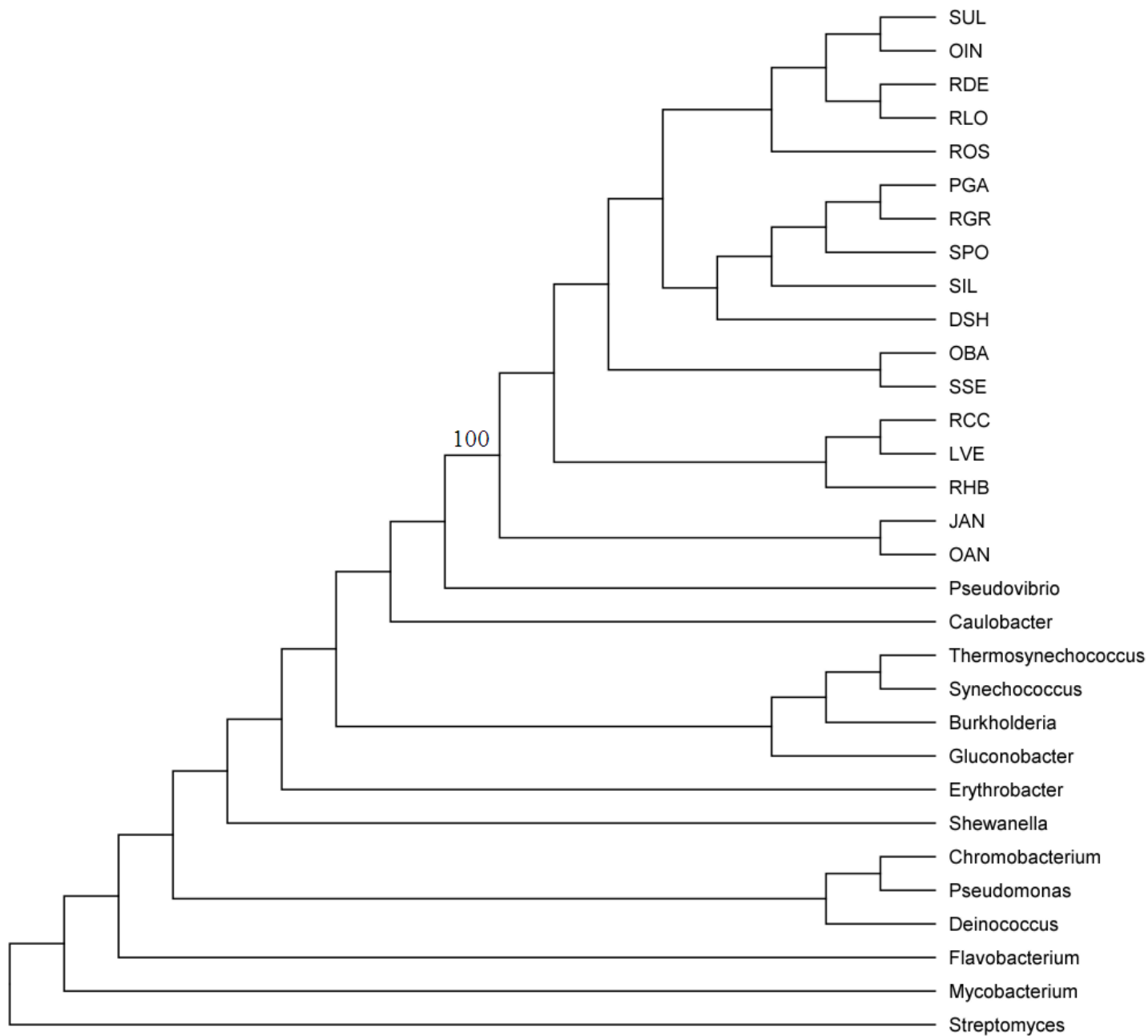

Supplement: File S2 — Tree topologies with the extended data. The multi-documents have been combined into a single ZIP-formatted file. The trees should be considered unrooted. The tree topologies were calculated in PhyML as described in Methods. Numbers refer to bootstrap values. The tree topology (separate pdf) shows that Roseobacter bacteria form a monophyletic group and was deposited in a document named “high bootstrap”. The other organisms embedded within the Roseobacter clade, or Roseobacter bacteria embedded within other phyla are shown in red (deposited in a document named “inter-phylum”). Individual file name corresponds to gene family code listed in Table S1. The non Roseobacter organism taxonomic name is detailed in the amino acid fasta of the sequences (a document named “sequences”). (4.62 MB ZIP) [file pone.0011604.s008.zip › high bootstrap/ort405.pdf]

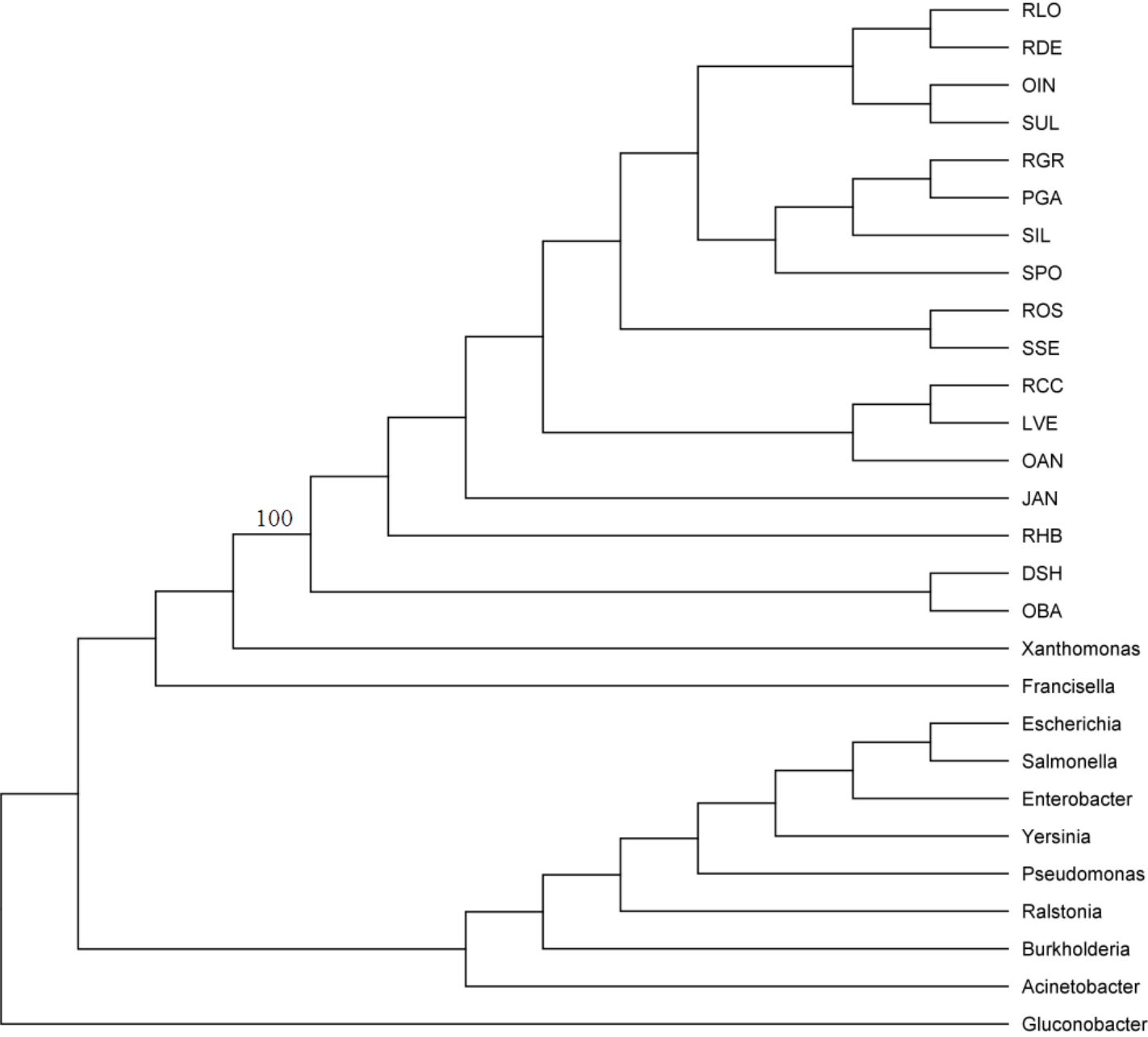

Supplement: File S2 — Tree topologies with the extended data. The multi-documents have been combined into a single ZIP-formatted file. The trees should be considered unrooted. The tree topologies were calculated in PhyML as described in Methods. Numbers refer to bootstrap values. The tree topology (separate pdf) shows that Roseobacter bacteria form a monophyletic group and was deposited in a document named “high bootstrap”. The other organisms embedded within the Roseobacter clade, or Roseobacter bacteria embedded within other phyla are shown in red (deposited in a document named “inter-phylum”). Individual file name corresponds to gene family code listed in Table S1. The non Roseobacter organism taxonomic name is detailed in the amino acid fasta of the sequences (a document named “sequences”). (4.62 MB ZIP) [file pone.0011604.s008.zip › high bootstrap/ort406.pdf]

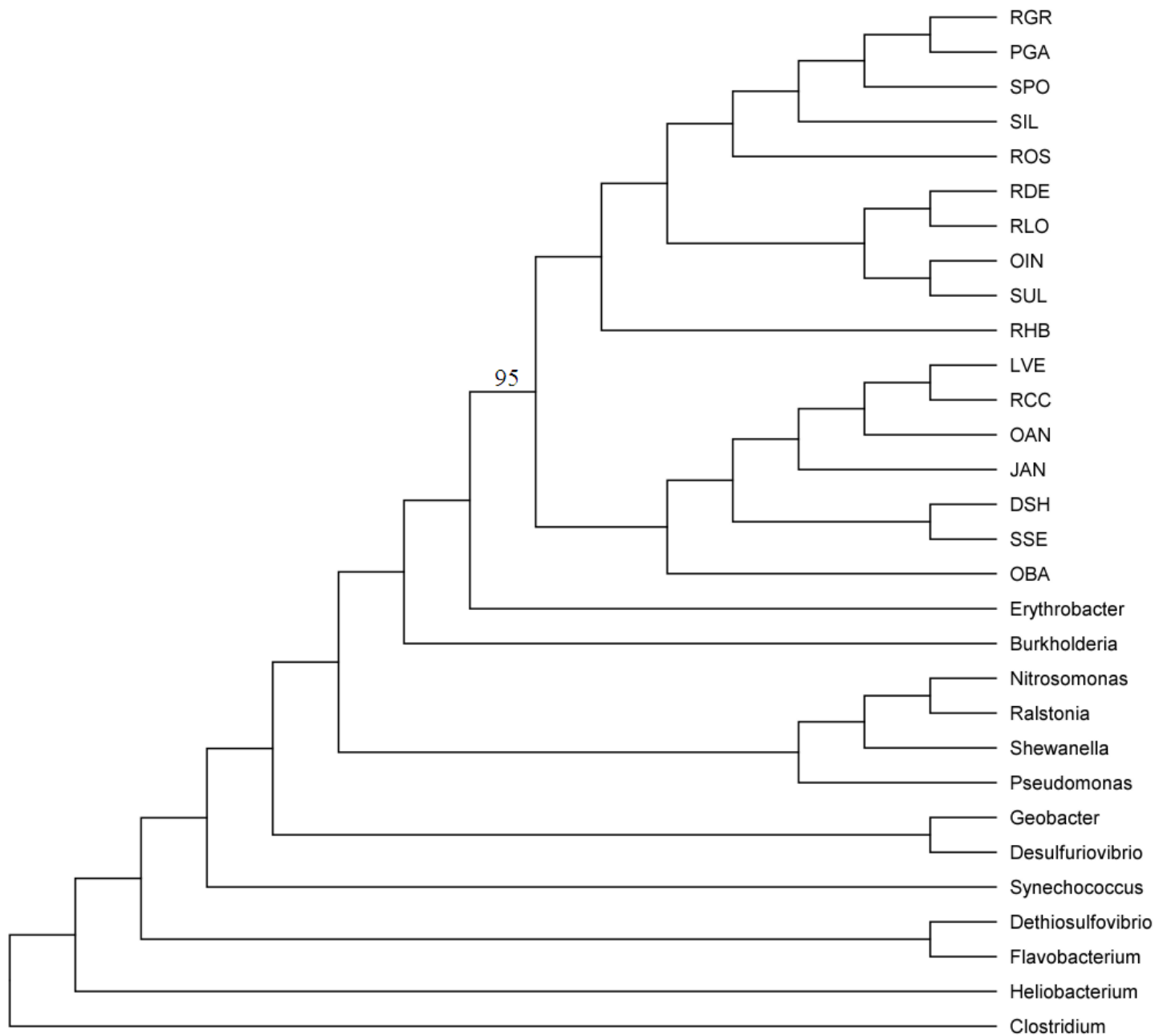

Supplement: File S2 — Tree topologies with the extended data. The multi-documents have been combined into a single ZIP-formatted file. The trees should be considered unrooted. The tree topologies were calculated in PhyML as described in Methods. Numbers refer to bootstrap values. The tree topology (separate pdf) shows that Roseobacter bacteria form a monophyletic group and was deposited in a document named “high bootstrap”. The other organisms embedded within the Roseobacter clade, or Roseobacter bacteria embedded within other phyla are shown in red (deposited in a document named “inter-phylum”). Individual file name corresponds to gene family code listed in Table S1. The non Roseobacter organism taxonomic name is detailed in the amino acid fasta of the sequences (a document named “sequences”). (4.62 MB ZIP) [file pone.0011604.s008.zip › high bootstrap/ort414.pdf]

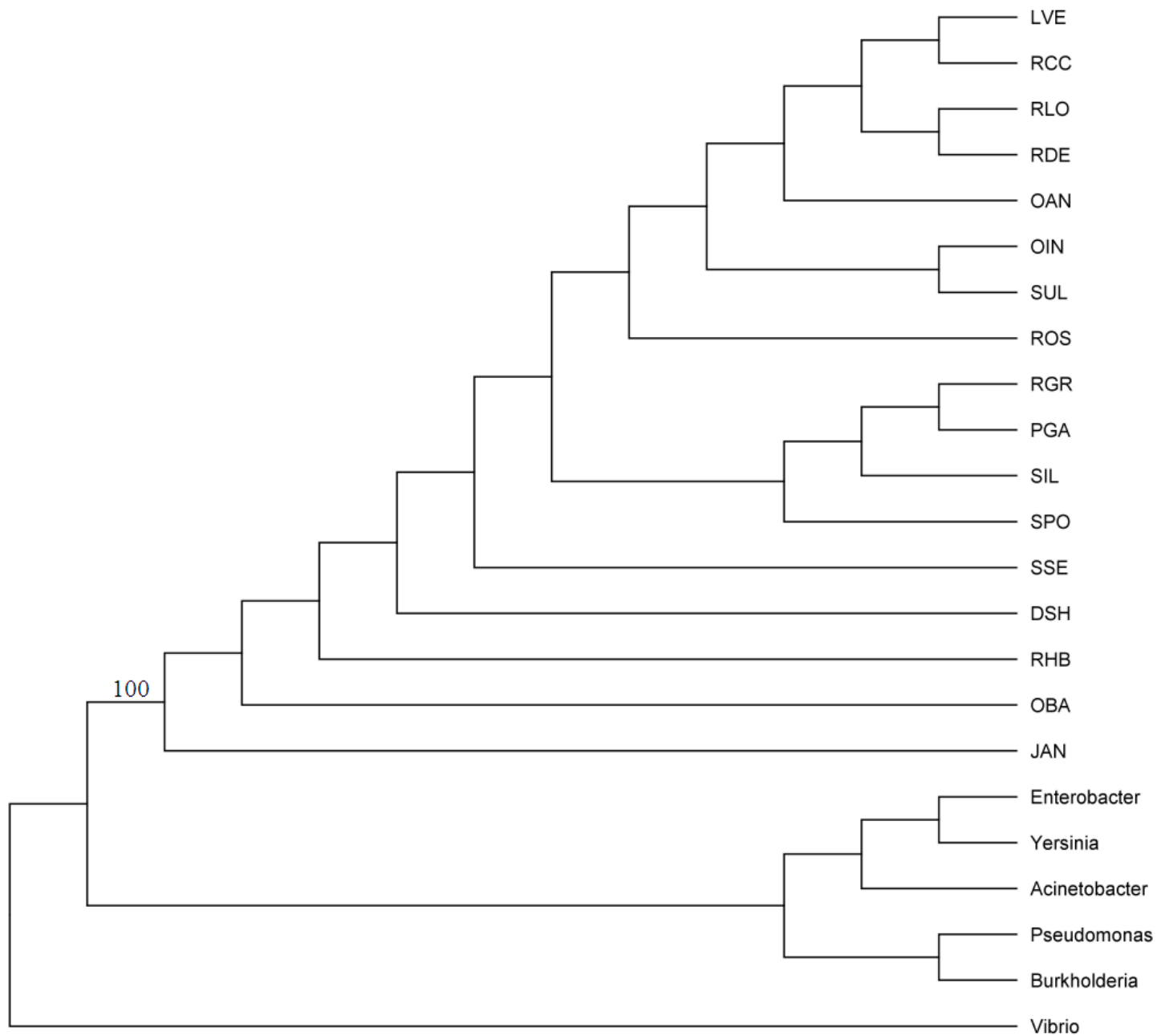

Supplement: File S2 — Tree topologies with the extended data. The multi-documents have been combined into a single ZIP-formatted file. The trees should be considered unrooted. The tree topologies were calculated in PhyML as described in Methods. Numbers refer to bootstrap values. The tree topology (separate pdf) shows that Roseobacter bacteria form a monophyletic group and was deposited in a document named “high bootstrap”. The other organisms embedded within the Roseobacter clade, or Roseobacter bacteria embedded within other phyla are shown in red (deposited in a document named “inter-phylum”). Individual file name corresponds to gene family code listed in Table S1. The non Roseobacter organism taxonomic name is detailed in the amino acid fasta of the sequences (a document named “sequences”). (4.62 MB ZIP) [file pone.0011604.s008.zip › high bootstrap/ort426.pdf]

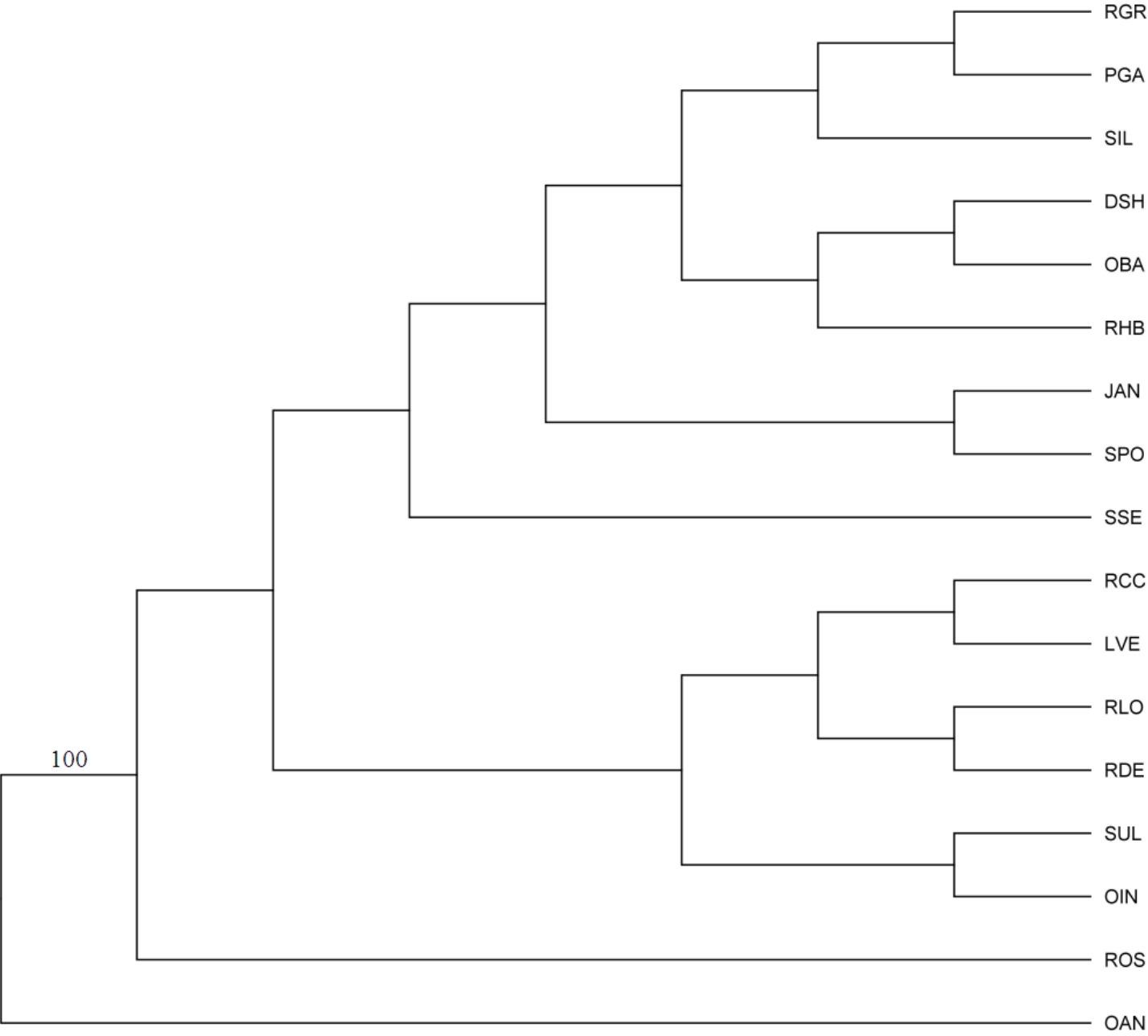

Supplement: File S2 — Tree topologies with the extended data. The multi-documents have been combined into a single ZIP-formatted file. The trees should be considered unrooted. The tree topologies were calculated in PhyML as described in Methods. Numbers refer to bootstrap values. The tree topology (separate pdf) shows that Roseobacter bacteria form a monophyletic group and was deposited in a document named “high bootstrap”. The other organisms embedded within the Roseobacter clade, or Roseobacter bacteria embedded within other phyla are shown in red (deposited in a document named “inter-phylum”). Individual file name corresponds to gene family code listed in Table S1. The non Roseobacter organism taxonomic name is detailed in the amino acid fasta of the sequences (a document named “sequences”). (4.62 MB ZIP) [file pone.0011604.s008.zip › high bootstrap/ort427.pdf]

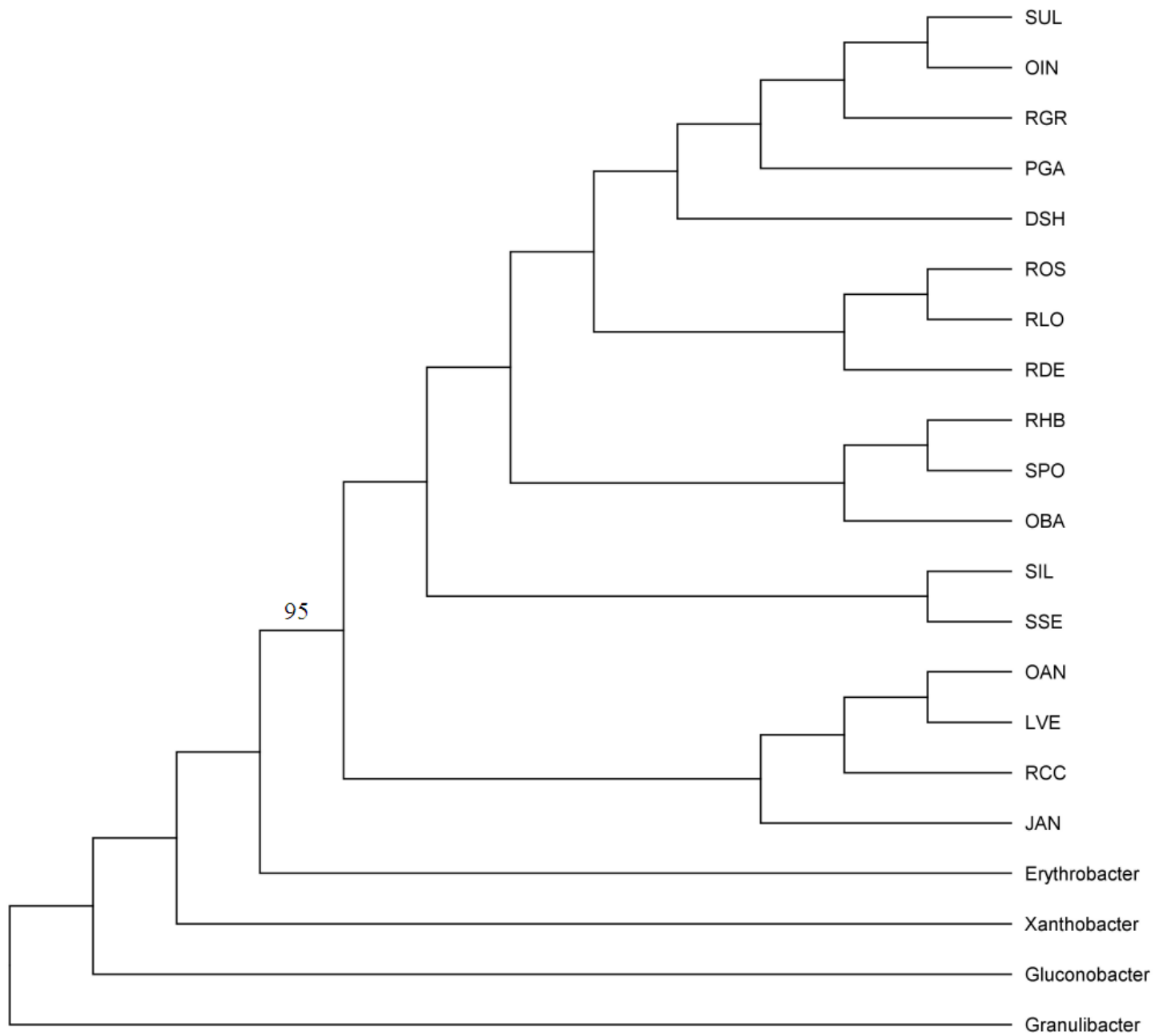

Supplement: File S2 — Tree topologies with the extended data. The multi-documents have been combined into a single ZIP-formatted file. The trees should be considered unrooted. The tree topologies were calculated in PhyML as described in Methods. Numbers refer to bootstrap values. The tree topology (separate pdf) shows that Roseobacter bacteria form a monophyletic group and was deposited in a document named “high bootstrap”. The other organisms embedded within the Roseobacter clade, or Roseobacter bacteria embedded within other phyla are shown in red (deposited in a document named “inter-phylum”). Individual file name corresponds to gene family code listed in Table S1. The non Roseobacter organism taxonomic name is detailed in the amino acid fasta of the sequences (a document named “sequences”). (4.62 MB ZIP) [file pone.0011604.s008.zip › high bootstrap/ort441.pdf]

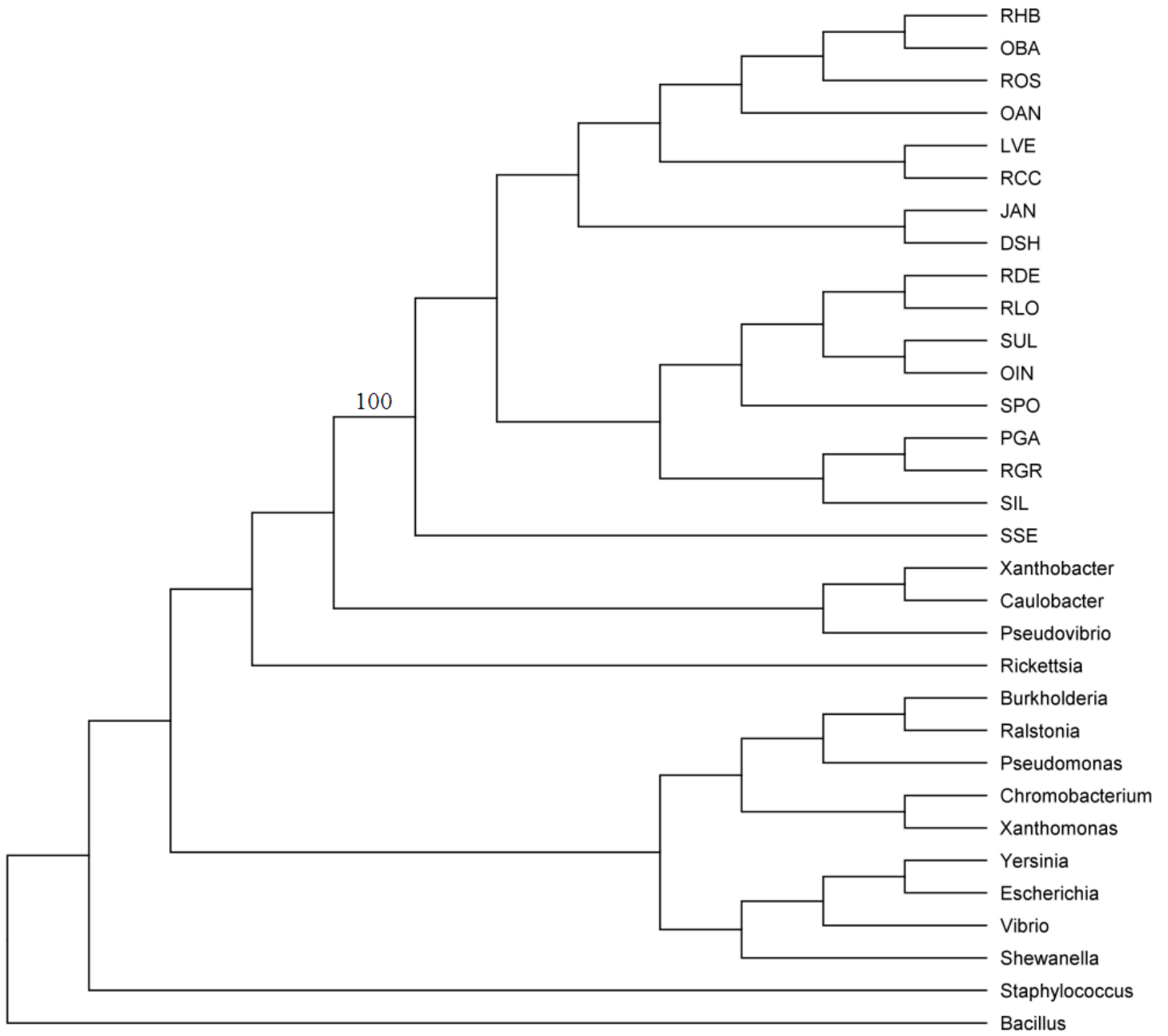

Supplement: File S2 — Tree topologies with the extended data. The multi-documents have been combined into a single ZIP-formatted file. The trees should be considered unrooted. The tree topologies were calculated in PhyML as described in Methods. Numbers refer to bootstrap values. The tree topology (separate pdf) shows that Roseobacter bacteria form a monophyletic group and was deposited in a document named “high bootstrap”. The other organisms embedded within the Roseobacter clade, or Roseobacter bacteria embedded within other phyla are shown in red (deposited in a document named “inter-phylum”). Individual file name corresponds to gene family code listed in Table S1. The non Roseobacter organism taxonomic name is detailed in the amino acid fasta of the sequences (a document named “sequences”). (4.62 MB ZIP) [file pone.0011604.s008.zip › high bootstrap/ort444.pdf]

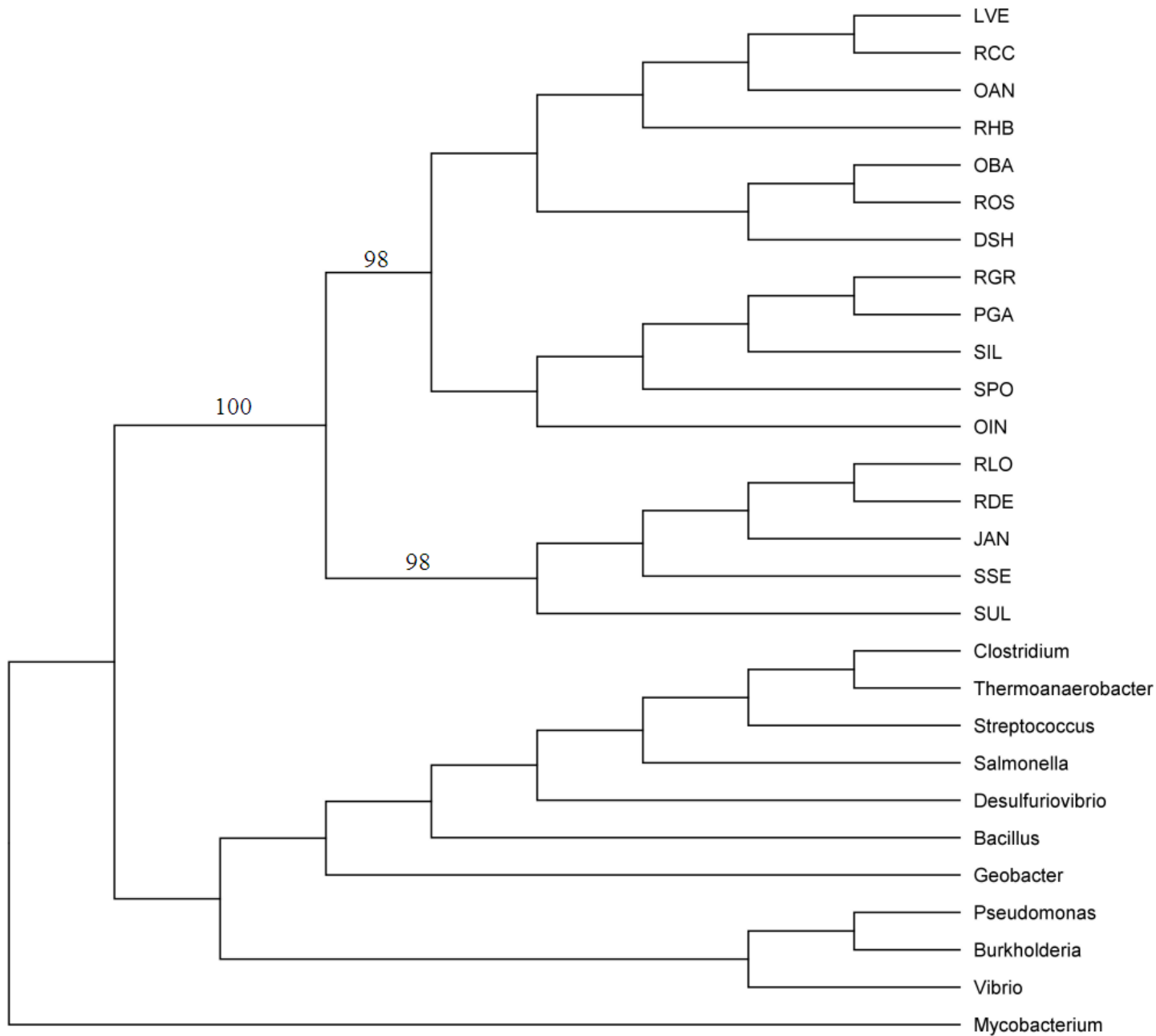

Supplement: File S2 — Tree topologies with the extended data. The multi-documents have been combined into a single ZIP-formatted file. The trees should be considered unrooted. The tree topologies were calculated in PhyML as described in Methods. Numbers refer to bootstrap values. The tree topology (separate pdf) shows that Roseobacter bacteria form a monophyletic group and was deposited in a document named “high bootstrap”. The other organisms embedded within the Roseobacter clade, or Roseobacter bacteria embedded within other phyla are shown in red (deposited in a document named “inter-phylum”). Individual file name corresponds to gene family code listed in Table S1. The non Roseobacter organism taxonomic name is detailed in the amino acid fasta of the sequences (a document named “sequences”). (4.62 MB ZIP) [file pone.0011604.s008.zip › high bootstrap/ort477.pdf]

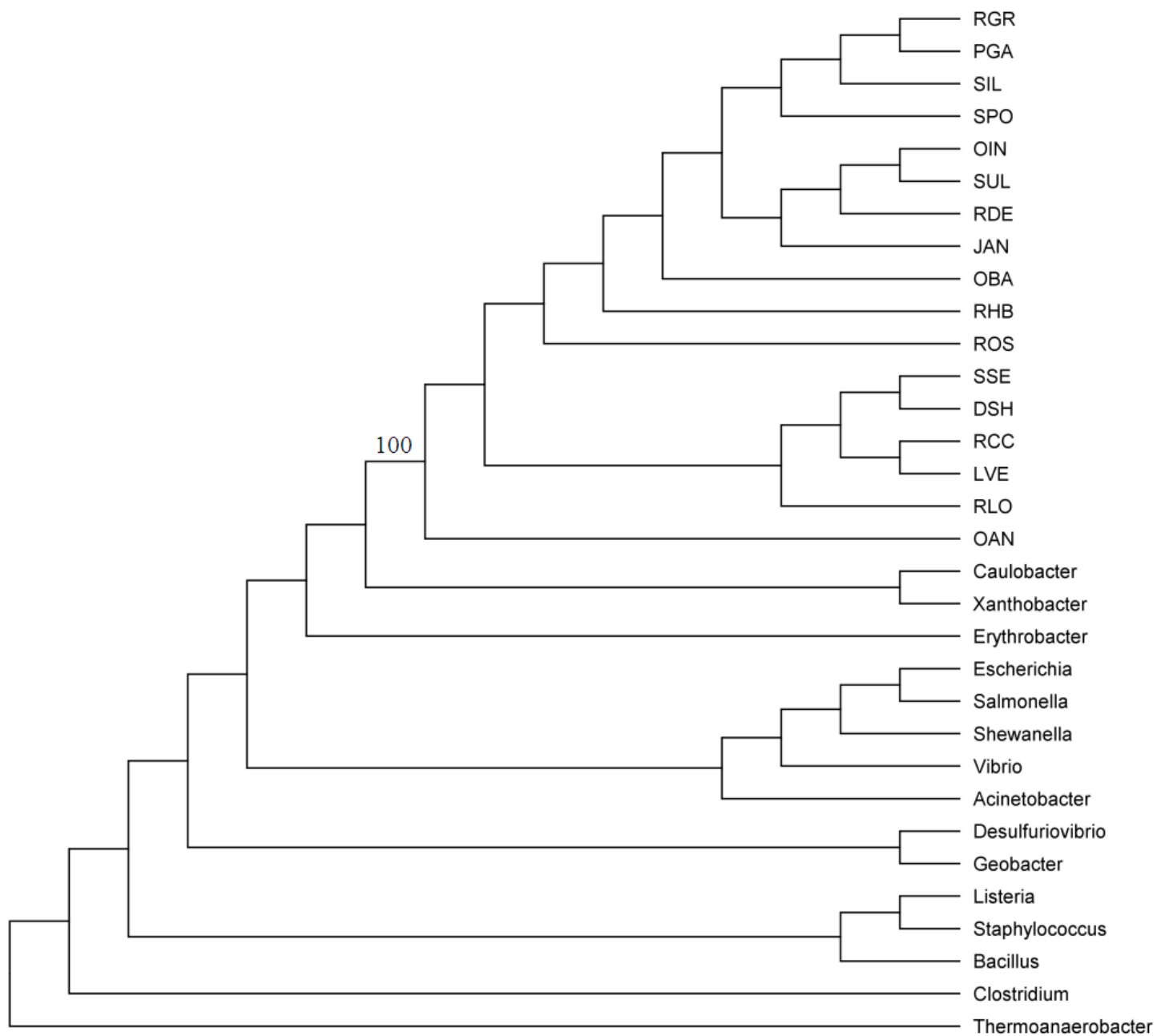

Supplement: File S2 — Tree topologies with the extended data. The multi-documents have been combined into a single ZIP-formatted file. The trees should be considered unrooted. The tree topologies were calculated in PhyML as described in Methods. Numbers refer to bootstrap values. The tree topology (separate pdf) shows that Roseobacter bacteria form a monophyletic group and was deposited in a document named “high bootstrap”. The other organisms embedded within the Roseobacter clade, or Roseobacter bacteria embedded within other phyla are shown in red (deposited in a document named “inter-phylum”). Individual file name corresponds to gene family code listed in Table S1. The non Roseobacter organism taxonomic name is detailed in the amino acid fasta of the sequences (a document named “sequences”). (4.62 MB ZIP) [file pone.0011604.s008.zip › high bootstrap/ort499.pdf]

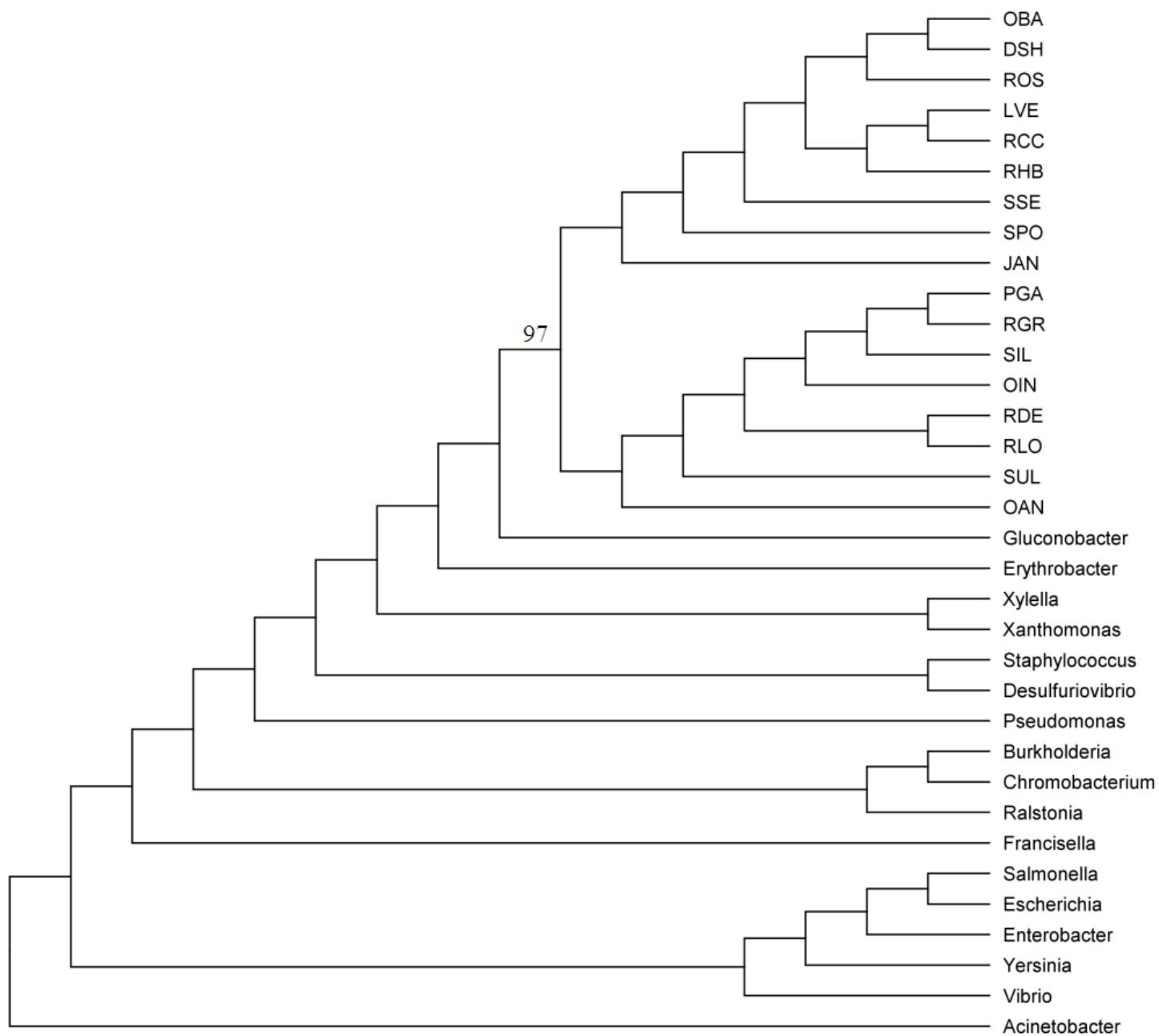

Supplement: File S2 — Tree topologies with the extended data. The multi-documents have been combined into a single ZIP-formatted file. The trees should be considered unrooted. The tree topologies were calculated in PhyML as described in Methods. Numbers refer to bootstrap values. The tree topology (separate pdf) shows that Roseobacter bacteria form a monophyletic group and was deposited in a document named “high bootstrap”. The other organisms embedded within the Roseobacter clade, or Roseobacter bacteria embedded within other phyla are shown in red (deposited in a document named “inter-phylum”). Individual file name corresponds to gene family code listed in Table S1. The non Roseobacter organism taxonomic name is detailed in the amino acid fasta of the sequences (a document named “sequences”). (4.62 MB ZIP) [file pone.0011604.s008.zip › high bootstrap/ort512.pdf]

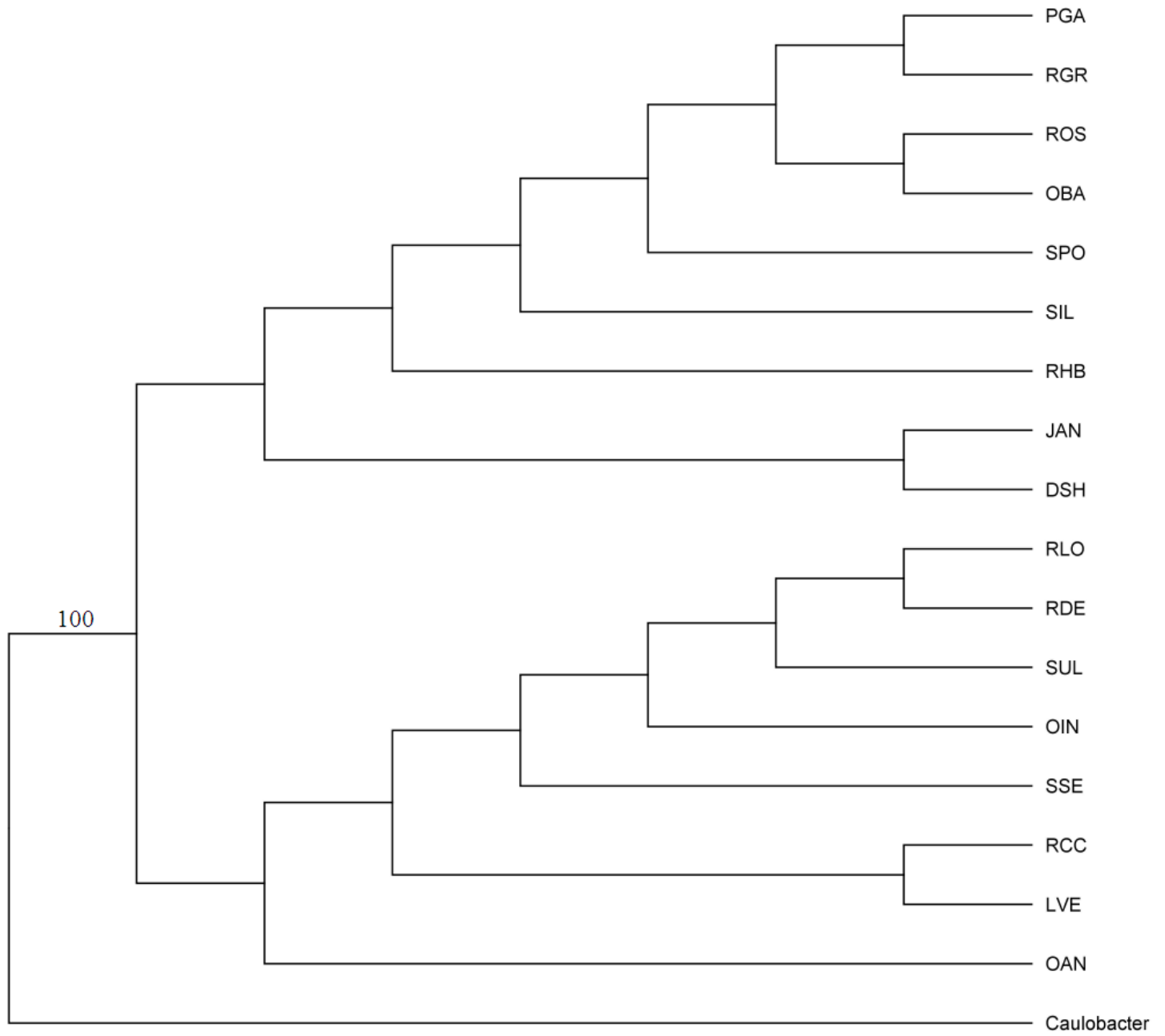

Supplement: File S2 — Tree topologies with the extended data. The multi-documents have been combined into a single ZIP-formatted file. The trees should be considered unrooted. The tree topologies were calculated in PhyML as described in Methods. Numbers refer to bootstrap values. The tree topology (separate pdf) shows that Roseobacter bacteria form a monophyletic group and was deposited in a document named “high bootstrap”. The other organisms embedded within the Roseobacter clade, or Roseobacter bacteria embedded within other phyla are shown in red (deposited in a document named “inter-phylum”). Individual file name corresponds to gene family code listed in Table S1. The non Roseobacter organism taxonomic name is detailed in the amino acid fasta of the sequences (a document named “sequences”). (4.62 MB ZIP) [file pone.0011604.s008.zip › high bootstrap/ort518.pdf]

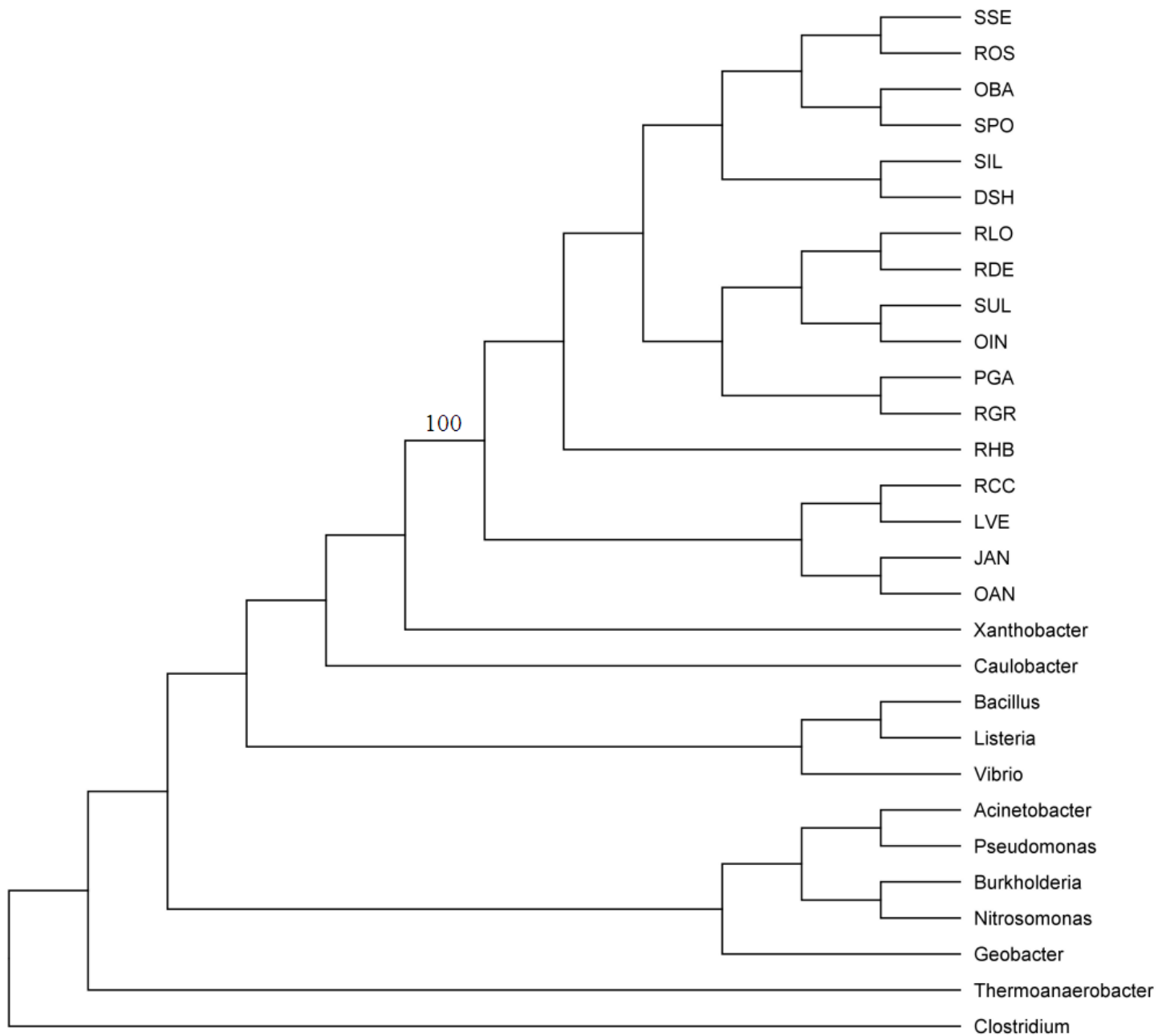

Supplement: File S2 — Tree topologies with the extended data. The multi-documents have been combined into a single ZIP-formatted file. The trees should be considered unrooted. The tree topologies were calculated in PhyML as described in Methods. Numbers refer to bootstrap values. The tree topology (separate pdf) shows that Roseobacter bacteria form a monophyletic group and was deposited in a document named “high bootstrap”. The other organisms embedded within the Roseobacter clade, or Roseobacter bacteria embedded within other phyla are shown in red (deposited in a document named “inter-phylum”). Individual file name corresponds to gene family code listed in Table S1. The non Roseobacter organism taxonomic name is detailed in the amino acid fasta of the sequences (a document named “sequences”). (4.62 MB ZIP) [file pone.0011604.s008.zip › high bootstrap/ort533.pdf]

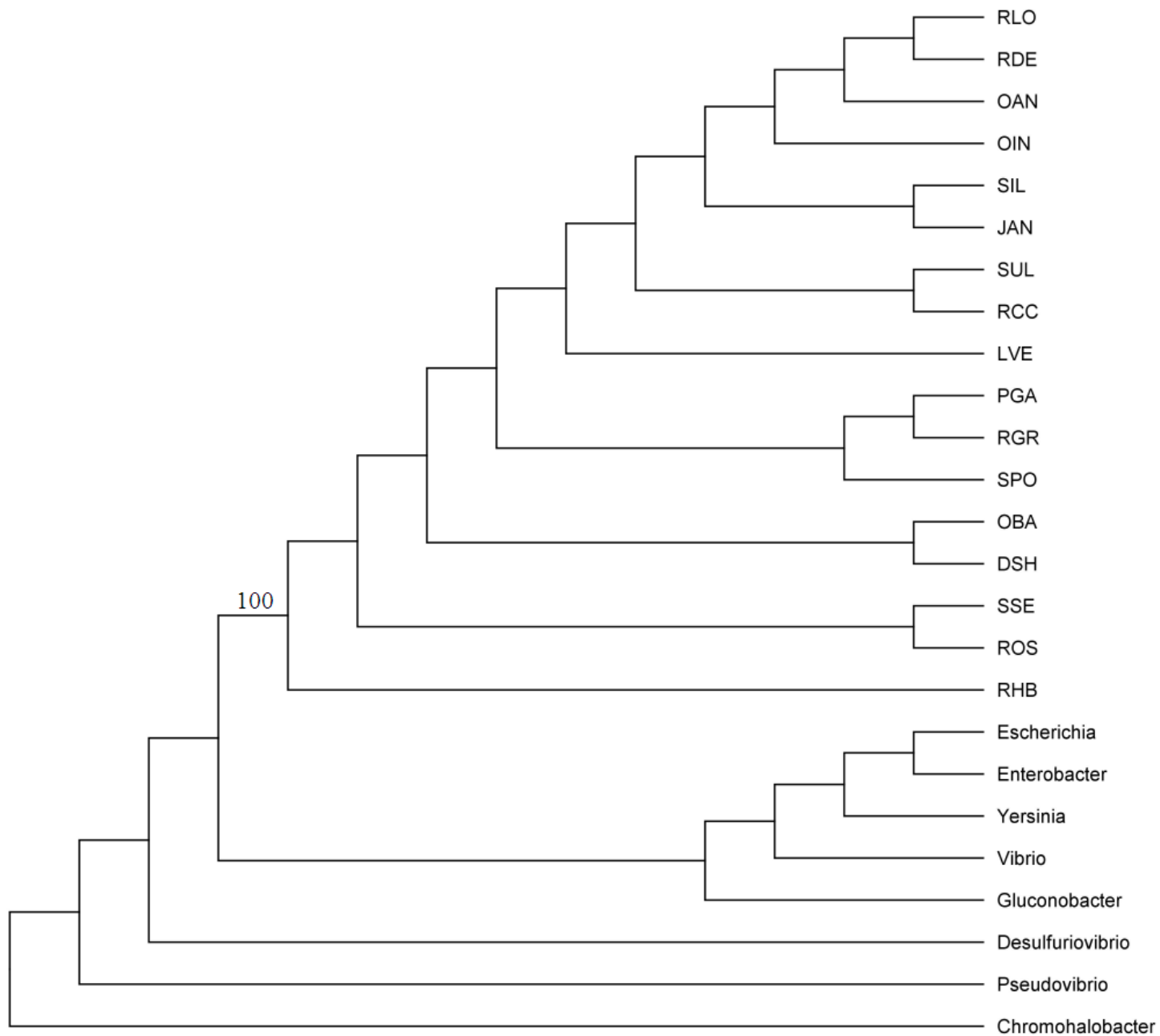

Supplement: File S2 — Tree topologies with the extended data. The multi-documents have been combined into a single ZIP-formatted file. The trees should be considered unrooted. The tree topologies were calculated in PhyML as described in Methods. Numbers refer to bootstrap values. The tree topology (separate pdf) shows that Roseobacter bacteria form a monophyletic group and was deposited in a document named “high bootstrap”. The other organisms embedded within the Roseobacter clade, or Roseobacter bacteria embedded within other phyla are shown in red (deposited in a document named “inter-phylum”). Individual file name corresponds to gene family code listed in Table S1. The non Roseobacter organism taxonomic name is detailed in the amino acid fasta of the sequences (a document named “sequences”). (4.62 MB ZIP) [file pone.0011604.s008.zip › high bootstrap/ort538.pdf]

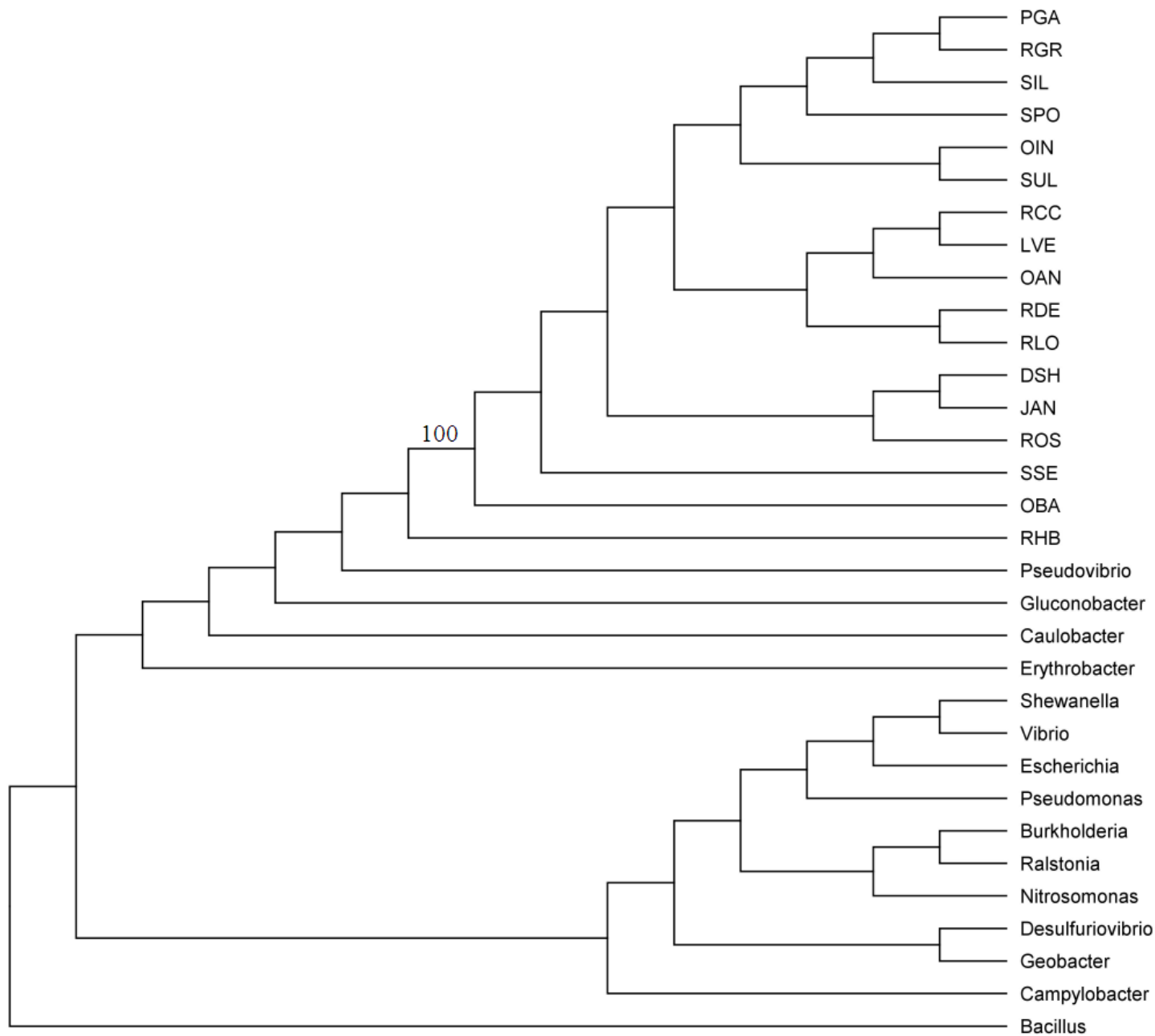

Supplement: File S2 — Tree topologies with the extended data. The multi-documents have been combined into a single ZIP-formatted file. The trees should be considered unrooted. The tree topologies were calculated in PhyML as described in Methods. Numbers refer to bootstrap values. The tree topology (separate pdf) shows that Roseobacter bacteria form a monophyletic group and was deposited in a document named “high bootstrap”. The other organisms embedded within the Roseobacter clade, or Roseobacter bacteria embedded within other phyla are shown in red (deposited in a document named “inter-phylum”). Individual file name corresponds to gene family code listed in Table S1. The non Roseobacter organism taxonomic name is detailed in the amino acid fasta of the sequences (a document named “sequences”). (4.62 MB ZIP) [file pone.0011604.s008.zip › high bootstrap/ort545.pdf]

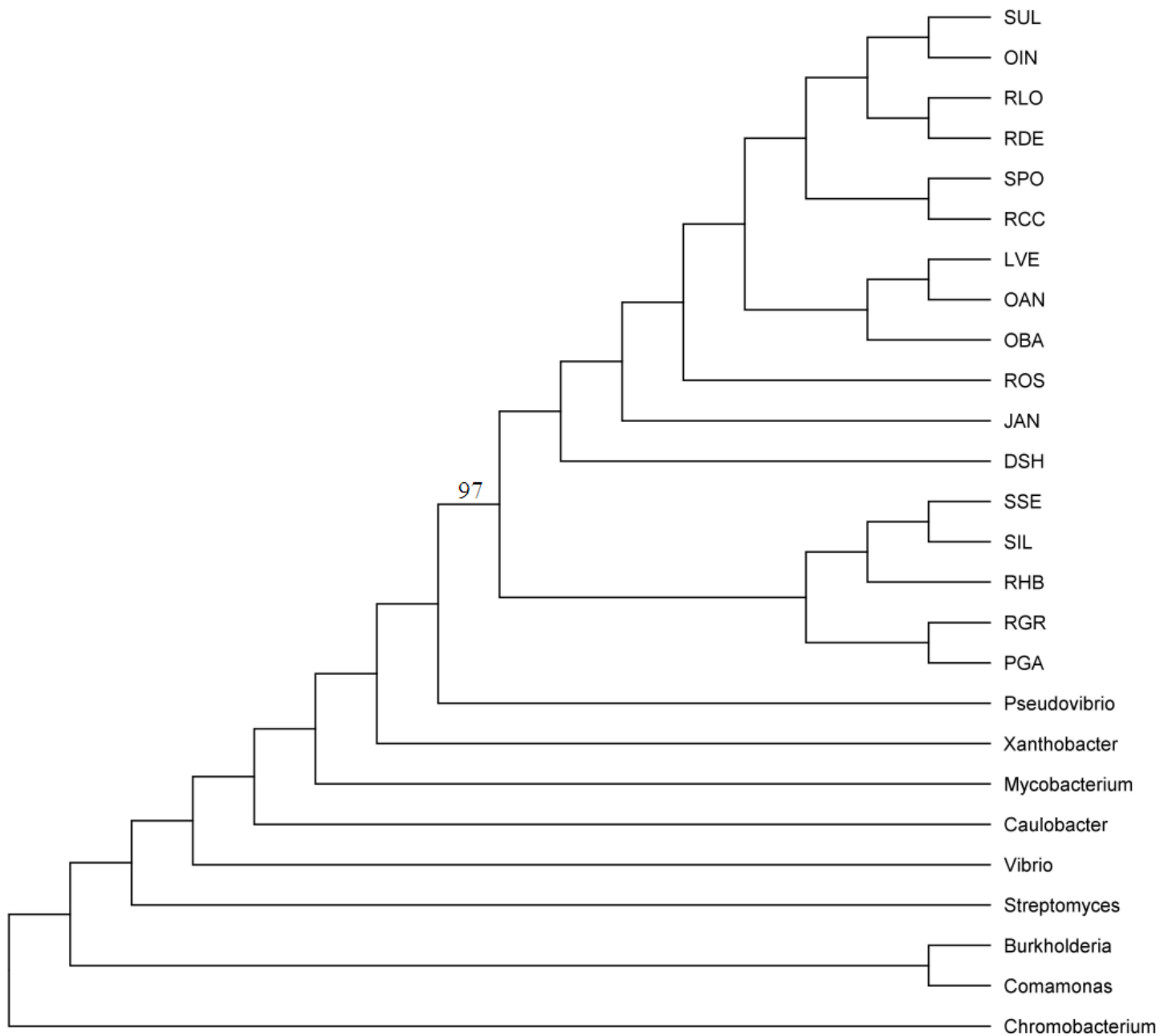

Supplement: File S2 — Tree topologies with the extended data. The multi-documents have been combined into a single ZIP-formatted file. The trees should be considered unrooted. The tree topologies were calculated in PhyML as described in Methods. Numbers refer to bootstrap values. The tree topology (separate pdf) shows that Roseobacter bacteria form a monophyletic group and was deposited in a document named “high bootstrap”. The other organisms embedded within the Roseobacter clade, or Roseobacter bacteria embedded within other phyla are shown in red (deposited in a document named “inter-phylum”). Individual file name corresponds to gene family code listed in Table S1. The non Roseobacter organism taxonomic name is detailed in the amino acid fasta of the sequences (a document named “sequences”). (4.62 MB ZIP) [file pone.0011604.s008.zip › high bootstrap/ort559.pdf]

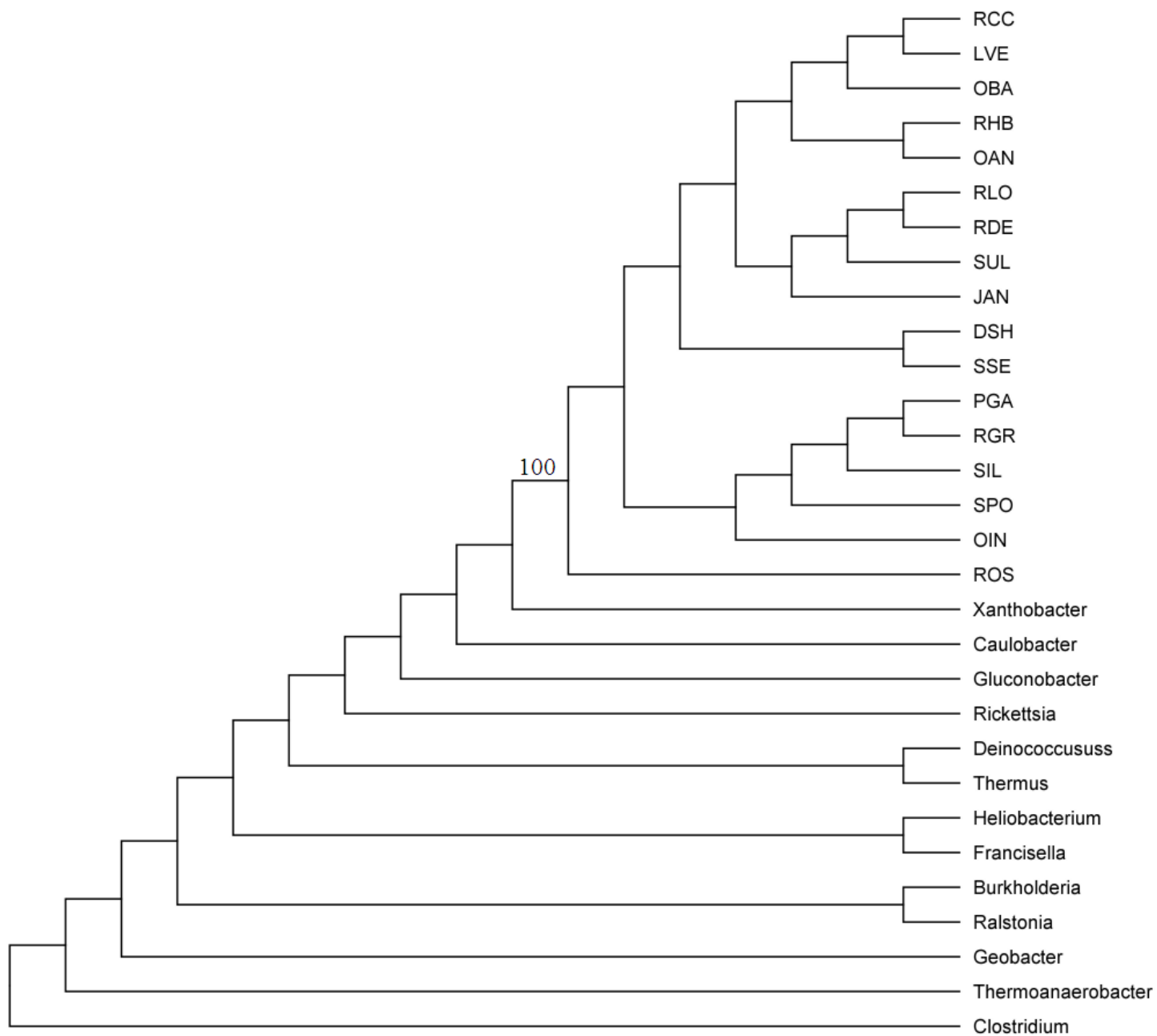

Supplement: File S2 — Tree topologies with the extended data. The multi-documents have been combined into a single ZIP-formatted file. The trees should be considered unrooted. The tree topologies were calculated in PhyML as described in Methods. Numbers refer to bootstrap values. The tree topology (separate pdf) shows that Roseobacter bacteria form a monophyletic group and was deposited in a document named “high bootstrap”. The other organisms embedded within the Roseobacter clade, or Roseobacter bacteria embedded within other phyla are shown in red (deposited in a document named “inter-phylum”). Individual file name corresponds to gene family code listed in Table S1. The non Roseobacter organism taxonomic name is detailed in the amino acid fasta of the sequences (a document named “sequences”). (4.62 MB ZIP) [file pone.0011604.s008.zip › high bootstrap/ort571.pdf]

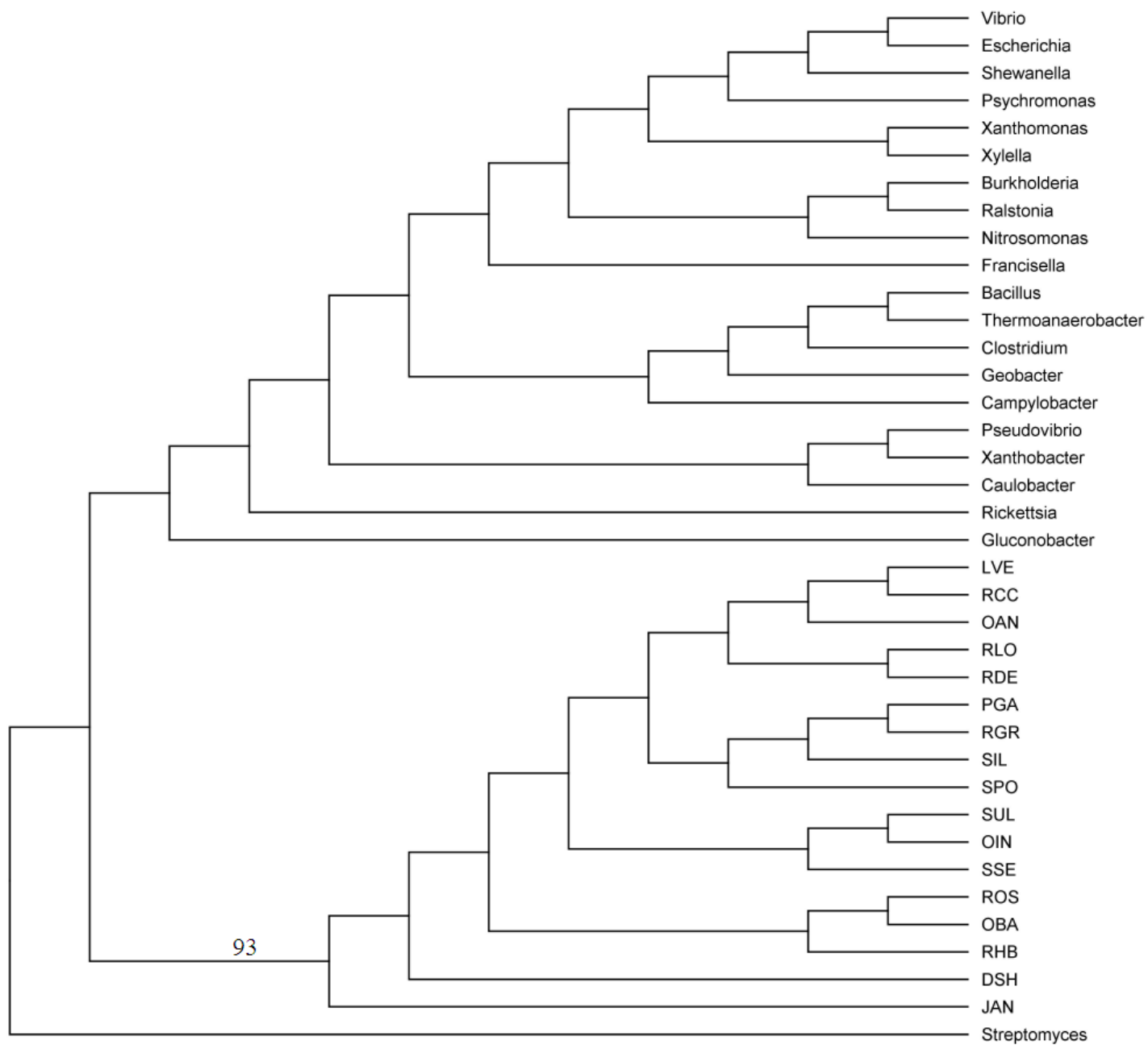

Supplement: File S2 — Tree topologies with the extended data. The multi-documents have been combined into a single ZIP-formatted file. The trees should be considered unrooted. The tree topologies were calculated in PhyML as described in Methods. Numbers refer to bootstrap values. The tree topology (separate pdf) shows that Roseobacter bacteria form a monophyletic group and was deposited in a document named “high bootstrap”. The other organisms embedded within the Roseobacter clade, or Roseobacter bacteria embedded within other phyla are shown in red (deposited in a document named “inter-phylum”). Individual file name corresponds to gene family code listed in Table S1. The non Roseobacter organism taxonomic name is detailed in the amino acid fasta of the sequences (a document named “sequences”). (4.62 MB ZIP) [file pone.0011604.s008.zip › high bootstrap/ort580.pdf]

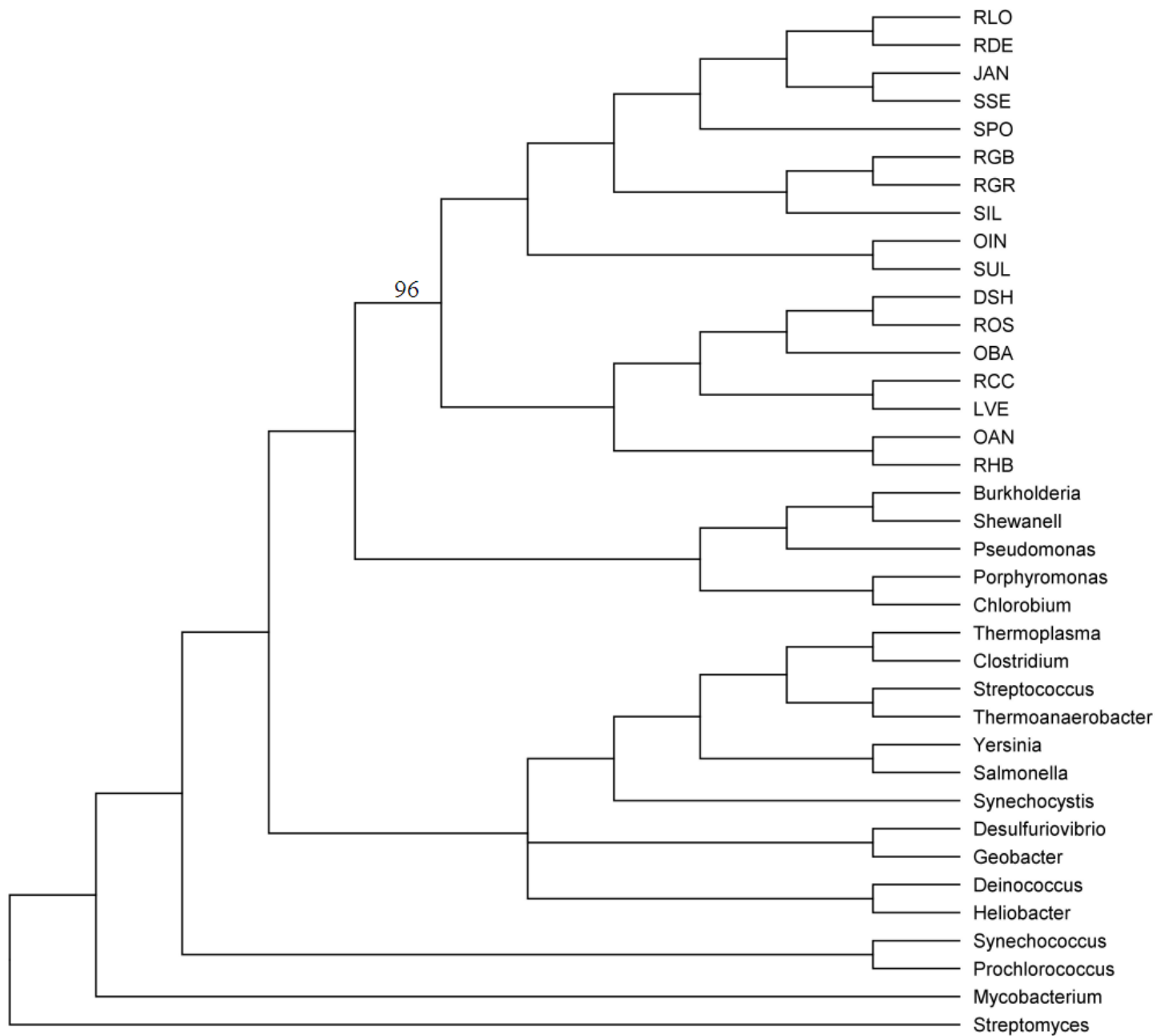

Supplement: File S2 — Tree topologies with the extended data. The multi-documents have been combined into a single ZIP-formatted file. The trees should be considered unrooted. The tree topologies were calculated in PhyML as described in Methods. Numbers refer to bootstrap values. The tree topology (separate pdf) shows that Roseobacter bacteria form a monophyletic group and was deposited in a document named “high bootstrap”. The other organisms embedded within the Roseobacter clade, or Roseobacter bacteria embedded within other phyla are shown in red (deposited in a document named “inter-phylum”). Individual file name corresponds to gene family code listed in Table S1. The non Roseobacter organism taxonomic name is detailed in the amino acid fasta of the sequences (a document named “sequences”). (4.62 MB ZIP) [file pone.0011604.s008.zip › high bootstrap/ort594.pdf]

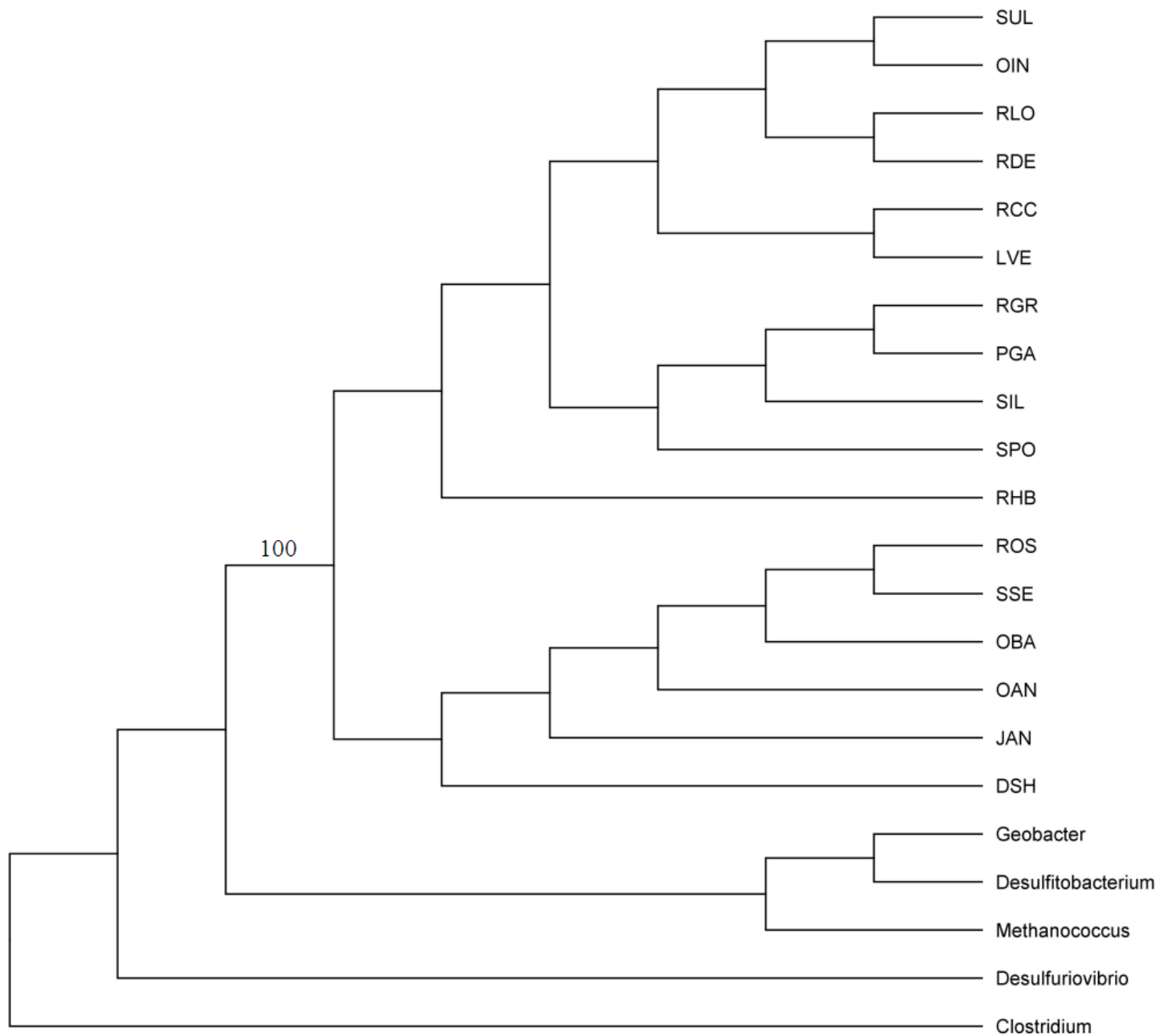

Supplement: File S2 — Tree topologies with the extended data. The multi-documents have been combined into a single ZIP-formatted file. The trees should be considered unrooted. The tree topologies were calculated in PhyML as described in Methods. Numbers refer to bootstrap values. The tree topology (separate pdf) shows that Roseobacter bacteria form a monophyletic group and was deposited in a document named “high bootstrap”. The other organisms embedded within the Roseobacter clade, or Roseobacter bacteria embedded within other phyla are shown in red (deposited in a document named “inter-phylum”). Individual file name corresponds to gene family code listed in Table S1. The non Roseobacter organism taxonomic name is detailed in the amino acid fasta of the sequences (a document named “sequences”). (4.62 MB ZIP) [file pone.0011604.s008.zip › high bootstrap/ort601.pdf]

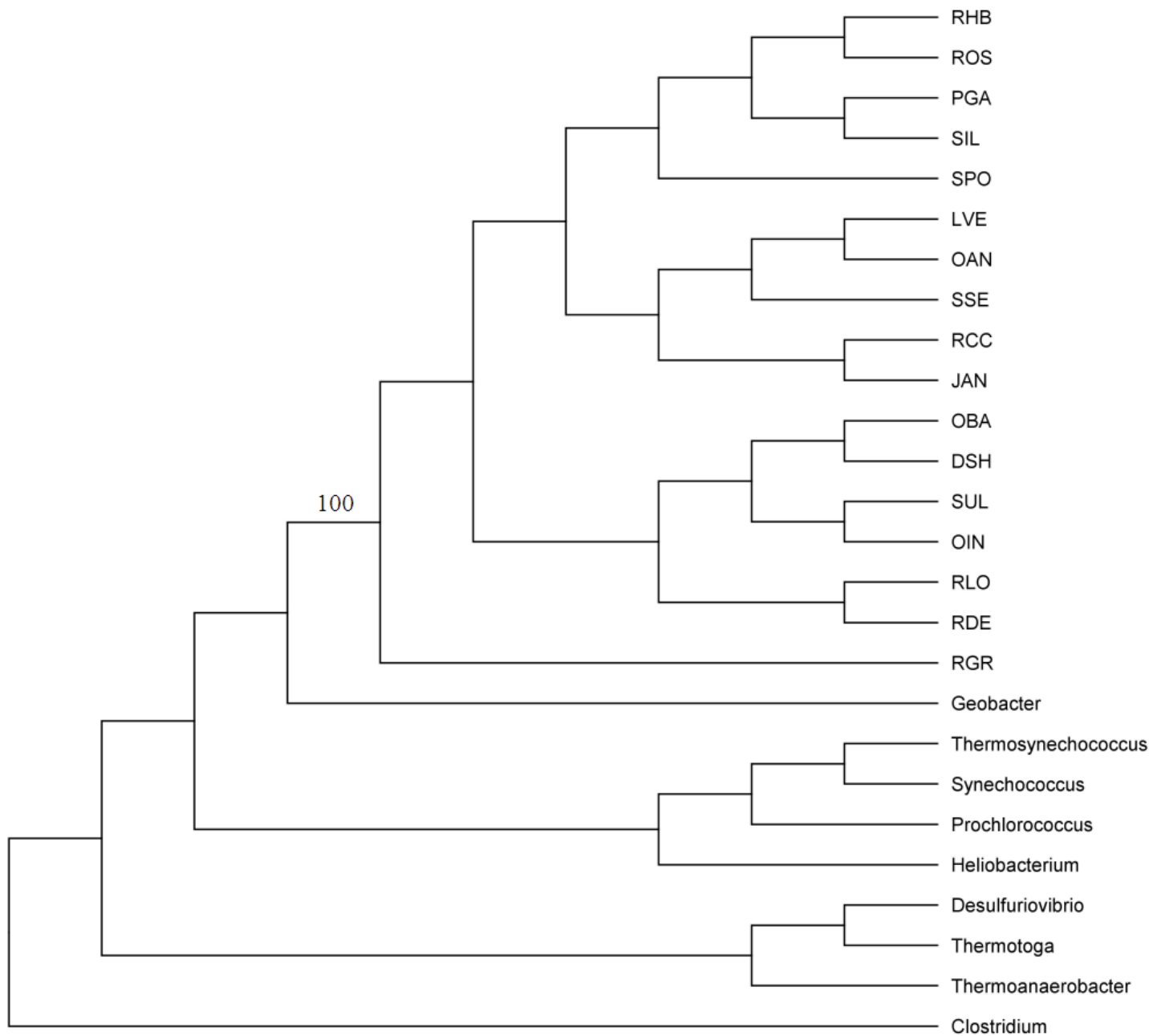

Supplement: File S2 — Tree topologies with the extended data. The multi-documents have been combined into a single ZIP-formatted file. The trees should be considered unrooted. The tree topologies were calculated in PhyML as described in Methods. Numbers refer to bootstrap values. The tree topology (separate pdf) shows that Roseobacter bacteria form a monophyletic group and was deposited in a document named “high bootstrap”. The other organisms embedded within the Roseobacter clade, or Roseobacter bacteria embedded within other phyla are shown in red (deposited in a document named “inter-phylum”). Individual file name corresponds to gene family code listed in Table S1. The non Roseobacter organism taxonomic name is detailed in the amino acid fasta of the sequences (a document named “sequences”). (4.62 MB ZIP) [file pone.0011604.s008.zip › high bootstrap/ort602.pdf]

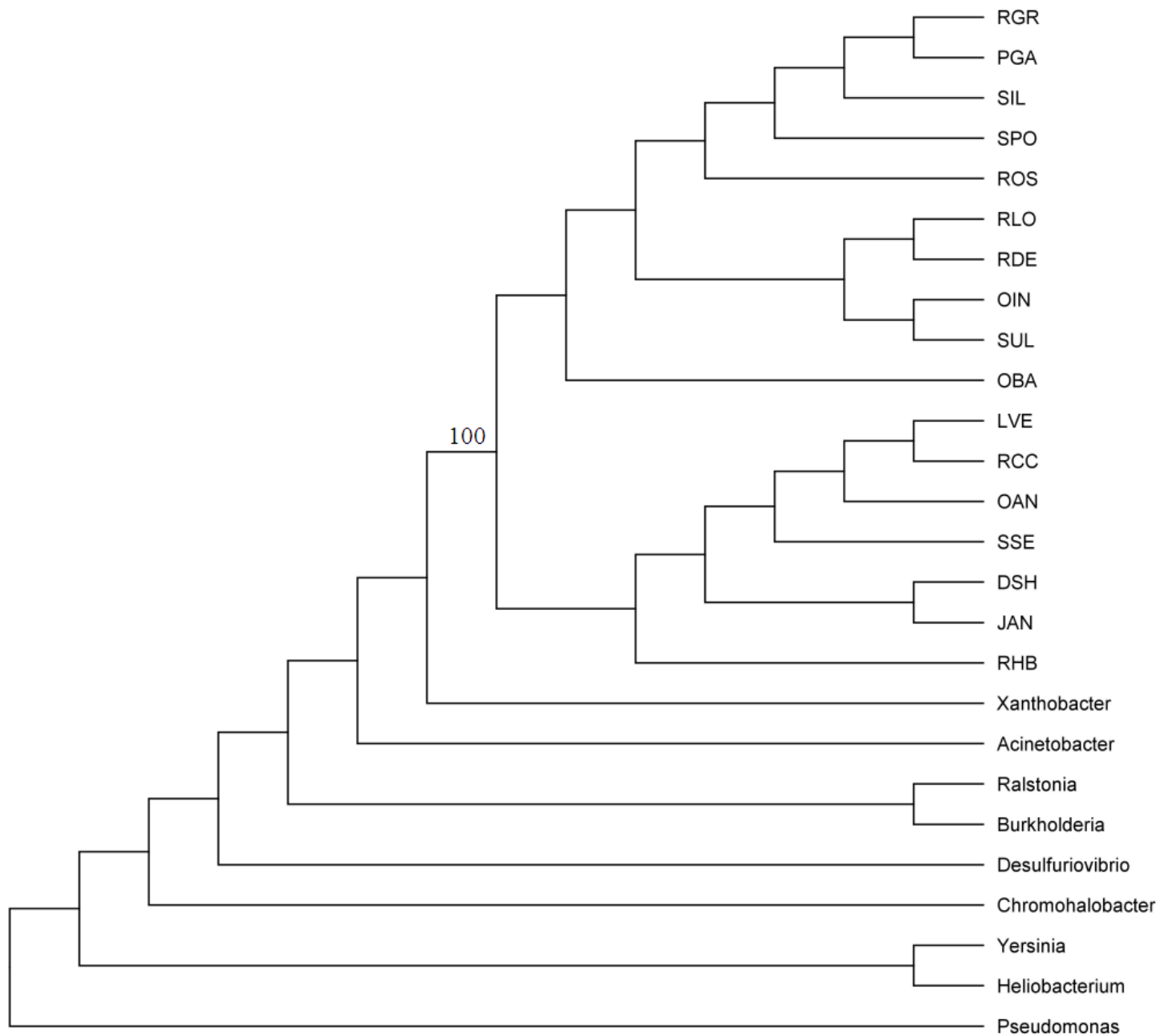

Supplement: File S2 — Tree topologies with the extended data. The multi-documents have been combined into a single ZIP-formatted file. The trees should be considered unrooted. The tree topologies were calculated in PhyML as described in Methods. Numbers refer to bootstrap values. The tree topology (separate pdf) shows that Roseobacter bacteria form a monophyletic group and was deposited in a document named “high bootstrap”. The other organisms embedded within the Roseobacter clade, or Roseobacter bacteria embedded within other phyla are shown in red (deposited in a document named “inter-phylum”). Individual file name corresponds to gene family code listed in Table S1. The non Roseobacter organism taxonomic name is detailed in the amino acid fasta of the sequences (a document named “sequences”). (4.62 MB ZIP) [file pone.0011604.s008.zip › high bootstrap/ort665.pdf]

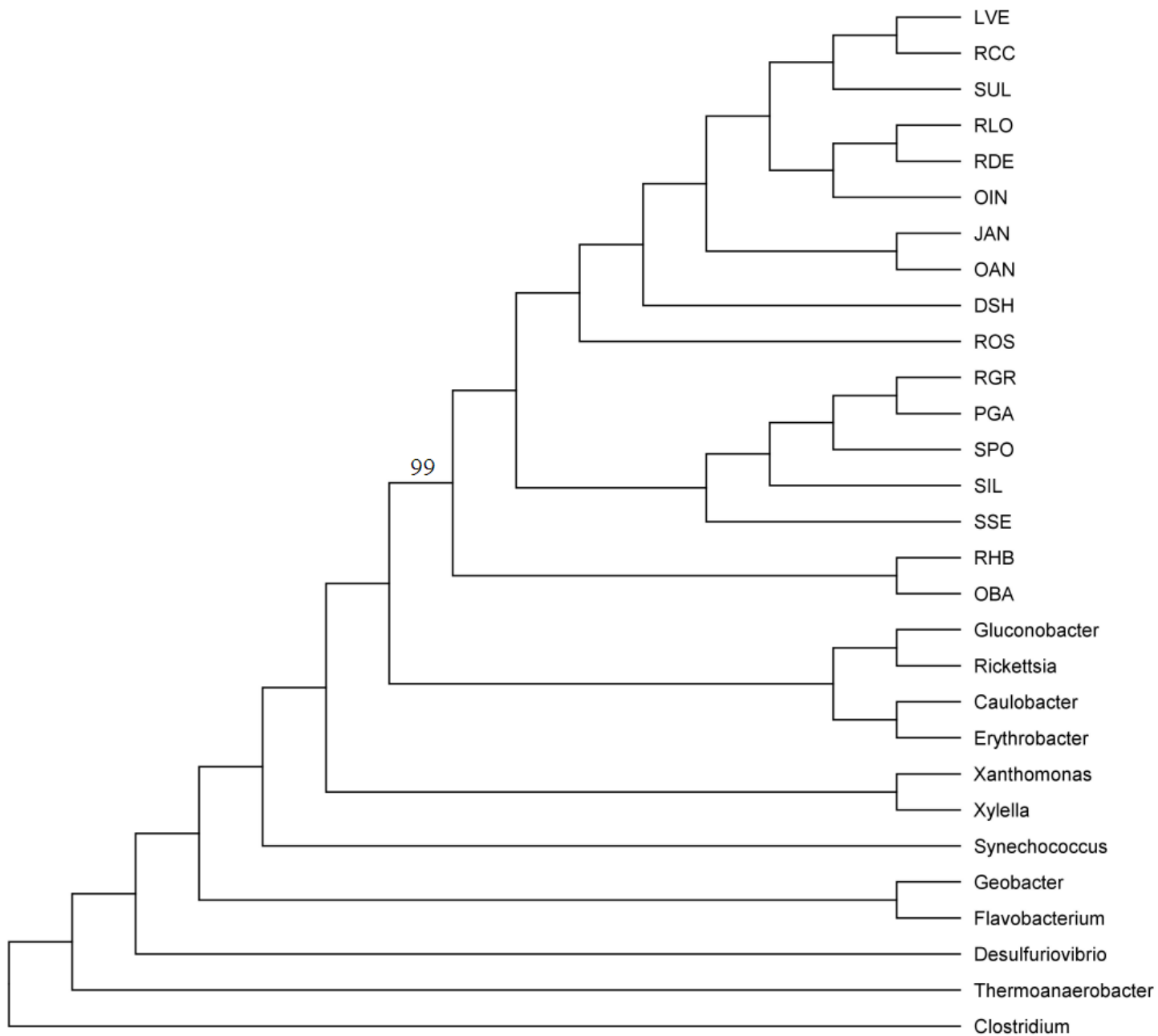

Supplement: File S2 — Tree topologies with the extended data. The multi-documents have been combined into a single ZIP-formatted file. The trees should be considered unrooted. The tree topologies were calculated in PhyML as described in Methods. Numbers refer to bootstrap values. The tree topology (separate pdf) shows that Roseobacter bacteria form a monophyletic group and was deposited in a document named “high bootstrap”. The other organisms embedded within the Roseobacter clade, or Roseobacter bacteria embedded within other phyla are shown in red (deposited in a document named “inter-phylum”). Individual file name corresponds to gene family code listed in Table S1. The non Roseobacter organism taxonomic name is detailed in the amino acid fasta of the sequences (a document named “sequences”). (4.62 MB ZIP) [file pone.0011604.s008.zip › high bootstrap/ort697.pdf]

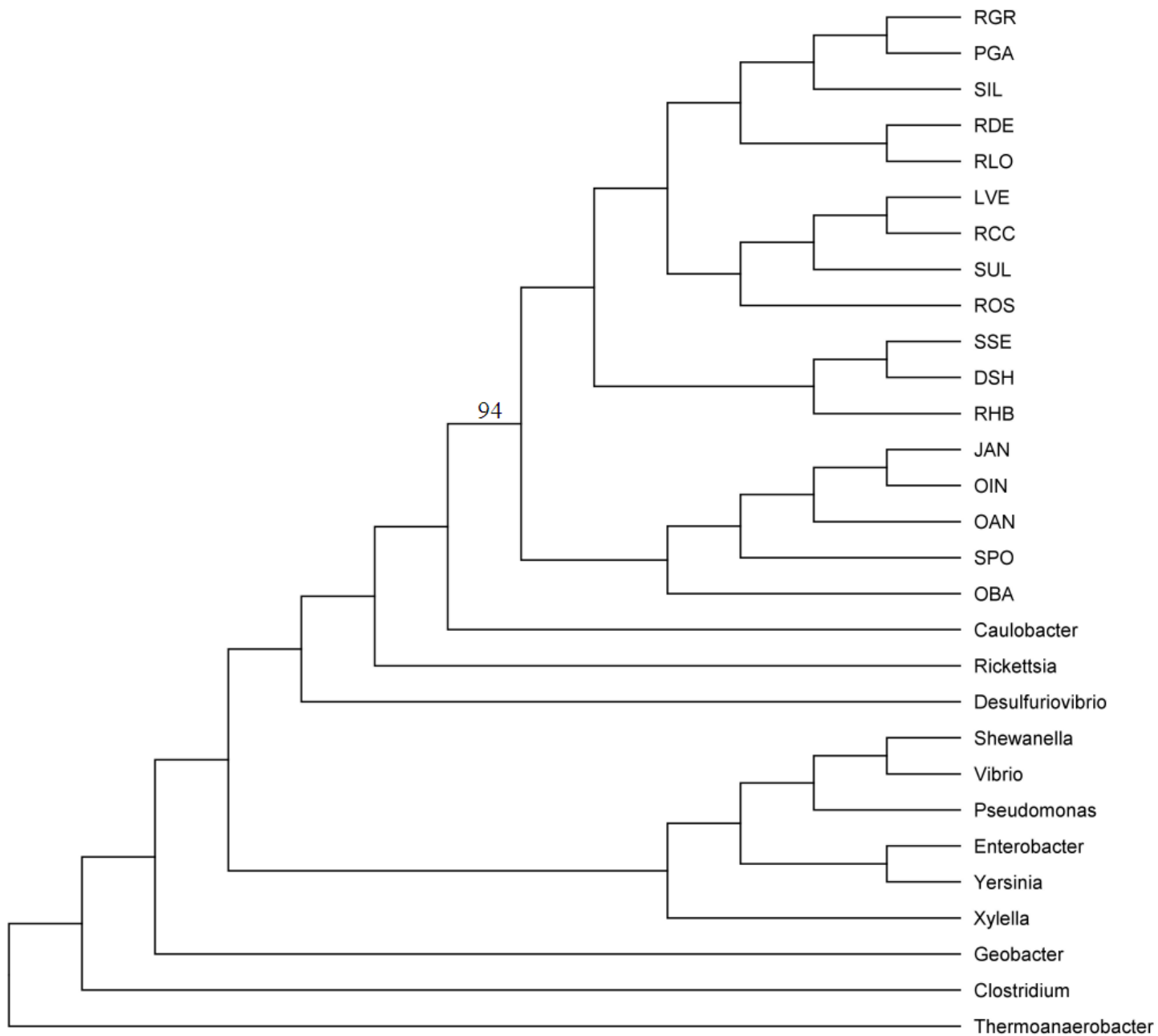

Supplement: File S2 — Tree topologies with the extended data. The multi-documents have been combined into a single ZIP-formatted file. The trees should be considered unrooted. The tree topologies were calculated in PhyML as described in Methods. Numbers refer to bootstrap values. The tree topology (separate pdf) shows that Roseobacter bacteria form a monophyletic group and was deposited in a document named “high bootstrap”. The other organisms embedded within the Roseobacter clade, or Roseobacter bacteria embedded within other phyla are shown in red (deposited in a document named “inter-phylum”). Individual file name corresponds to gene family code listed in Table S1. The non Roseobacter organism taxonomic name is detailed in the amino acid fasta of the sequences (a document named “sequences”). (4.62 MB ZIP) [file pone.0011604.s008.zip › high bootstrap/ort720.pdf]

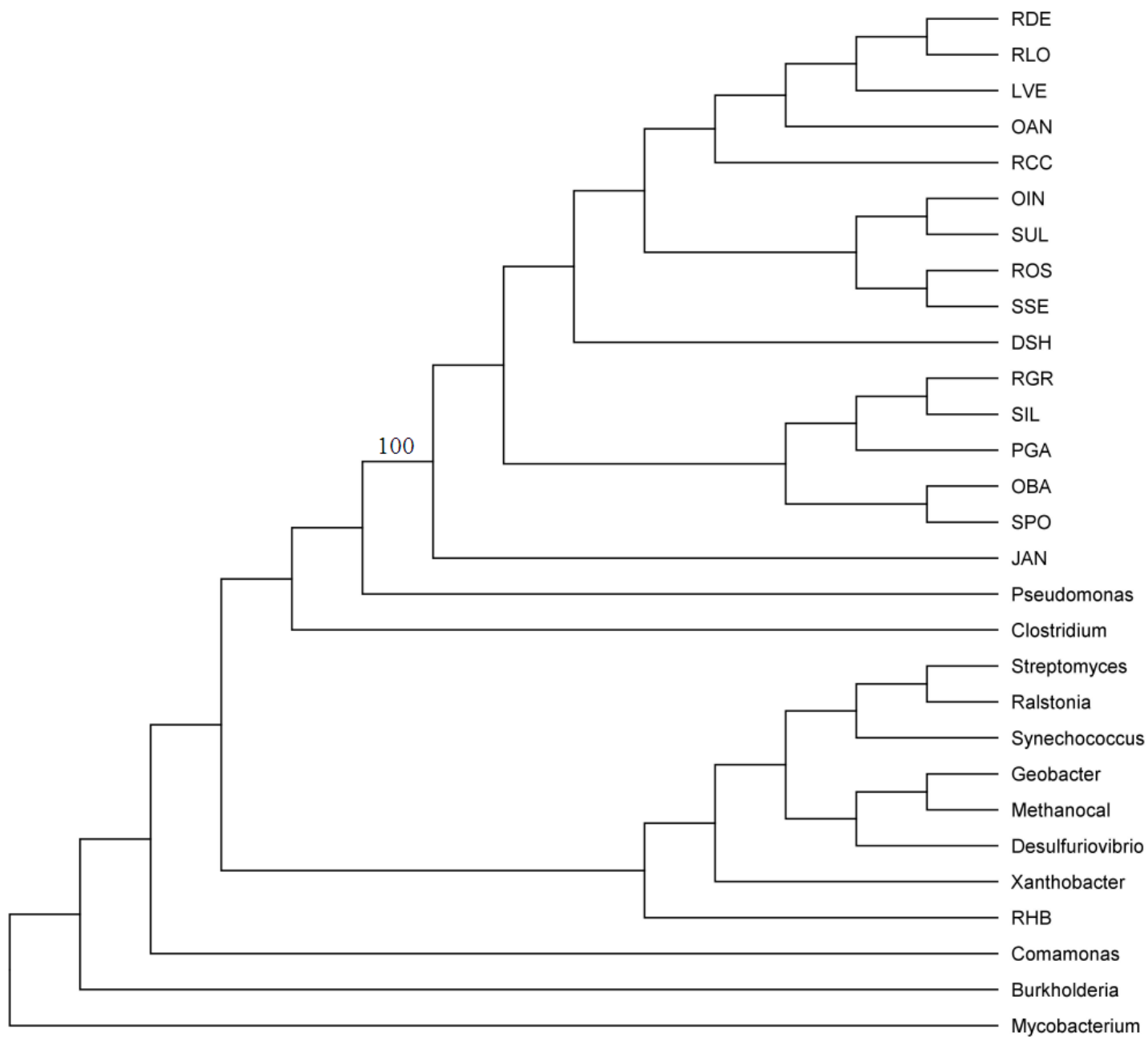

Supplement: File S2 — Tree topologies with the extended data. The multi-documents have been combined into a single ZIP-formatted file. The trees should be considered unrooted. The tree topologies were calculated in PhyML as described in Methods. Numbers refer to bootstrap values. The tree topology (separate pdf) shows that Roseobacter bacteria form a monophyletic group and was deposited in a document named “high bootstrap”. The other organisms embedded within the Roseobacter clade, or Roseobacter bacteria embedded within other phyla are shown in red (deposited in a document named “inter-phylum”). Individual file name corresponds to gene family code listed in Table S1. The non Roseobacter organism taxonomic name is detailed in the amino acid fasta of the sequences (a document named “sequences”). (4.62 MB ZIP) [file pone.0011604.s008.zip › high bootstrap/ort725.pdf]

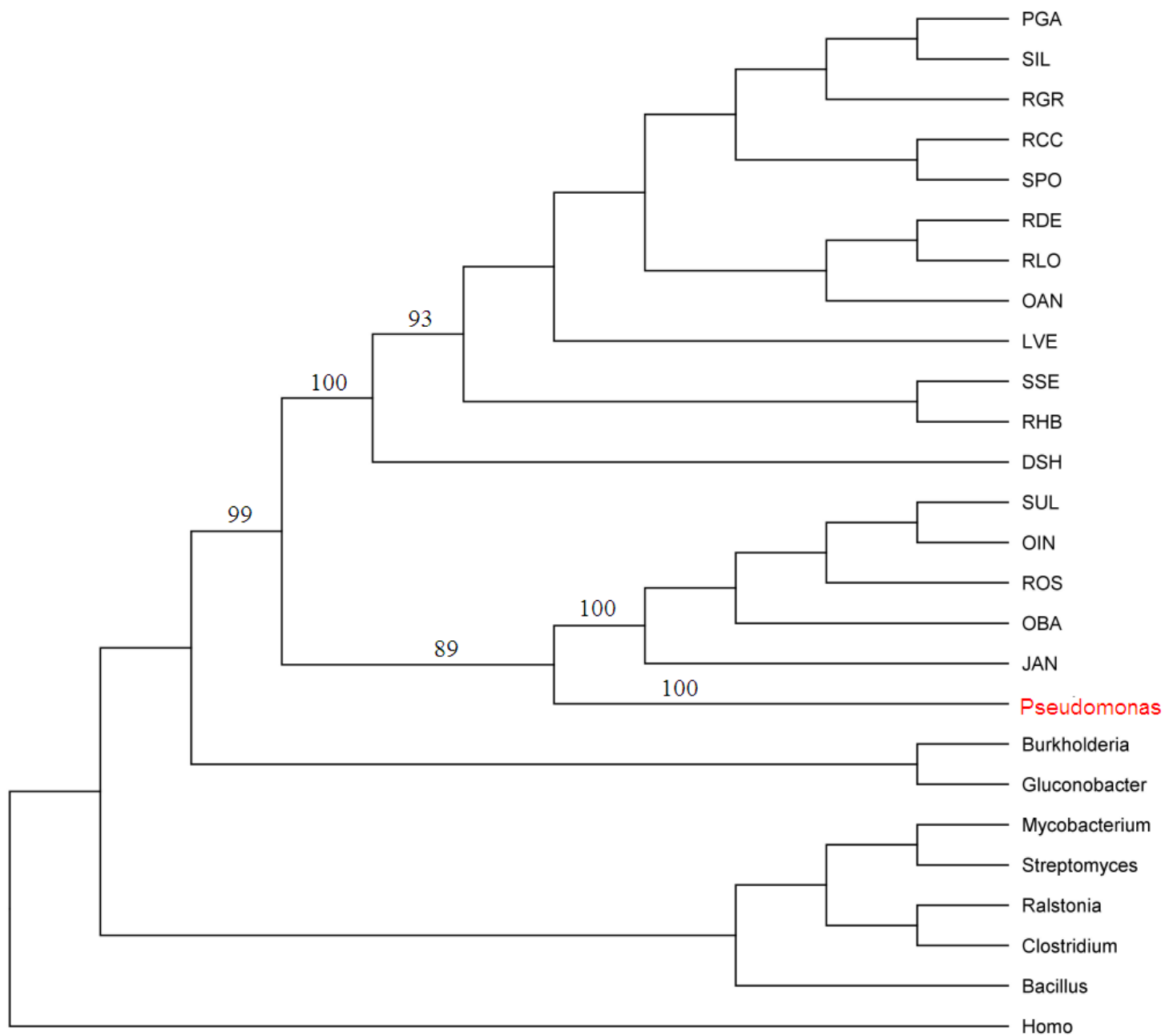

Supplement: File S2 — Tree topologies with the extended data. The multi-documents have been combined into a single ZIP-formatted file. The trees should be considered unrooted. The tree topologies were calculated in PhyML as described in Methods. Numbers refer to bootstrap values. The tree topology (separate pdf) shows that Roseobacter bacteria form a monophyletic group and was deposited in a document named “high bootstrap”. The other organisms embedded within the Roseobacter clade, or Roseobacter bacteria embedded within other phyla are shown in red (deposited in a document named “inter-phylum”). Individual file name corresponds to gene family code listed in Table S1. The non Roseobacter organism taxonomic name is detailed in the amino acid fasta of the sequences (a document named “sequences”). (4.62 MB ZIP) [file pone.0011604.s008.zip › high bootstrap/ort726.pdf]

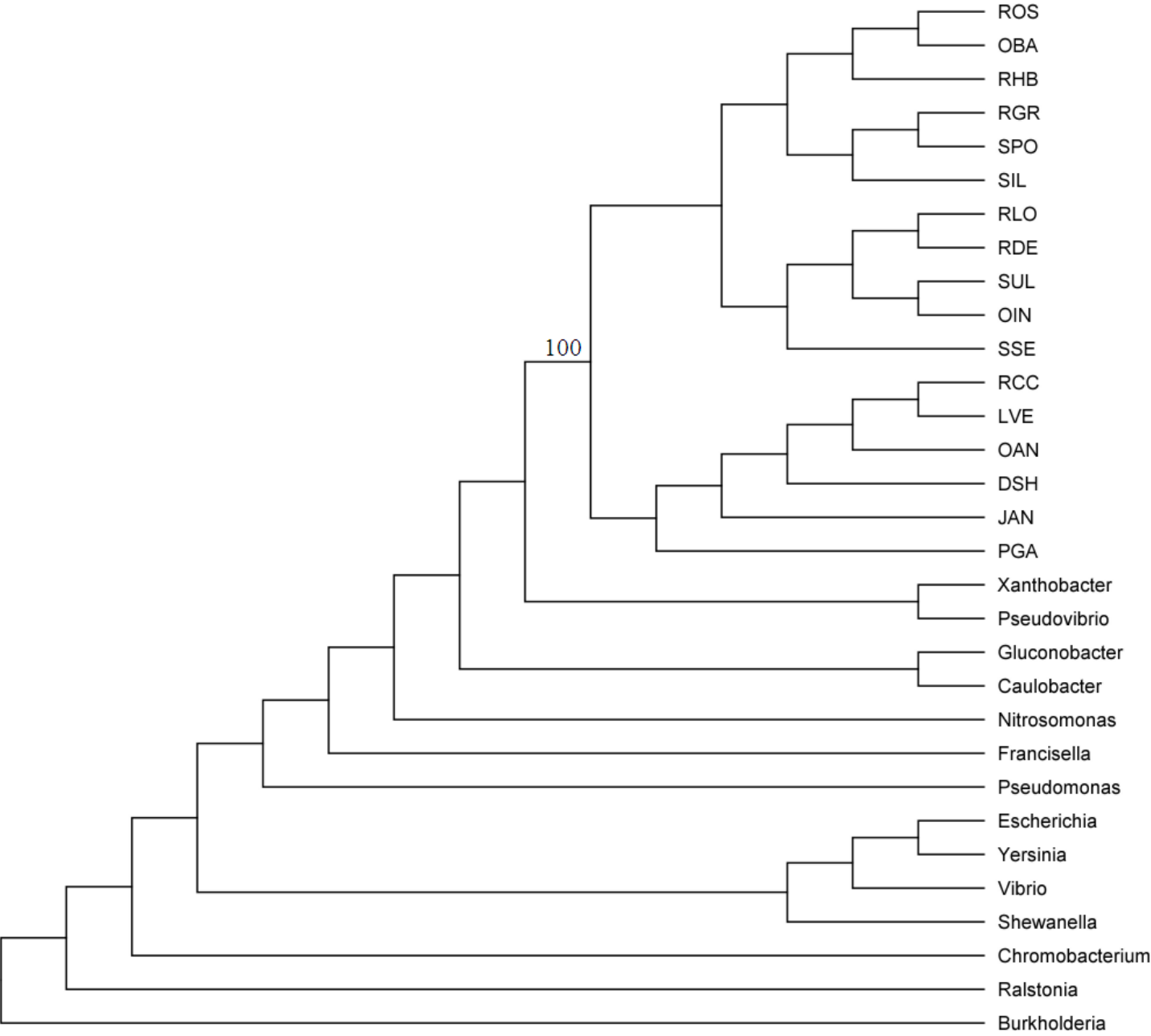

Supplement: File S2 — Tree topologies with the extended data. The multi-documents have been combined into a single ZIP-formatted file. The trees should be considered unrooted. The tree topologies were calculated in PhyML as described in Methods. Numbers refer to bootstrap values. The tree topology (separate pdf) shows that Roseobacter bacteria form a monophyletic group and was deposited in a document named “high bootstrap”. The other organisms embedded within the Roseobacter clade, or Roseobacter bacteria embedded within other phyla are shown in red (deposited in a document named “inter-phylum”). Individual file name corresponds to gene family code listed in Table S1. The non Roseobacter organism taxonomic name is detailed in the amino acid fasta of the sequences (a document named “sequences”). (4.62 MB ZIP) [file pone.0011604.s008.zip › high bootstrap/ort732.pdf]

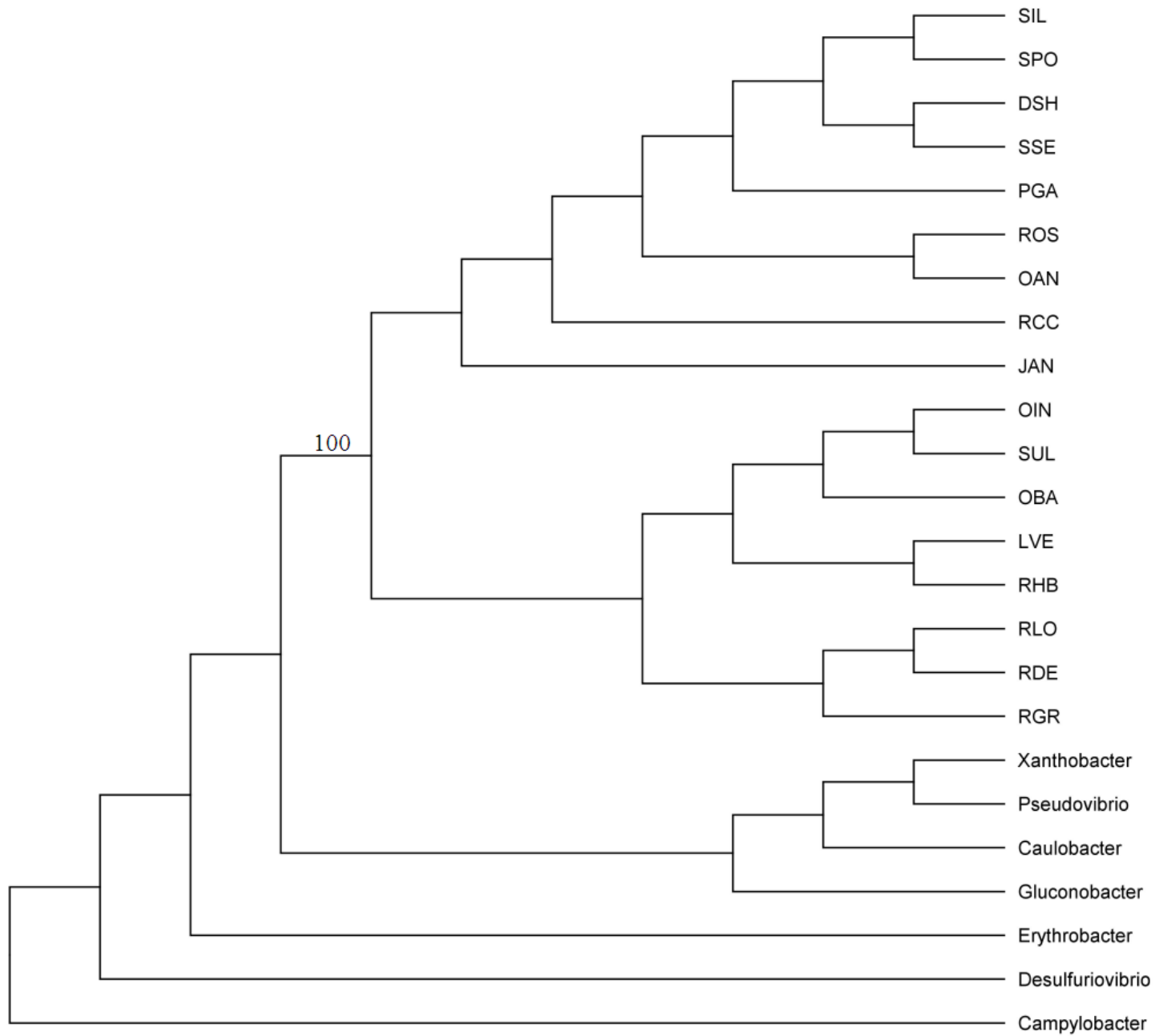

Supplement: File S2 — Tree topologies with the extended data. The multi-documents have been combined into a single ZIP-formatted file. The trees should be considered unrooted. The tree topologies were calculated in PhyML as described in Methods. Numbers refer to bootstrap values. The tree topology (separate pdf) shows that Roseobacter bacteria form a monophyletic group and was deposited in a document named “high bootstrap”. The other organisms embedded within the Roseobacter clade, or Roseobacter bacteria embedded within other phyla are shown in red (deposited in a document named “inter-phylum”). Individual file name corresponds to gene family code listed in Table S1. The non Roseobacter organism taxonomic name is detailed in the amino acid fasta of the sequences (a document named “sequences”). (4.62 MB ZIP) [file pone.0011604.s008.zip › high bootstrap/ort736.pdf]

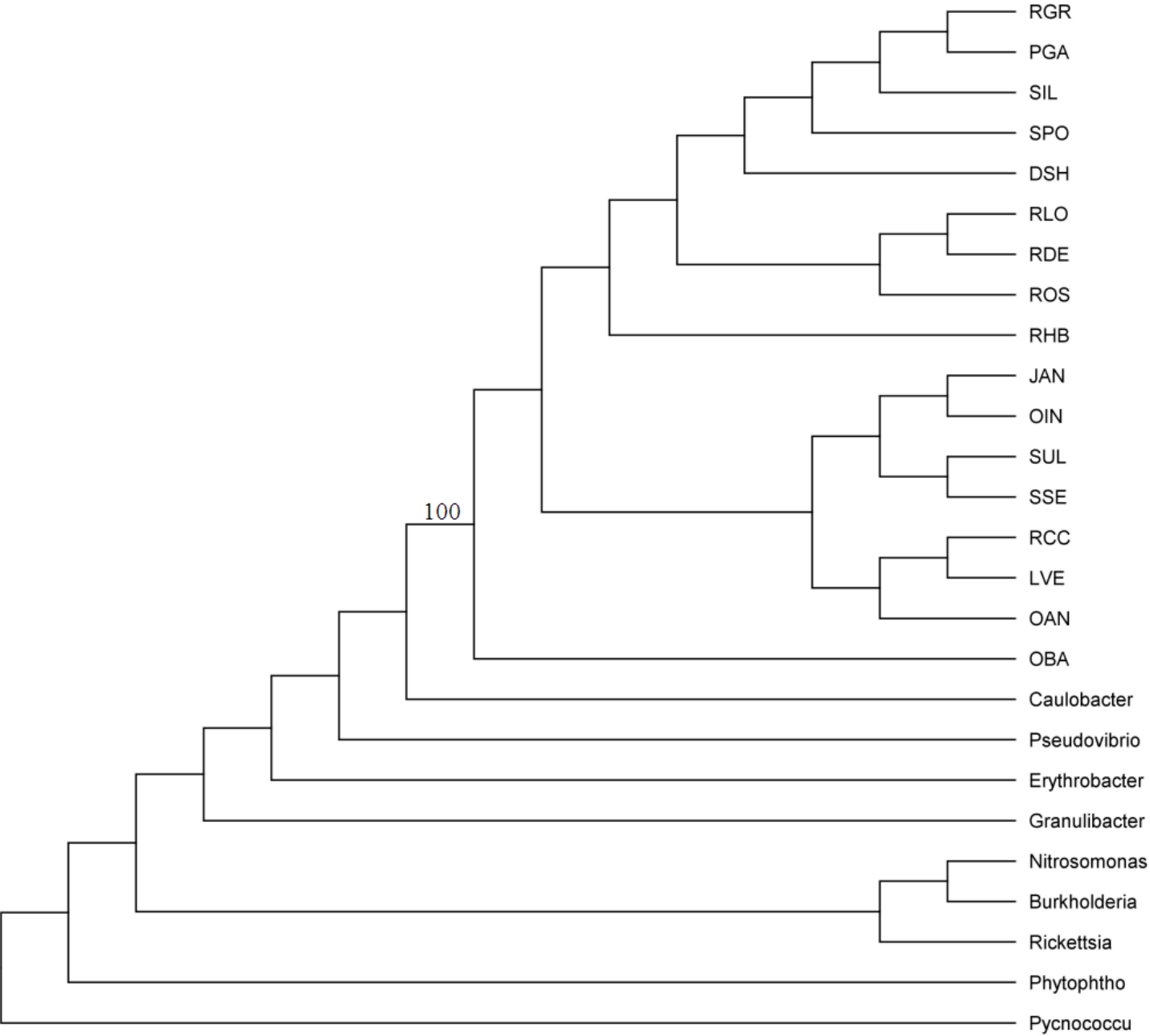

Supplement: File S2 — Tree topologies with the extended data. The multi-documents have been combined into a single ZIP-formatted file. The trees should be considered unrooted. The tree topologies were calculated in PhyML as described in Methods. Numbers refer to bootstrap values. The tree topology (separate pdf) shows that Roseobacter bacteria form a monophyletic group and was deposited in a document named “high bootstrap”. The other organisms embedded within the Roseobacter clade, or Roseobacter bacteria embedded within other phyla are shown in red (deposited in a document named “inter-phylum”). Individual file name corresponds to gene family code listed in Table S1. The non Roseobacter organism taxonomic name is detailed in the amino acid fasta of the sequences (a document named “sequences”). (4.62 MB ZIP) [file pone.0011604.s008.zip › high bootstrap/ort752.pdf]

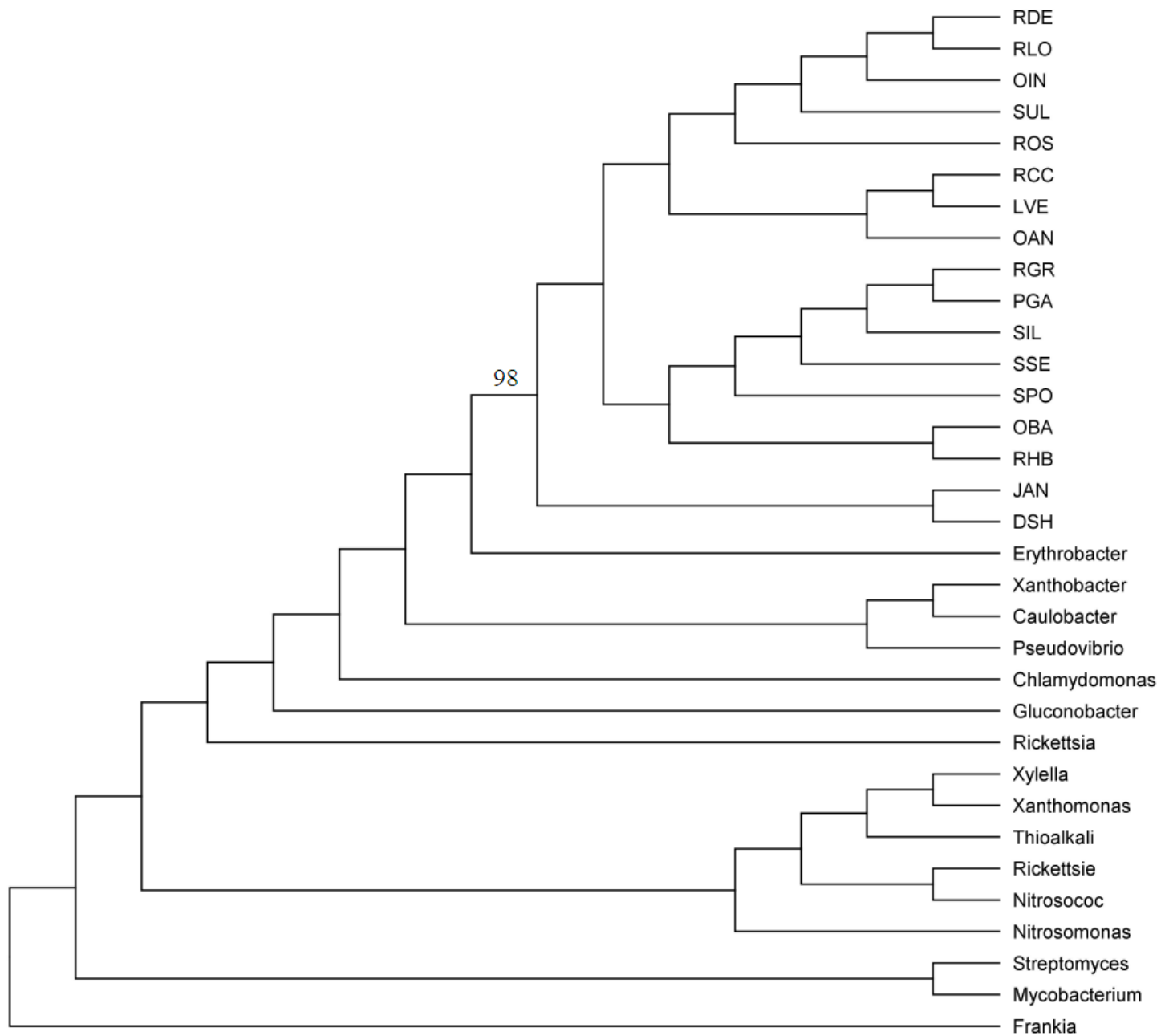

Supplement: File S2 — Tree topologies with the extended data. The multi-documents have been combined into a single ZIP-formatted file. The trees should be considered unrooted. The tree topologies were calculated in PhyML as described in Methods. Numbers refer to bootstrap values. The tree topology (separate pdf) shows that Roseobacter bacteria form a monophyletic group and was deposited in a document named “high bootstrap”. The other organisms embedded within the Roseobacter clade, or Roseobacter bacteria embedded within other phyla are shown in red (deposited in a document named “inter-phylum”). Individual file name corresponds to gene family code listed in Table S1. The non Roseobacter organism taxonomic name is detailed in the amino acid fasta of the sequences (a document named “sequences”). (4.62 MB ZIP) [file pone.0011604.s008.zip › high bootstrap/ort754.pdf]

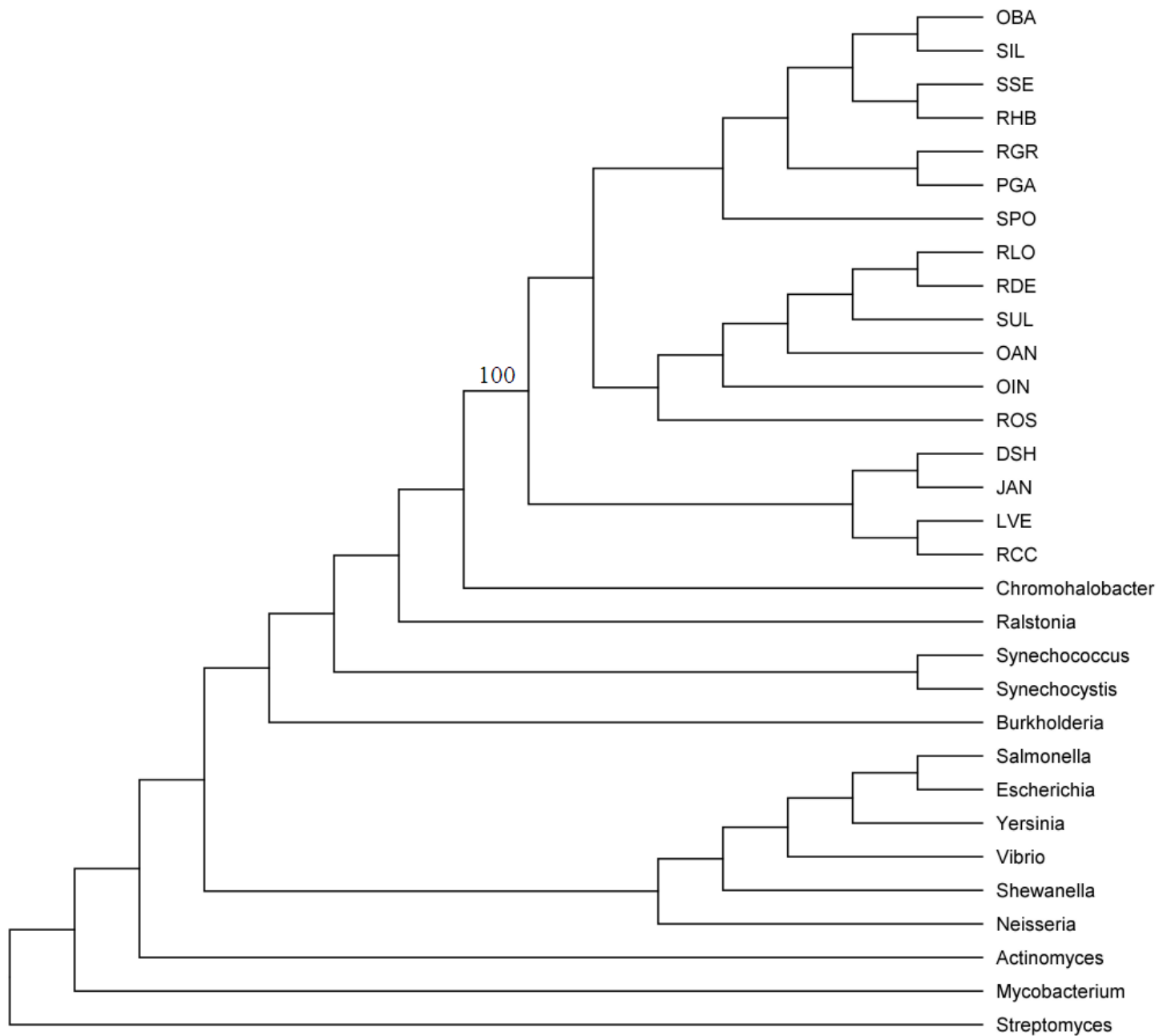

Supplement: File S2 — Tree topologies with the extended data. The multi-documents have been combined into a single ZIP-formatted file. The trees should be considered unrooted. The tree topologies were calculated in PhyML as described in Methods. Numbers refer to bootstrap values. The tree topology (separate pdf) shows that Roseobacter bacteria form a monophyletic group and was deposited in a document named “high bootstrap”. The other organisms embedded within the Roseobacter clade, or Roseobacter bacteria embedded within other phyla are shown in red (deposited in a document named “inter-phylum”). Individual file name corresponds to gene family code listed in Table S1. The non Roseobacter organism taxonomic name is detailed in the amino acid fasta of the sequences (a document named “sequences”). (4.62 MB ZIP) [file pone.0011604.s008.zip › high bootstrap/ort770.pdf]

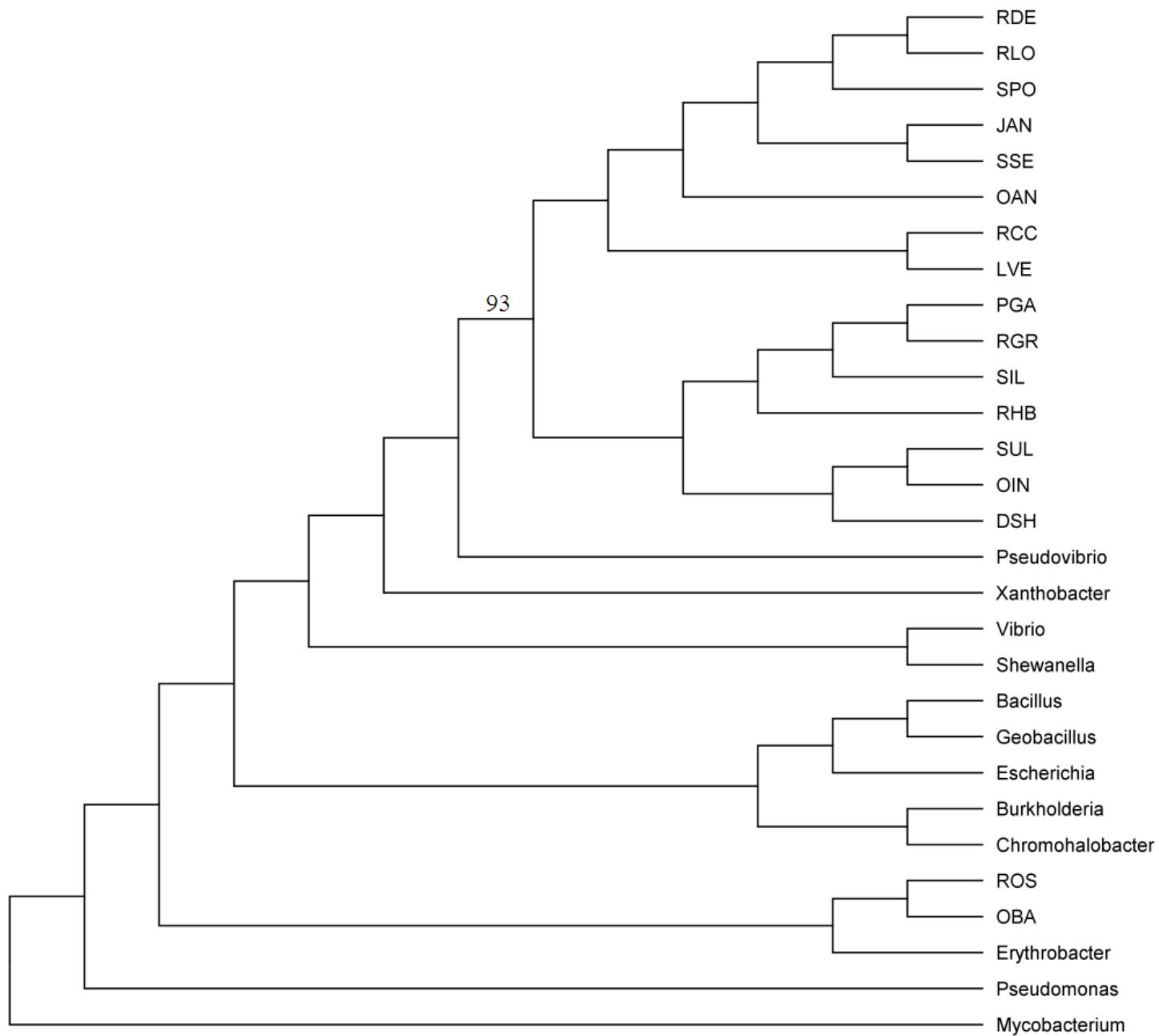

Supplement: File S2 — Tree topologies with the extended data. The multi-documents have been combined into a single ZIP-formatted file. The trees should be considered unrooted. The tree topologies were calculated in PhyML as described in Methods. Numbers refer to bootstrap values. The tree topology (separate pdf) shows that Roseobacter bacteria form a monophyletic group and was deposited in a document named “high bootstrap”. The other organisms embedded within the Roseobacter clade, or Roseobacter bacteria embedded within other phyla are shown in red (deposited in a document named “inter-phylum”). Individual file name corresponds to gene family code listed in Table S1. The non Roseobacter organism taxonomic name is detailed in the amino acid fasta of the sequences (a document named “sequences”). (4.62 MB ZIP) [file pone.0011604.s008.zip › high bootstrap/ort772.pdf]

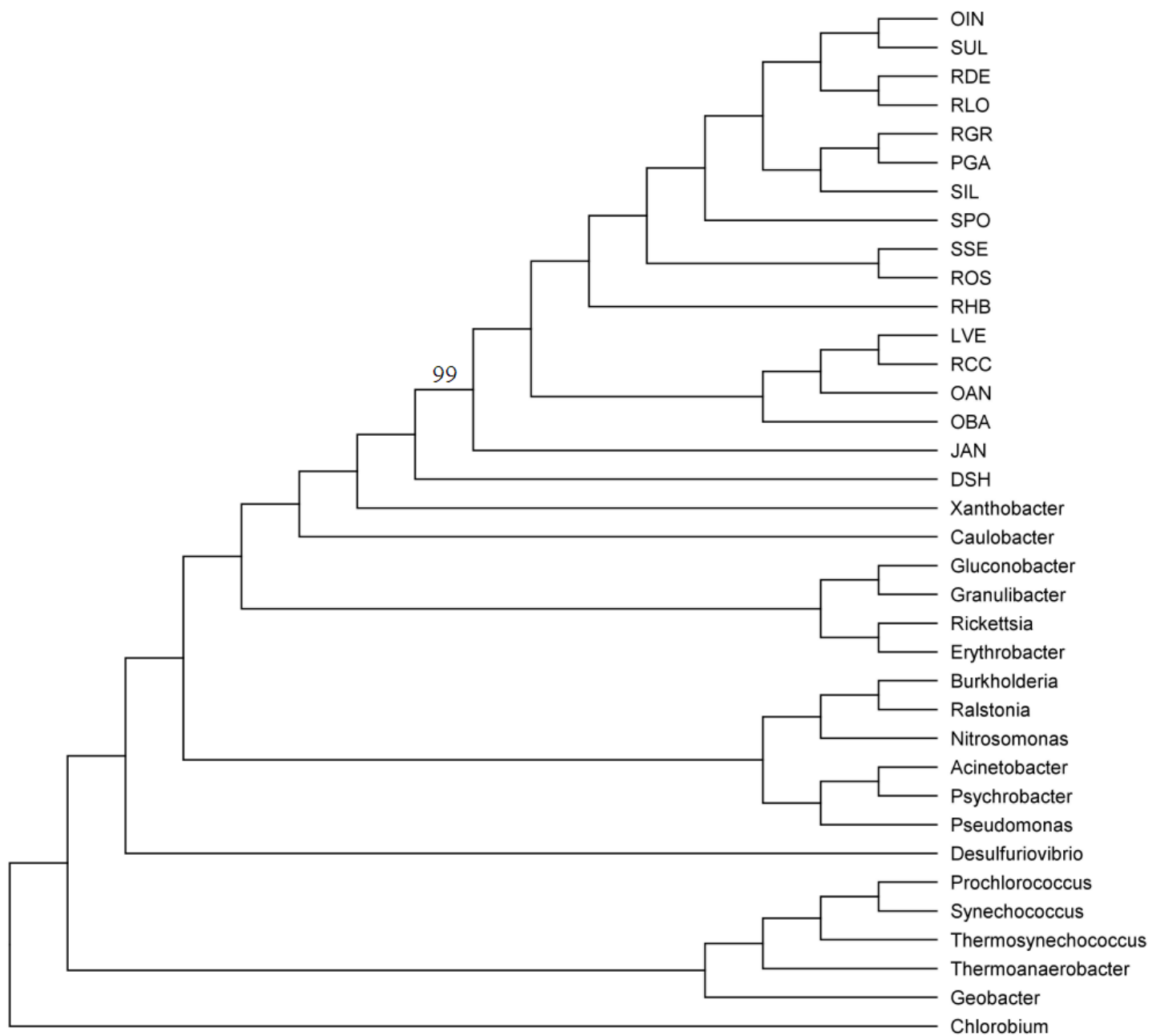

Supplement: File S2 — Tree topologies with the extended data. The multi-documents have been combined into a single ZIP-formatted file. The trees should be considered unrooted. The tree topologies were calculated in PhyML as described in Methods. Numbers refer to bootstrap values. The tree topology (separate pdf) shows that Roseobacter bacteria form a monophyletic group and was deposited in a document named “high bootstrap”. The other organisms embedded within the Roseobacter clade, or Roseobacter bacteria embedded within other phyla are shown in red (deposited in a document named “inter-phylum”). Individual file name corresponds to gene family code listed in Table S1. The non Roseobacter organism taxonomic name is detailed in the amino acid fasta of the sequences (a document named “sequences”). (4.62 MB ZIP) [file pone.0011604.s008.zip › high bootstrap/ort773.pdf]

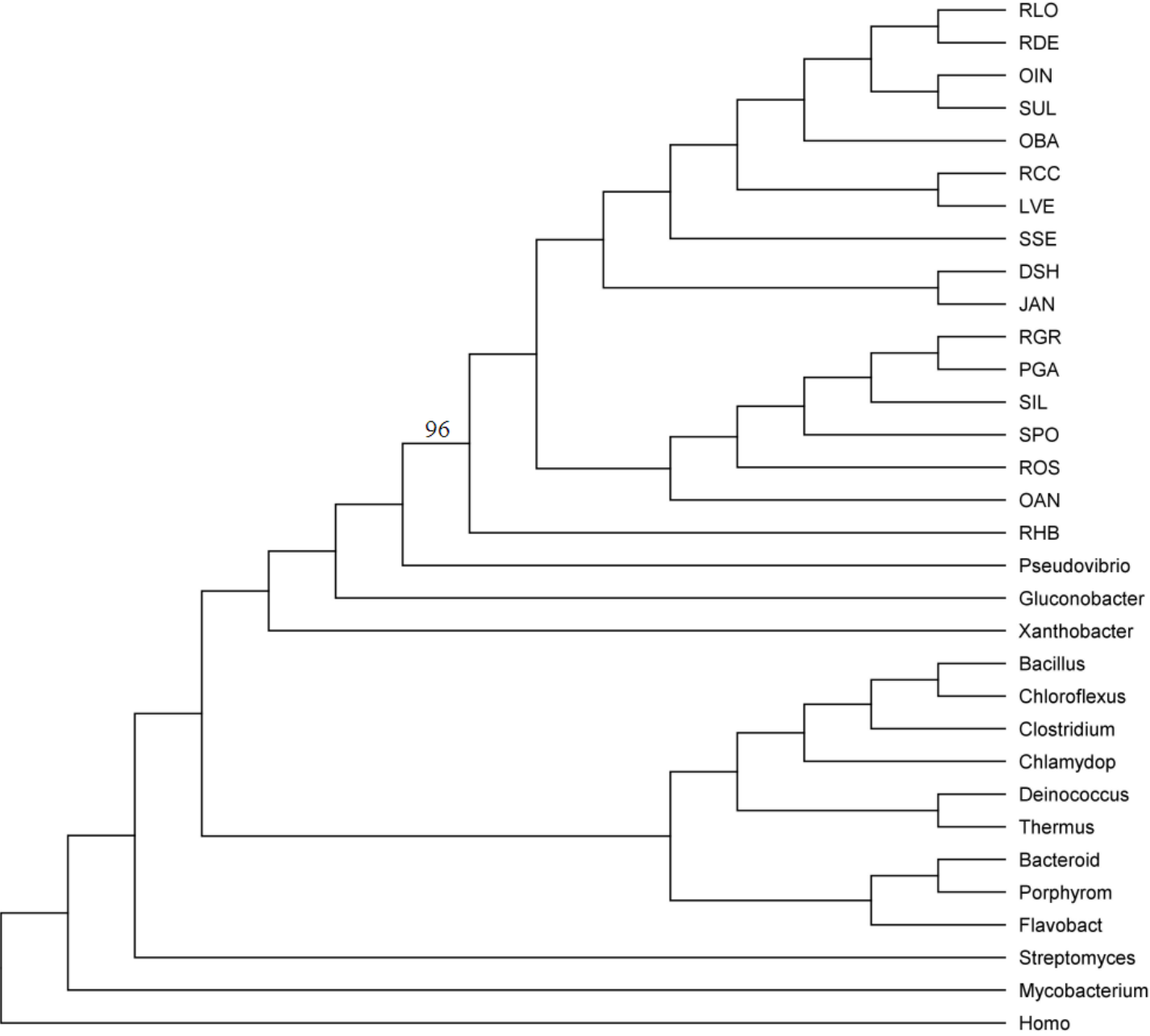

Supplement: File S2 — Tree topologies with the extended data. The multi-documents have been combined into a single ZIP-formatted file. The trees should be considered unrooted. The tree topologies were calculated in PhyML as described in Methods. Numbers refer to bootstrap values. The tree topology (separate pdf) shows that Roseobacter bacteria form a monophyletic group and was deposited in a document named “high bootstrap”. The other organisms embedded within the Roseobacter clade, or Roseobacter bacteria embedded within other phyla are shown in red (deposited in a document named “inter-phylum”). Individual file name corresponds to gene family code listed in Table S1. The non Roseobacter organism taxonomic name is detailed in the amino acid fasta of the sequences (a document named “sequences”). (4.62 MB ZIP) [file pone.0011604.s008.zip › high bootstrap/ort784.pdf]

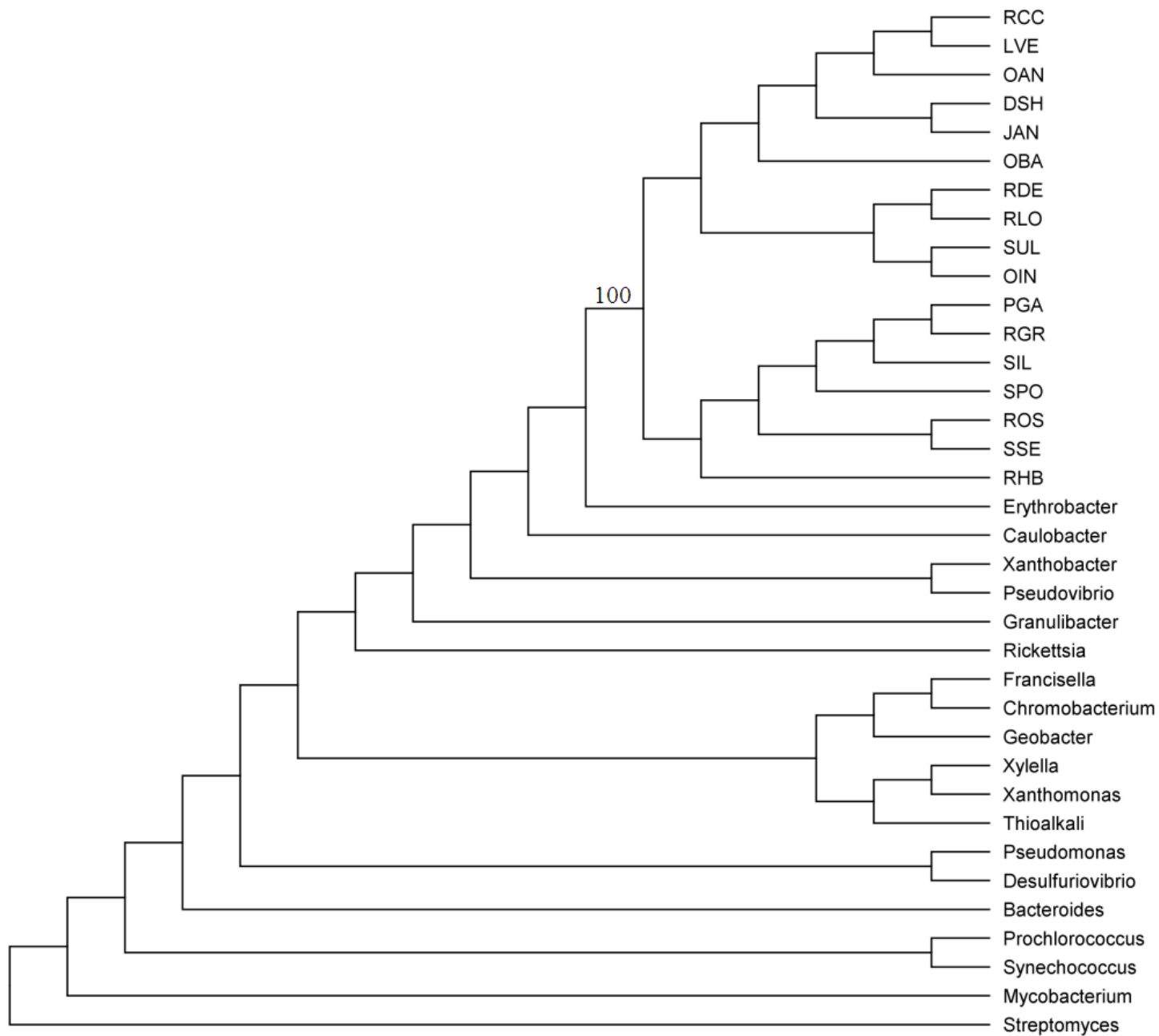

Supplement: File S2 — Tree topologies with the extended data. The multi-documents have been combined into a single ZIP-formatted file. The trees should be considered unrooted. The tree topologies were calculated in PhyML as described in Methods. Numbers refer to bootstrap values. The tree topology (separate pdf) shows that Roseobacter bacteria form a monophyletic group and was deposited in a document named “high bootstrap”. The other organisms embedded within the Roseobacter clade, or Roseobacter bacteria embedded within other phyla are shown in red (deposited in a document named “inter-phylum”). Individual file name corresponds to gene family code listed in Table S1. The non Roseobacter organism taxonomic name is detailed in the amino acid fasta of the sequences (a document named “sequences”). (4.62 MB ZIP) [file pone.0011604.s008.zip › high bootstrap/ort817.pdf]

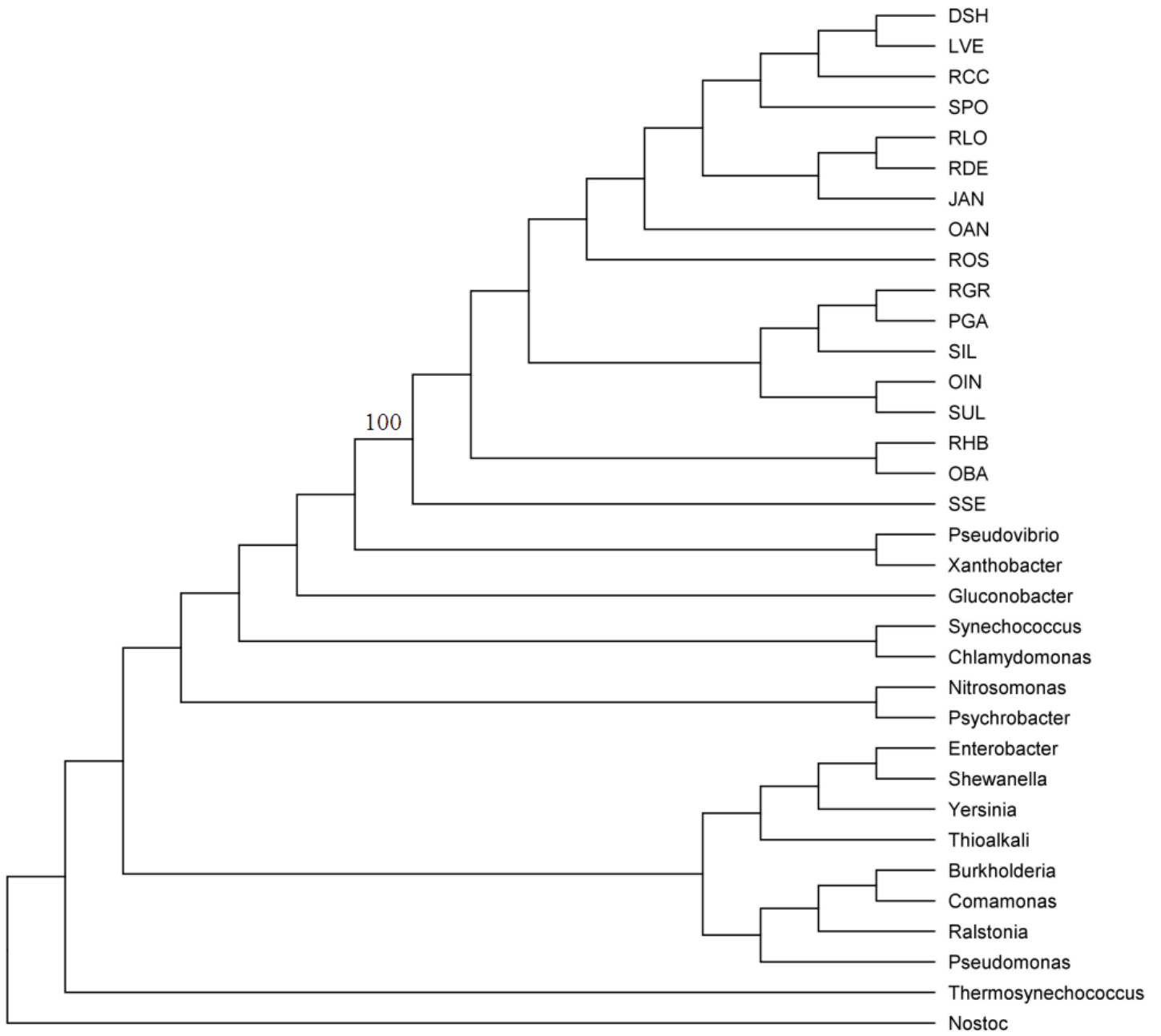

Supplement: File S2 — Tree topologies with the extended data. The multi-documents have been combined into a single ZIP-formatted file. The trees should be considered unrooted. The tree topologies were calculated in PhyML as described in Methods. Numbers refer to bootstrap values. The tree topology (separate pdf) shows that Roseobacter bacteria form a monophyletic group and was deposited in a document named “high bootstrap”. The other organisms embedded within the Roseobacter clade, or Roseobacter bacteria embedded within other phyla are shown in red (deposited in a document named “inter-phylum”). Individual file name corresponds to gene family code listed in Table S1. The non Roseobacter organism taxonomic name is detailed in the amino acid fasta of the sequences (a document named “sequences”). (4.62 MB ZIP) [file pone.0011604.s008.zip › high bootstrap/ort853.pdf]

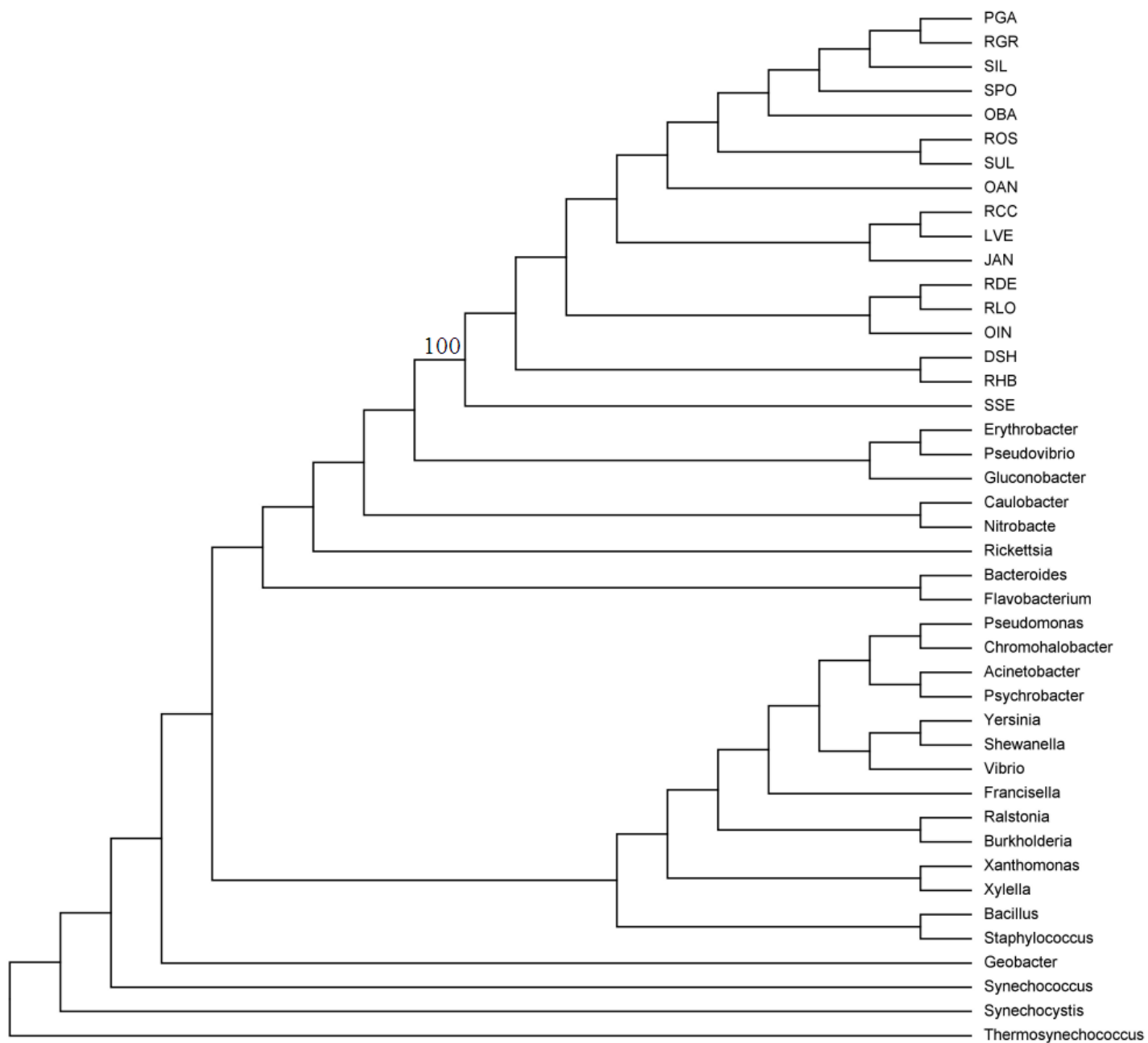

Supplement: File S2 — Tree topologies with the extended data. The multi-documents have been combined into a single ZIP-formatted file. The trees should be considered unrooted. The tree topologies were calculated in PhyML as described in Methods. Numbers refer to bootstrap values. The tree topology (separate pdf) shows that Roseobacter bacteria form a monophyletic group and was deposited in a document named “high bootstrap”. The other organisms embedded within the Roseobacter clade, or Roseobacter bacteria embedded within other phyla are shown in red (deposited in a document named “inter-phylum”). Individual file name corresponds to gene family code listed in Table S1. The non Roseobacter organism taxonomic name is detailed in the amino acid fasta of the sequences (a document named “sequences”). (4.62 MB ZIP) [file pone.0011604.s008.zip › high bootstrap/ort882.pdf]

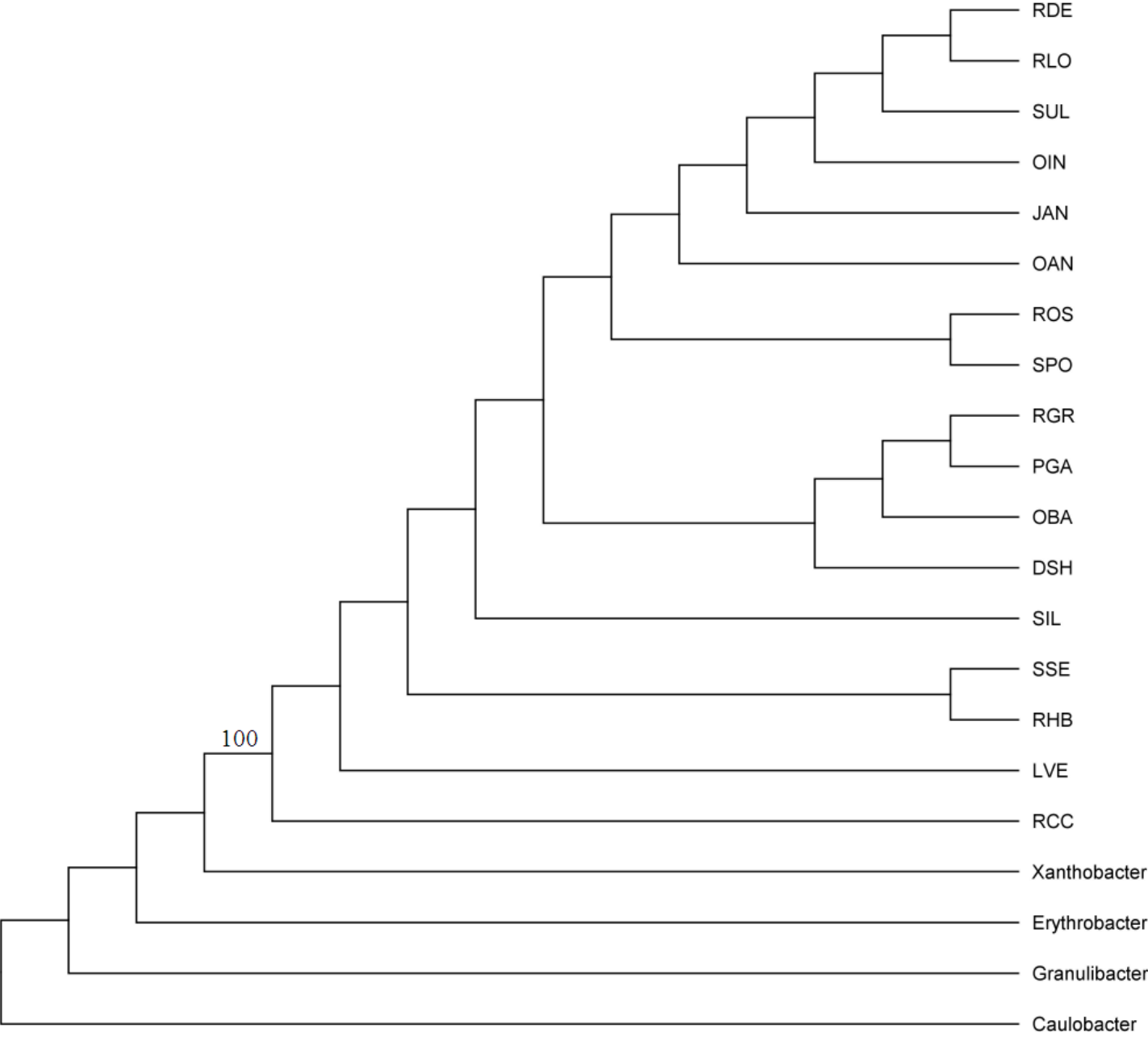

Supplement: File S2 — Tree topologies with the extended data. The multi-documents have been combined into a single ZIP-formatted file. The trees should be considered unrooted. The tree topologies were calculated in PhyML as described in Methods. Numbers refer to bootstrap values. The tree topology (separate pdf) shows that Roseobacter bacteria form a monophyletic group and was deposited in a document named “high bootstrap”. The other organisms embedded within the Roseobacter clade, or Roseobacter bacteria embedded within other phyla are shown in red (deposited in a document named “inter-phylum”). Individual file name corresponds to gene family code listed in Table S1. The non Roseobacter organism taxonomic name is detailed in the amino acid fasta of the sequences (a document named “sequences”). (4.62 MB ZIP) [file pone.0011604.s008.zip › high bootstrap/ort884.pdf]

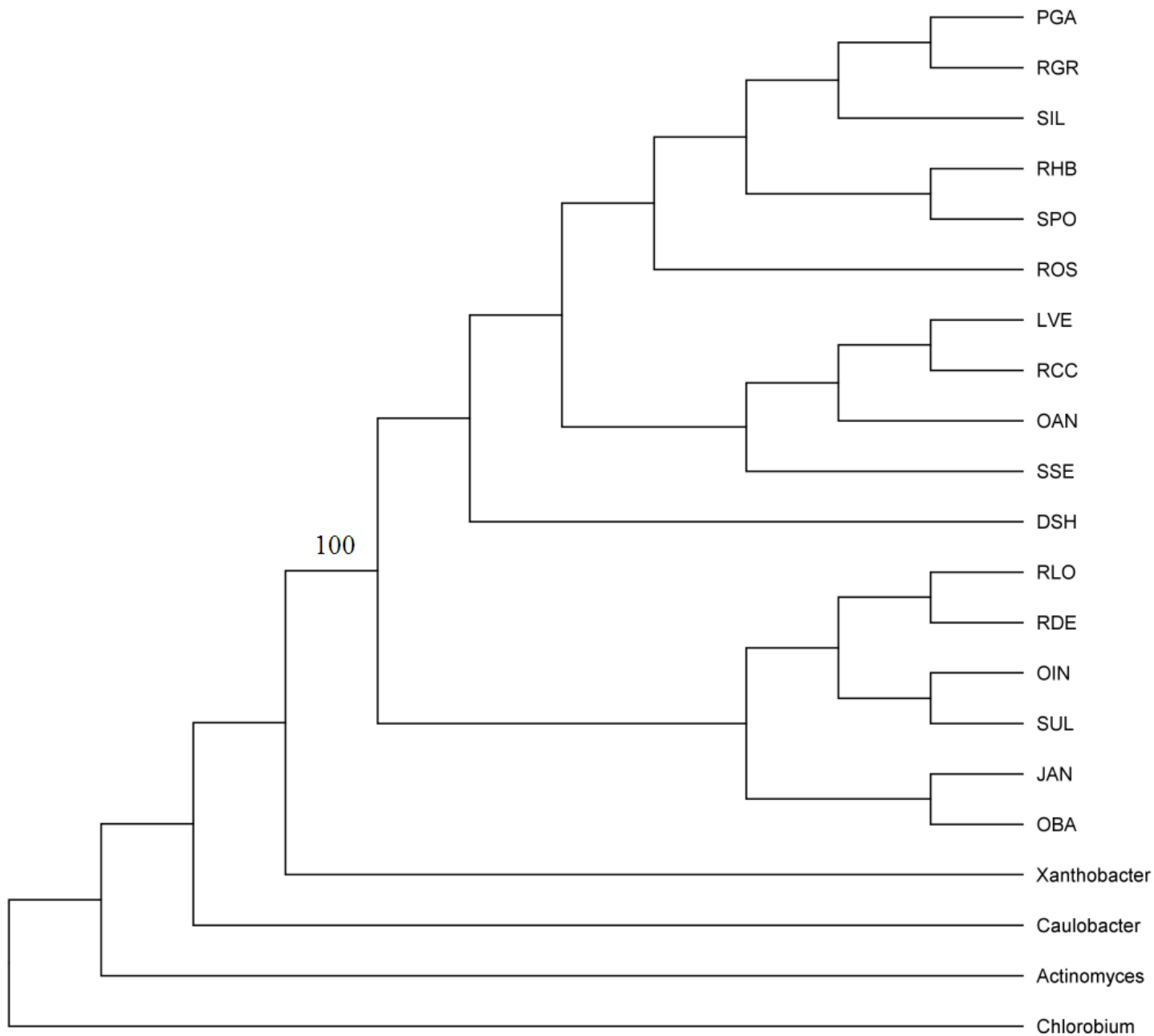

Supplement: File S2 — Tree topologies with the extended data. The multi-documents have been combined into a single ZIP-formatted file. The trees should be considered unrooted. The tree topologies were calculated in PhyML as described in Methods. Numbers refer to bootstrap values. The tree topology (separate pdf) shows that Roseobacter bacteria form a monophyletic group and was deposited in a document named “high bootstrap”. The other organisms embedded within the Roseobacter clade, or Roseobacter bacteria embedded within other phyla are shown in red (deposited in a document named “inter-phylum”). Individual file name corresponds to gene family code listed in Table S1. The non Roseobacter organism taxonomic name is detailed in the amino acid fasta of the sequences (a document named “sequences”). (4.62 MB ZIP) [file pone.0011604.s008.zip › high bootstrap/ort90.pdf]

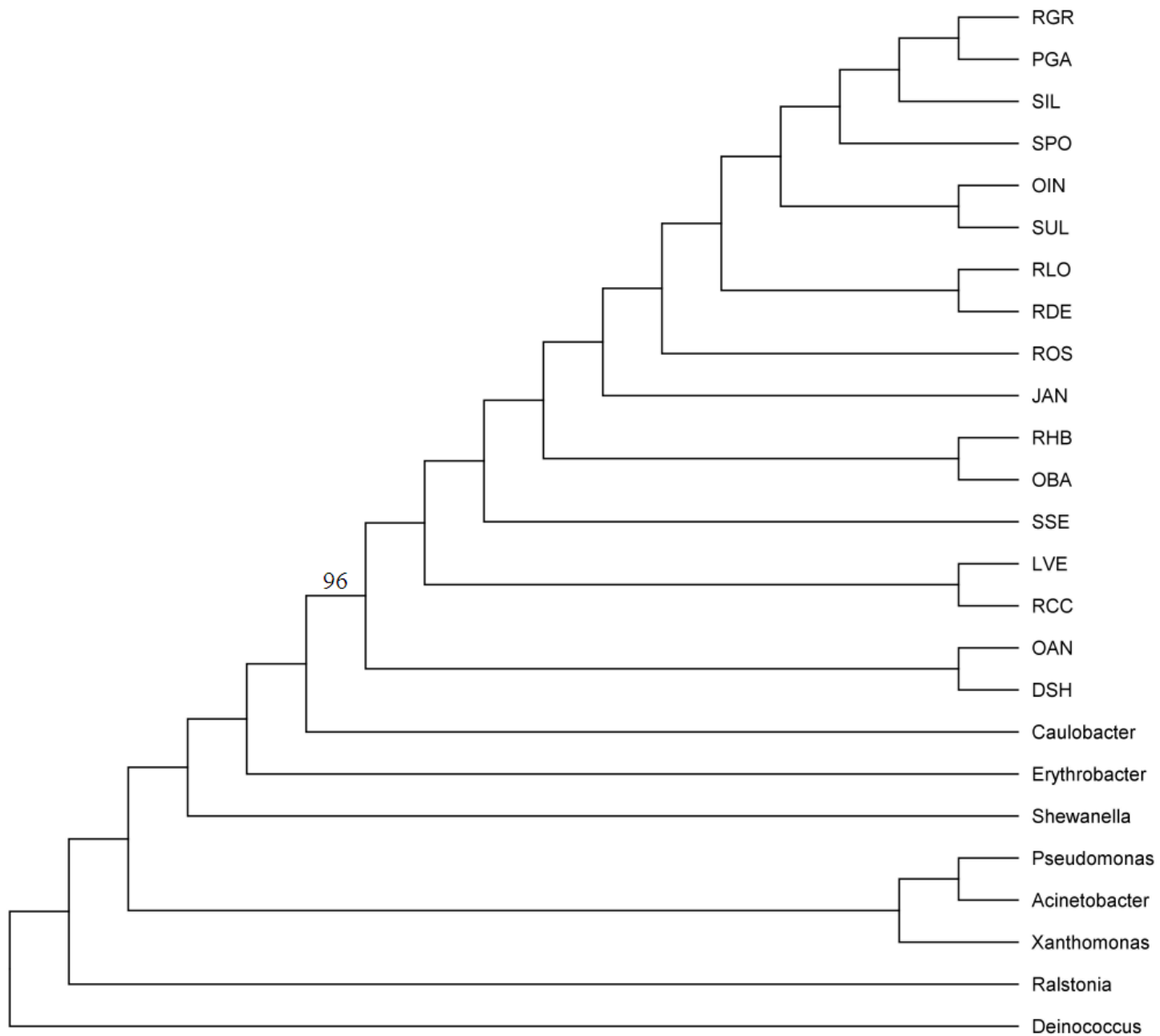

Supplement: File S2 — Tree topologies with the extended data. The multi-documents have been combined into a single ZIP-formatted file. The trees should be considered unrooted. The tree topologies were calculated in PhyML as described in Methods. Numbers refer to bootstrap values. The tree topology (separate pdf) shows that Roseobacter bacteria form a monophyletic group and was deposited in a document named “high bootstrap”. The other organisms embedded within the Roseobacter clade, or Roseobacter bacteria embedded within other phyla are shown in red (deposited in a document named “inter-phylum”). Individual file name corresponds to gene family code listed in Table S1. The non Roseobacter organism taxonomic name is detailed in the amino acid fasta of the sequences (a document named “sequences”). (4.62 MB ZIP) [file pone.0011604.s008.zip › high bootstrap/ort904.pdf]

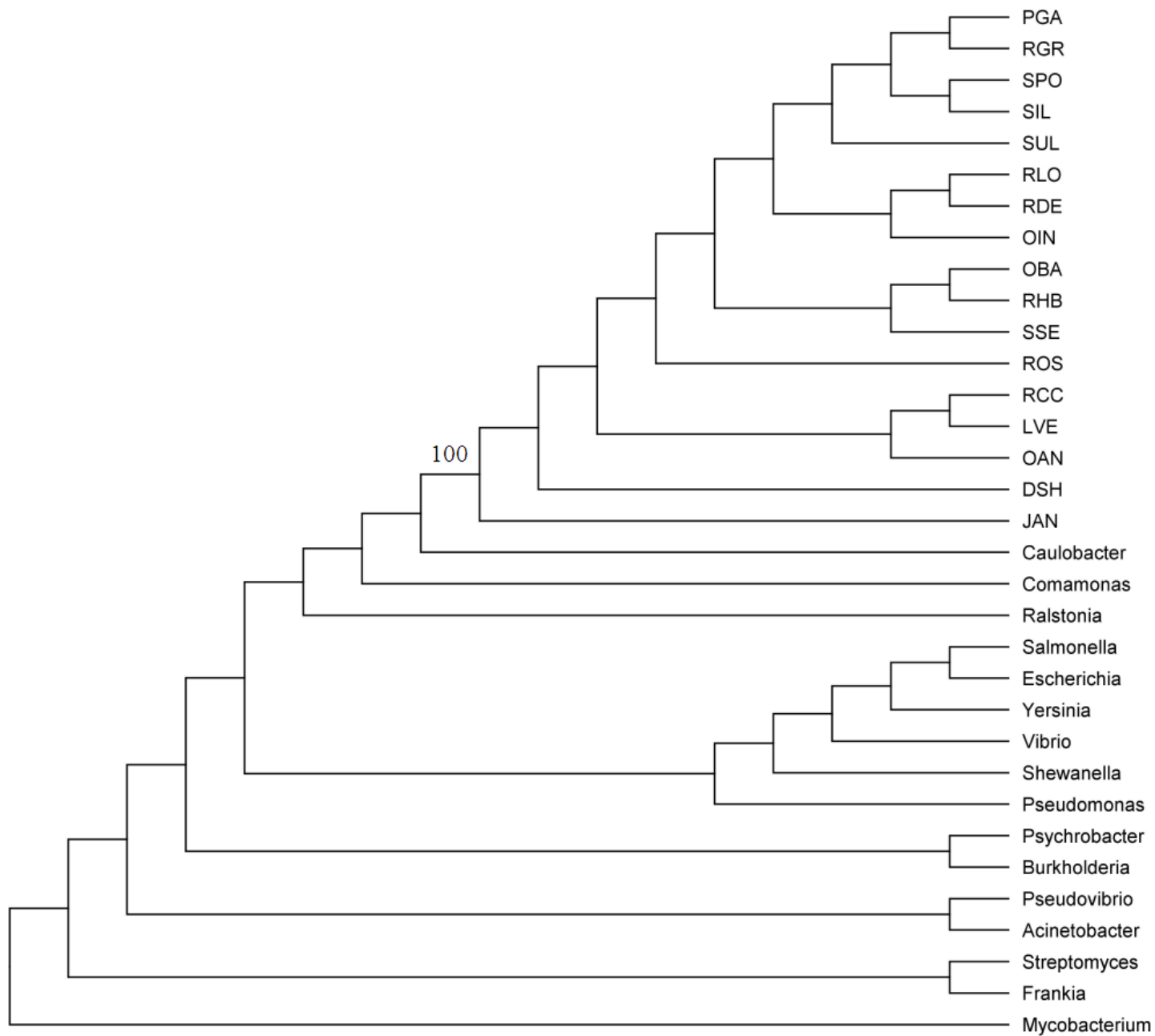

Supplement: File S2 — Tree topologies with the extended data. The multi-documents have been combined into a single ZIP-formatted file. The trees should be considered unrooted. The tree topologies were calculated in PhyML as described in Methods. Numbers refer to bootstrap values. The tree topology (separate pdf) shows that Roseobacter bacteria form a monophyletic group and was deposited in a document named “high bootstrap”. The other organisms embedded within the Roseobacter clade, or Roseobacter bacteria embedded within other phyla are shown in red (deposited in a document named “inter-phylum”). Individual file name corresponds to gene family code listed in Table S1. The non Roseobacter organism taxonomic name is detailed in the amino acid fasta of the sequences (a document named “sequences”). (4.62 MB ZIP) [file pone.0011604.s008.zip › high bootstrap/ort907.pdf]

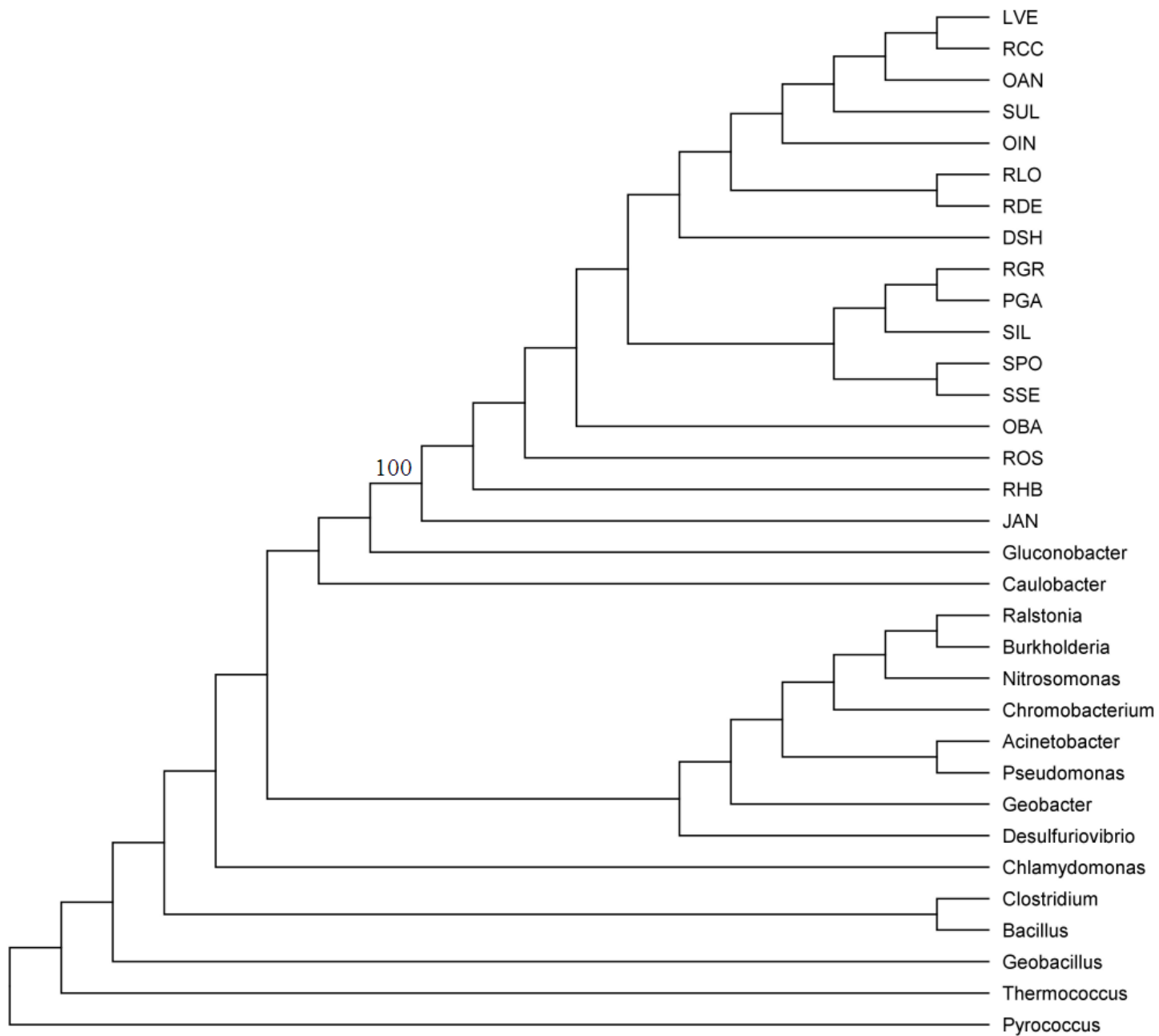

Supplement: File S2 — Tree topologies with the extended data. The multi-documents have been combined into a single ZIP-formatted file. The trees should be considered unrooted. The tree topologies were calculated in PhyML as described in Methods. Numbers refer to bootstrap values. The tree topology (separate pdf) shows that Roseobacter bacteria form a monophyletic group and was deposited in a document named “high bootstrap”. The other organisms embedded within the Roseobacter clade, or Roseobacter bacteria embedded within other phyla are shown in red (deposited in a document named “inter-phylum”). Individual file name corresponds to gene family code listed in Table S1. The non Roseobacter organism taxonomic name is detailed in the amino acid fasta of the sequences (a document named “sequences”). (4.62 MB ZIP) [file pone.0011604.s008.zip › high bootstrap/ort915.pdf]

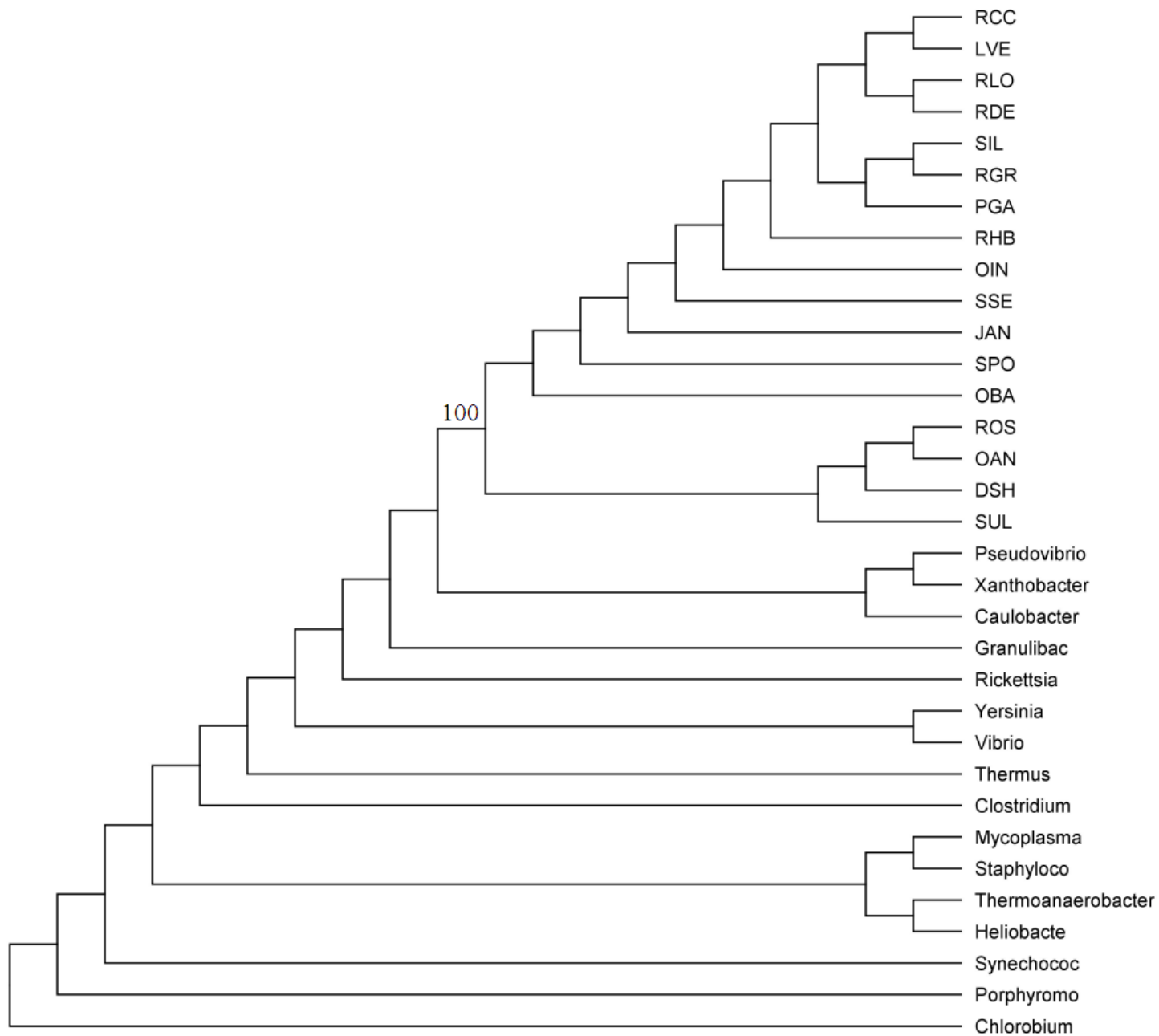

Supplement: File S2 — Tree topologies with the extended data. The multi-documents have been combined into a single ZIP-formatted file. The trees should be considered unrooted. The tree topologies were calculated in PhyML as described in Methods. Numbers refer to bootstrap values. The tree topology (separate pdf) shows that Roseobacter bacteria form a monophyletic group and was deposited in a document named “high bootstrap”. The other organisms embedded within the Roseobacter clade, or Roseobacter bacteria embedded within other phyla are shown in red (deposited in a document named “inter-phylum”). Individual file name corresponds to gene family code listed in Table S1. The non Roseobacter organism taxonomic name is detailed in the amino acid fasta of the sequences (a document named “sequences”). (4.62 MB ZIP) [file pone.0011604.s008.zip › high bootstrap/ort937.pdf]

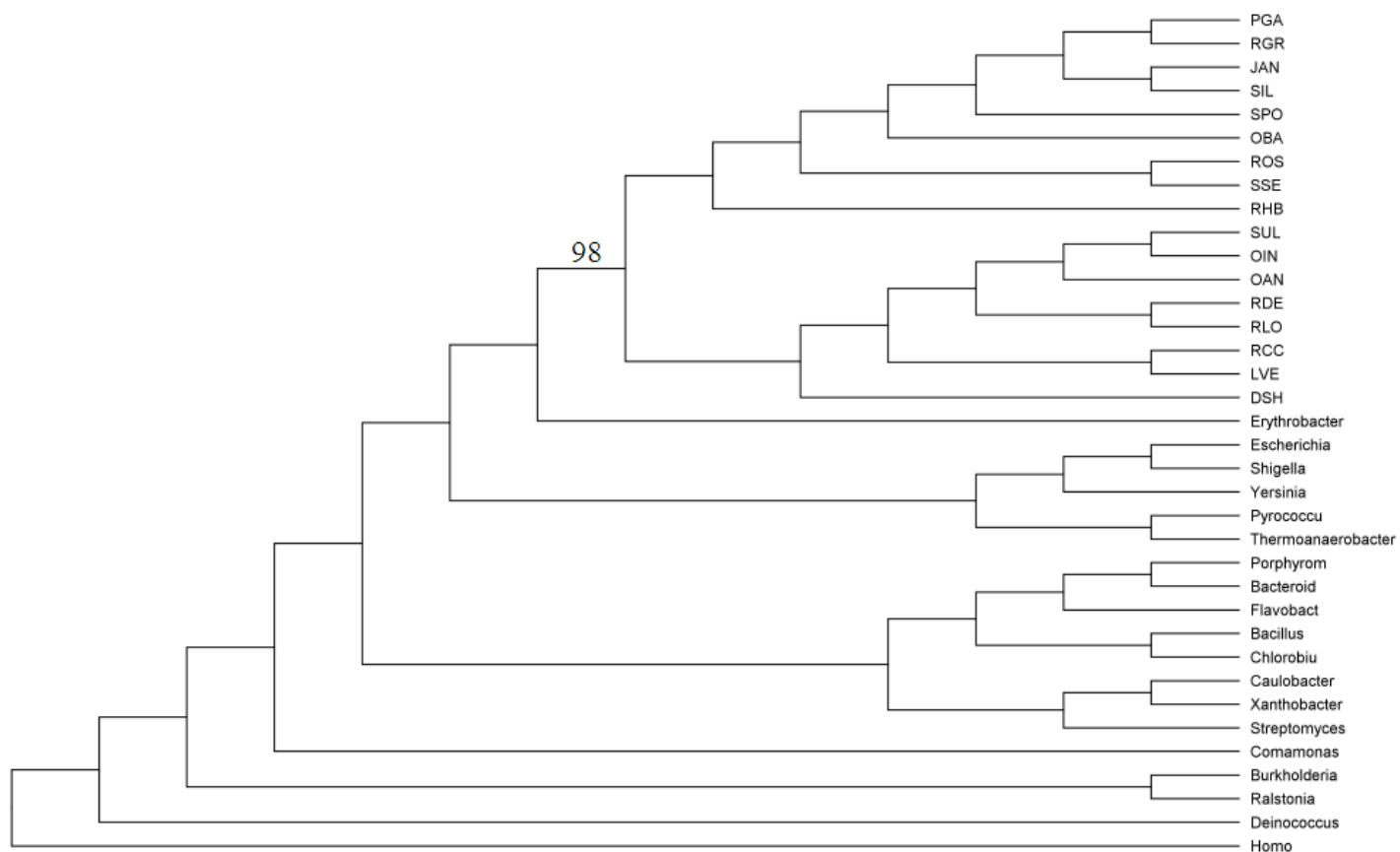

Supplement: File S2 — Tree topologies with the extended data. The multi-documents have been combined into a single ZIP-formatted file. The trees should be considered unrooted. The tree topologies were calculated in PhyML as described in Methods. Numbers refer to bootstrap values. The tree topology (separate pdf) shows that Roseobacter bacteria form a monophyletic group and was deposited in a document named “high bootstrap”. The other organisms embedded within the Roseobacter clade, or Roseobacter bacteria embedded within other phyla are shown in red (deposited in a document named “inter-phylum”). Individual file name corresponds to gene family code listed in Table S1. The non Roseobacter organism taxonomic name is detailed in the amino acid fasta of the sequences (a document named “sequences”). (4.62 MB ZIP) [file pone.0011604.s008.zip › high bootstrap/ort945.pdf]

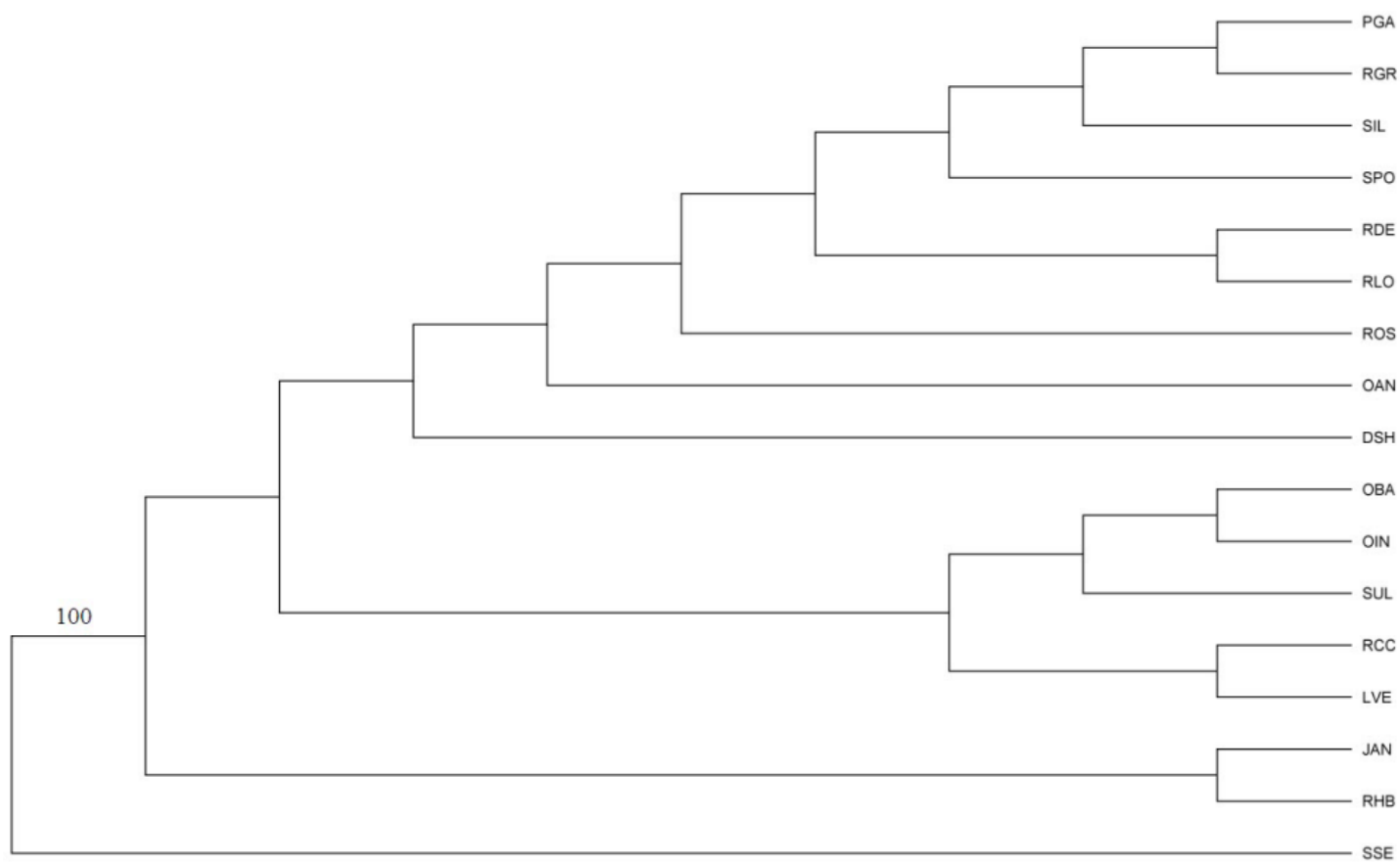

Supplement: File S2 — Tree topologies with the extended data. The multi-documents have been combined into a single ZIP-formatted file. The trees should be considered unrooted. The tree topologies were calculated in PhyML as described in Methods. Numbers refer to bootstrap values. The tree topology (separate pdf) shows that Roseobacter bacteria form a monophyletic group and was deposited in a document named “high bootstrap”. The other organisms embedded within the Roseobacter clade, or Roseobacter bacteria embedded within other phyla are shown in red (deposited in a document named “inter-phylum”). Individual file name corresponds to gene family code listed in Table S1. The non Roseobacter organism taxonomic name is detailed in the amino acid fasta of the sequences (a document named “sequences”). (4.62 MB ZIP) [file pone.0011604.s008.zip › high bootstrap/ort962.pdf]

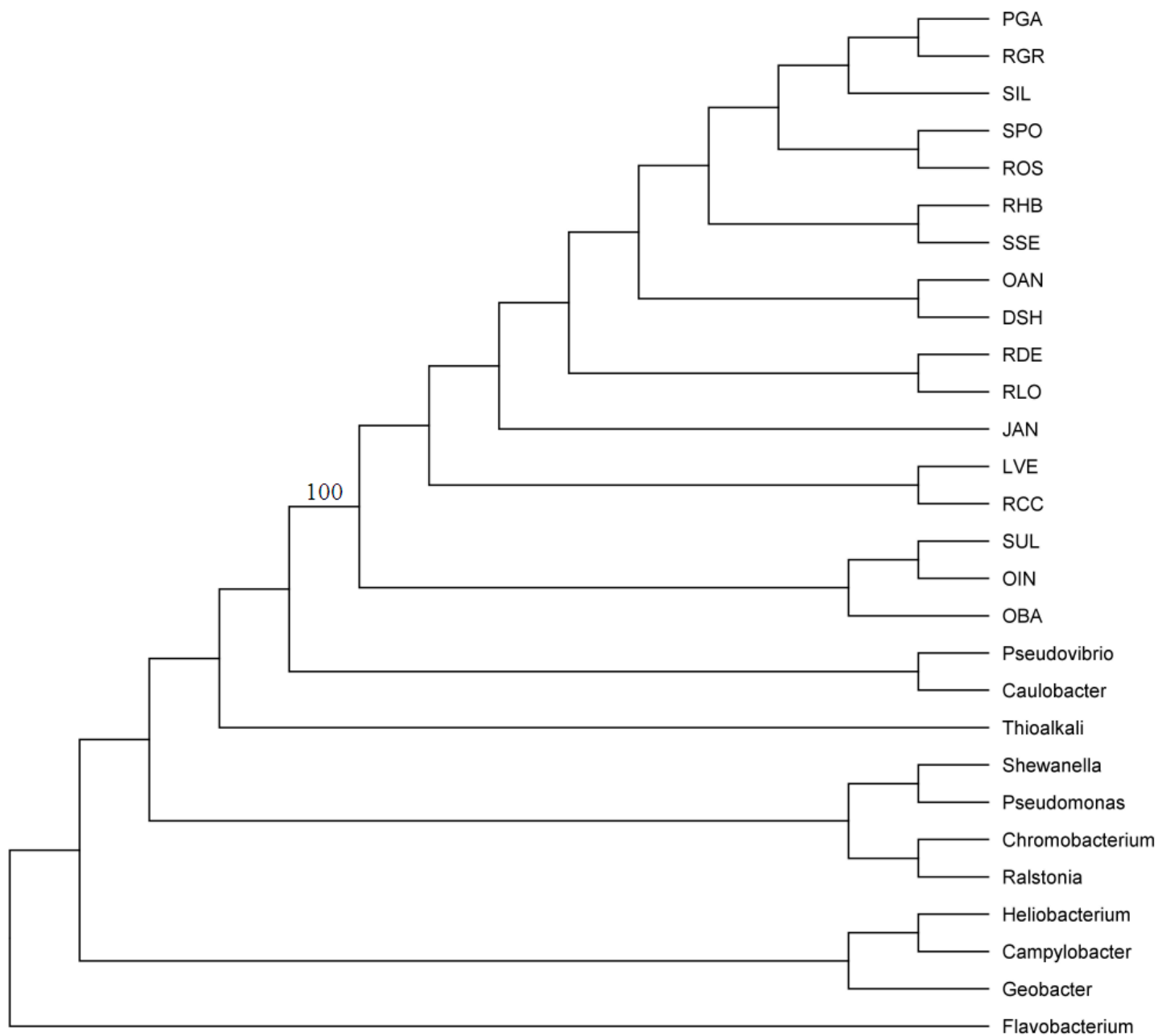

Supplement: File S2 — Tree topologies with the extended data. The multi-documents have been combined into a single ZIP-formatted file. The trees should be considered unrooted. The tree topologies were calculated in PhyML as described in Methods. Numbers refer to bootstrap values. The tree topology (separate pdf) shows that Roseobacter bacteria form a monophyletic group and was deposited in a document named “high bootstrap”. The other organisms embedded within the Roseobacter clade, or Roseobacter bacteria embedded within other phyla are shown in red (deposited in a document named “inter-phylum”). Individual file name corresponds to gene family code listed in Table S1. The non Roseobacter organism taxonomic name is detailed in the amino acid fasta of the sequences (a document named “sequences”). (4.62 MB ZIP) [file pone.0011604.s008.zip › high bootstrap/ort963.pdf]

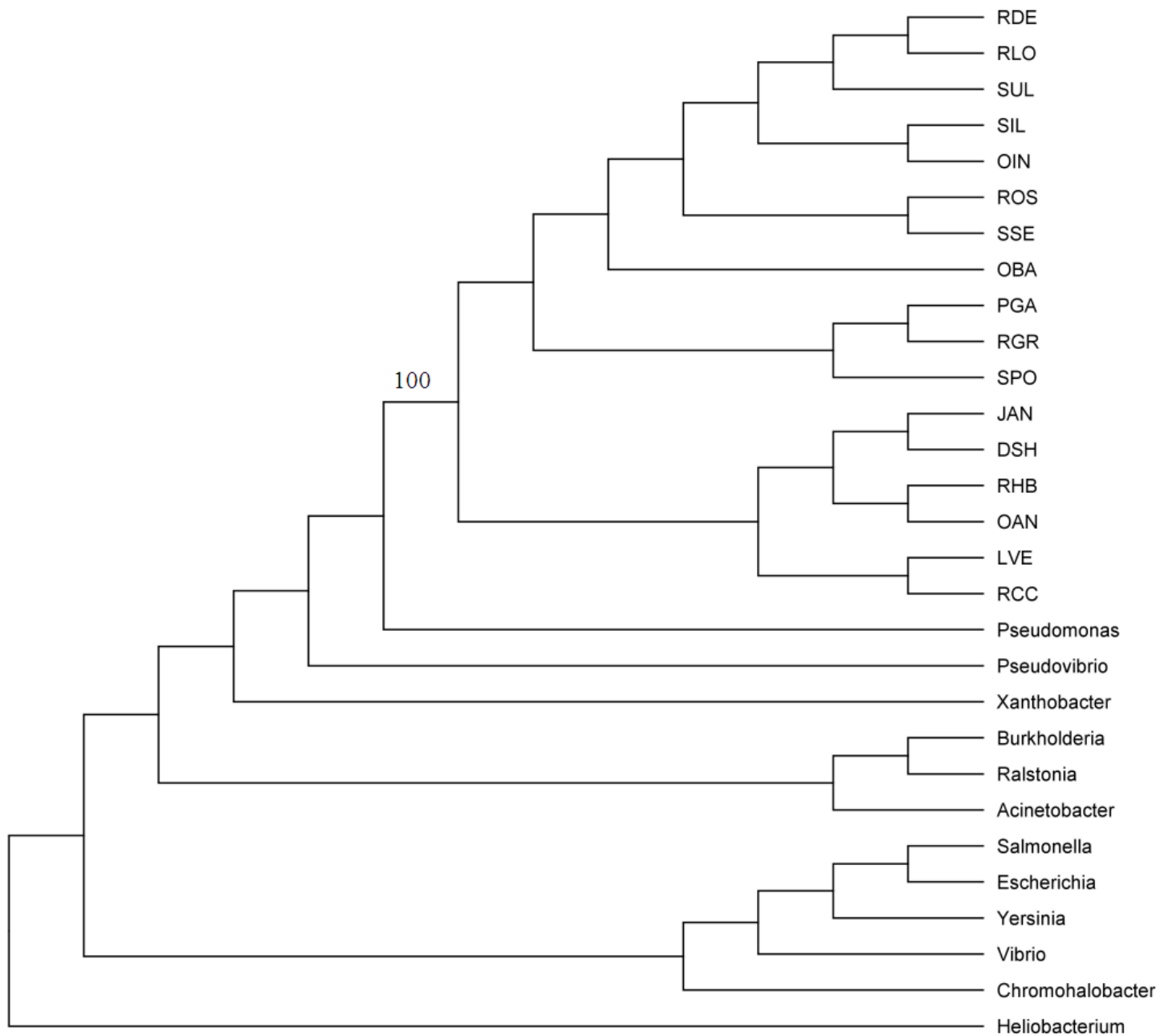

Supplement: File S2 — Tree topologies with the extended data. The multi-documents have been combined into a single ZIP-formatted file. The trees should be considered unrooted. The tree topologies were calculated in PhyML as described in Methods. Numbers refer to bootstrap values. The tree topology (separate pdf) shows that Roseobacter bacteria form a monophyletic group and was deposited in a document named “high bootstrap”. The other organisms embedded within the Roseobacter clade, or Roseobacter bacteria embedded within other phyla are shown in red (deposited in a document named “inter-phylum”). Individual file name corresponds to gene family code listed in Table S1. The non Roseobacter organism taxonomic name is detailed in the amino acid fasta of the sequences (a document named “sequences”). (4.62 MB ZIP) [file pone.0011604.s008.zip › high bootstrap/ort972.pdf]

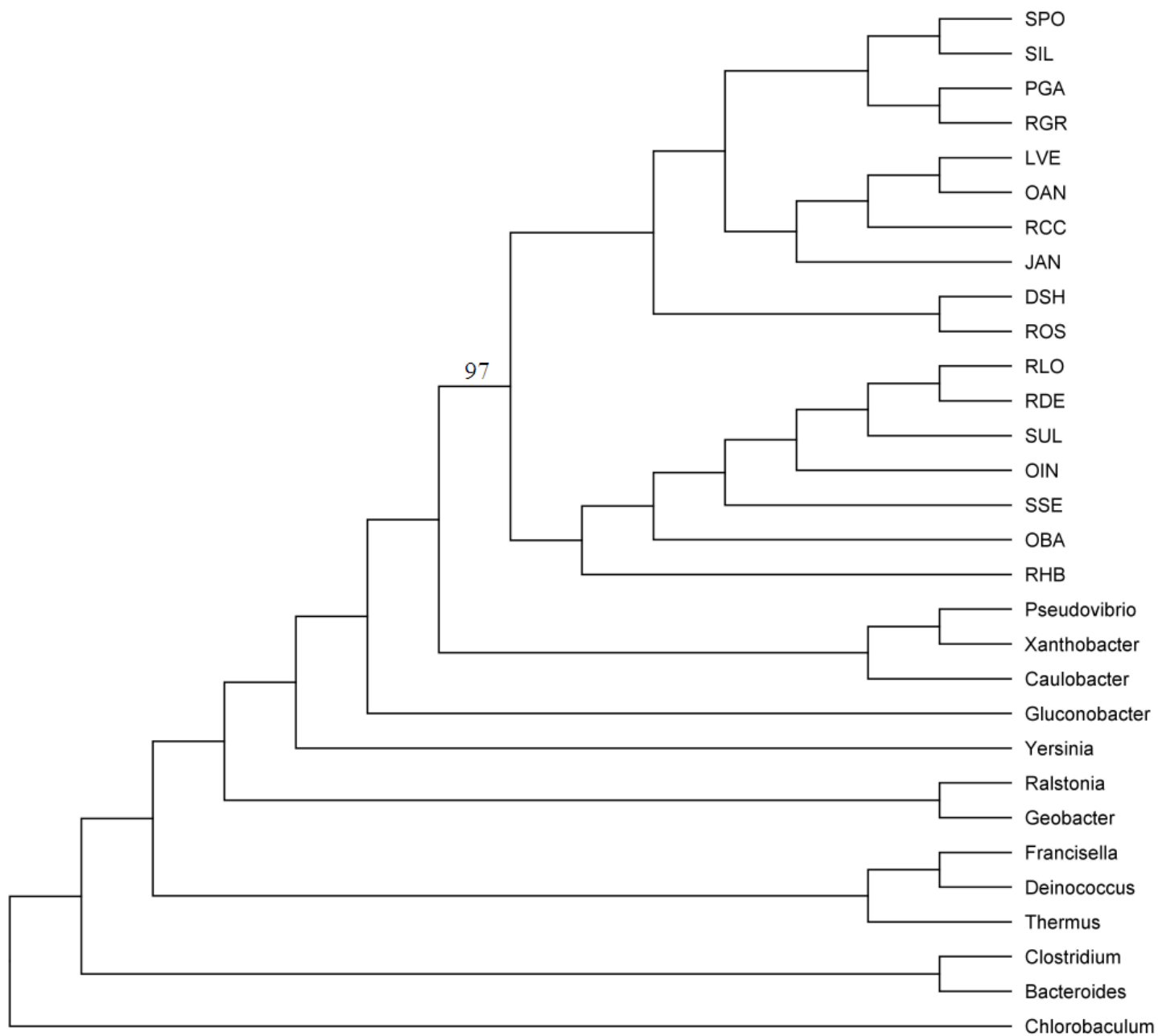

Supplement: File S2 — Tree topologies with the extended data. The multi-documents have been combined into a single ZIP-formatted file. The trees should be considered unrooted. The tree topologies were calculated in PhyML as described in Methods. Numbers refer to bootstrap values. The tree topology (separate pdf) shows that Roseobacter bacteria form a monophyletic group and was deposited in a document named “high bootstrap”. The other organisms embedded within the Roseobacter clade, or Roseobacter bacteria embedded within other phyla are shown in red (deposited in a document named “inter-phylum”). Individual file name corresponds to gene family code listed in Table S1. The non Roseobacter organism taxonomic name is detailed in the amino acid fasta of the sequences (a document named “sequences”). (4.62 MB ZIP) [file pone.0011604.s008.zip › high bootstrap/ort974.pdf]

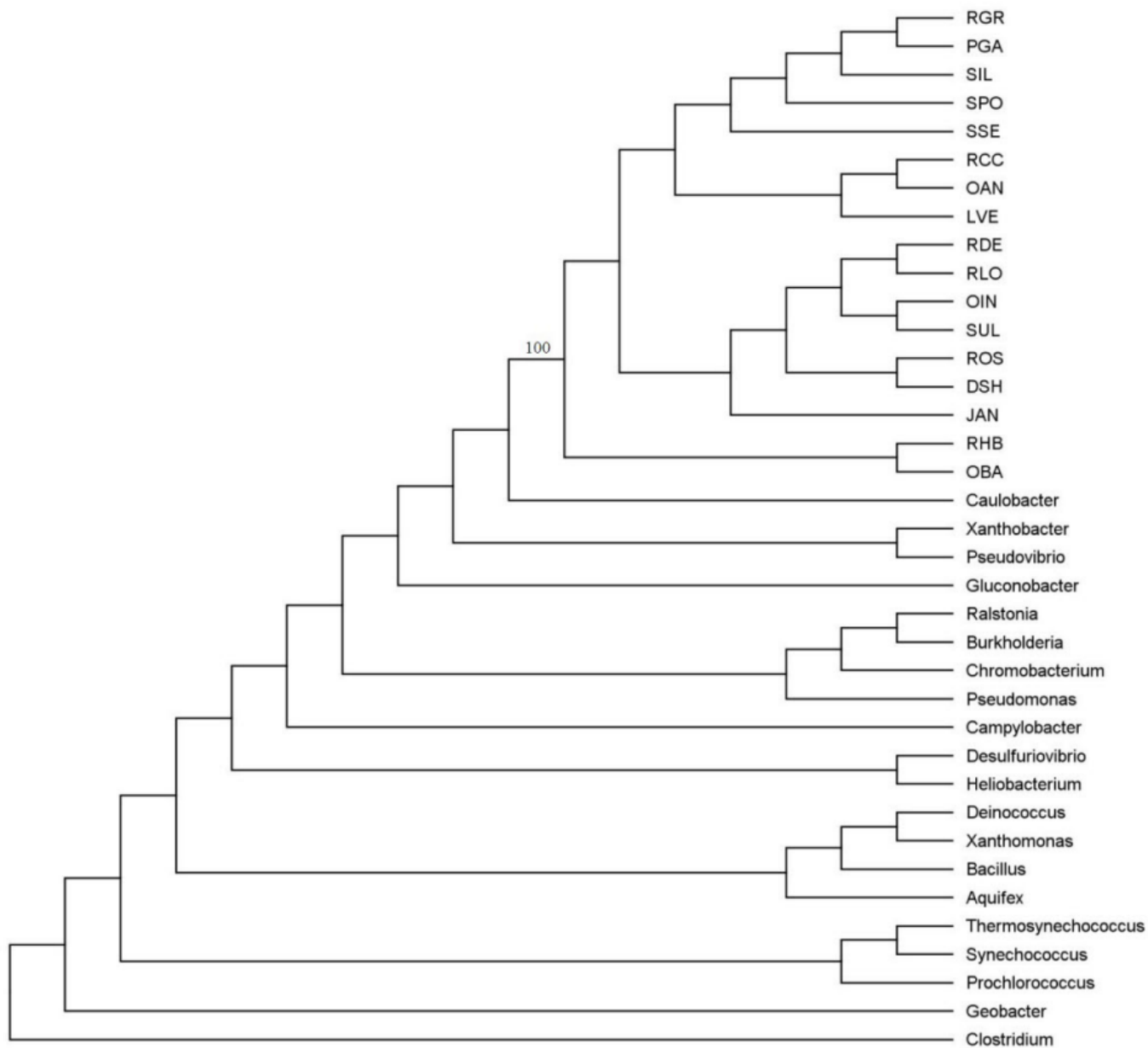

Supplement: File S2 — Tree topologies with the extended data. The multi-documents have been combined into a single ZIP-formatted file. The trees should be considered unrooted. The tree topologies were calculated in PhyML as described in Methods. Numbers refer to bootstrap values. The tree topology (separate pdf) shows that Roseobacter bacteria form a monophyletic group and was deposited in a document named “high bootstrap”. The other organisms embedded within the Roseobacter clade, or Roseobacter bacteria embedded within other phyla are shown in red (deposited in a document named “inter-phylum”). Individual file name corresponds to gene family code listed in Table S1. The non Roseobacter organism taxonomic name is detailed in the amino acid fasta of the sequences (a document named “sequences”). (4.62 MB ZIP) [file pone.0011604.s008.zip › high bootstrap/ort989.pdf]

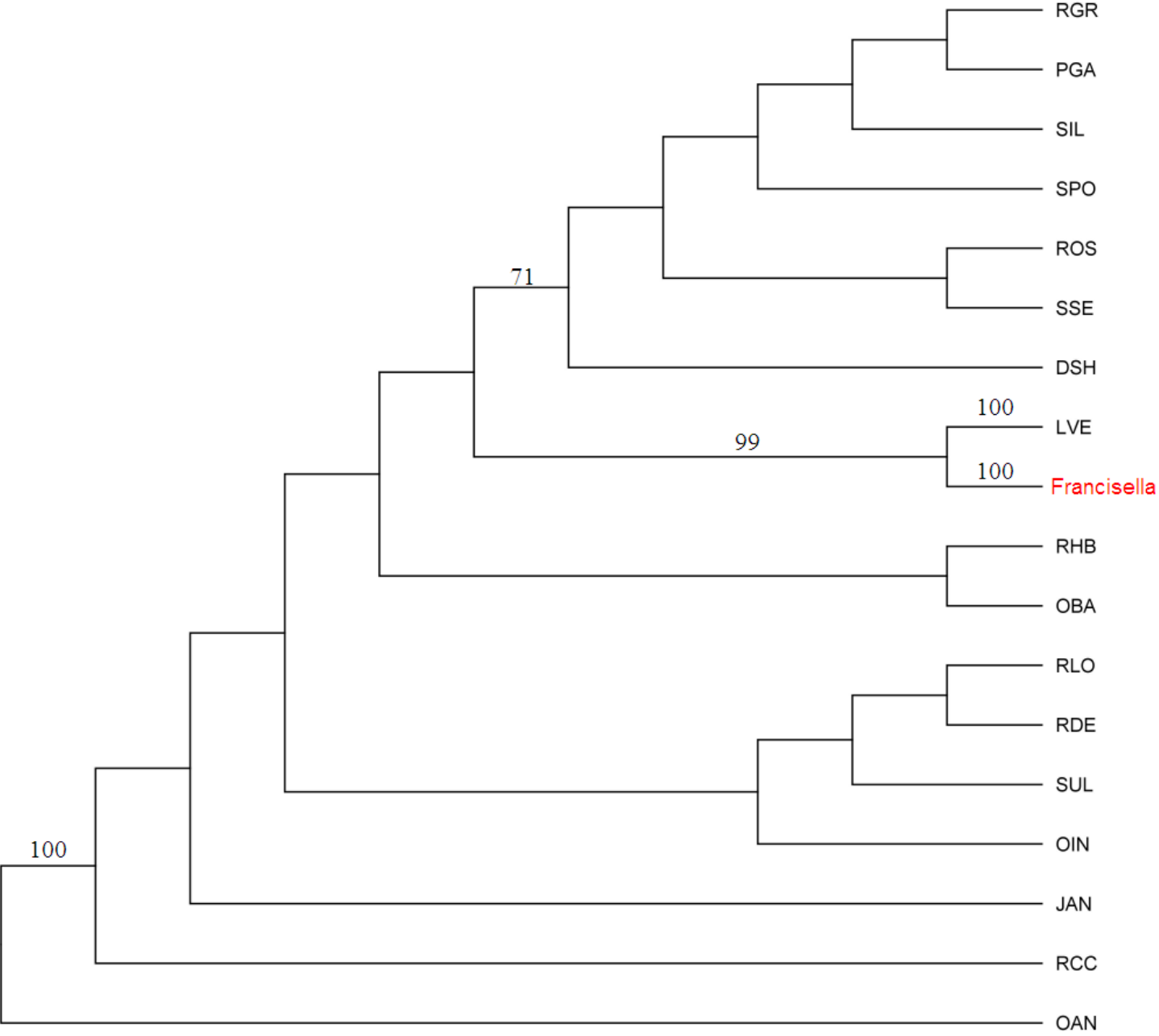

Supplement: File S2 — Tree topologies with the extended data. The multi-documents have been combined into a single ZIP-formatted file. The trees should be considered unrooted. The tree topologies were calculated in PhyML as described in Methods. Numbers refer to bootstrap values. The tree topology (separate pdf) shows that Roseobacter bacteria form a monophyletic group and was deposited in a document named “high bootstrap”. The other organisms embedded within the Roseobacter clade, or Roseobacter bacteria embedded within other phyla are shown in red (deposited in a document named “inter-phylum”). Individual file name corresponds to gene family code listed in Table S1. The non Roseobacter organism taxonomic name is detailed in the amino acid fasta of the sequences (a document named “sequences”). (4.62 MB ZIP) [file pone.0011604.s008.zip › inter-phylum/ort1023.pdf]

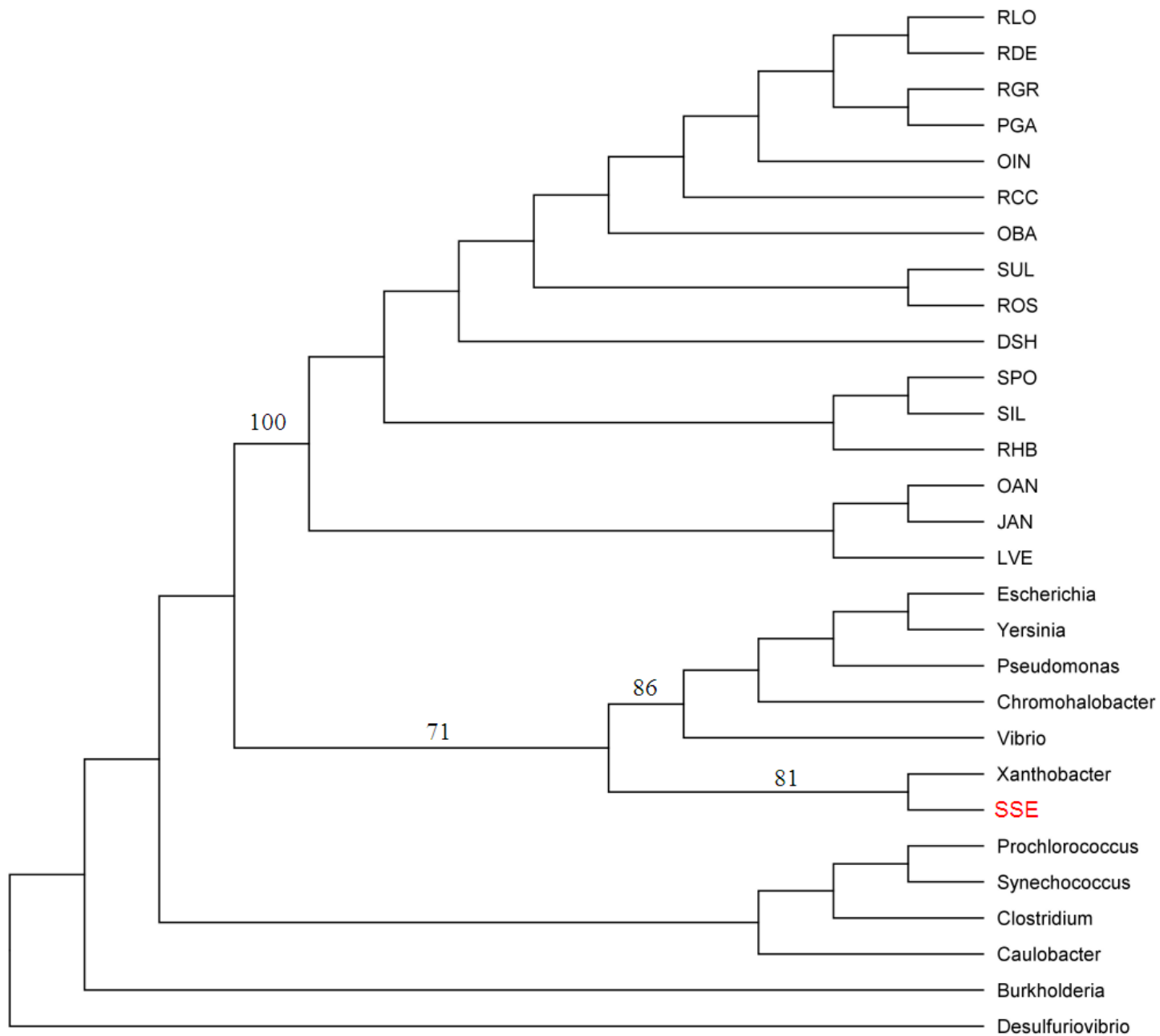

Supplement: File S2 — Tree topologies with the extended data. The multi-documents have been combined into a single ZIP-formatted file. The trees should be considered unrooted. The tree topologies were calculated in PhyML as described in Methods. Numbers refer to bootstrap values. The tree topology (separate pdf) shows that Roseobacter bacteria form a monophyletic group and was deposited in a document named “high bootstrap”. The other organisms embedded within the Roseobacter clade, or Roseobacter bacteria embedded within other phyla are shown in red (deposited in a document named “inter-phylum”). Individual file name corresponds to gene family code listed in Table S1. The non Roseobacter organism taxonomic name is detailed in the amino acid fasta of the sequences (a document named “sequences”). (4.62 MB ZIP) [file pone.0011604.s008.zip › inter-phylum/ort1031.pdf]

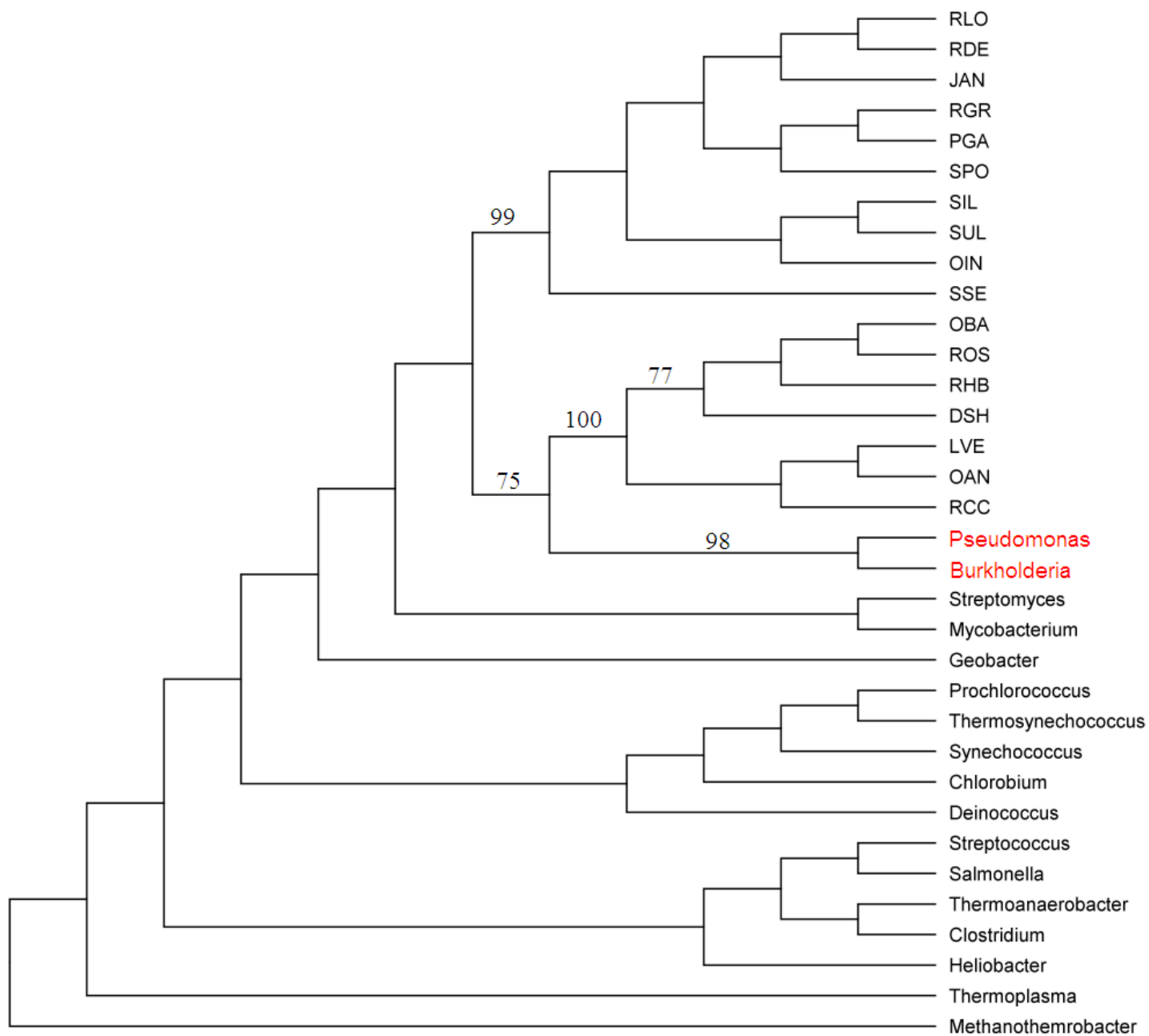

Supplement: File S2 — Tree topologies with the extended data. The multi-documents have been combined into a single ZIP-formatted file. The trees should be considered unrooted. The tree topologies were calculated in PhyML as described in Methods. Numbers refer to bootstrap values. The tree topology (separate pdf) shows that Roseobacter bacteria form a monophyletic group and was deposited in a document named “high bootstrap”. The other organisms embedded within the Roseobacter clade, or Roseobacter bacteria embedded within other phyla are shown in red (deposited in a document named “inter-phylum”). Individual file name corresponds to gene family code listed in Table S1. The non Roseobacter organism taxonomic name is detailed in the amino acid fasta of the sequences (a document named “sequences”). (4.62 MB ZIP) [file pone.0011604.s008.zip › inter-phylum/ort1114.pdf]

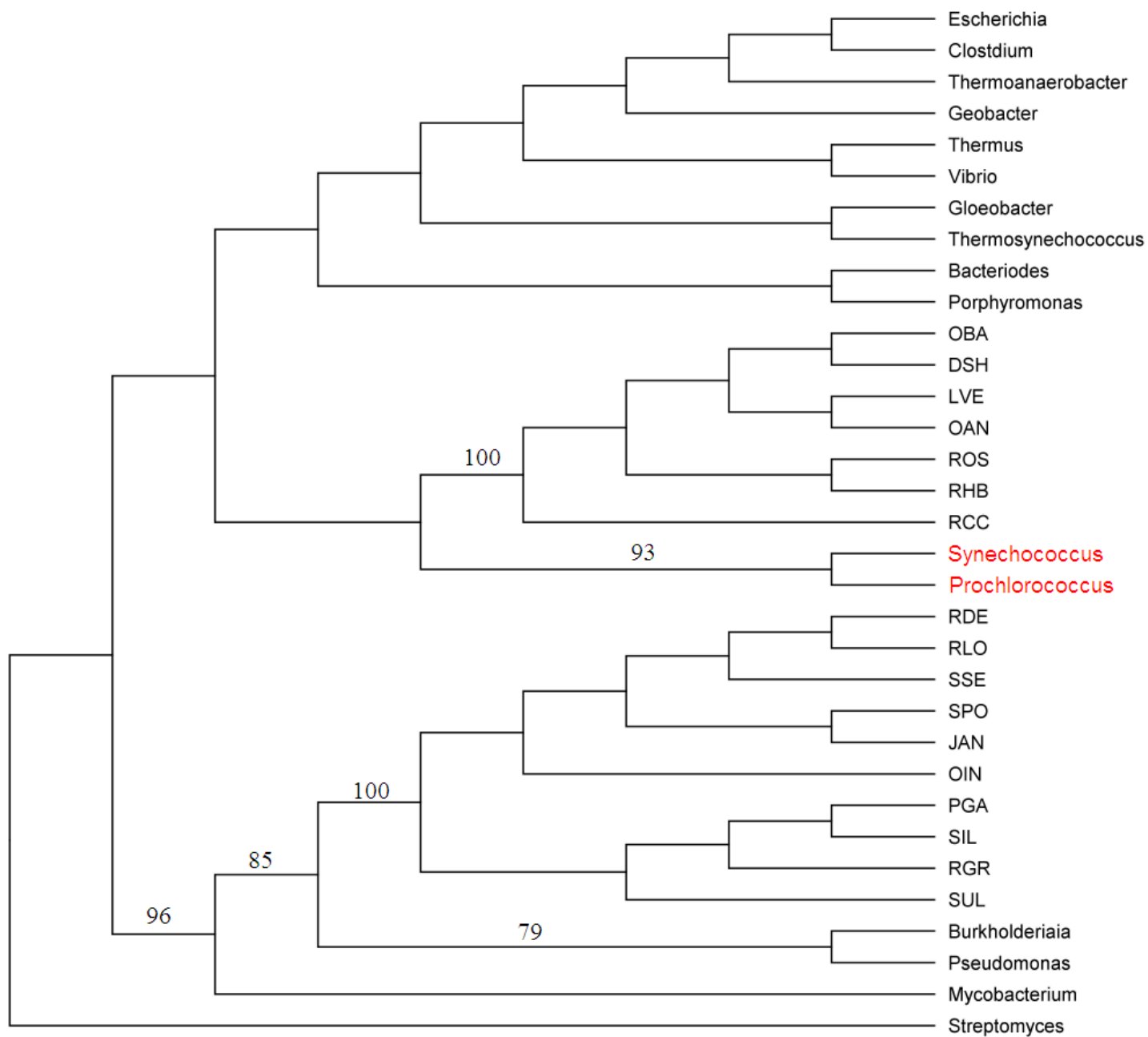

Supplement: File S2 — Tree topologies with the extended data. The multi-documents have been combined into a single ZIP-formatted file. The trees should be considered unrooted. The tree topologies were calculated in PhyML as described in Methods. Numbers refer to bootstrap values. The tree topology (separate pdf) shows that Roseobacter bacteria form a monophyletic group and was deposited in a document named “high bootstrap”. The other organisms embedded within the Roseobacter clade, or Roseobacter bacteria embedded within other phyla are shown in red (deposited in a document named “inter-phylum”). Individual file name corresponds to gene family code listed in Table S1. The non Roseobacter organism taxonomic name is detailed in the amino acid fasta of the sequences (a document named “sequences”). (4.62 MB ZIP) [file pone.0011604.s008.zip › inter-phylum/ort1115.pdf]

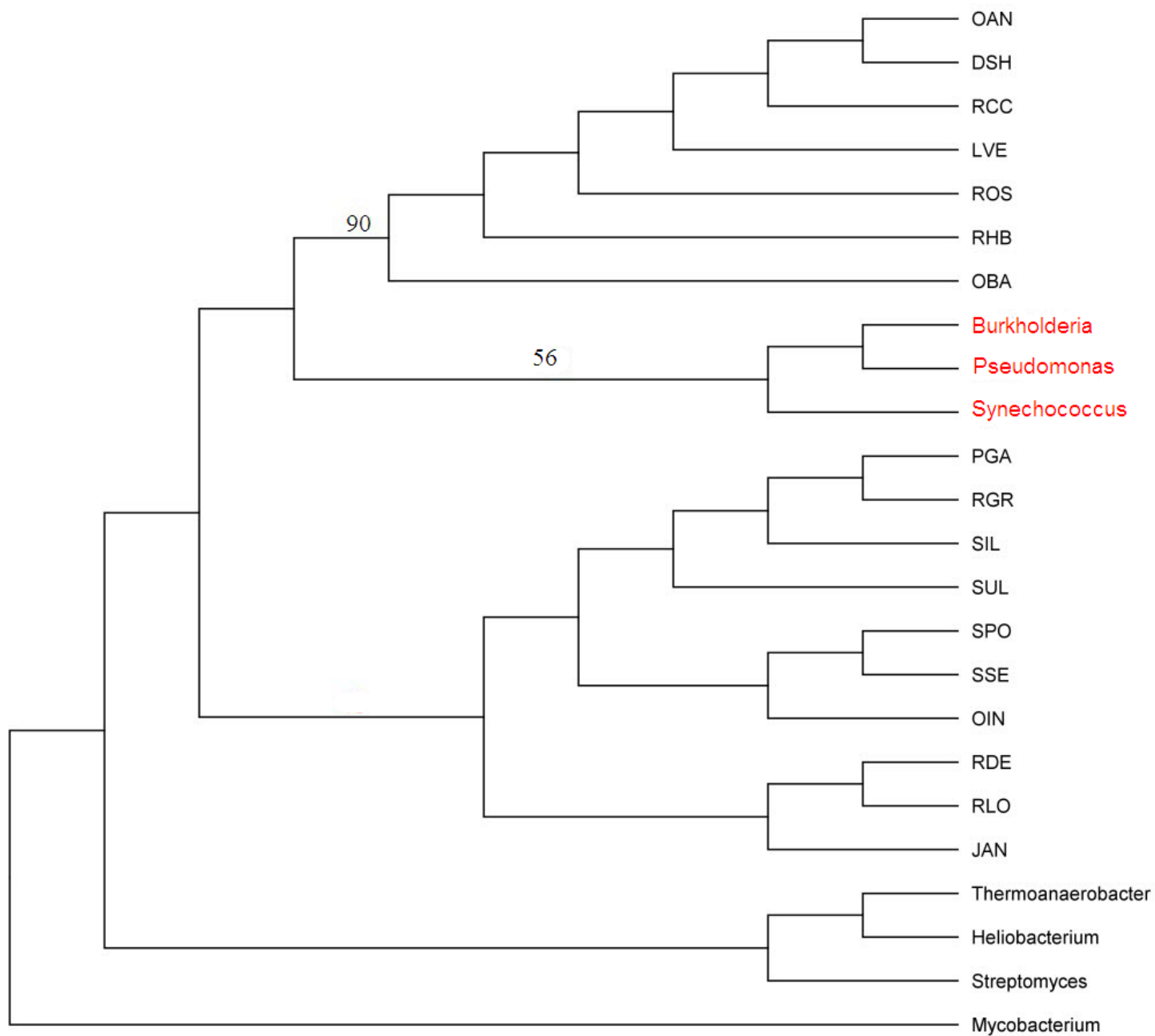

Supplement: File S2 — Tree topologies with the extended data. The multi-documents have been combined into a single ZIP-formatted file. The trees should be considered unrooted. The tree topologies were calculated in PhyML as described in Methods. Numbers refer to bootstrap values. The tree topology (separate pdf) shows that Roseobacter bacteria form a monophyletic group and was deposited in a document named “high bootstrap”. The other organisms embedded within the Roseobacter clade, or Roseobacter bacteria embedded within other phyla are shown in red (deposited in a document named “inter-phylum”). Individual file name corresponds to gene family code listed in Table S1. The non Roseobacter organism taxonomic name is detailed in the amino acid fasta of the sequences (a document named “sequences”). (4.62 MB ZIP) [file pone.0011604.s008.zip › inter-phylum/ort1118.pdf]

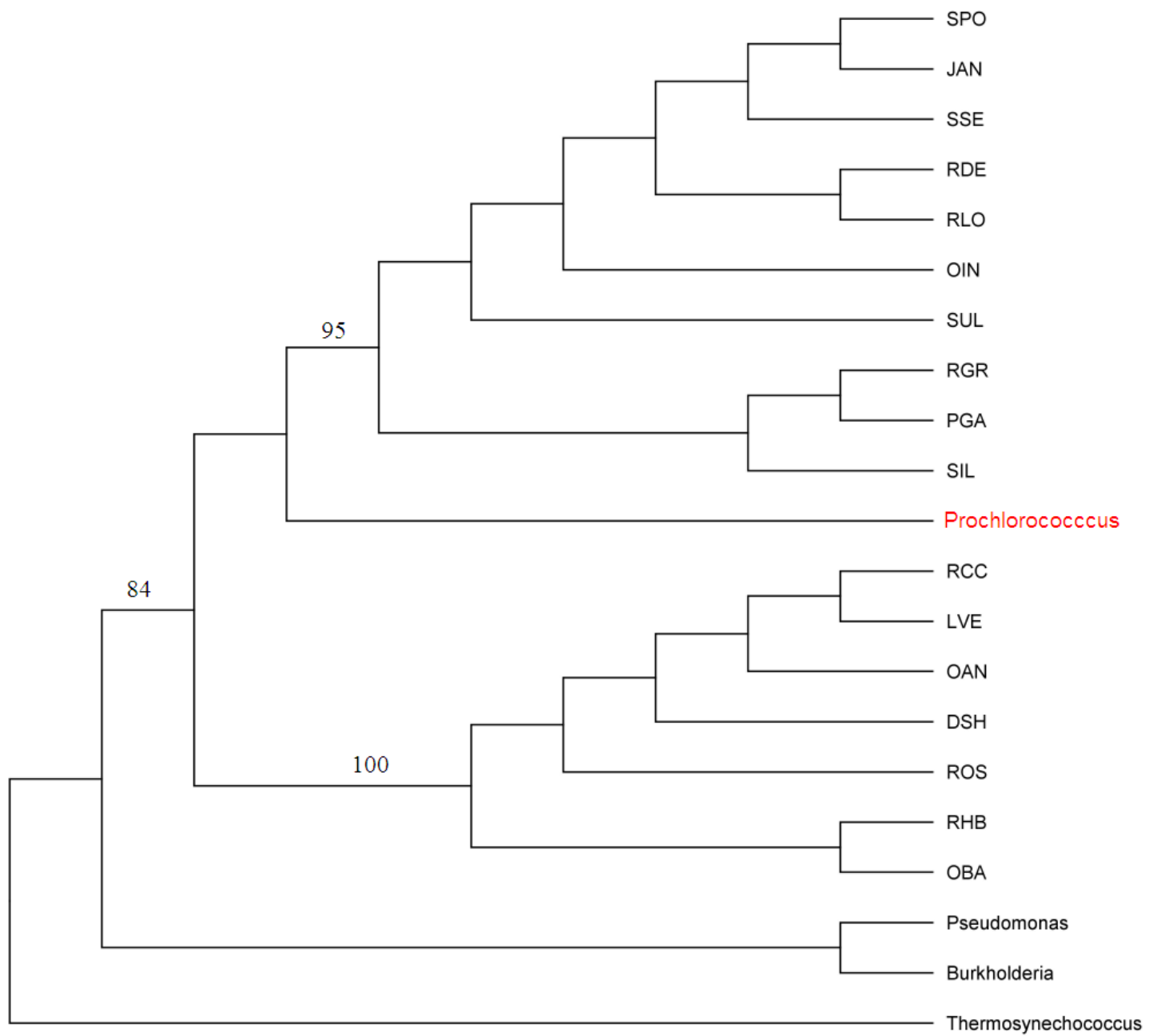

Supplement: File S2 — Tree topologies with the extended data. The multi-documents have been combined into a single ZIP-formatted file. The trees should be considered unrooted. The tree topologies were calculated in PhyML as described in Methods. Numbers refer to bootstrap values. The tree topology (separate pdf) shows that Roseobacter bacteria form a monophyletic group and was deposited in a document named “high bootstrap”. The other organisms embedded within the Roseobacter clade, or Roseobacter bacteria embedded within other phyla are shown in red (deposited in a document named “inter-phylum”). Individual file name corresponds to gene family code listed in Table S1. The non Roseobacter organism taxonomic name is detailed in the amino acid fasta of the sequences (a document named “sequences”). (4.62 MB ZIP) [file pone.0011604.s008.zip › inter-phylum/ort1123.pdf]

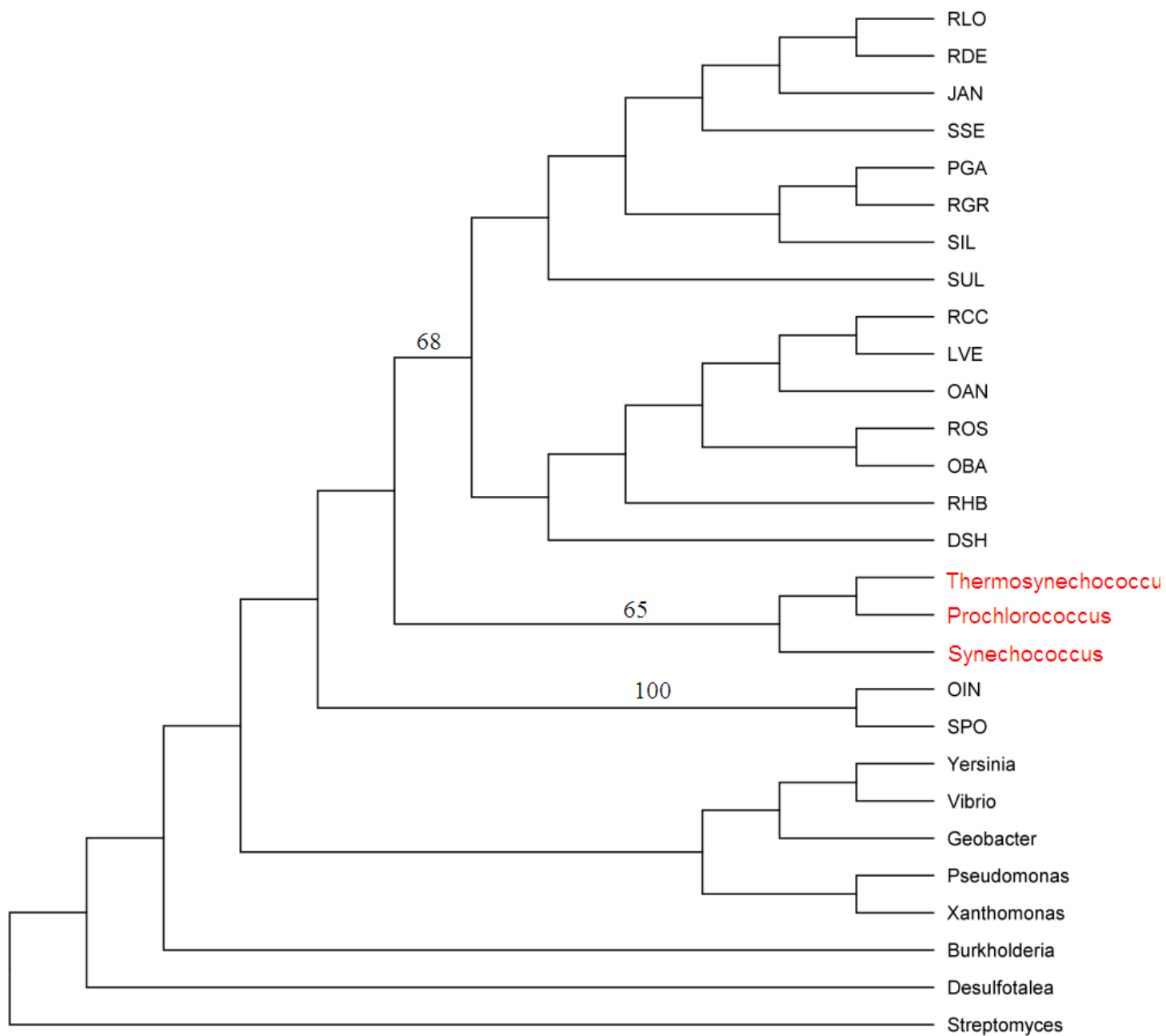

Supplement: File S2 — Tree topologies with the extended data. The multi-documents have been combined into a single ZIP-formatted file. The trees should be considered unrooted. The tree topologies were calculated in PhyML as described in Methods. Numbers refer to bootstrap values. The tree topology (separate pdf) shows that Roseobacter bacteria form a monophyletic group and was deposited in a document named “high bootstrap”. The other organisms embedded within the Roseobacter clade, or Roseobacter bacteria embedded within other phyla are shown in red (deposited in a document named “inter-phylum”). Individual file name corresponds to gene family code listed in Table S1. The non Roseobacter organism taxonomic name is detailed in the amino acid fasta of the sequences (a document named “sequences”). (4.62 MB ZIP) [file pone.0011604.s008.zip › inter-phylum/ort1125.pdf]

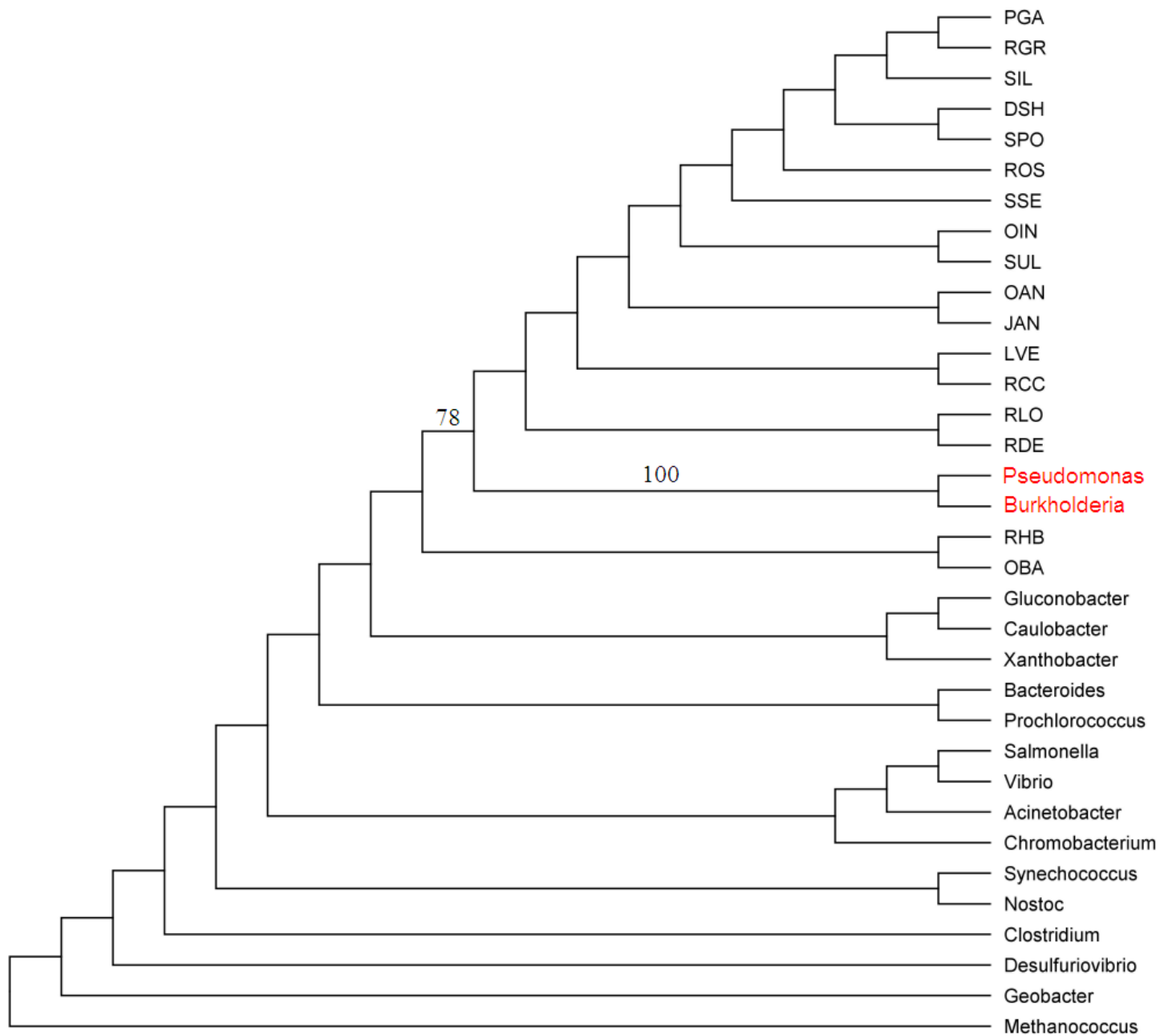

Supplement: File S2 — Tree topologies with the extended data. The multi-documents have been combined into a single ZIP-formatted file. The trees should be considered unrooted. The tree topologies were calculated in PhyML as described in Methods. Numbers refer to bootstrap values. The tree topology (separate pdf) shows that Roseobacter bacteria form a monophyletic group and was deposited in a document named “high bootstrap”. The other organisms embedded within the Roseobacter clade, or Roseobacter bacteria embedded within other phyla are shown in red (deposited in a document named “inter-phylum”). Individual file name corresponds to gene family code listed in Table S1. The non Roseobacter organism taxonomic name is detailed in the amino acid fasta of the sequences (a document named “sequences”). (4.62 MB ZIP) [file pone.0011604.s008.zip › inter-phylum/ort118.pdf]

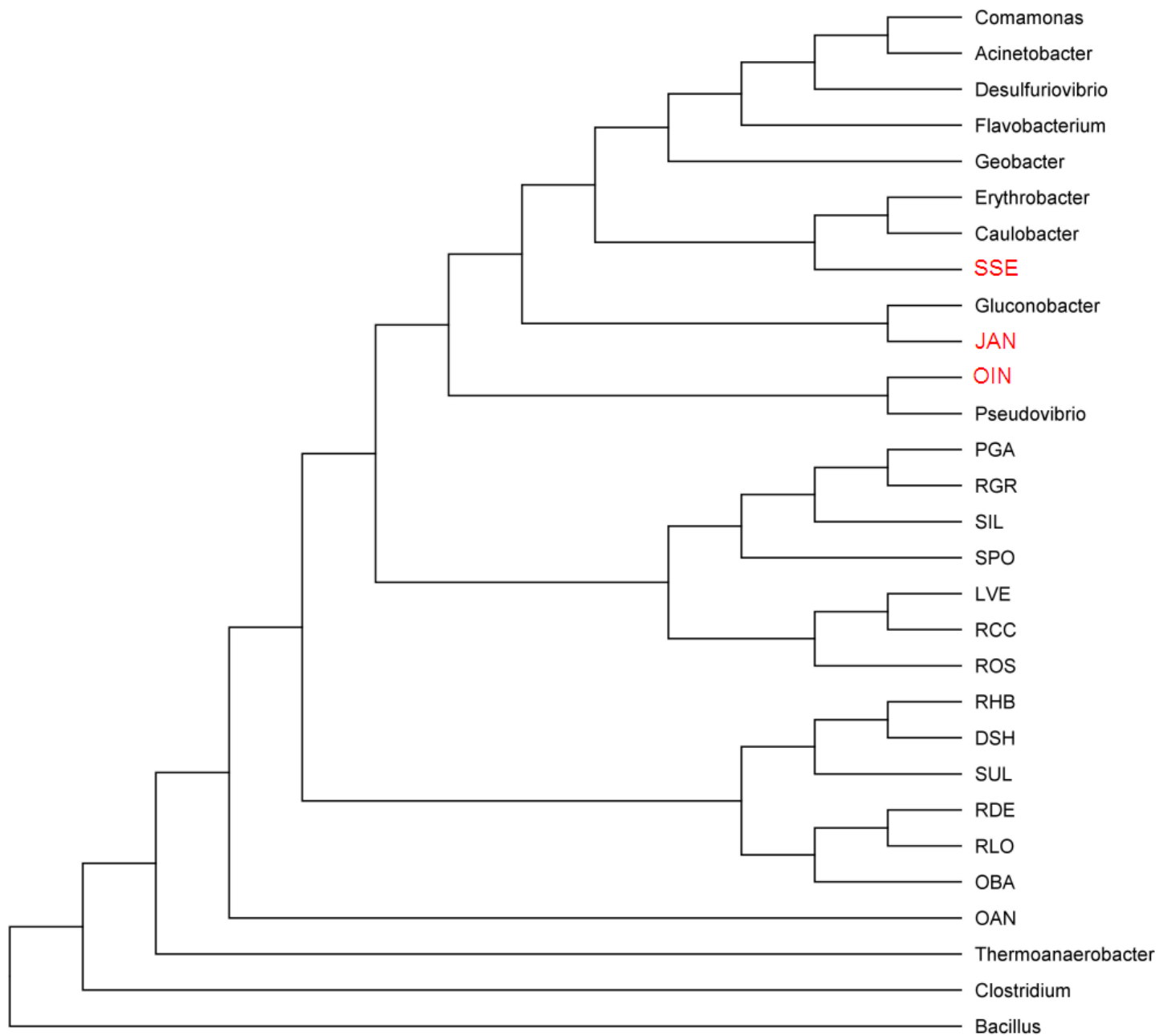

Supplement: File S2 — Tree topologies with the extended data. The multi-documents have been combined into a single ZIP-formatted file. The trees should be considered unrooted. The tree topologies were calculated in PhyML as described in Methods. Numbers refer to bootstrap values. The tree topology (separate pdf) shows that Roseobacter bacteria form a monophyletic group and was deposited in a document named “high bootstrap”. The other organisms embedded within the Roseobacter clade, or Roseobacter bacteria embedded within other phyla are shown in red (deposited in a document named “inter-phylum”). Individual file name corresponds to gene family code listed in Table S1. The non Roseobacter organism taxonomic name is detailed in the amino acid fasta of the sequences (a document named “sequences”). (4.62 MB ZIP) [file pone.0011604.s008.zip › inter-phylum/ort324.pdf]

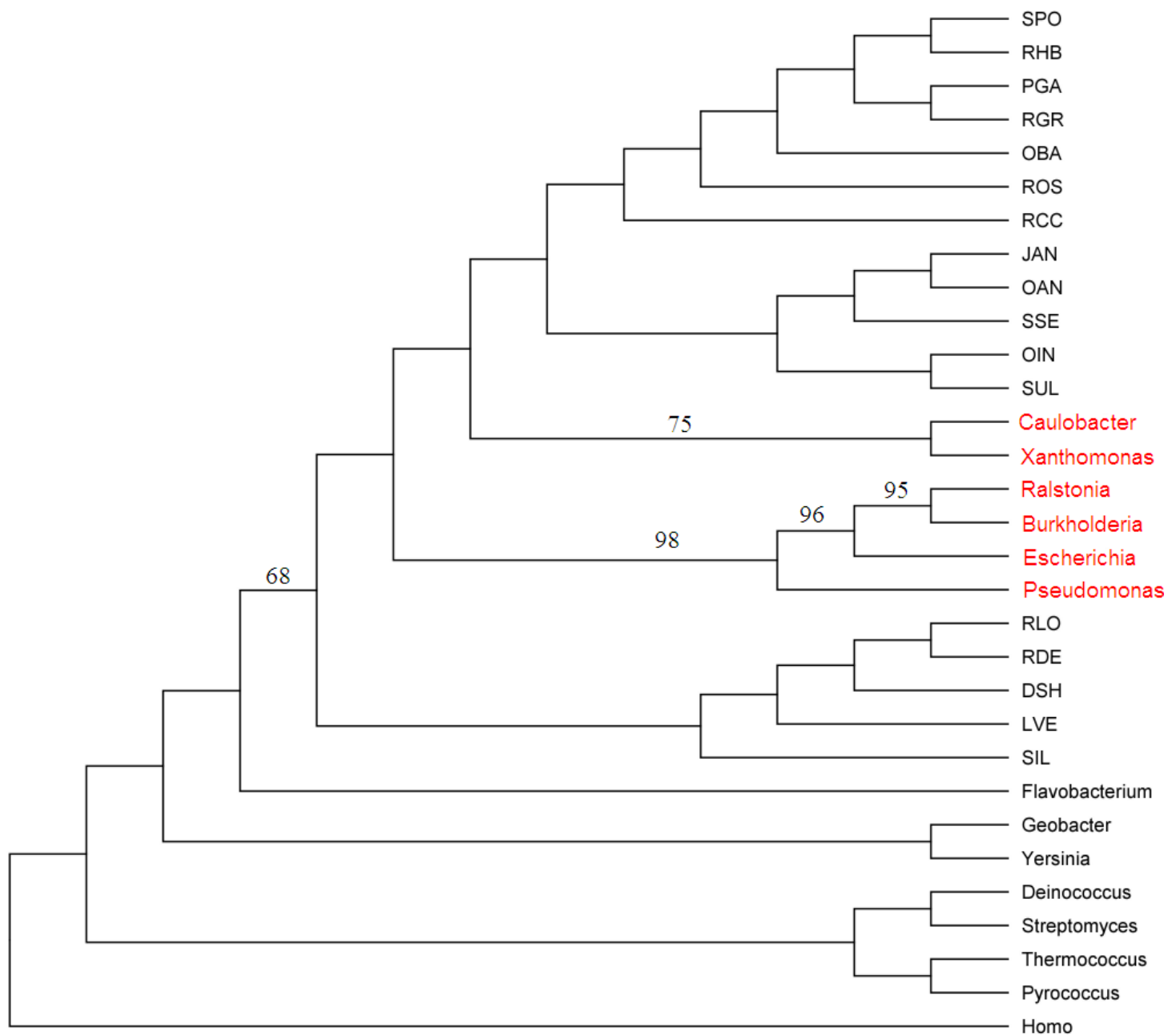

Supplement: File S2 — Tree topologies with the extended data. The multi-documents have been combined into a single ZIP-formatted file. The trees should be considered unrooted. The tree topologies were calculated in PhyML as described in Methods. Numbers refer to bootstrap values. The tree topology (separate pdf) shows that Roseobacter bacteria form a monophyletic group and was deposited in a document named “high bootstrap”. The other organisms embedded within the Roseobacter clade, or Roseobacter bacteria embedded within other phyla are shown in red (deposited in a document named “inter-phylum”). Individual file name corresponds to gene family code listed in Table S1. The non Roseobacter organism taxonomic name is detailed in the amino acid fasta of the sequences (a document named “sequences”). (4.62 MB ZIP) [file pone.0011604.s008.zip › inter-phylum/ort347.pdf]

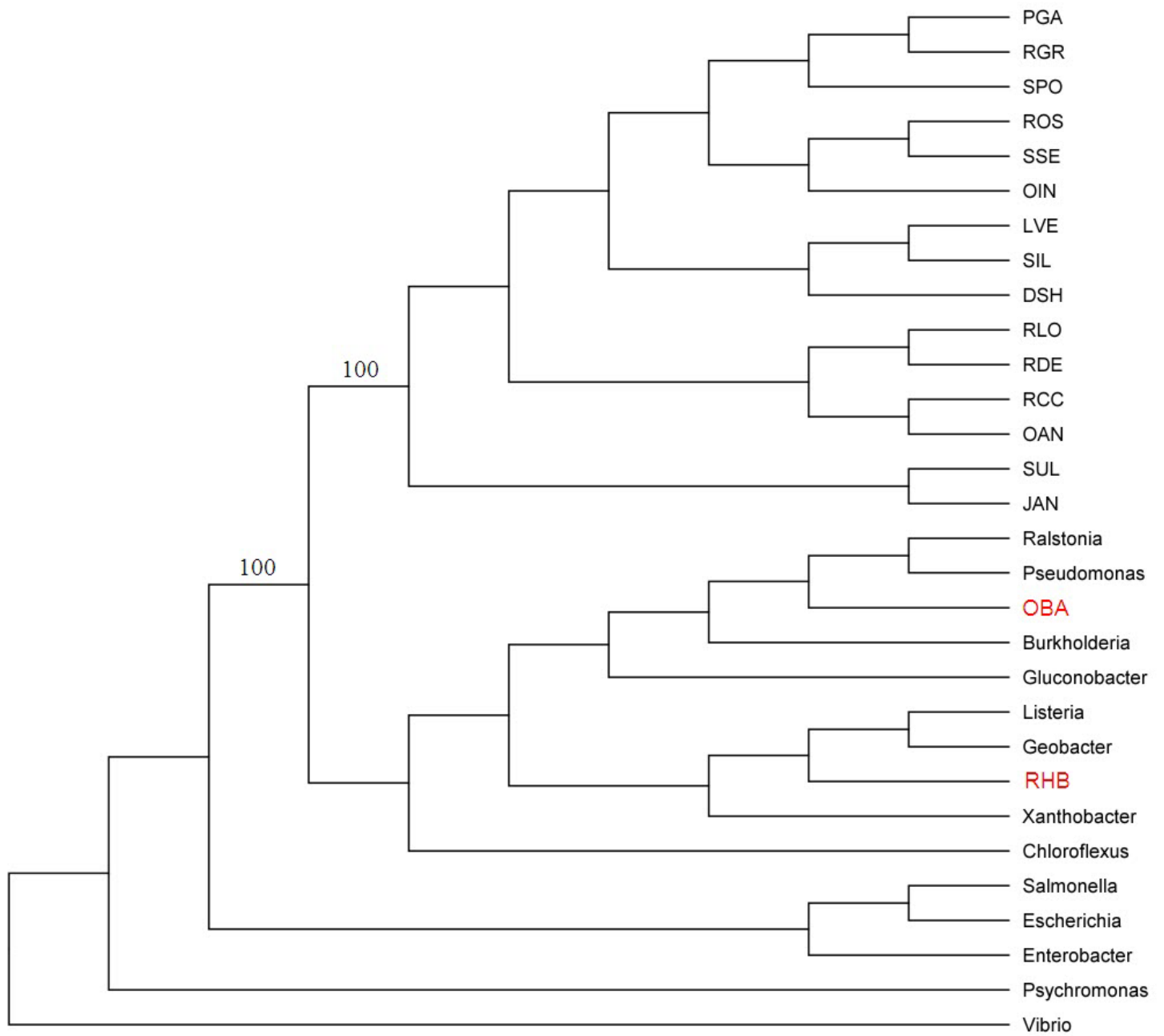

Supplement: File S2 — Tree topologies with the extended data. The multi-documents have been combined into a single ZIP-formatted file. The trees should be considered unrooted. The tree topologies were calculated in PhyML as described in Methods. Numbers refer to bootstrap values. The tree topology (separate pdf) shows that Roseobacter bacteria form a monophyletic group and was deposited in a document named “high bootstrap”. The other organisms embedded within the Roseobacter clade, or Roseobacter bacteria embedded within other phyla are shown in red (deposited in a document named “inter-phylum”). Individual file name corresponds to gene family code listed in Table S1. The non Roseobacter organism taxonomic name is detailed in the amino acid fasta of the sequences (a document named “sequences”). (4.62 MB ZIP) [file pone.0011604.s008.zip › inter-phylum/ort402.pdf]

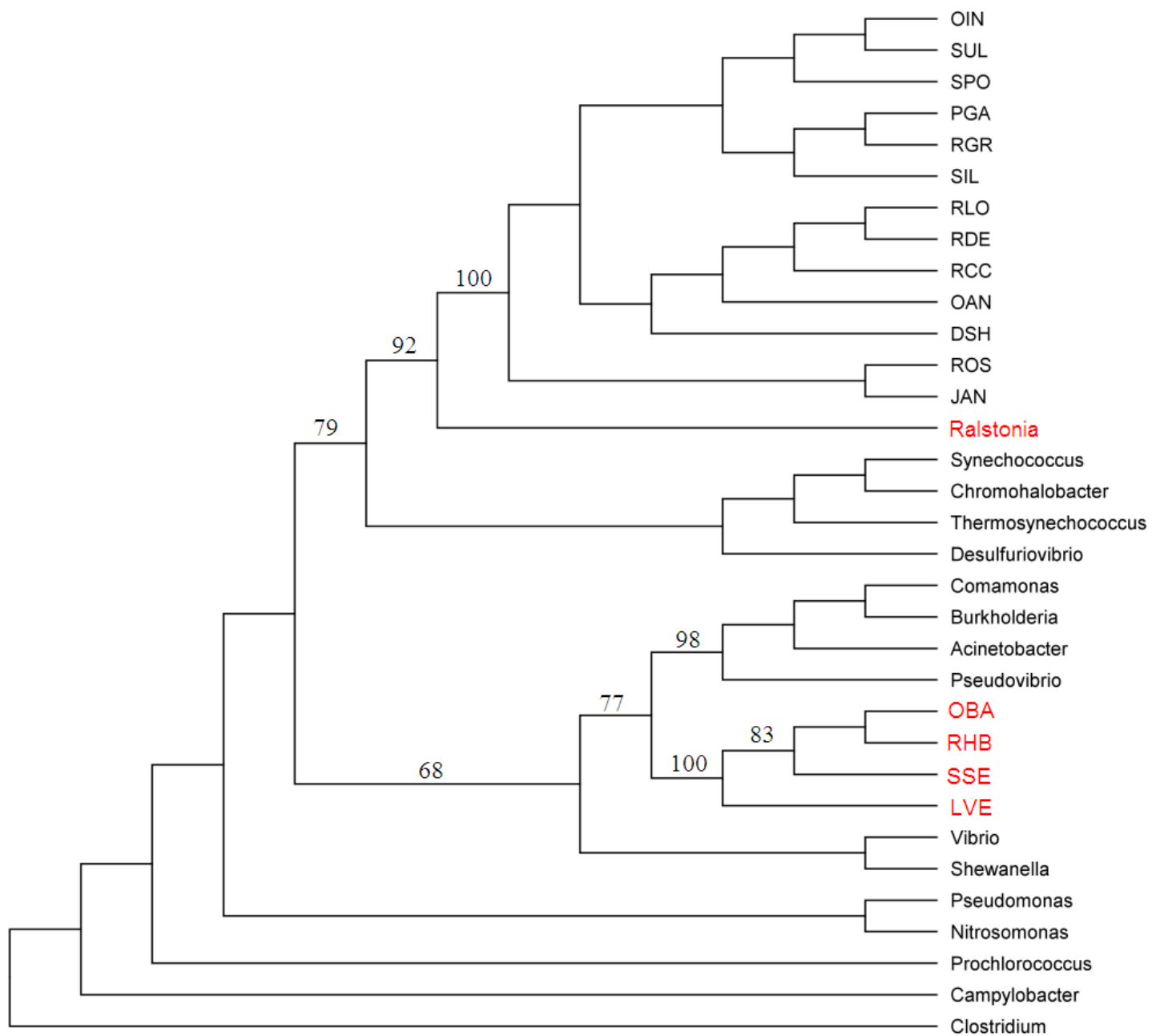

Supplement: File S2 — Tree topologies with the extended data. The multi-documents have been combined into a single ZIP-formatted file. The trees should be considered unrooted. The tree topologies were calculated in PhyML as described in Methods. Numbers refer to bootstrap values. The tree topology (separate pdf) shows that Roseobacter bacteria form a monophyletic group and was deposited in a document named “high bootstrap”. The other organisms embedded within the Roseobacter clade, or Roseobacter bacteria embedded within other phyla are shown in red (deposited in a document named “inter-phylum”). Individual file name corresponds to gene family code listed in Table S1. The non Roseobacter organism taxonomic name is detailed in the amino acid fasta of the sequences (a document named “sequences”). (4.62 MB ZIP) [file pone.0011604.s008.zip › inter-phylum/ort495.pdf]

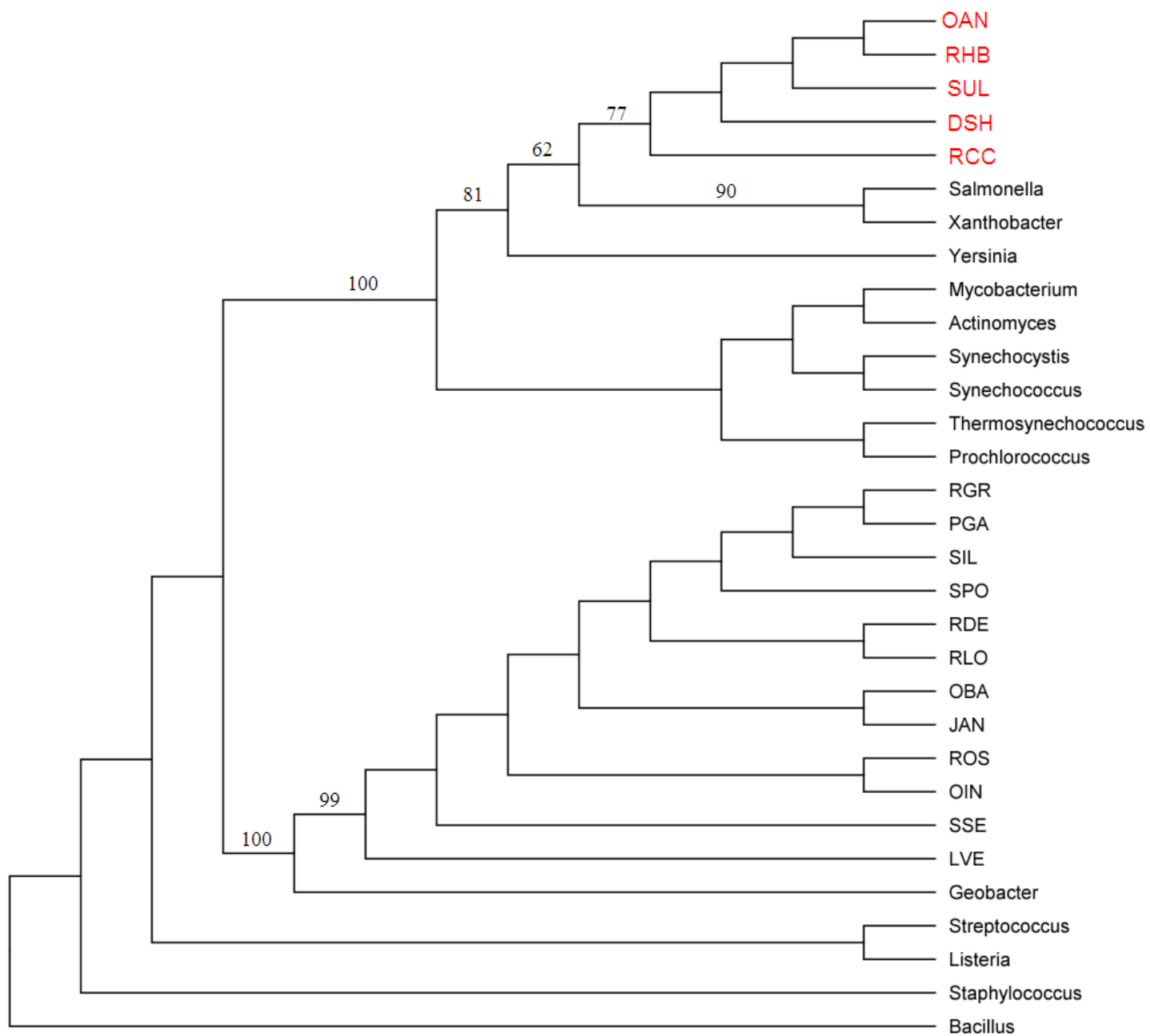

Supplement: File S2 — Tree topologies with the extended data. The multi-documents have been combined into a single ZIP-formatted file. The trees should be considered unrooted. The tree topologies were calculated in PhyML as described in Methods. Numbers refer to bootstrap values. The tree topology (separate pdf) shows that Roseobacter bacteria form a monophyletic group and was deposited in a document named “high bootstrap”. The other organisms embedded within the Roseobacter clade, or Roseobacter bacteria embedded within other phyla are shown in red (deposited in a document named “inter-phylum”). Individual file name corresponds to gene family code listed in Table S1. The non Roseobacter organism taxonomic name is detailed in the amino acid fasta of the sequences (a document named “sequences”). (4.62 MB ZIP) [file pone.0011604.s008.zip › inter-phylum/ort607.pdf]

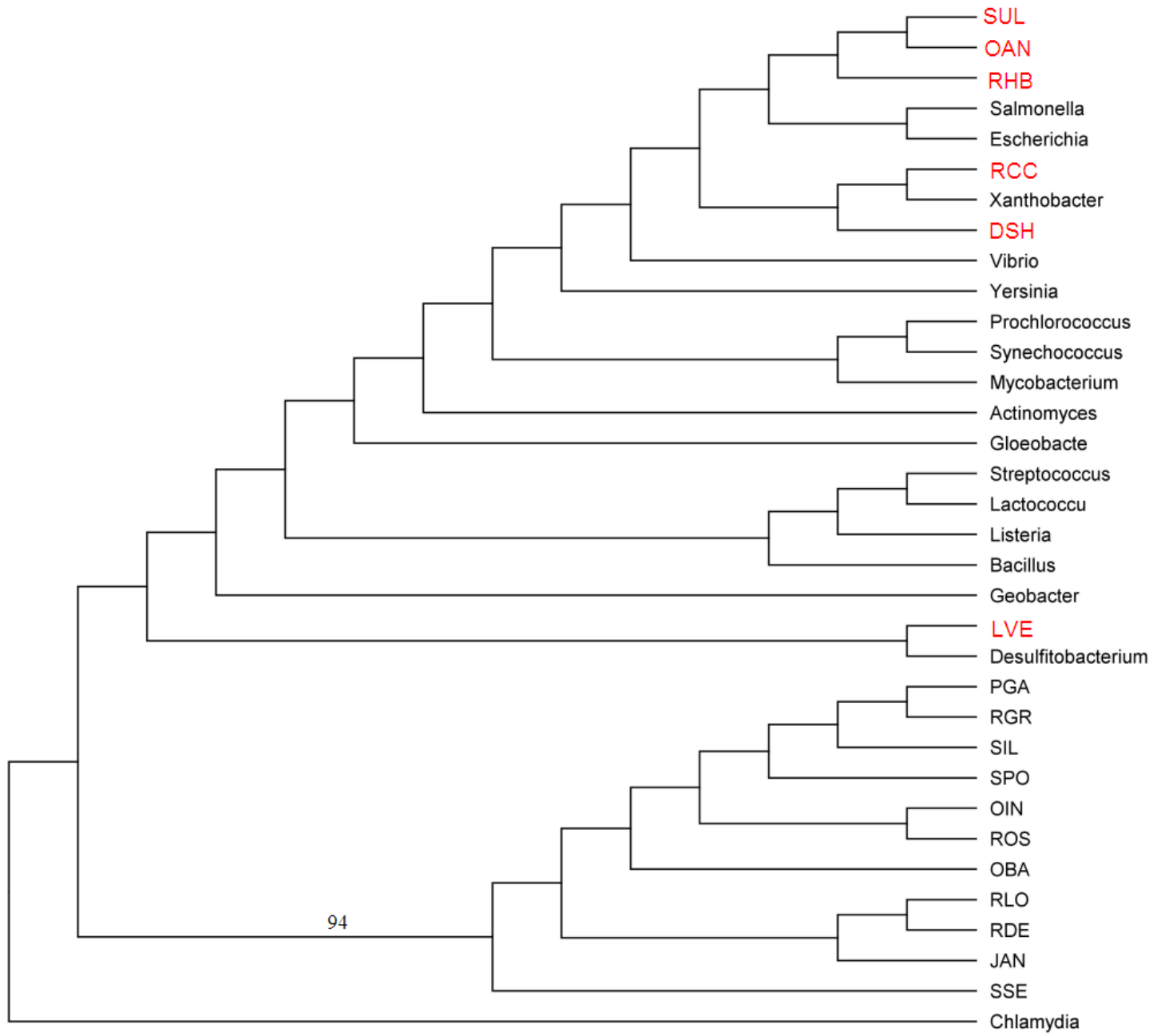

Supplement: File S2 — Tree topologies with the extended data. The multi-documents have been combined into a single ZIP-formatted file. The trees should be considered unrooted. The tree topologies were calculated in PhyML as described in Methods. Numbers refer to bootstrap values. The tree topology (separate pdf) shows that Roseobacter bacteria form a monophyletic group and was deposited in a document named “high bootstrap”. The other organisms embedded within the Roseobacter clade, or Roseobacter bacteria embedded within other phyla are shown in red (deposited in a document named “inter-phylum”). Individual file name corresponds to gene family code listed in Table S1. The non Roseobacter organism taxonomic name is detailed in the amino acid fasta of the sequences (a document named “sequences”). (4.62 MB ZIP) [file pone.0011604.s008.zip › inter-phylum/ort608.pdf]

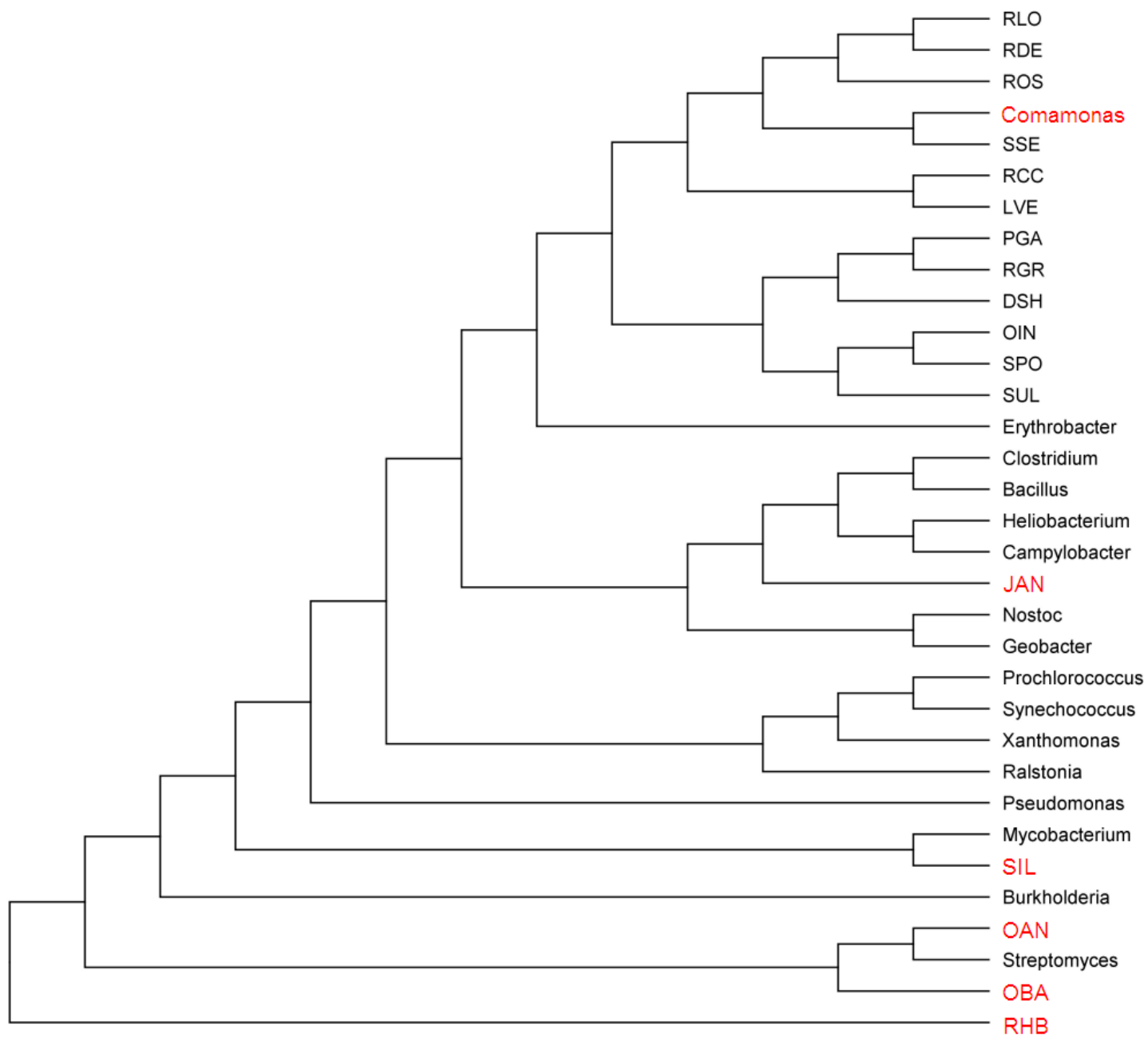

Supplement: File S2 — Tree topologies with the extended data. The multi-documents have been combined into a single ZIP-formatted file. The trees should be considered unrooted. The tree topologies were calculated in PhyML as described in Methods. Numbers refer to bootstrap values. The tree topology (separate pdf) shows that Roseobacter bacteria form a monophyletic group and was deposited in a document named “high bootstrap”. The other organisms embedded within the Roseobacter clade, or Roseobacter bacteria embedded within other phyla are shown in red (deposited in a document named “inter-phylum”). Individual file name corresponds to gene family code listed in Table S1. The non Roseobacter organism taxonomic name is detailed in the amino acid fasta of the sequences (a document named “sequences”). (4.62 MB ZIP) [file pone.0011604.s008.zip › inter-phylum/ort661.pdf]

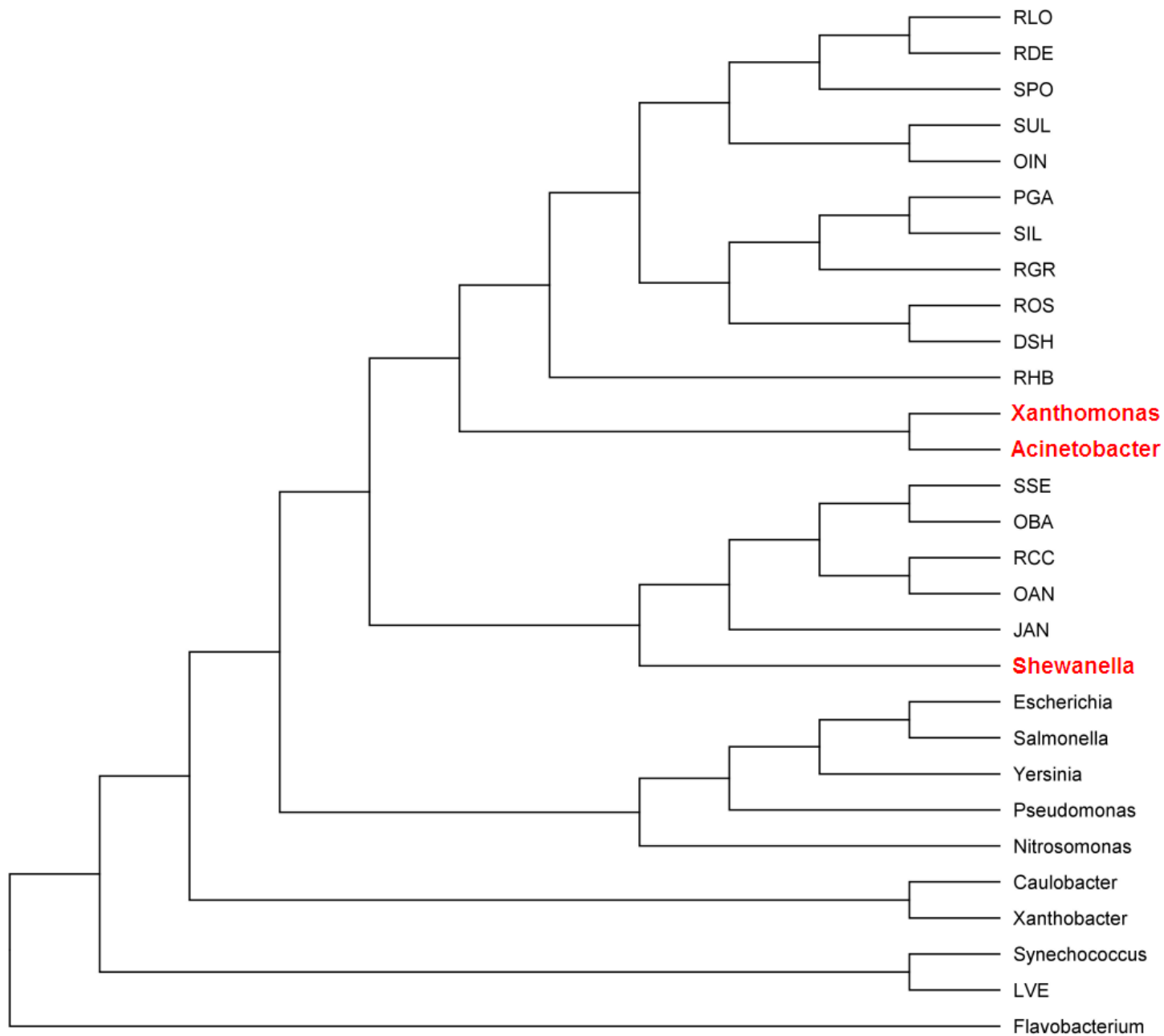

Supplement: File S2 — Tree topologies with the extended data. The multi-documents have been combined into a single ZIP-formatted file. The trees should be considered unrooted. The tree topologies were calculated in PhyML as described in Methods. Numbers refer to bootstrap values. The tree topology (separate pdf) shows that Roseobacter bacteria form a monophyletic group and was deposited in a document named “high bootstrap”. The other organisms embedded within the Roseobacter clade, or Roseobacter bacteria embedded within other phyla are shown in red (deposited in a document named “inter-phylum”). Individual file name corresponds to gene family code listed in Table S1. The non Roseobacter organism taxonomic name is detailed in the amino acid fasta of the sequences (a document named “sequences”). (4.62 MB ZIP) [file pone.0011604.s008.zip › inter-phylum/ort678.pdf]

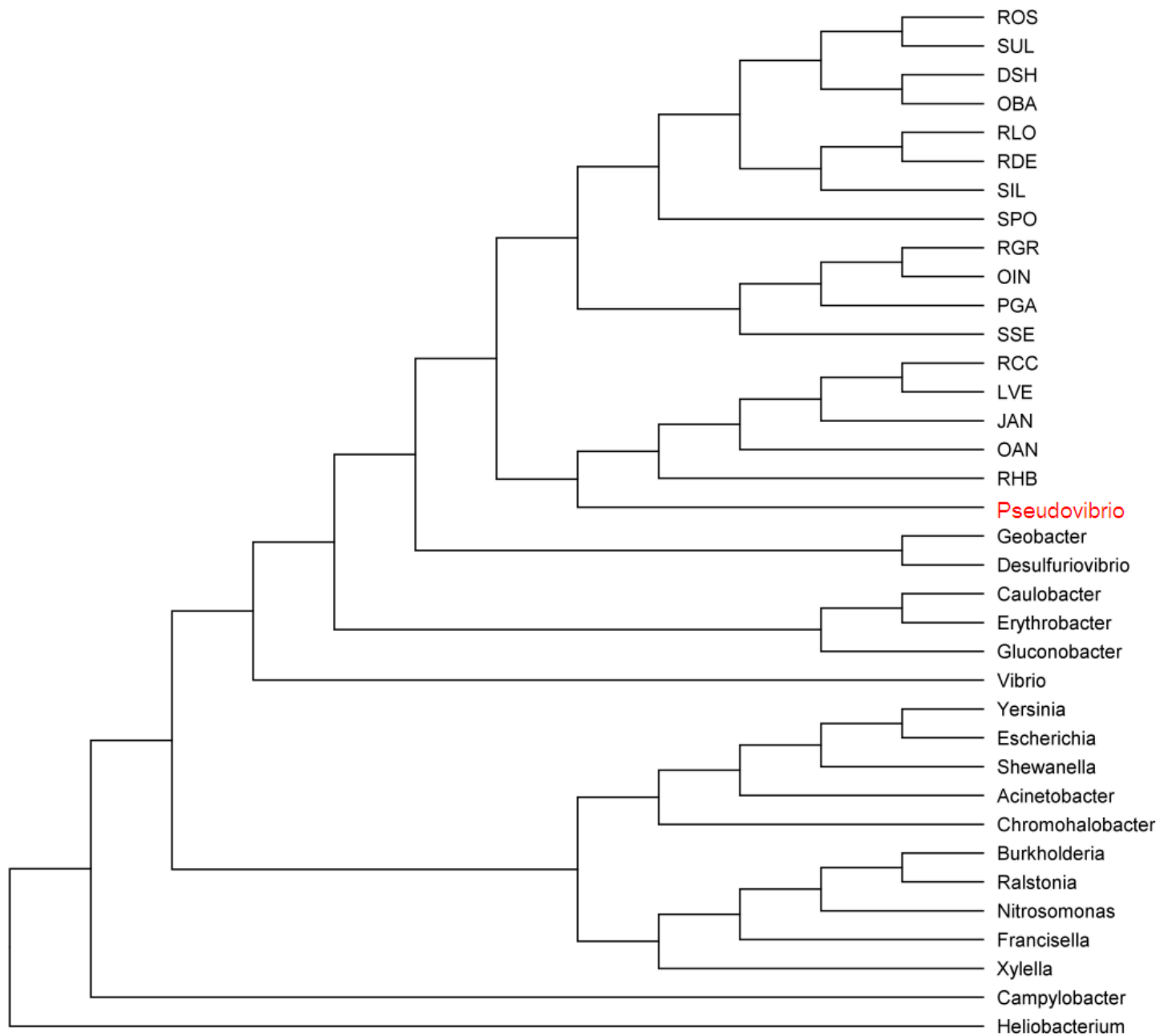

Supplement: File S2 — Tree topologies with the extended data. The multi-documents have been combined into a single ZIP-formatted file. The trees should be considered unrooted. The tree topologies were calculated in PhyML as described in Methods. Numbers refer to bootstrap values. The tree topology (separate pdf) shows that Roseobacter bacteria form a monophyletic group and was deposited in a document named “high bootstrap”. The other organisms embedded within the Roseobacter clade, or Roseobacter bacteria embedded within other phyla are shown in red (deposited in a document named “inter-phylum”). Individual file name corresponds to gene family code listed in Table S1. The non Roseobacter organism taxonomic name is detailed in the amino acid fasta of the sequences (a document named “sequences”). (4.62 MB ZIP) [file pone.0011604.s008.zip › inter-phylum/ort731.pdf]

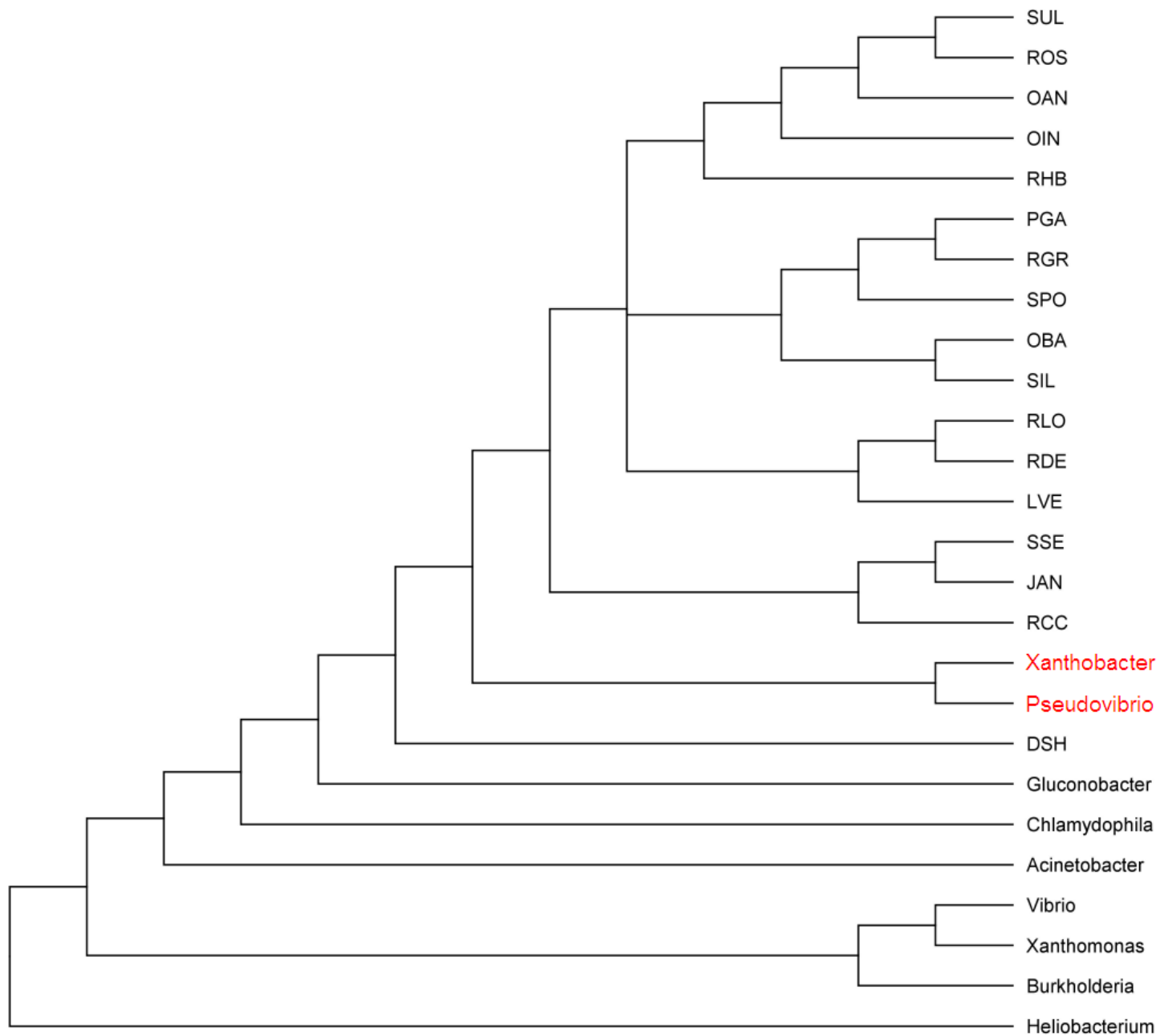

Supplement: File S2 — Tree topologies with the extended data. The multi-documents have been combined into a single ZIP-formatted file. The trees should be considered unrooted. The tree topologies were calculated in PhyML as described in Methods. Numbers refer to bootstrap values. The tree topology (separate pdf) shows that Roseobacter bacteria form a monophyletic group and was deposited in a document named “high bootstrap”. The other organisms embedded within the Roseobacter clade, or Roseobacter bacteria embedded within other phyla are shown in red (deposited in a document named “inter-phylum”). Individual file name corresponds to gene family code listed in Table S1. The non Roseobacter organism taxonomic name is detailed in the amino acid fasta of the sequences (a document named “sequences”). (4.62 MB ZIP) [file pone.0011604.s008.zip › inter-phylum/ort830.pdf]

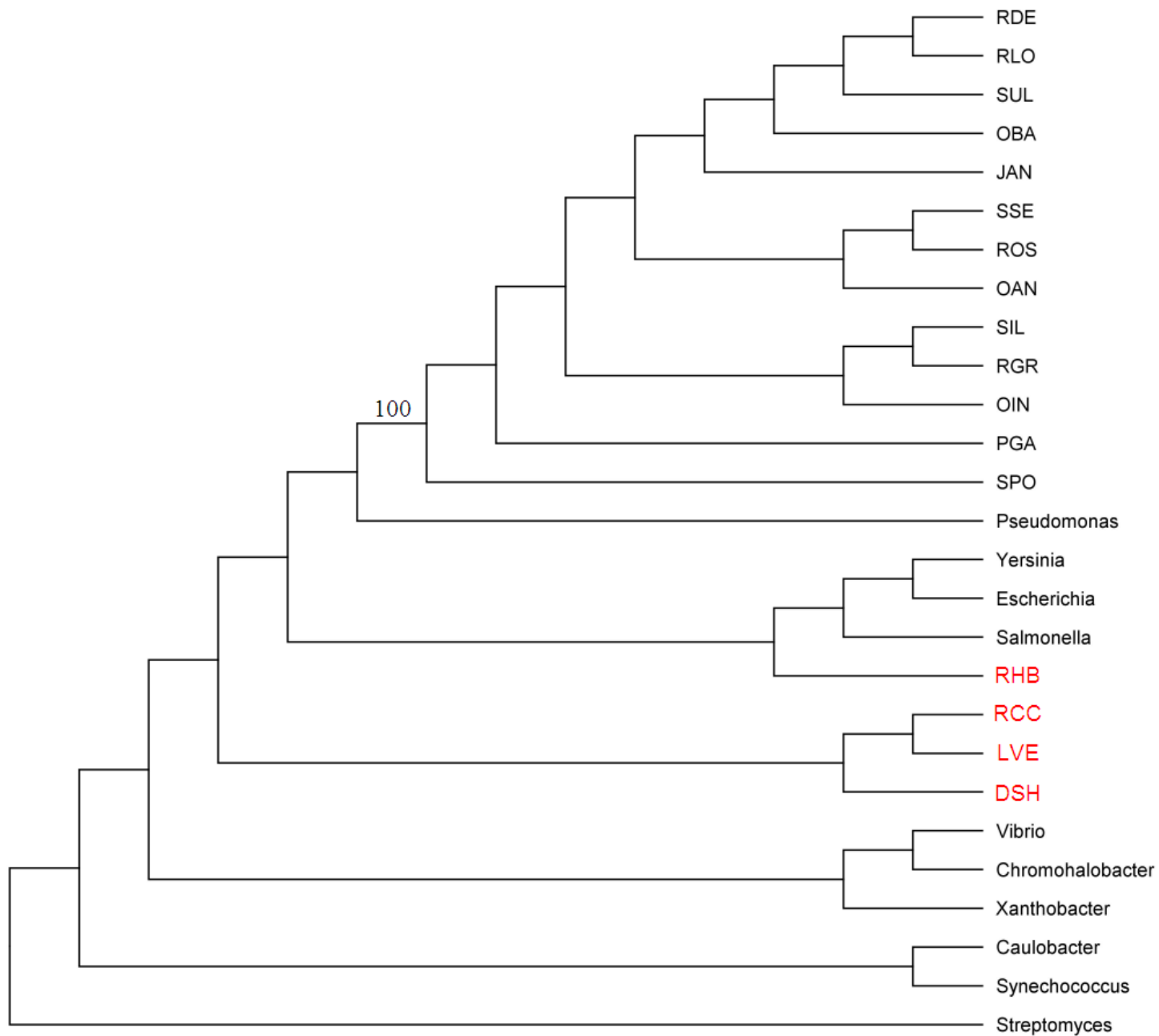

Supplement: File S2 — Tree topologies with the extended data. The multi-documents have been combined into a single ZIP-formatted file. The trees should be considered unrooted. The tree topologies were calculated in PhyML as described in Methods. Numbers refer to bootstrap values. The tree topology (separate pdf) shows that Roseobacter bacteria form a monophyletic group and was deposited in a document named “high bootstrap”. The other organisms embedded within the Roseobacter clade, or Roseobacter bacteria embedded within other phyla are shown in red (deposited in a document named “inter-phylum”). Individual file name corresponds to gene family code listed in Table S1. The non Roseobacter organism taxonomic name is detailed in the amino acid fasta of the sequences (a document named “sequences”). (4.62 MB ZIP) [file pone.0011604.s008.zip › inter-phylum/ort840.pdf]

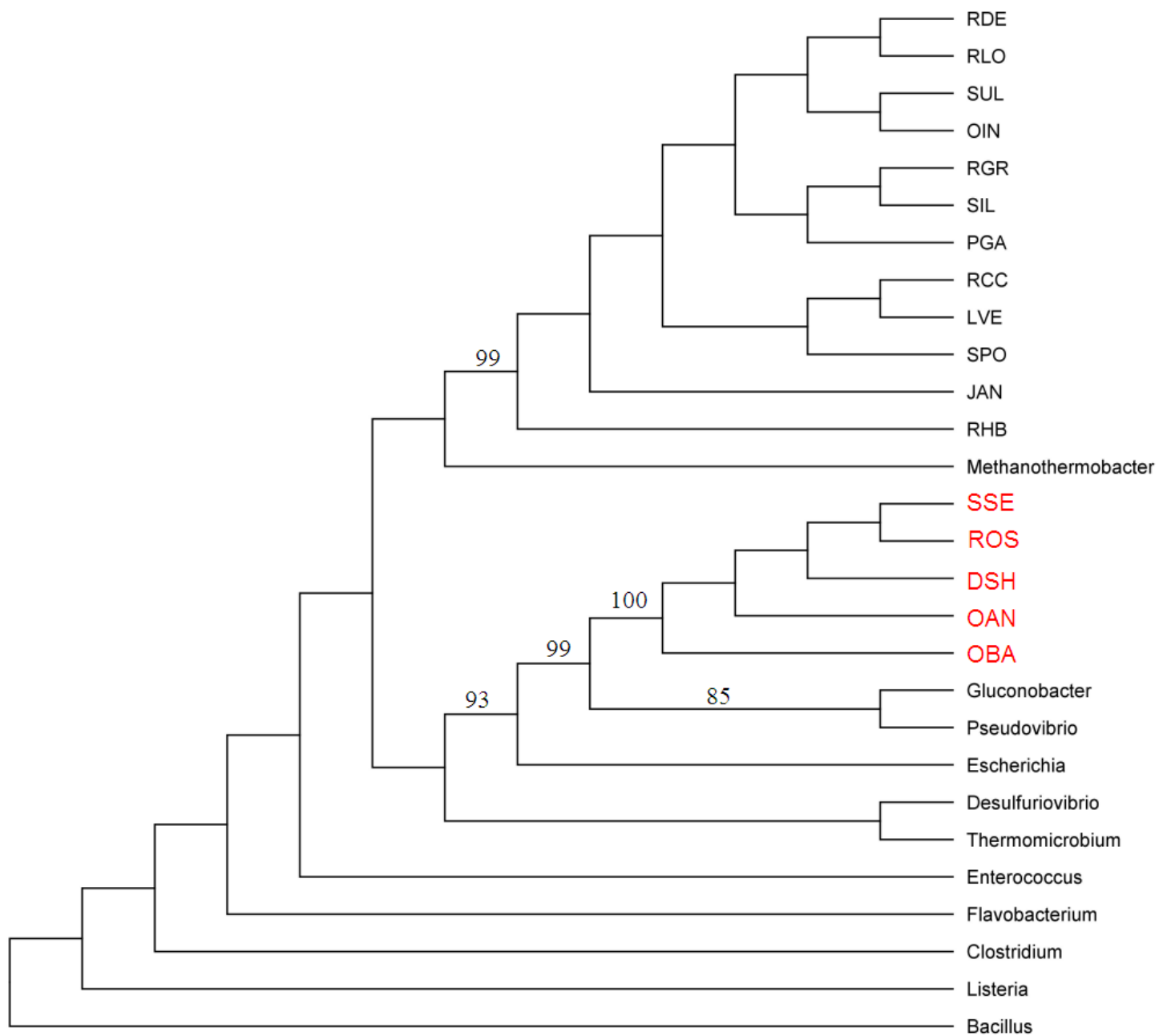

Supplement: File S2 — Tree topologies with the extended data. The multi-documents have been combined into a single ZIP-formatted file. The trees should be considered unrooted. The tree topologies were calculated in PhyML as described in Methods. Numbers refer to bootstrap values. The tree topology (separate pdf) shows that Roseobacter bacteria form a monophyletic group and was deposited in a document named “high bootstrap”. The other organisms embedded within the Roseobacter clade, or Roseobacter bacteria embedded within other phyla are shown in red (deposited in a document named “inter-phylum”). Individual file name corresponds to gene family code listed in Table S1. The non Roseobacter organism taxonomic name is detailed in the amino acid fasta of the sequences (a document named “sequences”). (4.62 MB ZIP) [file pone.0011604.s008.zip › inter-phylum/ort873.pdf]

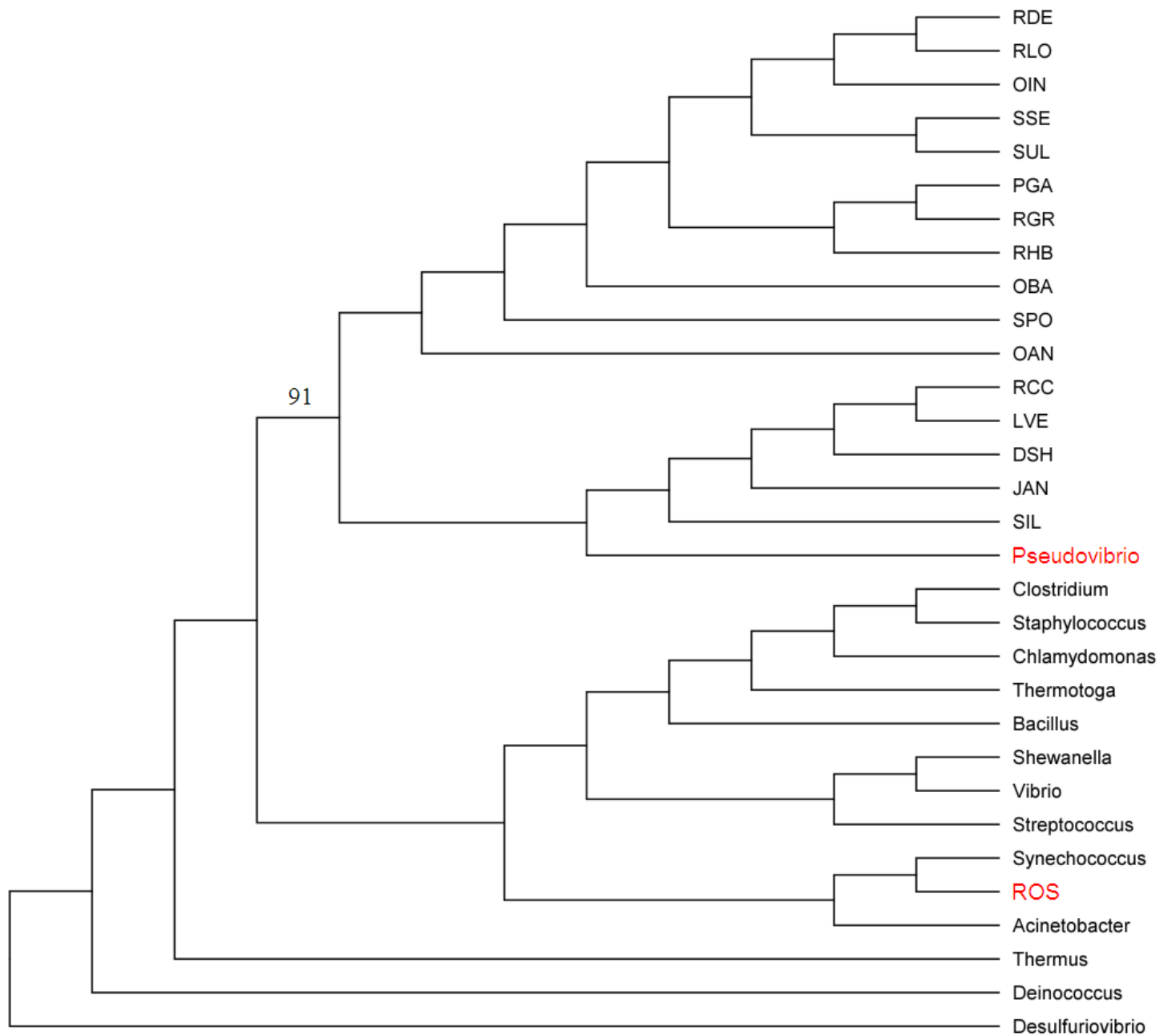

Supplement: File S2 — Tree topologies with the extended data. The multi-documents have been combined into a single ZIP-formatted file. The trees should be considered unrooted. The tree topologies were calculated in PhyML as described in Methods. Numbers refer to bootstrap values. The tree topology (separate pdf) shows that Roseobacter bacteria form a monophyletic group and was deposited in a document named “high bootstrap”. The other organisms embedded within the Roseobacter clade, or Roseobacter bacteria embedded within other phyla are shown in red (deposited in a document named “inter-phylum”). Individual file name corresponds to gene family code listed in Table S1. The non Roseobacter organism taxonomic name is detailed in the amino acid fasta of the sequences (a document named “sequences”). (4.62 MB ZIP) [file pone.0011604.s008.zip › inter-phylum/ort896.pdf]
