# Supplementary material for: Phylogenomic Analysis of Marine Roseobacters
Source: PLoS One. 2010 Jul 15;5(7):e11604. doi: 10.1371/journal.pone.0011604 (PMC2904699; doi:10.1371/journal.pone.0011604)
Supplement: Table S2 — Summary of the distribution of the COG functional categories of the likely orthologous genes in the Roseobacter clade. (0.06 MB DOC) [file pone.0011604.s005.doc]

Table S2 Summary of the distribution of the COG functional categories of the likely orthologous genes in the *Roseobacter* clade

|  | **Pathway Category** | **Number of genes** | **% of the total genes** |
| --- | --- | --- | --- |
| Information storage | J Translation, ribosomal structure and biogenesis | 138 | 11.54 |
| and processing | K Transcription | 64 | 5.35 |
|  | L Replication, recombination and repair | 65 | 5.43 |
| Cellular processes | O Posttranslational modification, protein turnover, chaperones | 69 | 5.77 |
| and signalling | D Cell cycle control, cell division, chromosome partitioning | 20 | 1.67 |
|  | M Cell wall/membrane/envelope biogenesis | 49 | 4.10 |
|  | U Intracellular trafficking, secretion, and vesicular transport | 18 | 1.51 |
|  | T Signal transduction mechanisms | 23 | 1.92 |
|  | V Defense mechanisms | 10 | 0.84 |
| Metabolism | C Energy production and conversion | 76 | 6.35 |
|  | G Carbohydrate transport and metabolism | 27 | 2.26 |
|  | E Amino acid transport and metabolism | 113 | 9.45 |
|  | F Nucleotide transport and metabolism | 46 | 3.85 |
|  | H Coenzyme transport and metabolism | 64 | 5.35 |
|  | I Lipid transport and metabolism | 55 | 4.60 |
|  | P Inorganic ion transport and metabolism | 36 | 3.01 |
|  | Q Secondary metabolites biosynthesis, transport and catabolism | 7 | 0.59 |
| Poorly characterized | R General function prediction only | 125 | 10.45 |
|  | S Function unknown | 87 | 7.27 |
|  | X Miscellaneous | 104 | 8.70 |

*All the photosynthetic genes were assigned to the energy storage and production category.

Genes having more than one designation were assigned to an additional category called "miscellaneous".

One orthologous gene can not be annotated with a COG.
