# Supplementary material for: Phylogenomic Analysis of Marine Roseobacters
Source: PLoS One. 2010 Jul 15;5(7):e11604. doi: 10.1371/journal.pone.0011604 (PMC2904699; doi:10.1371/journal.pone.0011604)
Supplement: Table S3 — Photosynthetic genes and DMSP degradation genes p−values for AU and SH tests against species tree. (0.07 MB DOC) [file pone.0011604.s006.doc]

Table S3 Photosynthetic genes and DMSP degradation genes p-values for AU and SH tests against species tree

| **Gene name** | **Protein name** | **p-SH** | **p-AU** |
| --- | --- | --- | --- |
| Photosynthetic genes |  |  |  |
| *puhE* | PuhE protein | 0.958 | 0.517 |
| *acsF* | Aerobic magnesium-protoporphyrin IX monomethyl ester [oxidative] cyclase (EC=1.14.13.81) | 0.872 | 0.091 |
| *puhC* | PuhC protein | 1.000 | 0.623 |
| *puhB* | Putative photosynthetic complex assembly protein | 0.996 | 0.832 |
| *puhA* | Reaction center protein H chain | 0.804 | 0.090 |
| *pucC* | PUCC protein | 0.904 | 0.359 |
| *bchM* | Magnesium-protoporphyrin O-methyltransferase (EC=2.1.1.11) | 0.855 | 0.086 |
| *bchL* | Light-independent protochlorophyllide reductase iron-sulfur ATP-binding protein (EC=1.18.-.-) | 0.204 | 0.229 |
| *bchH* | Magnesium-chelatase subunit H (in EC=6.6.1.2) | 0.997 | 0.292 |
| *bchB* | Light-independent protochlorophyllide reductase subunit B (in EC=1.18.-.-) | 0.999 | 0.702 |
| *bchN* | Light-independent protochlorophyllide reductase subunit N (in EC=1.18.-.-) | 0.849 | 0.189 |
| *bchF* | 2-vinyl bacteriochlorophyllide hydratase (EC=4.2.1.-) | 0.993 | 0.781 |
| *ppaA* | Regulatory protein PpaA | 0.913 | 0.229 |
| *ppsR* | Transcriptional regulator PpsR | 0.643 | 0.006 |
| *bchG* | Bacteriochlorophyll/chlorophyll synthetase (EC=2.5.1.62) | 1.000 | 0.656 |
| *pucC2* | PUCC protein | 0.258 | 0.010 |
| *pufM* | Reaction center protein M chain | 0.918 | 0.111 |
| *pufL* | Reaction center protein L chain | 0.221 | 0.013 |
| *pufA* | Light-harvesting protein B-870 alpha chain | 0.413 | 0.139 |
| *pufB* | Light-harvesting protein B-870 beta chain | 0.580 | 0.014 |
| *pufQ* | Protein pufQ | 0.995 | 0.744 |
| *bchZ* | Bacteriachlorophyllide reductase iron protein subunit Z (in EC=1.3.1.33) | 0.702 | 0.039 |
| *bchY* | Bacteriachlorophyllide reductase iron protein subunit Y (in EC=1.3.1.33) | 0.997 | 0.718 |
| *bchX* | Bacteriachlorophyllide reductase iron protein subunit X (in EC=1.3.1.33) | 0.596 | 0.028 |
| *bchC* | 2-desacetyl-2-hydroxyethyl bacteriochlorophyllide A dehydrogenase (EC=1.-.-.-) | 0.876 | 0.084 |
| *crtE* | Geranylgeranyl pyrophosphate synthetase (EC=2.5.1.29) | 1.000 | 0.586 |
| *crtD* | Methoxyneurosporene dehydrogenase (EC=1.14.99.-) | 0.959 | 0.379 |
| *crtC* | Hydroxyneurosporene dehydrogenase (EC=1.-.-.-) | 0.998 | 0.753 |
| *tspO* | Tryptophan rich sensory protein | 1.000 | 0.628 |
| *bchO* | Magnesium-chelatase 30 kDa subunit (in EC=6.6.1.1) | 0.752 | 0.123 |
| *bchD* | Magnesium-chelatase 60 kDa subunit (in EC=6.6.1.1) | 0.998 | 0.721 |
| *bchI* | Magnesium-chelatase 38 kDa subunit (in EC=6.6.1.1) | 0.864 | 0.256 |
| *crtA* | Spheroidene monooxygenase | 0.946 | 0.544 |
| DMSP degradation genes |  |  |  |
| *dmdA* | Dimethylsulfoniopropionate demthylase | 0.017 | 0.010 |
| *dddL* | Dimethylsulfoniopropionate lyase | 0.001 | 0.000 |
