# Supplementary figures and images for: Phylogenomic Analysis of Marine Roseobacters (part 2 of 2)
Source: PLoS One. 2010 Jul 15;5(7):e11604. doi: 10.1371/journal.pone.0011604 (PMC2904699; doi:10.1371/journal.pone.0011604)

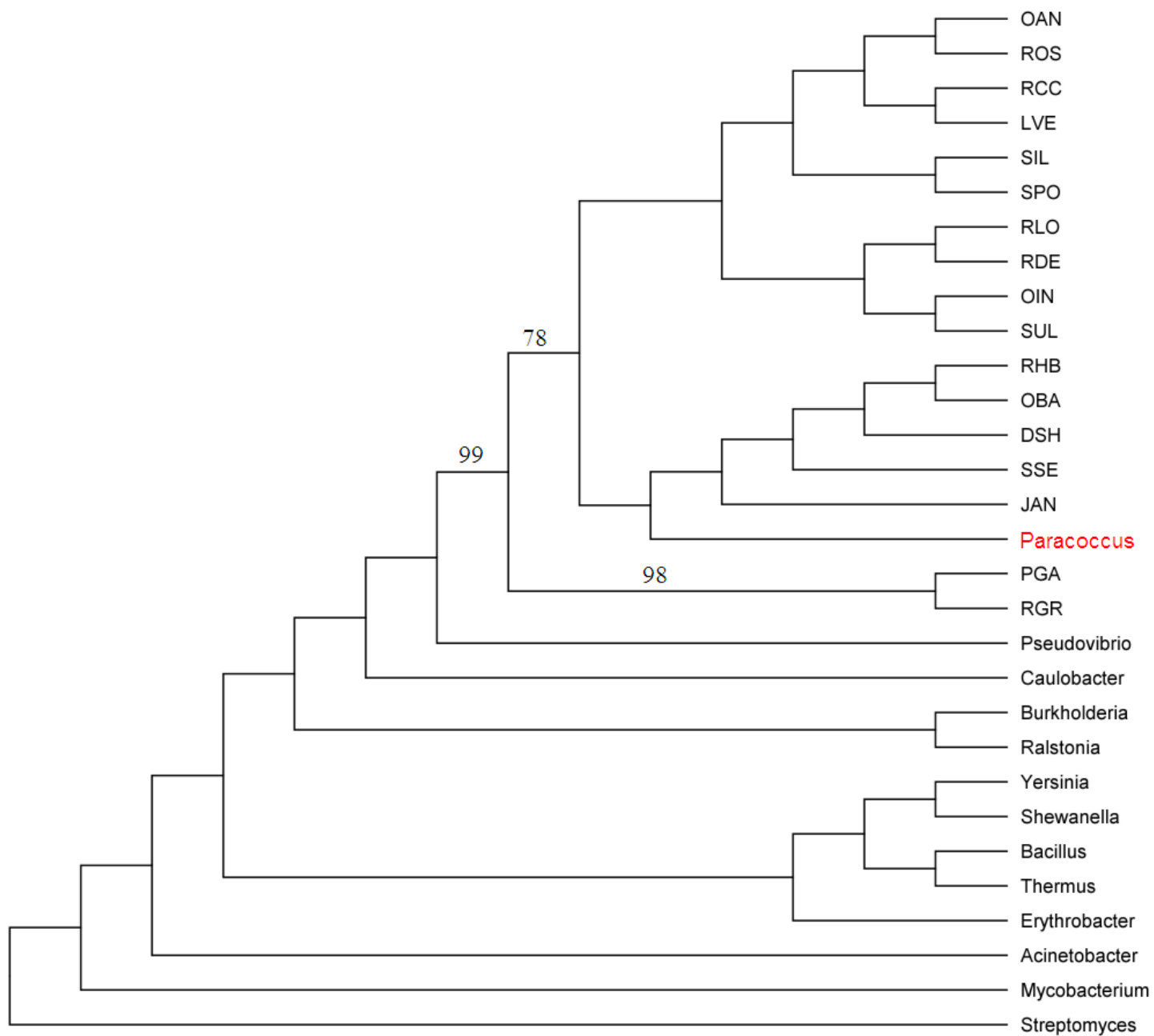

Supplement: File S2 — Tree topologies with the extended data. The multi-documents have been combined into a single ZIP-formatted file. The trees should be considered unrooted. The tree topologies were calculated in PhyML as described in Methods. Numbers refer to bootstrap values. The tree topology (separate pdf) shows that Roseobacter bacteria form a monophyletic group and was deposited in a document named “high bootstrap”. The other organisms embedded within the Roseobacter clade, or Roseobacter bacteria embedded within other phyla are shown in red (deposited in a document named “inter-phylum”). Individual file name corresponds to gene family code listed in Table S1. The non Roseobacter organism taxonomic name is detailed in the amino acid fasta of the sequences (a document named “sequences”). (4.62 MB ZIP) [file pone.0011604.s008.zip › inter-phylum/ort906.pdf]
